# Targeted Protein Degradation of NUDT5 Dissociates Catalytic Inhibition from Protein Loss in 6-Thioguanine Response

**DOI:** 10.1038/s41467-026-74489-9

**Published:** 2026-06-30

**Authors:** Anne-Sophie M. C. Marques, Ludwig G. Bauer, Tuan-Anh Nguyen, Alejandro Gonzalez Orta, Jan-Lennart Venne, Carol Cheng, Esra Balıkçı, Yusi Liu, Barr Tivon, Alena Kroupova, Alessio Ciulli, Nir London, Stefan Kubicek, Kilian V. M. Huber

**Affiliations:** 1 Centre for Medicines Discovery, Nuffield Department of Medicine, University of Oxford, Old Road Campus, Roosevelt Drive, Oxford, UK; 2 Target Discovery Institute, Nuffield Department of Medicine, University of Oxford, Old Road Campus, Roosevelt Drive, Oxford, UK; 3 CeMM Research Center for Molecular Medicine of the Austrian Academy of Sciences, Vienna, Austria; 4 Department of Chemical and Structural Biology, The Weizmann Institute of Science, Rehovot, Israel; 5 Centre for Targeted Protein Degradation, School of Life Sciences, University of Dundee, 1 James Lindsay Place, DD1 5JJ Dundee, UK

**Keywords:** Chemical tools, Mechanism of action

## Abstract

6-Thioguanine (6-TG) is an FDA-approved antimetabolite drug that is widely used clinically, including for the treatment of leukemia. Its cellular effects require metabolic activation and are regulated through interactions with various proteins such as NUDT15, which catalyzes the hydrolysis of the active 6-TG metabolites 6-thio-deoxyGTP (6-thio-dGTP) and 6-thio-GTP. Recent genome-wide CRISPR loss-of-function studies have identified another NUDIX hydrolase, NUDT5, as a crucial mediator of 6-TG toxicity. Here, we develop and validate a selective, cell-active NUDT5 degrader toolkit and orthogonally characterize target engagement, ternary complex formation, degradation kinetics, and proteome-wide selectivity. These degraders, in conjunction with orthogonal CRISPR knock-out and reconstitution experiments, support a non-enzymatic role for NUDT5 in modulating the cellular response to 6-TG. Depletion of NUDT5 protein is antagonistic to NUDT15 inhibition, suggesting a distinct mode-of-action with potential implications for patient therapy.

## Abstract (web summary)

Thiopurines are widely used drugs whose activity depends on cellular metabolism. Here, the authors develop chemical tools to remove NUDT5 and show that protein loss, but not inhibition, alters drug response, revealing a non-enzymatic role with implications for therapy.

## Introduction

NUDT5 is a conserved NUDIX hydrolase that cleaves the pyrophosphate bond within a range of nucleotide-derived substrates such as ADP-ribose, ADP-glucose, and GDP-glucose^1^. Among the 22 human NUDIX proteins, NUDT5 plays a key role in ATP metabolism and chromatin remodeling, thereby conferring a potential vulnerability in hormone-dependent breast cancer^2,3^. Consistent with these data, NUDT5 knockdown suppresses migration and colony formation of T47D cells^1,3^. Recent reports suggest NUDT5 as a potential target in triple-negative breast cancer (TNBC) where its expression correlates with poor prognosis^4–6^. In addition to its role in cancer^7–9^, NUDT5 has been found to be an essential factor for modulating the toxicity of 6-thioguanine (6-TG)^10,11^. Introduced in the 1950s, 6-TG has been utilized as an antimetabolite drug to treat leukemia, autoimmune conditions, and inflammatory bowel disease (IBD) both in adults and children^12^. Mechanistically, 6-TG is thought to act as a prodrug, undergoing a cascade of metabolic conversions before being incorporated into DNA or RNA to exhibit its phenotypic effects. While NUDT5 inhibitors have been reported^1^, the impact of NUDT5 catalytic inhibition on 6-TG-mediated toxicity remains elusive, particularly in relation to the resistance phenotype observed upon genetic loss of NUDT5. To address this gap, we leverage an event-driven chemical biology strategy to resolve a discrepancy between pharmacological inhibition and genetic loss in thiopurine response. We report the development of a selective NUDT5 degrader (dNUDT5) alongside a matched inactive control (dNUDT5nc) and validate these tools using orthogonal cellular and biochemical assays, including live-cell engagement assays and ternary complex formation readouts, degradation mechanism controls, proteome-wide selectivity profiling, and structural characterization. Using these probes together with genetic knockout and catalytic-inactive reconstitution, we demonstrate that reduction of NUDT5 protein abundance is sufficient to govern 6-TG sensitivity in cells, whereas catalytic inhibition alone is not. We present a working model, supported by metabolomic trends and phenotype triangulation, for how NUDT5 loss shifts nucleotide metabolism in a manner that reduces susceptibility to thiopurine challenge and is distinct from the established role of NUDT15 as a genetic determinant and patient biomarker for 6-TG sensitivity.

## Results

### NUDT5 catalytic inhibition does not phenocopy genetic loss in 6-thioguanine response

**Fig. 1:**
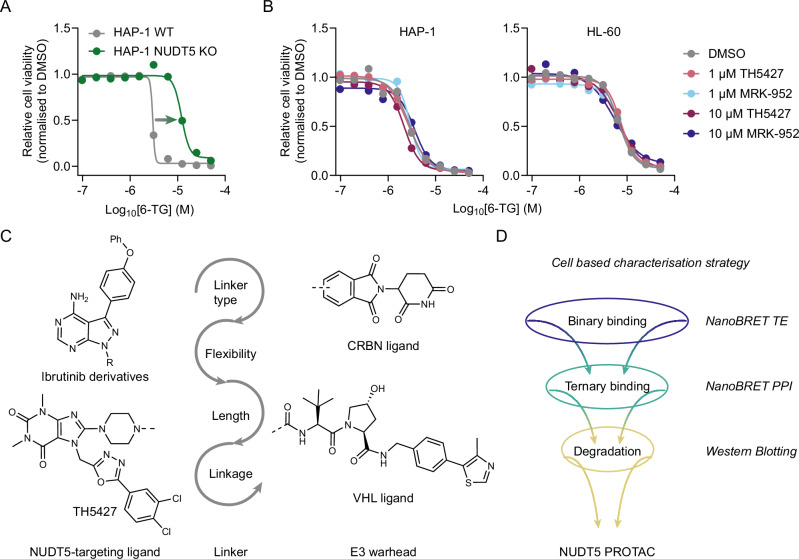
NUDT5 enzymatic activity is dispensable for 6-TG-mediated cancer cell cytotoxicity. **A** NUDT5 KO de-sensitizes HAP1 leukemia cells towards 6-TG (KO, knock-out; WT, wild-type). Data are shown as mean values derived from three technical replicates. Graph is representative of two independent biological replicates (*n* = 2). **B** Inhibition of NUDT5 enzymatic activity fails to rescue 6-TG-mediated toxicity in HAP1 and HL-60 cells. Data are shown as mean values derived from three technical replicates. Graph is representative of two independent biological replicates (*n* = 2). **C** NUDT5 PROTAC design strategy. **D** Cell-based assay screening cascade for development of NUDT5 PROTACs (TE, target engagement: PPI, protein-protein interaction).

In a previous CRISPR genome-wide loss-of-function screen conducted in HEK293T, HT29, and A375 cells, NUDT5 was identified as a crucial mediator of 6-TG toxicity^10^. Given the widespread use of 6-TG in leukemia treatment, we confirmed that NUDT5 knockout (NUDT5 KO) confers resistance in HAP1 cells, a Philadelphia chromosome-positive, near-haploid human cell line derived from KBM-7 chronic myelogenous leukemia (CML) cells. We observed a four-fold increase in 6-TG IC_50_ values when comparing NUDT5 wild-type (WT) versus NUDT5 KO cells (Fig. 1A). Next, we tested the published NUDT5 inhibitor TH5427 as well as a novel, chemically distinct NUDT5 chemical probe, MRK-952 (https://thesgc.org/chemical-probes/mrk-952, to be published) for their ability to desensitize HAP1 cells to 6-TG treatment. Contrary to expectations, neither inhibitor was able to phenocopy the NUDT5 KO in HAP1 or HL-60 cells (Fig. 1B). These results suggest that suppression of NUDT5 enzymatic activity alone is insufficient to rescue cells from 6-TG-mediated toxicity. Intrigued by these findings, we decided to further dissect the role of NUDT5 in this context using a targeted protein degradation (TPD) strategy. We envisioned linking established NUDT5 ligands to E3 ligase moieties to generate proteolysis targeting chimeras (PROTACs), which would allow differentiation between enzymatic and potential non-enzymatic functions of NUDT5 (Fig. 1C).

### Rational development of NUDT5 degraders to probe the 6-thioguanine response

We previously reported that ibrutinib, an FDA-approved BTK inhibitor, engages NUDT5 in cells^13^. This finding, in conjunction with the fact that multiple series of ibrutinib-based BTK PROTACs have been published^14,15^, prompted us to investigate if any such compounds might be able to also degrade NUDT5. Close inspection of co-crystal structures of ibrutinib bound to kinases compared to our recent NUDT5 complexes supported this hypothesis. We identified the N-3 and N-4 piperidine nitrogen in ibrutinib and compound 9 (^ref. 13^), respectively, as potential solvent-exposed exit vectors for further functionalization (Supplementary Fig. 1). Next, we synthesized a library of VHL-based PROTACs featuring various PEG- and carbon-based linkers (Supplementary Information) and complemented this set with additional published ibrutinib-based degraders including the commercial MT-802 (ref. ^14^) and previously reported CRBN-targeting BTK degraders^15^. We assessed the performance of all PROTACs via a cell-based screening cascade surveying binary NUDT5 target engagement (TE) followed by ternary complex formation assays monitoring the interaction between NUDT5 and the respective E3 ligase using intact cell NanoBRET technology. Only active compounds were then further evaluated for NUDT5 degradation by Western Blotting (Fig. 1D). However, although some ibrutinib-derived PROTACs were able to engage NUDT5 in cells, none of them appeared to induce formation of a productive E3-target ternary complex (Supplementary Table 1).

**Fig. 2:**
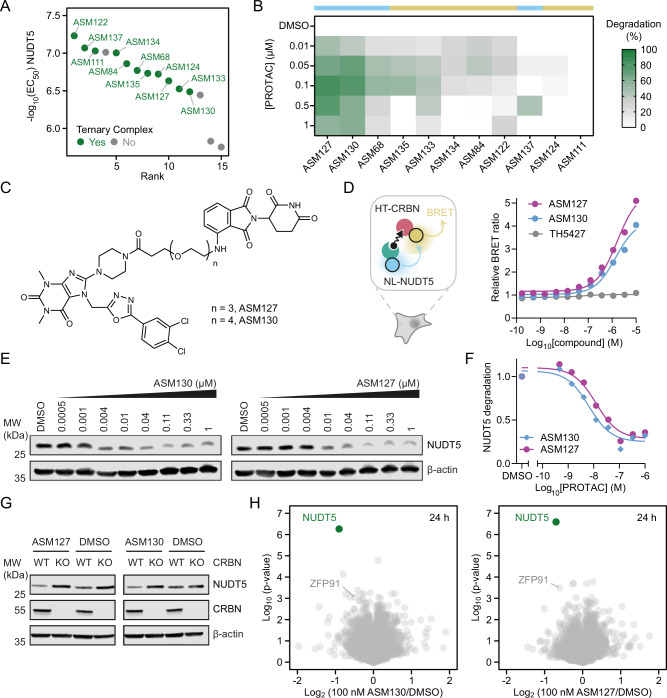
Characterization of the NUDT5 degraders ASM127 and ASM130. **A** Multiparametric ranking of PROTACs based on binary NUDT5 target engagement and compound-induced NUDT5-E3 ternary complex formation in HEK293 cells. **B** Heat map summary of PROTAC degradation efficiency across five concentrations after 20 h treatment in HEK293 cells. Yellow: PEG-based linker, blue: carbon-based linker. Degradation was calculated from normalized Western blot intensities as the mean from two independent biological replicates (*n* = 2). **C** Chemical structures of ASM127 and ASM130. **D** NanoBRET ternary complex formation assay results for ASM127, ASM130, and TH5427 using NanoLuc-NUDT5 (NL) and HaloTag-CRBN (HT) after 4 h treatment in HEK293 cells (BRET, bioluminescence resonance energy transfer). Data are shown as mean values derived from three technical replicates. Graph is representative of two independent biological replicates (*n* = 2). **E** Western blot showing dose-dependent degradation of NUDT5 in HEK293 cells after 20 h incubation with indicated compounds. Blot is representative of two independent biological replicates (*n* = 2). **F** Half-maximal degradation concentration (DC_50_) value determination for ASM127 and ASM130 (12 nM and 7 nM, respectively); estimated maximal degradation concentration (D_max_) 71% and 75%, respectively. Graphs show the mean normalized NUDT5 degradation efficiency, derived from Western blot band intensities, across two independent biological replicates (*n* = 2). **G** CRBN knockout (KO) prevents PROTAC-mediated degradation of NUDT5 in HEK293 cells after 20 h of treatment with 100 nM ASM127 or ASM130 (WT, wild-type). Western blot is representative of two biological replicates (*n *= 2). **H** Total proteome analysis results of HEK293 cells treated with 100 nM ASM127 or 100 nM ASM130 for 24 h. *P* values were determined by a two-sided unpaired t-test (*n* = 4 per group).

Next, we focused our synthetic strategy on the highly potent NUDT5 inhibitor TH5427 as a potential warhead for PROTACs^1^ (Fig. 1C). We obtained 15 bifunctional compounds with different linkers targeting either VHL or CRBN which we subsequently triaged in our cell-based screening cascade. While most compounds seemed to potently engage NUDT5, only CRBN-recruiting degraders efficiently promoted ternary complex formation after 4 h or 24 h (Fig. 2A and Supplementary Table 2). We assessed degradation for the most active compounds by Western blotting across five concentrations (Fig. 2B and Supplementary Fig. 2). Overall, we observed that NUDT5 cellular engagement is not a primary determinant for degradation as many potent NUDT5 binders exhibited no significant effects on NUDT5 protein levels (e.g. ASM111, ASM122, ASM137). Alkyl linker-based PROTACs only induced moderate degradation independent of linker length, whereas for PEG-based compounds longer linkers were associated with higher D_max_ (DC_50_: ASM135 < ASM68 < ASM127 < ASM130) (Fig. 2B). For the most potent and efficient NUDT5 degraders from this series, ASM127 and ASM130, harboring three and four PEG units in the linker, respectively (Fig. 2C, D), we determined DC_50_ values of 12 nM and 7 nM, and apparent D_max_ values of 71% and 75%, respectively (Fig. 2E, F). No effects on NUDT5 protein levels were observed in HEK293 CRBN^KO^ cells, indicating a CRBN-dependent degradation mechanism (Fig. 2G). Global proteomic analysis of HEK293 cells treated with either ASM127 or ASM130 revealed NUDT5 as the most significantly downregulated protein across 7135 quantified proteins (Fig. 2H). The data also suggested a slight effect on the zinc finger protein ZFP91, a common off-target of PROTACs featuring an ortho-substituted phthalimide^16^. To rationalize the observed effects on NUDT5, we conducted ProsettaC^17^ analyses, which provided plausible models, suggesting a similar binding mode for the active PEG-linker compounds (ASM68, ASM127, and ASM130) (Fig. 2D and Supplementary Fig. 3).

**Fig. 3:**
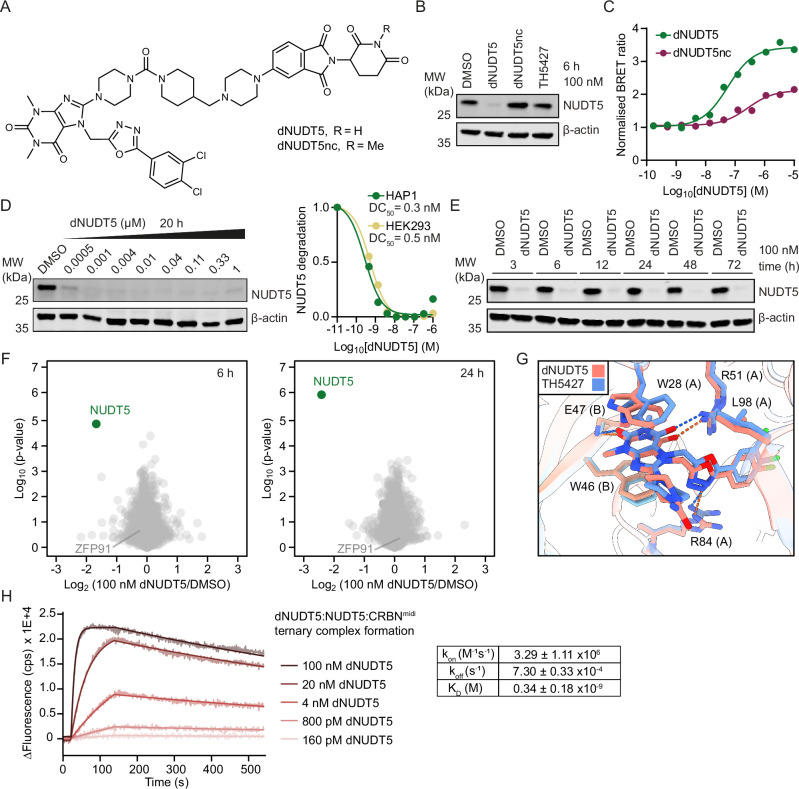
dNUDT5 induces target degradation through ternary complex formation and selective engagement of NUDT5. **A** Chemical structures of dNUDT5 and dNUDT5nc. **B** Western blot showing that dNUDT5, but not the methylated imide dNUDT5nc, is able to degrade NUDT5 in HAP1 cells. Blot is representative of two independent biological replicates (*n* = 2). **C** NanoBRET assay results demonstrating induced ternary complex formation by dNUDT5 in intact HEK293 cells. Data are shown as mean values derived from three technical replicates. Graph is representative of two independent biological replicates (*n* = 2). **D** Western blot showing dose-dependent NUDT5 degradation by dNUDT5 in HAP1 and HEK293 cells (DC_50_, half-maximal degradation concentration; D_max_, maximal degradation concentration). Blot is representative of two independent biological replicates (*n* = 2). **E** Western blot showing time-resolved NUDT5 degradation by dNUDT5 (100 nM) in HAP1 cells. Blot is representative of two independent biological replicates (*n* = 2). **F** Total proteome analysis of HAP1 cells treated with dNUDT5 for 6 h and 24 h, respectively. P values were determined by a two-sided unpaired t-test (*n* = 3 per group). **G** Superimposition of dNUDT5 (pink, PDB: 9G6Y) and TH5427 (blue) NUDT5 co-crystal structures (PDB: 5NWH). **H** HeliX proximity binding assay showing ternary complex formation mediated by dNUDT5 in vitro. The reported kinetic parameters (k_on_, k_off_) and dissociation constant (K_D_) values are the mean ± SEM from three independent measurements (*n* = 3).

In an attempt to further improve degradation efficiency and limit any potential effect on ZFP91, we next turned our attention to more rigid linkers which have proven efficient in increasing therapeutic potency and metabolic stability^18,19^. The majority of new PROTACs from this series exhibited only modest effects with regard to NUDT5 degradation (Supplementary Table 3 and Supplementary Fig. 4). However, we identified one compound, hereafter referred to as dNUDT5 (Fig. 3A), featuring a tetrasubstituted urea linker motif, which induced robust degradation of endogenous NUDT5 protein (Fig. 3B and Supplementary Fig. 5). To support further mechanistic studies, we synthesized a corresponding negative control, dNUDT5nc (Fig. 3A), by *N*-methylation of the imide ring to attenuate CRBN binding, thus preventing NUDT5 degradation (Fig. 3B and Supplementary Fig. 6A). Both dNUDT5 and dNUDT5nc engaged their cognate target NUDT5 in live cells as confirmed by NanoBRET assays (Supplementary Fig. 6B). As expected, only dNUDT5 induced proficient ternary complex formation between NUDT5 and CRBN in cells (Fig. 3C). We observed strong NUDT5 degradation in a concentration-dependent manner both in HAP1 cells (DC_50_ = 0.3 nM), as well as HEK293 cells (DC_50_ = 0.5 nM) (Fig. 3D and Supplementary Fig. 6C). Notably, concentrations above 300 nM appeared to result in a slight recovery of protein levels, likely due to the hook effect commonly observed with PROTACs^20,21^. We conducted kinetic experiments indicating sustained degradation by dNUDT5 over a time course of 72 h (Fig. 3E and Supplementary Fig. 6D). Furthermore, we observed robust and consistent NUDT5 downregulation by dNUDT5 across a panel of cell lines such as A549, MCF7, and HAP1 representing different tissue backgrounds (Supplementary Fig. 7). We confirmed that degradation by dNUDT5 is UPS-dependent by performing a series of rescue experiments using neddylation, proteasome, and NUDT5 inhibitors as well as CRBN KO in HEK293 cells (Supplementary Fig. 6E). Global proteome analysis of HAP1 cells treated with dNUDT5 for 6 h and 24 h revealed that NUDT5 was the only significantly downregulated protein, demonstrating the degrader’s high selectivity (Fig. 3F). We confirmed that ZFP91 levels remained unchanged in both HAP1 and HEK293 cells when treated with dNUDT5 at 100 nM for 24 h by Western blotting with a specific ZFP91 antibody (Supplementary Fig. 8). To understand the improved activity of dNUDT5 in comparison to previous compounds, we solved a co-crystal structure of dNUDT5 in complex with NUDT5, which indicated that dNUDT5 assumes a binding pose analogous to that of TH5427 (Fig. 3G). To further assess the kinetics and affinity of ternary complex formation by dNUDT5 in vitro, we performed heliX Y-structure proximity binding assays^22^ using recombinantly purified NUDT5 and CRBN^midi^^23^. We observed that dNUDT5 induces the formation of a high-affinity ternary complex between NUDT5 and CRBN^midi^ with an apparent dissociation constant (K_D_) of 0.34 ± 0.18 nM, as well as a slow off rate (k_off_) of 7.30 ± 0.33 •10^−4 ^s^−1^ (Fig. 3H). Both low K_D_ and low k_off_ values, which reflect a high-affinity, stable, and long-lived ternary complex in vitro, are generally associated with potent cellular degradation^24,25^, a consistent trend for dNUDT5 as indicated by the sub-nanomolar cellular degradation. Together, these data establish dNUDT5/dNUDT5nc as a probe set suitable for mechanistic interpretation in phenotypic assays.

### NUDT5 degradation rescues 6-thioguanine-induced toxicity

**Fig. 4:**
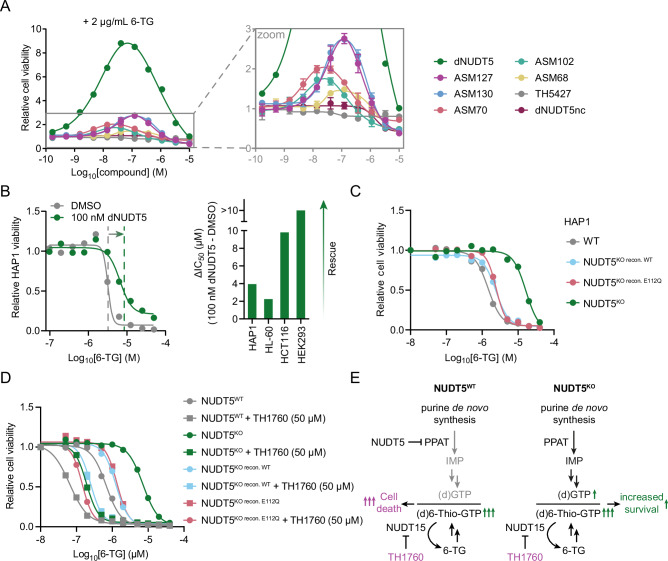
Depletion of NUDT5 protein by degraders or CRISPR KO rescues 6-thioguanine-mediated toxicity and acts antagonistically to NUDT15 inhibition. **A** Titration of indicated NUDT5 degraders and controls against a fixed concentration of 6-thioguanine (6-TG, 2 µg/mL) in HAP1 cells for 72 h. Data are shown as mean values derived from three technical replicates. Graph is representative of two independent biological replicates (*n* = 2). **B** dNUDT5 protects cells against 6-TG-induced toxicity across different cell lines (ΔIC_50_, half maximal inhibitory concentration difference). Data are shown as mean values derived from three technical replicates. Graph is representative of two independent biological replicates (*n* = 2). **C** Reconstitution of HAP1 NUDT5 knock-out (KO) cells with either NUDT5 wild-type (WT) or the catalytic-deficient E112Q mutant does not prevent 6-TG-mediated cell death. Data are normalized to DMSO. Data are shown as mean values derived from three technical replicates. Graph is representative of two independent biological replicates (*n *= 2). **D** Effect of NUDT15 inhibition by TH1760 on 6-TG sensitivity across different cellular backgrounds. Data are shown as mean values derived from three technical replicates. Graph is representative of two independent biological replicates (*n* = 2).** E** Proposed mechanism of NUDT5 and NUDT15 in 6-TG-mediated cell death.

With these potent and selective degraders in hand, we wondered whether our NUDT5 PROTACs would be able to rescue 6-TG-induced toxicity, analogous to CRISPR KO cells. We exposed HAP1 cells to escalating doses of our most potent compounds, including dNUDT5, ASM127, and ASM130, in the presence of 2 µg/mL 6-TG for 72 h (Fig. 4A). The degraders exhibited a dose-dependent rescue of HAP1 cell viability, with maximal effects observed between concentrations of 50-100 nM, in accordance with their degradation efficiency (Supplementary Figs. 2, 4, 5). Consistent with our previous results, dNUDT5 was the most efficient compound in abrogating 6-TG-induced cell death, whereas the inactive dNUDT5nc as well as the NUDT5 enzymatic inhibitor TH5427 failed to rescue cell viability. Notably, cell sensitivity towards 6-TG increased again at higher PROTAC concentrations, almost perfectly aligning with the hook effect apparent in our Western blot experiments. Evaluation of an extended panel of cell lines confirmed that the rescue effect conferred by dNUDT5 is not cell-type specific since we observed similar results in HL-60, HCT116, and HEK293 cells (Fig. 4B). Importantly, treatment of HEK293 or HAP1 cells indicated that the degraders themselves did not affect cell viability per se, even when applied at high µM concentrations (Supplementary Fig. 9). To investigate the metabolic consequences of dNUDT5-mediated degradation, we conducted metabolomic experiments comparing cells treated with dNUDT5 with NUDT5 KO cells. Indeed, we observed an apparent increase in de novo derived IMP (in ^13^C-D formate, 1 mM ^15^N adenosine 100 µM conditions) after 24 h (Supplementary Fig. 10).

These data support the conclusion that 6-TG-mediated cell death depends on a non-catalytic function of NUDT5. To corroborate this hypothesis, we reconstituted HAP1 NUDT5 KO cells with either wild-type (WT) or a catalytic-deficient E112Q NUDT5 mutant and evaluated their sensitivity against 6-TG. Indeed, both the catalytic-deficient mutant and the reconstituted WT exhibited comparable behavior towards 6-TG treatment, similar to that observed in the parental cells (Fig. 4C). Collectively, these orthogonal perturbations support a protein-dependent (non-catalytic) contribution of NUDT5 to thiopurine response and highlight the utility of targeted protein degradation for resolving cases where enzymatic inhibition fails to phenocopy genetic loss-of-function.

### NUDT5 and NUDT15 act antagonistically with respect to 6-thioguanine-induced toxicity

Interestingly, another member of the NUDIX family, NUDT15, has also been demonstrated to be a crucial factor influencing sensitivity towards 6-TG^26^. NUDT15 catalyses the conversion of 6-thio(d)GTP into 6-thio(d)GMP thereby preventing the incorporation of 6-TG metabolites into RNA and DNA. Importantly, recurring NUDT15 genetic variants observed in patients, which reduce NUDT15 catalytic activity, dramatically increase sensitivity towards 6-TG, necessitating appropriate dose adjustments to prevent side effects such as leukopenia. Consequently, NUDT15 represents an established biomarker for thiopurine therapy. These observations have inspired the development of NUDT15 small molecule inhibitors, such as TH1760 (ref. ^27^) to sensitize cancer cells towards 6-TG. Intrigued by these reports and our findings, we sought to investigate the relationship and potential mechanistic interplay between NUDT5 and NUDT15 with regard to 6-TG-mediated toxicity. We first titrated increasing concentrations of the NUDT15 inhibitor TH1760 against a fixed concentration of 6-TG (1 µM), which approximately corresponds to its cell viability IC_50_ value in HAP1 WT cells. Consistent with the literature, we observed that inhibition of NUDT15 by TH1760 sensitized HAP1 WT cells towards 6-TG in a dose-dependent manner (Supplementary Fig. 11). Analogous to our previous results, NUDT5 KO attenuated 6-TG cytotoxicity, while reconstitution of NUDT5 KO cells with either NUDT5 WT or the catalytic-deficient variant E112Q restored sensitivity (Supplementary Fig. 11). Likewise, titration of 6-TG against a fixed concentration of TH1760 (50 µM) indicated cells treated with TH1760 were more sensitive toward 6-TG while this effect was partially rescued in NUDT5 KO cells (2.8-fold difference) (Fig. 4D). Importantly, reconstitution of NUDT5 KO cells with the inactive NUDT5 E112Q mutant increased 6-TG-mediated cell death, further supporting the notion that NUDT5 enzymatic activity is dispensable in this context. Taken together, these results suggest that NUDT5 and NUDT15 act antagonistically with respect to 6-TG-mediated toxicity.

## Discussion

Targeted protein degradation (TPD) represents an intriguing alternative to classic small molecule inhibitors due to its distinct event-driven pharmacology. Whilst PROTACs are advancing in clinical trials^28^, well-characterized degraders also bear natural potential as chemical biology tools for drug target validation^29^, complementing genetic LOF screens^30,31^. Here, we establish a validated NUDT5 degrader toolkit and apply it to resolve a mechanistic ambiguity in thiopurine response that is not tractable with catalytic inhibitors alone. By integrating genetic knockout, catalytic inhibition, targeted degradation, an inactive degrader control, and catalytic-inactive reconstitution, we show that NUDT5 protein abundance is sufficient to govern cellular sensitivity to 6-TG across multiple contexts. These findings highlight an increasingly important principle in chemical biology: for some enzymes, phenotypes attributed to catalytic activity may instead reflect protein-dependent functions that are selectively interrogated by targeted degradation^32,33^. While NUDT5 inhibitors have been tested extensively in various cancer models^1,3,5,6^, the exact mechanism by which NUDT5 KO confers resistance to 6-TG has not been elucidated^11,34^. Contrary to expectations, we find that inhibition of NUDT5 enzymatic activity fails to rescue cell death induced by 6-TG^35–38^. Importantly, reconstitution of NUDT5 KO cells with either WT or a catalytically deficient NUDT5 mutant resensitizes cells to 6-TG, confirming that NUDT5 enzymatic activity is not required to trigger cell death upon thiopurine treatment. Intriguingly, our results further establish NUDT5 as the second NUDIX family member influencing 6-TG pharmacology. Indeed, genetic mutations or catalytic inhibition of NUDT15 have been shown to enhance sensitivity towards 6-TG^39–44^. Notably, our data suggest NUDT5 and NUDT15 exert discrete and conceptually antagonistic effects toward 6-TG. While NUDT5 depletion results in 6-TG resistance, suppression of NUDT15 enzymatic activity sensitizes cells towards 6-TG treatment, leading to increased cell death. In conjunction with results from another study^45^, we hypothesize that cell sensitivity towards 6-TG is determined by the ratio between (d)GTP and 6-thio(d)GTP, regulated by NUDT5 and NUDT15, respectively (Fig. 4E). Systematic profiling of NUDT5 protein-protein interactions suggests an obligate interaction with PPAT, the enzyme that catalyses the first rate-limiting step in the purine biosynthesis pathway^46^. Under NUDT5 WT conditions, NUDT5 binds to PPAT and represses the formation of (d)GTP, allowing more 6-thio(d)GTP to be incorporated into RNA and DNA. In NUDT5 KO cells, increased purine de novo synthesis leads to elevated levels of (d)GTP, effectively outcompeting toxic 6-TG metabolites. To provide quantitative support for this working model, we performed metabolomics profiling under conditions of NUDT5 depletion/degradation and compared these profiles to genetic loss-of-function. The resulting data show a consistent trend in nucleotide metabolism in the direction predicted by the phenotype, supporting the interpretation that NUDT5 loss alters nucleotide availability in a manner that reduces susceptibility to thiopurine challenge. We note that direct measurement of thiopurine-derived nucleotide species (e.g., 6-thio(d)GTP pools) is analytically challenging in many cellular settings due to low abundance, chemical instability, and matrix effects that complicate confident quantification. A deeper mechanistic framework for non-enzymatic NUDT5 regulation of de novo purine synthesis has been described in our accompanying published study^45^. Here, we complement this framework by providing a broadly usable degrader probe set and by directly demonstrating inhibitor-degrader-genetic discordance in a clinically relevant drug-response setting.

In summary, we provide a rigorously validated chemical degradation platform for NUDT5, including an inactive control compound, proteome-wide selectivity assessment, and orthogonal structural/biophysical characterization. Applying this toolkit reveals a clear dissociation between catalytic inhibition and protein loss in thiopurine response, supporting a protein-dependent contribution of NUDT5 that is not captured by enzymatic inhibition alone. Given the widespread reliance on inhibitors to infer enzyme function, our findings underscore the value of pairing inhibitors with targeted degradation to refine mechanism-based interpretation in drug response. The NUDT5 degrader toolkit should enable broad interrogation of NUDT5 biology across contexts and support future studies linking nucleotide metabolism regulation to therapeutic sensitivity.

## Methods

### Cell culture

HEK293, MCF7, A549 were cultured in DMEM + 10% FBS, HAP1 and HL-60 were cultured in IMDM + 10% FBS. All cell lines were maintained at 37 °C and 5% CO_2_.

### Western blotting

Cells were harvested using TrypLE (Gibco, 12605010), washed 1x with PBS, and frozen at –80 °C. Pellets were lysed on ice using RIPA buffer (25 mM Tris (pH 7.5), 150 mM NaCl, 1% Igepal, 1% SDS, 0.1% SDC, 1x protease inhibitor (Roche, cOmplete mini, 11836170001), 1x phosphatase (Thermo Scientific, Halt, 78429), 0.1% benzonase (Merck, E1014-5KU)), incubated for 15 min and then centrifuged for 20 min at 4 °C at maximum speed in a table-top centrifuge. Protein concentration was determined by a DC assay (Bio-Rad, 5000112), adjusted, mixed with 1x Laemmli buffer (Bio-Rad, 1610747), and boiled for 5 min at 95 °C. Samples were resolved by SDS-PAGE (Bio-Rad, 3450125) and transferred to a nitrocellulose membrane (Cytiva, 10600014) via wet-transfer. Membranes were blocked with 6% milk in PBS-T for 1 h at RT, washed, and then incubated with primary antibody diluted in PBS-T supplemented with 0.1% NaN_3_ overnight at 4 °C. Membranes were incubated with fluorophore-conjugated secondary antibodies, Alexa Fluor 680 anti-rabbit IgG (Life Technologies, A21109), Alexa Fluor 750 anti-mouse IgG (Life Technologies, A21037) diluted 1:5000 in 2% milk PBS-T, washed and imaged with an Odyssey imager (Licor). Primary antibodies: 1:1000 NUDT5 (Abcam, ab129172), 1:200 β-Actin (Santa Cruz, sc-47778), 1:1000 ZFP91 (Bethyl Laboratories, A303-245A).

### NanoBRET TE and ternary complex assays

NanoBRET target engagement assays were performed by transfection of NL-NUDT5 and 2.5 nM CBH-004 as previously described^13^. NanoBRET ternary complex assays were performed following the manufacturer’s instructions (Promega). In brief, HEK293 cells were transfected with NL-NUDT5 and either HL-VHL or HL-CRBN in a ratio of 1:100 and incubated overnight. Cells were adjusted to 200,000 cells/mL in Optimem + 4% FBS, 100 nM HaloTag NanoBRET 618 Ligand spiked in, and cells were seeded out in a 384 white well plate containing compounds that were pre-dispensed with an ECHO Liquid Handler. After 4 h or 20 h of incubation, a mix of substrate in assay medium was added to each well. Donor and acceptor signals were measured in a PHERAstar FSX or FS plate reader and analyzed by GraphPad Prism (v.9 or v.10). The acceptor/donor ratio was calculated, the no ligand background signal was subtracted, and multiplied by 1000 to yield mBRET units. Data were then normalized to DMSO.

### Untargeted global proteomics

#### Sample preparation and acquisition

Proteomics samples were digested with trypsin using STrap columns following the manufacturer’s protocol (C02-micro, ProtiFi). For untargeted global proteomics HAP1 cells were treated with DMSO or 100 nM dNUDT5 for 6 h or 24 h. Then, the cell pellets were collected and lysed in 5% SDS containing 1 μL/mL benzonase (E1014, Millipore) and subsequently short sonication to shear genomic DNA (1×2 s pulse, 20% amplitude, microtip, Sonics Vibra Cell). 50 μg protein from the total cell lysate or 80% of the elution fraction of the chemical pulldown were first reduced by 20 mM dithiothreitol (M02712, Fluorochem), and alkylated by 40 mM iodoacetamide (I1149, Sigma). Phosphoric acid and S-Trap protein binding buffer were added to the sample lysates. Then, the SDS lysate/S-Trap buffer were loaded into a S-Trap column (C02-micro-80, ProtiFi). 1 μg of trypsin (V5111, Promega) was used to digest each sample at 37 °C for 20 h. The samples were then eluted and dried by vacuum centrifugation. Peptide pellets were resuspended in mass spectrometry-grade water with 2% acetonitrile (85188, Thermo Scientific) and 0.1% trifluoroacetic acid (85183, Thermo Scientific). The samples were injected into Orbitrap Fusion Lumos Tribrid Mass Spectrometer (Thermo Scientific). Sample acquisition was performed as previously described^47^.

#### Data analysis

Raw data were searched against the human database (UP000005640, downloaded 08/21) using DIA-NN (v.1.8.1)^48^ by enabling FASTA digest for library-free search and Deep learning-based spectra, RTs, and IMs prediction. Trypsin/P was selected for the in silico digestion of the provided FASTA file, allowing up to 2 missed cleavages. Fixed modifications included N-terminal methionine excision and cysteine carbamidomethylation. Variable modifications were methionine oxidation and N-terminal acetylation, with a maximum of one modification per precursor. Precursor FDR was kept at 1% and MBR enabled. The protein groups matrix was further analysed using Perseus (2.0.9.0)^49^ or R studio (4.3.2). Potential contaminants were filtered against the top 300 most common Homo sapiens contaminants reported in CRAPome^50^. Proteins were removed if detected less than 70% across all runs. After column-wise normalisation by median subtraction, missing values were imputed by random numbers from a normal distribution (1.8 standard deviation, downshift width 0.3). Volcano plots were generated by calculating fold change and -log10(p-value) from an unpaired two-sided Student’s t-test.

### Cell viability and 6-TG assays

Cells were counted and adjusted to a density of 25,000 cells/mL in culture medium. A volume of 40 µL of the cell suspension was dispensed into white polypropylene 384-well plates (781207, Greiner) pre-dispensed with test compounds using an Echo Liquid Handler. Plates were sealed with breathable film and incubated at 37 °C with 5% CO₂ for 72 hours. Cell viability was assessed using the CellTiter-Glo Luminescent Cell Viability Assay, following the manufacturer’s protocol (Promega). Luminescence was measured using a PHERAstar FS or FSX plate reader, and data were analyzed using GraphPad Prism software (versions 9 or 10). Results were normalized to the vehicle control (DMSO). To evaluate cell viability in the presence of 6-TG, the compound was spiked into the cell suspension at a concentration of 2 μg/mL or as otherwise specified, and the experiment was conducted as described above.

### TH1760/6-TG assay

HAP1 cell lines were cultured in IMDM full media in 96-well plates (1200 cells/100 µL/well) containing the indicated concentration series of TH1760/6-TG, and where specifically stated additionally with a corresponding concentration of 6-TG (1 µM) or TH1760 (50 µM), respectively. After 72 h incubation, cell viability was measured using the CellTiter-Glo assay (Promega). Signals were normalized to the control condition.

### Conjugation of protein to DNA for heliX proximity binding assay

NUDT5 and CRBN^midi^ were covalently coupled via their primary amines to ssDNA Ligand strand or Ligand strand 2, respectively (coupling kits HK-NHS-1 or HK-NHS-4, Dynamic Biosensors). The protein-DNA conjugate was purified using the proFIRE system (Dynamic Biosensors). Fractions containing the desired protein-DNA conjugates were buffer exchanged into buffer containing 10 mM HEPES pH7.5, 40 mM NaCl, 0.05% Tween20, concentrated to 500 nM and stored at −80 °C prior to use.

### heliX proximity binding assay

All measurements were performed using the heliX instrument (Dynamic Biosensors) at 25 °C in assay buffer 10 mM HEPES pH7.5, 140 mM NaCl, 0.05% Tween, 2% DMSO, 2 mM MgCl_2_. dNUDT5 was injected at concentrations of 0.16, 0.8, 4, 20, and 100 nM (2 replicates) or 0.37, 1.11, 3.33, and 10 nM (1 replicate) with the chip surface freshly functionalized before each concentration measurement. The Y-structure was assembled using the Y-structure kit 1 (HK-YS-1, Dynamic Biosensors) to a final concentration of 50 nM with NUDT5 conjugated to Ligand strand and CRBN^midi^ conjugated to Ligand strand 2. Measurements were performed in fluorescence resonance energy transfer (FRET) mode, with the green LED power set to auto and the red signal detected.

Data analysis was performed with heliOS software (Dynamic Biosensors). The highest concentration was excluded for analysis when hook effect was apparent. The association and dissociation rates (k_on_ and k_off)_, and dissociation constants (K_D_) were calculated using a global single exponential fit model after blank referencing correction. The values reported in Fig. 3H are the mean ± standard error of the mean (SEM) from three independent experiments.

### Metabolomics

Experiments were performed in duplicates. Approximately 1.5 × 10^6^ HAP1 wt and NUDT5 KO cells were seeded in IMDM (full) prior to treatment with isotopically labeled metabolites. Furthermore, a separate set of HAP1 wt cells were treated with 100 nM dNUDT5. After 24 h, plates were washed with PBS and replaced with IMDM containing dialysed FBS (Gibco). Cells were treated with 1 mM of isotopically labeled sodium formate (Eurisotop, CDLM-6203-0.25) or with 1 mM of isotopically labeled sodium and 100 µM isotopically labeled adenosine (Silantes, 125303601). After 24 h, 5 × 10^6^ cells for each replicate was harvested, washed with PBS, immediately snap frozen and stored at −80 °C. Cell pellets were lysed with 150 µL ice-cold 80:20 MeOH:H_2_O, vortexed and sonicated for 5 minutes on ice. The extract was centrifuged for 10 minutes at 10,000 × *g* at 4 °C and the supernatant was collected in a glass insert vial. This procedure was repeated and the two supernatants were combined. Solvent was evaporated in a vacuum concentrator. Metabolites were reconstituted in 50 µL pure MeOH. A Vanquish UHPLC system (Thermo Scientific) coupled with an Orbitrap Q Exactive (Thermo Scientific) mass spectrometer was used for the LC-MS analysis. Metabolite separation was performed by reversed-phase chromatography employing an Acquity UPLC BEH Amide 1.7 µm, 2.1 mm×100 mm (Waters) analytical column at a column temperature of 40 °C. As mobile phase A, MS-grade water containing 0.15% formic acid and 10 mM NH_4_HCO_3_ was used. Mobile phase B consisted of MS-grade MeOH containing 0.15% formic acid and 10 mM NH_4_HCO_3_. The flow rate was set to 400 µL/min, and a stepped gradient of mobile phase B was applied to ensure optimal separation of the analyzed metabolites: 100% (min 0), 100% (min 6), 94.1% (min 6.1), 82.4% (min 10), 70.6% (min 12), 100% (min 3), followed by a washing and equilibration phase. The mass spectrometer was operated in ESI-positive mode. Seven full MS-SIM scans were performed consecutively, all at 70,000 resolution, an AGC target of 1e6, and 200 ms maximum injection time. The mass range differed between the seven scans: 1) 140 – 160 m/z, 2) 225 – 240 m/z, 3) 255 – 265 m/z, 4) 265 – 280 m/z, 5) 305 – 320 m/z, 6) 321 – 359 m/z, and 7) 360 – 380 m/z. Data analysis was performed using Skyline software (version: 25.1.0.237)^51^. Quantification of target metabolites was performed by manually integrating the area under the curve, and data were plotted using GraphPad Prism v.10.

### Protein expression and purification

The gene encoding NUDT5 (ENSG00000165609) (residues 1-208) was sub-cloned into a pNIC28-Bsa4 vector using ligation-independent cloning (LIC), resulting in an N-terminal 6x histidine tag followed by a TEV cleavage site. The plasmid was transformed into Escherichia coli BL21 (DE3), and protein expression was induced at an optical density (OD600) of 0.8 with 0.3 mM isopropyl-β-D-1-thiogalactopyranoside (IPTG) at 18 °C overnight. Cell pellets were resuspended in lysis buffer (20 mM HEPES, 500 mM NaCl, 5% glycerol, 0.5 mM TCEP, pH 7.5) supplemented with lysozyme, a protease inhibitor cocktail (Set III, EDTA-free, Calbiochem), and benzonase (2 µL of 10,000 U/µL, Merck). Cells were lysed using an ultrasonic cell disruptor (Vibra-Cell, Sonics).

The clarified supernatant was applied to a Ni-NTA affinity chromatography column (HisTrap Crude FF, GE Healthcare) and eluted with an increasing gradient of imidazole concentrations. The affinity-purified protein was incubated with TEV protease overnight at 4 °C to cleave the histidine tag. Following cleavage, reverse nickel affinity chromatography was performed to separate the cleaved tag from the protein. The protein was further purified using size-exclusion chromatography (16/600 Superdex 75 PG, GE Healthcare) in a final buffer consisting of 20 mM HEPES, pH 7.5, 500 mM NaCl, and 0.5 mM TCEP. The purity of the protein was confirmed by SDS-PAGE, and the protein was concentrated to 26 mg/mL using 10,000 MWCO Vivaspin concentrators (Vivascience). CRBN^midi^ (Addgene Plasmid #215330) was expressed and purified according to a previously published procedure^23^.

### Crystallization

Prior to crystallization trials, NUDT5 (26 mg/mL) was mixed with dNUDT5 at a 1:2 molar ratio in the presence of 2 mM MgCl2 and incubated on ice for 1 h. Crystallization screens were set up using the sitting-drop vapor diffusion method, with equal volumes of protein and precipitant solutions (1:1 ratio). Co-crystals were grown in a buffer solution comprising 0.1 M Tris-HCl (pH 8.5), 30% PEG 4000, and 0.2 M sodium acetate. Crystals were cryo-protected with the crystallization condition containing 20% glycerol and flash-frozen in liquid nitrogen.

### Data Collection, Structure Determination, and Refinement

X-ray datasets were collected at Diamond Light Source (Harwell, U.K.) on I03 beamline using an Eiger2 XE 16 M detector. Data processing and scaling was performed using the program DIALS in combination with XIA2^52^. The structure was solved in molecular replacement in PHASER^53^ using PDB 5NWH as the search model. The model was further built in Coot^54^ and refined in PHENIX^55^. Data collection and refinement statistics are summarized in Table 1. Figure was prepared using ChimeraX^56^. Structural data generated in this study have been deposited in the Protein Data Bank (PDB) under accession code 9G6Y.

### Compounds

Synthetic procedures for all compounds are provided in the Supplementary Information.

### Reporting summary

Further information on research design is available in the Nature Portfolio Reporting Summary linked to this article.

## Supplementary information

Supplementary Information

Reporting Summary

Transparent Peer Review file

## Source data

Source Data

## Data Availability

The mass spectrometry proteomics data have been deposited to the ProteomeXchange Consortium via the PRIDE partner repository with the dataset identifier PXD068003 (Total proteome analysis of NUDT5 PROTACs). The metabolomics raw data generated in this study have been deposited to the MassIVE repository with the dataset identifier MSV000101725 (Metabolomics analysis of dNUDT5 treated cells). The crystallographic coordinates and structure factor data generated in this study have been deposited in the Protein Data Bank with the accession code 9G6Y. Additional crystal structures used in this study are 8RDZ and 8RIY. All data presented in this study are included in the article and its Supplementary Information. Source data are provided with this paper.

## References

[1] Page, B. D. G. et al. Targeted NUDT5 inhibitors block hormone signaling in breast cancer cells (vol 9, 250, 2018). *Nat. Commun.* **10**, 10.1038/s41467-019-12806-1 (2019). 10.1038/s41467-017-02293-7 PMC5772648 29343827

[2] Wright, R. H. G. et al. ADP-ribose-derived nuclear ATP synthesis by NUDIX5 is required for chromatin remodeling. *Science* **352**, 1221–1225 (2016). 27257257 10.1126/science.aad9335

[3] Pickup, K. E. et al. Expression of Oncogenic Drivers in 3D Cell Culture Depends on Nuclear ATP Synthesis by NUDT5. *Cancers* **11**, 10.3390/cancers11091337 (2019). 10.3390/cancers11091337 PMC6770457 31510016

[4] Zhang, H. et al. The high expression of NUDT5 indicates poor prognosis of breast cancer by modulating AKT / Cyclin D signaling. *Plos One* **16**, 10.1371/journal.pone.0245876 (2021). 10.1371/journal.pone.0245876 PMC7877577 33571243

[5] Qian, J. et al. The novel phosphatase NUDT5 is a critical regulator of triple-negative breast cancer growth. *Breast Cancer Res.* **26**, 23 (2024). 38317231 10.1186/s13058-024-01778-w PMC10845800

[6] Qi, H., Grace Wright, R. H., Beato, M. & Price, B. D. The ADP-ribose hydrolase NUDT5 is important for DNA repair. *Cell Rep.* **41**, 111866 (2022). 36543120 10.1016/j.celrep.2022.111866

[7] Li, Y. N. et al. Mitochondrial related genome-wide Mendelian randomization identifies putatively causal genes for multiple cancer types. *Ebiomedicine* **88**, 10.1016/j.ebiom.2022.104432 (2023). 10.1016/j.ebiom.2022.104432 PMC9841346 36634566

[8] Ding, H. et al. NUDT5-Determines the fate of head and neck squamous cell carcinoma cells under endoplasmic reticulum stress by catalyzing nuclear ATP production to promote DNA repair. *Oral. Oncol.* **141**, 106397 (2023). 37156197 10.1016/j.oraloncology.2023.106397

[9] Sun, R. et al. Proteomic landscape profiling of primary prostate cancer reveals a 16-protein panel for prognosis prediction. *Cell Rep. Med* **5**, 101679 (2024). 39168102 10.1016/j.xcrm.2024.101679 PMC11384950

[10] Doench, J. G. et al. Optimized sgRNA design to maximize activity and minimize off-target effects of CRISPR-Cas9. *Nat. Biotechnol.* **34**, 184–191 (2016). 26780180 10.1038/nbt.3437 PMC4744125

[11] Phoomak, C. et al. Signal recognition particle receptor-beta (SR-beta) coordinates cotranslational N-glycosylation. *Sci. Adv.* **9**, eade8079 (2023). 36921042 10.1126/sciadv.ade8079 PMC10017033

[12] Bayoumy, A. B. et al. The continuous rediscovery and the benefit-risk ratio of thioguanine, a comprehensive review. *Expert Opin. Drug Metab. Toxicol.* **16**, 111–123 (2020). 32090622 10.1080/17425255.2020.1719996

[13] Balikçi, E. et al. Unexpected noncovalent off-target activity of clinical btk inhibitors leads to discovery of a dual NUDT5/14 Antagonist. *J. Med Chem.* **67**, 7245–7259 (2024). 38635563 10.1021/acs.jmedchem.4c00072 PMC11089510

[14] Buhimschi, A. D. et al. Targeting the C481S Ibrutinib-Resistance Mutation in Bruton’s Tyrosine Kinase Using PROTAC-Mediated Degradation. *Biochemistry* **57**, 3564–3575 (2018). 29851337 10.1021/acs.biochem.8b00391

[15] Gabizon, R. et al. Efficient targeted degradation via reversible and irreversible covalent PROTACs. *J. Am. Chem. Soc.* **142**, 11734–11742 (2020). 32369353 10.1021/jacs.9b13907 PMC7349657

[16] Nguyen, T. M. et al. Proteolysis-targeting chimeras with reduced off-targets. *Nat. Chem.* **16**, 218–228 (2024). 38110475 10.1038/s41557-023-01379-8 PMC10913580

[17] Zaidman, D., Prilusky, J. & London, N. PRosettaC: Rosetta Based Modeling of PROTAC Mediated Ternary Complexes. *J. Chem. Inf. Model* **60**, 4894–4903 (2020). 32976709 10.1021/acs.jcim.0c00589 PMC7592117

[18] Troup, R. I., Fallan, C. & Baud, M. G. J. Current strategies for the design of PROTAC linkers: a critical review. *Explor Target Antitumor Ther.* **1**, 273–312 (2020). 36046485 10.37349/etat.2020.00018 PMC9400730

[19] Zografou-Barredo, N. A., Hallatt, A. J., Goujon-Ricci, J. & Cano, C. A beginner’s guide to current synthetic linker strategies towards VHL-recruiting PROTACs. *Bioorg. Med Chem.* **88-89**, 117334 (2023). 37224698 10.1016/j.bmc.2023.117334

[20] Li, K. & Crews, C. M. PROTACs: past, present and future. *Chem. Soc. Rev.* **51**, 5214–5236 (2022). 35671157 10.1039/d2cs00193d PMC10237031

[21] Casement, R., Bond, A., Craigon, C. & Ciulli, A. Mechanistic and Structural Features of PROTAC Ternary Complexes. *Methods Mol. Biol.* **2365**, 79–113 (2021). 34432240 10.1007/978-1-0716-1665-9_5

[22] Ponzo, I. et al. Proximity binding assay for PROTAC ternary complex analysis. *ACS Sensors* **11**, 3876–3888 (2026). 10.1021/acssensors.5c04944 42011828

[23] Kroupova, A. et al. Design of a Cereblon construct for crystallographic and biophysical studies of protein degraders. *Nat. Commun.* **15**, 8885 (2024). 39406745 10.1038/s41467-024-52871-9 PMC11480361

[24] Roy, M. J. et al. SPR-measured dissociation kinetics of PROTAC ternary complexes influence target degradation rate. *ACS Chem. Biol.* **14**, 361–368 (2019). 30721025 10.1021/acschembio.9b00092 PMC6423499

[25] Wurz, R. P. et al. Affinity and cooperativity modulate ternary complex formation to drive targeted protein degradation. *Nat. Commun.* **14**, 4177 (2023). 37443112 10.1038/s41467-023-39904-5 PMC10344917

[26] Moyer, A. M. NUDT15: A bench to bedside success story. *Clin. Biochem* **92**, 1–8 (2021). 33675810 10.1016/j.clinbiochem.2021.02.007

[27] Zhang, S. M. et al. Development of a chemical probe against NUDT15. *Nat. Chem. Biol.* **16**, 1120–1128 (2020). 32690945 10.1038/s41589-020-0592-z PMC7610571

[28] Chirnomas, D., Hornberger, K. R. & Crews, C. M. Protein degraders enter the clinic - a new approach to cancer therapy. *Nat. Rev. Clin. Oncol.* **20**, 265–278 (2023). 36781982 10.1038/s41571-023-00736-3 PMC11698446

[29] Nemec, V., Schwalm, M. P., Muller, S. & Knapp, S. PROTAC degraders as chemical probes for studying target biology and target validation. *Chem. Soc. Rev.* **51**, 7971–7993 (2022). 36004812 10.1039/d2cs00478j

[30] Moffat, J., Komor, A. C. & Lum, L. Impact of CRISPR in cancer drug discovery. *Science* **386**, 378–379 (2024). 39446947 10.1126/science.adi6884

[31] Behan, F. M. et al. Prioritization of cancer therapeutic targets using CRISPR–Cas9 screens. *Nature* **568**, 511–516 (2019). 30971826 10.1038/s41586-019-1103-9

[32] Weiss, W. A., Taylor, S. S. & Shokat, K. M. Recognizing and exploiting differences between RNAi and small-molecule inhibitors. *Nat. Chem. Biol.* **3**, 739–744 (2007). 18007642 10.1038/nchembio1207-739 PMC2924165

[33] Shi, J. et al. Discovery of cancer drug targets by CRISPR-Cas9 screening of protein domains. *Nat. Biotechnol.* **33**, 661–667 (2015). 25961408 10.1038/nbt.3235 PMC4529991

[34] Ruiz, S. et al. A Genome-wide CRISPR Screen Identifies CDC25A as a Determinant of Sensitivity to ATR Inhibitors. *Mol. Cell* **62**, 307–313 (2016). 27067599 10.1016/j.molcel.2016.03.006 PMC5029544

[35] Vangamudi, B. et al. The SMARCA2/4 ATPase domain surpasses the bromodomain as a drug target in SWI/SNF-mutant cancers: insights from cDNA Rescue and PFI-3 Inhibitor Studies. *Cancer Res.* **75**, 3865–3878 (2015). 26139243 10.1158/0008-5472.CAN-14-3798 PMC4755107

[36] Farnaby, W. et al. BAF complex vulnerabilities in cancer demonstrated via structure-based PROTAC design. *Nat. Chem. Biol.* **15**, 672–680 (2019). 31178587 10.1038/s41589-019-0294-6 PMC6600871

[37] Wang, S. et al. Uncoupling of PARP1 trapping and inhibition using selective PARP1 degradation. *Nat. Chem. Biol.* **15**, 1223–1231 (2019). 31659317 10.1038/s41589-019-0379-2 PMC6864272

[38] Adhikari, B. et al. PROTAC-mediated degradation reveals a non-catalytic function of AURORA-A kinase. *Nat. Chem. Biol.* **16**, 1179–1188 (2020). 32989298 10.1038/s41589-020-00652-y PMC7610535

[39] Kakuta, Y. et al. NUDT15 R139C causes thiopurine-induced early severe hair loss and leukopenia in Japanese patients with IBD. * Pharmacogenomics J.* **16**, 280–285 (2016). 26076924 10.1038/tpj.2015.43

[40] Kanhatai, C. et al. NUDT15 c.415C>T increases risk of 6-mercaptopurine induced myelosuppression during maintenance therapy in children with acute lymphoblastic leukemia. *Haematologica* **101**, e24–e26 (2015). 26405151 10.3324/haematol.2015.134775 PMC4697903

[41] Moriyama, T. et al. NUDT15 polymorphisms alter thiopurine metabolism and hematopoietic toxicity. *Nat. Genet.* **48**, 367–373 (2016). 26878724 10.1038/ng.3508 PMC5029084

[42] Tanaka, Y. et al. Susceptibility to 6-MP toxicity conferred by a NUDT15 variant in Japanese children with acute lymphoblastic leukaemia. *Br. J. Haematol.* **171**, 109–115 (2015). 26033531 10.1111/bjh.13518

[43] Yang, J. J. et al. Inherited NUDT15 variant is a genetic determinant of mercaptopurine intolerance in children with acute lymphoblastic leukemia. *J. Clin. Oncol.* **33**, 1235–1242 (2015). 25624441 10.1200/JCO.2014.59.4671 PMC4375304

[44] Yang, S.-K. et al. A common missense variant in NUDT15 confers susceptibility to thiopurine-induced leukopenia. *Nat. Genet.* **46**, 1017–1020 (2014). 25108385 10.1038/ng.3060 PMC4999337

[45] Nguyen, T.-A. et al. A non-enzymatic role of Nudix hydrolase 5 in repressing purine de novo synthesis. *Science* **390**, 1143–1150 (2025). 41196952 10.1126/science.adv4257 PMC7618541

[46] Yamaoka, T. et al. Feedback inhibition of amidophosphoribosyltransferase regulates the rate of cell growth via purine nucleotide, DNA, and protein syntheses*. *J. Biol. Chem.* **276**, 21285–21291 (2001). 11290738 10.1074/jbc.M011103200

[47] Chen, X. M. et al. Paralogue-selective degradation of the lysine acetyltransferase EP300. *Jacs Au* **4**, 3094–3103 (2024). 39211607 10.1021/jacsau.4c00442 PMC11350577

[48] Demichev, V., Messner, C. B., Vernardis, S. I., Lilley, K. S. & Ralser, M. DIA-NN: neural networks and interference correction enable deep proteome coverage in high throughput. *Nat. Methods* **17**, 41–4 (2020). 31768060 10.1038/s41592-019-0638-x PMC6949130

[49] Tyanova, S. et al. The Perseus computational platform for comprehensive analysis of (prote)omics data. *Nat. Methods* **13**, 731–740 (2016). 27348712 10.1038/nmeth.3901

[50] Mellacheruvu, D. et al. The CRAPome: a contaminant repository for affinity purification-mass spectrometry data. *Nat. Methods* **10**, 730–736 (2013). 23921808 10.1038/nmeth.2557 PMC3773500

[51] Adams, K. J. et al. Skyline for small molecules: a unifying software package for quantitative metabolomics. *J. proteome Res.* **19**, 1447–1458 (2020). 31984744 10.1021/acs.jproteome.9b00640 PMC7127945

[52] Gildea, R. J. et al. New methods for indexing multi-lattice diffraction data. *Acta Crystallogr D.* **70**, 2652–2666 (2014). 25286849 10.1107/S1399004714017039 PMC4188007

[53] Mccoy, A. J. et al. Crystallographic software. *J. Appl Crystallogr* **40**, 658–674 (2007). 19461840 10.1107/S0021889807021206 PMC2483472

[54] Casañal, A., Lohkamp, B. & Emsley, P. Current developments in Coot for macromolecular model building of Electron Cryo-microscopy and Crystallographic Data. *Protein Sci.* **29**, 1069–1078 (2020). 31730249 10.1002/pro.3791 PMC7096722

[55] Afonine, P. V. et al. Real-space refinement in PHENIX for cryo-EM and crystallography. *Acta Crystallogr D.* **74**, 531–544 (2018). 10.1107/S2059798318006551 PMC6096492 29872004

[56] Goddard, T. D. et al. UCSF ChimeraX: Meeting modern challenges in visualization and analysis. *Protein Sci.* **27**, 14–25 (2018). 28710774 10.1002/pro.3235 PMC5734306

