# Supplementary material for: Targeted Protein Degradation of NUDT5 Dissociates Catalytic Inhibition from Protein Loss in 6-Thioguanine Response
Source: Nat Commun. 2026 Jun 30;17:8192. doi: 10.1038/s41467-026-74489-9 (PMC13462928; doi:10.1038/s41467-026-74489-9)
Supplement: Supplementary file 1 [file 41467_2026_74489_MOESM1_ESM.pdf]

Supplementary Figures

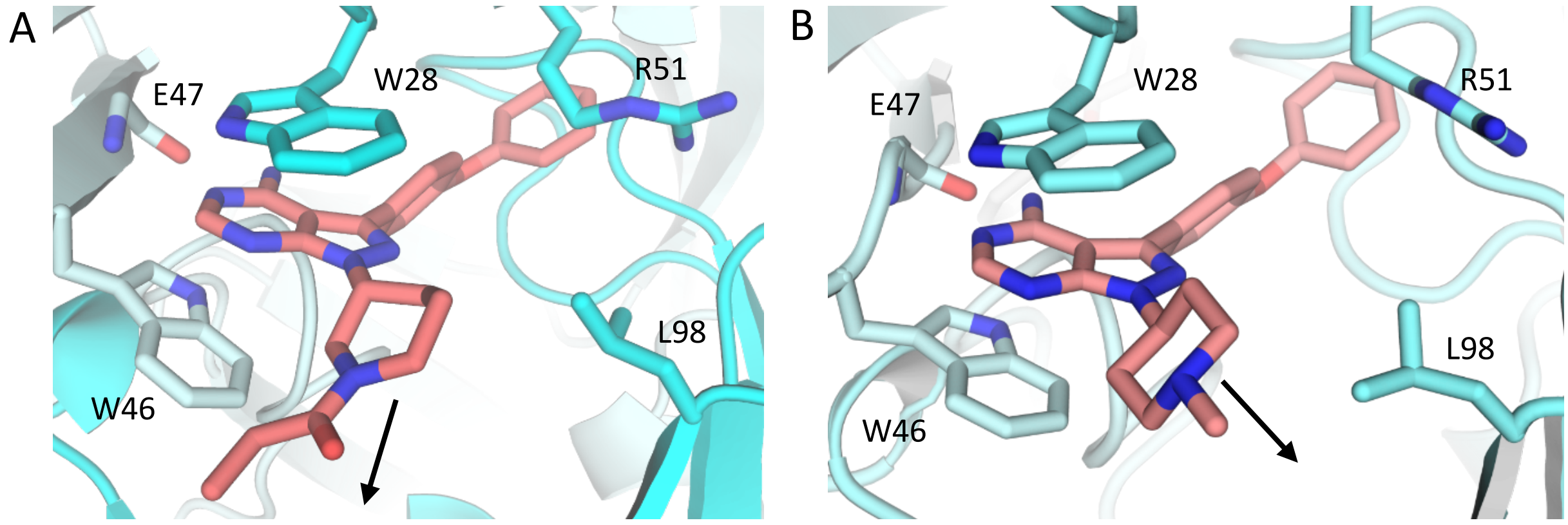

Supplementary Figure 1. Design strategy for the synthesis of ibrutinib-derived PROTACs

A) Co-crystal structure of NUDT5 bound to ibrutinib (PDB: 8RDZ). The arrow depicts the exit vector. B) Co-crystal structure of NUDT5 bound to the ibrutinib analogue (compound 9) (PDB: 8RIY). The arrow depicts the exit vector.

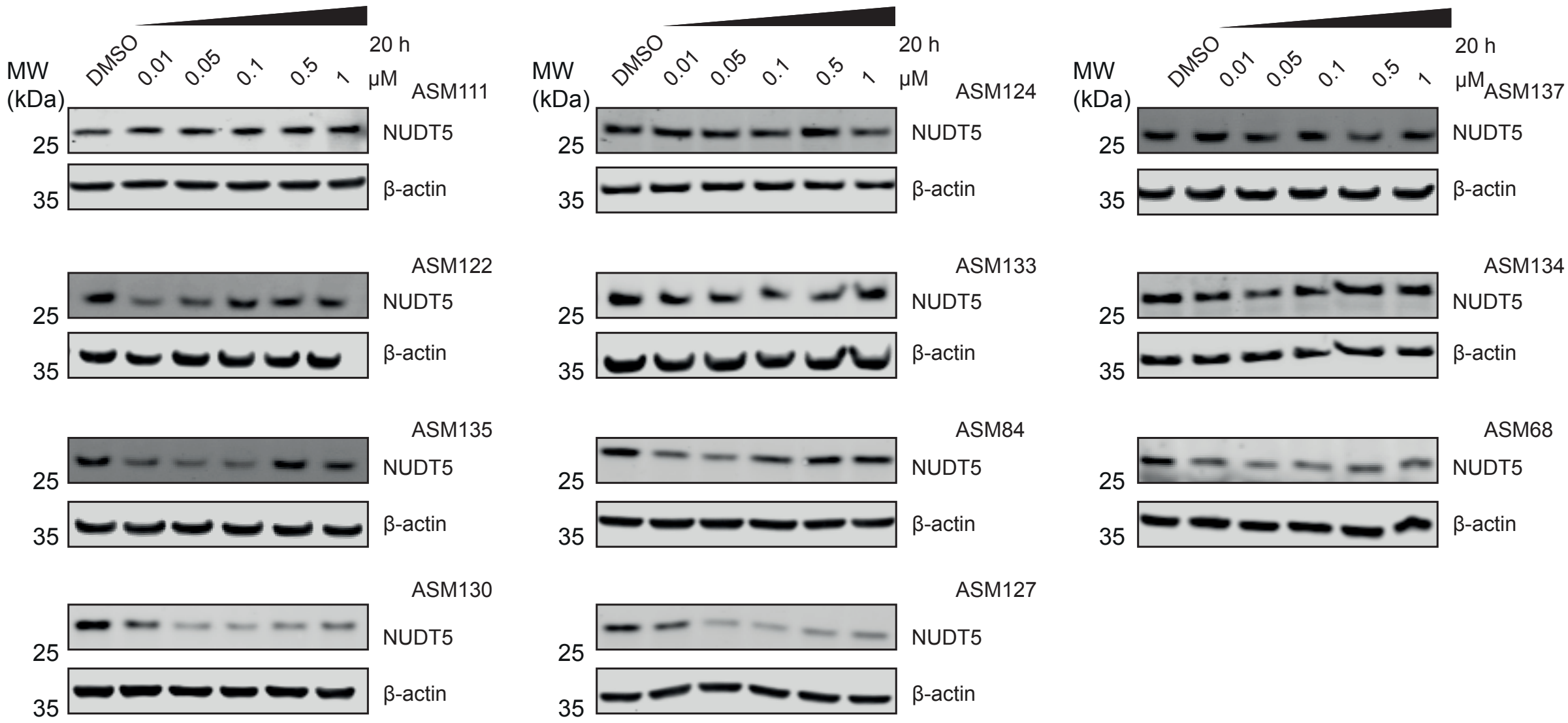

Supplementary Figure 2. Western blot analysis of NUDT5 degradation by flexible linker-based PROTACs in HEK293 cells. Western blots are representative of two independent biological replicates (n = 2).

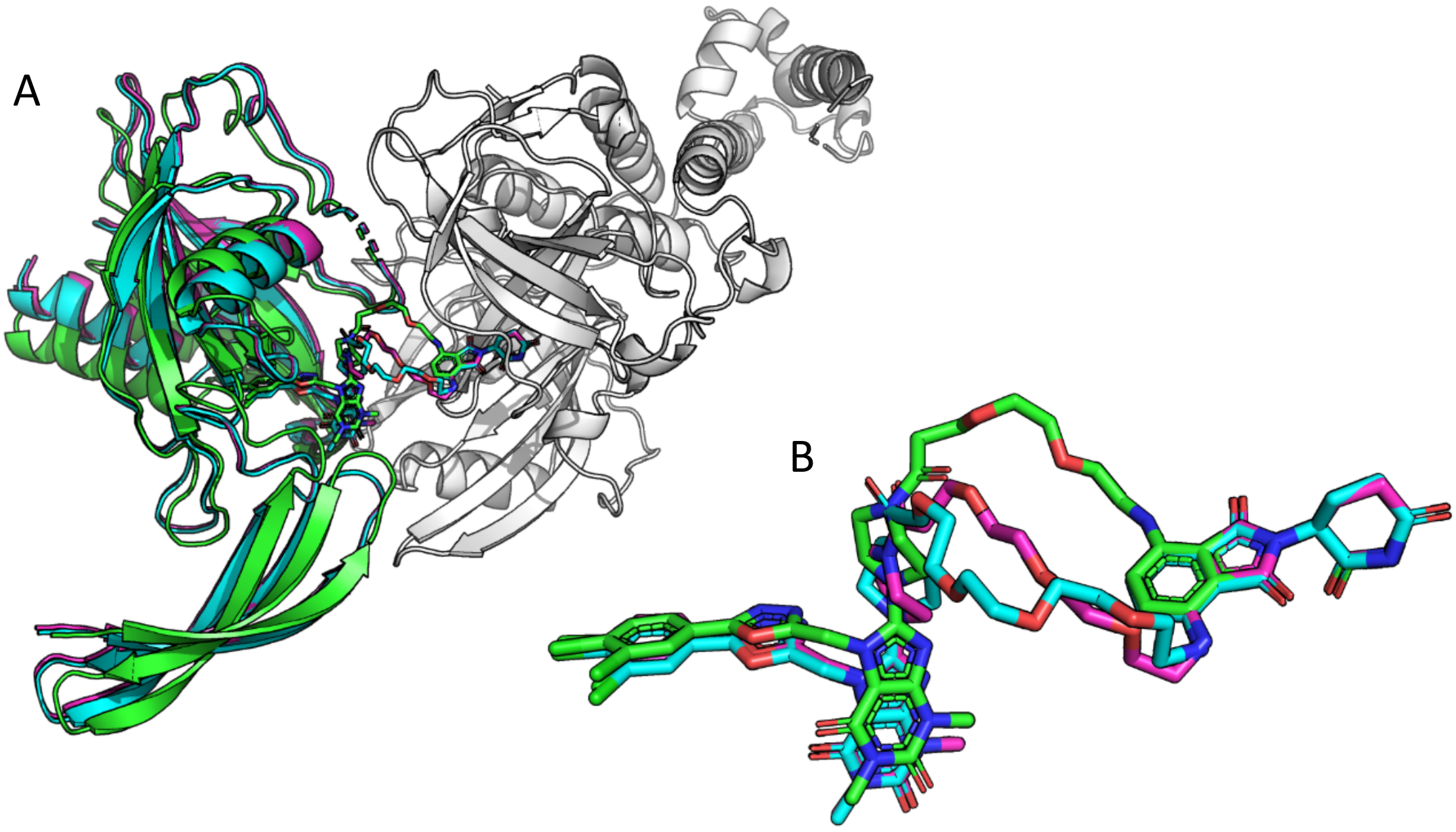

Supplementary Figure 3. PRosettaC ternary complex prediction for active PROTACs. A) Reclustering of the top-scoring models for ASM68 (green), ASM127 (magenta) and ASM130 (cyan) with an RMSD threshold of 2 Å, CRBN is show in grey. The top cluster includes 53 models (14 of ASM68, 24 of ASM127 and 15 of ASM130). The next clusters contain less than 20 structures and are typically limited to models arising from only one PROTAC, further increasing the confidence in this ternary complex prediction. B) Zoom in on the PROTAC binding mode shows that all three PROTACs are able to bind similarly in this ternary complex. We should note that while ASM68 is predicted to have a flipped conformation of the phthalimide moiety, other members of the same cluster showed a similar conformation to those of ASM127 and ASM130, indicating both exits may be tolerated.

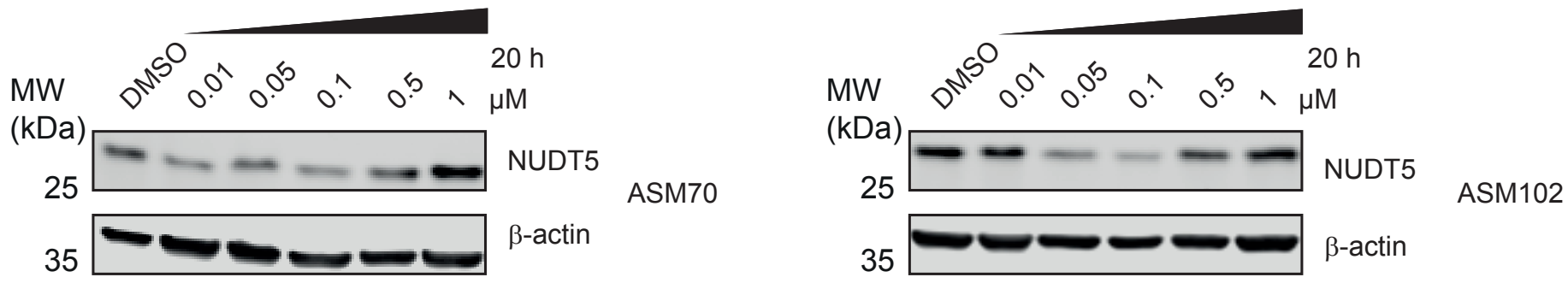

Supplementary Figure 4. Western blot analysis of NUDT5 degradation by rigid linker-based PROTACs in HEK293 cells. Western blots are representative of two independent biological replicates (n = 2).

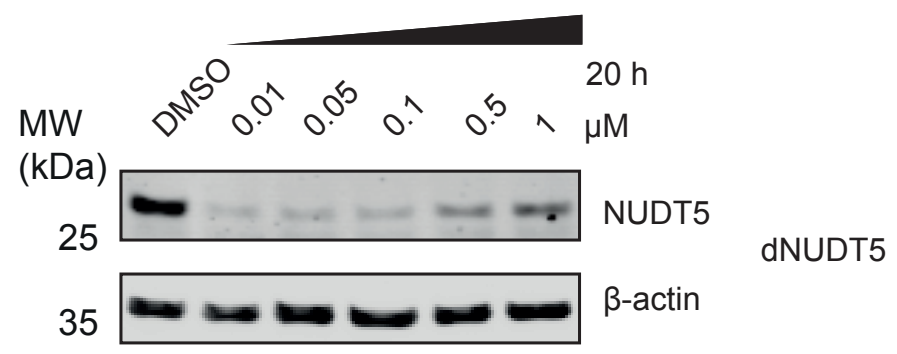

Supplementary Figure 5. Western blot analysis of NUDT5 degradation by dNUDT5 in HEK293 cells. Western blot is representative of two independent biological replicates (n = 2).

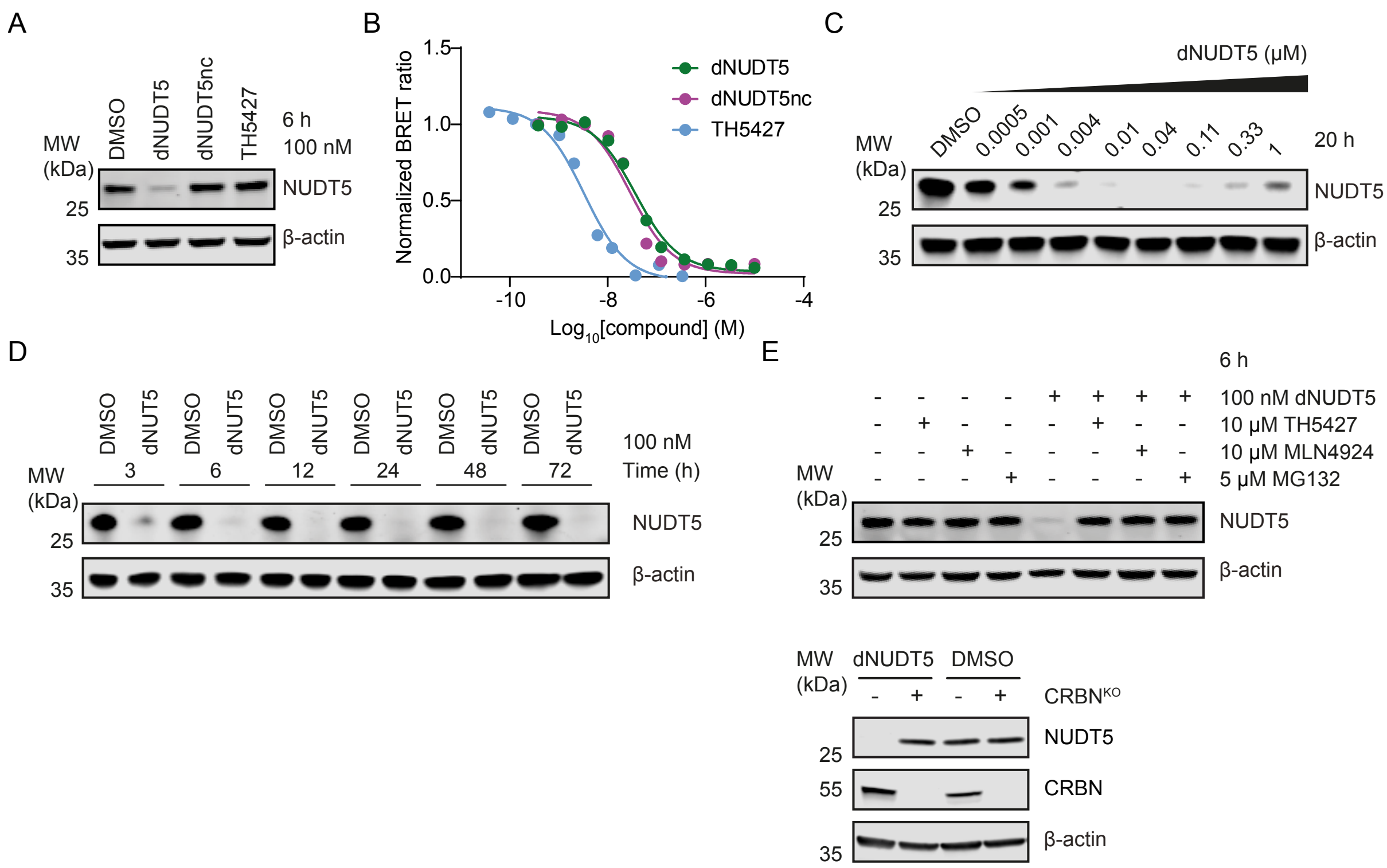

Supplementary Figure 6. Characterization of dNUDT5 in HEK293 cells.

A) Western blot analysis showing that dNUDT5, but not the CRBN-binding-deficient analogue dNUDT5nc or the NUDT5 catalytic inhibitor TH5427, induces degradation of NUDT5 in HEK293 cells. B) NUDT5 NanoBRET target engagement (TE) assay results for dNUDT5 (EC_{50} = 37 nM) and dNUDT5nc (EC_{50} = 28 nM) compared with the control TH5427 (EC_{50} = 3 nM) (BRET, bioluminescence resonance energy transfer; EC_{50}, half maximal effective concentration). Data are shown as mean values derived from three technical replicates. Graphs are representative of two independent biological replicates (n = 2). C) Dose-dependent degradation of NUDT5 following treatment with dNUDT5 in HEK293 cells. D) Time-dependent degradation of NUDT5 in HEK293 cells treated with 100 nM dNUDT5. E) Rescue of dNUDT5-induced NUDT5 degradation by co-treatment with TH5427 (10 µM), the neddylation inhibitor MLN4924 (10 µM), the proteasome inhibitor MG132 (5 µM) and in CRBN knock-out (KO) HEK293 cells. Western blots shown in this figure are representative of two independent biological replicates (n = 2).

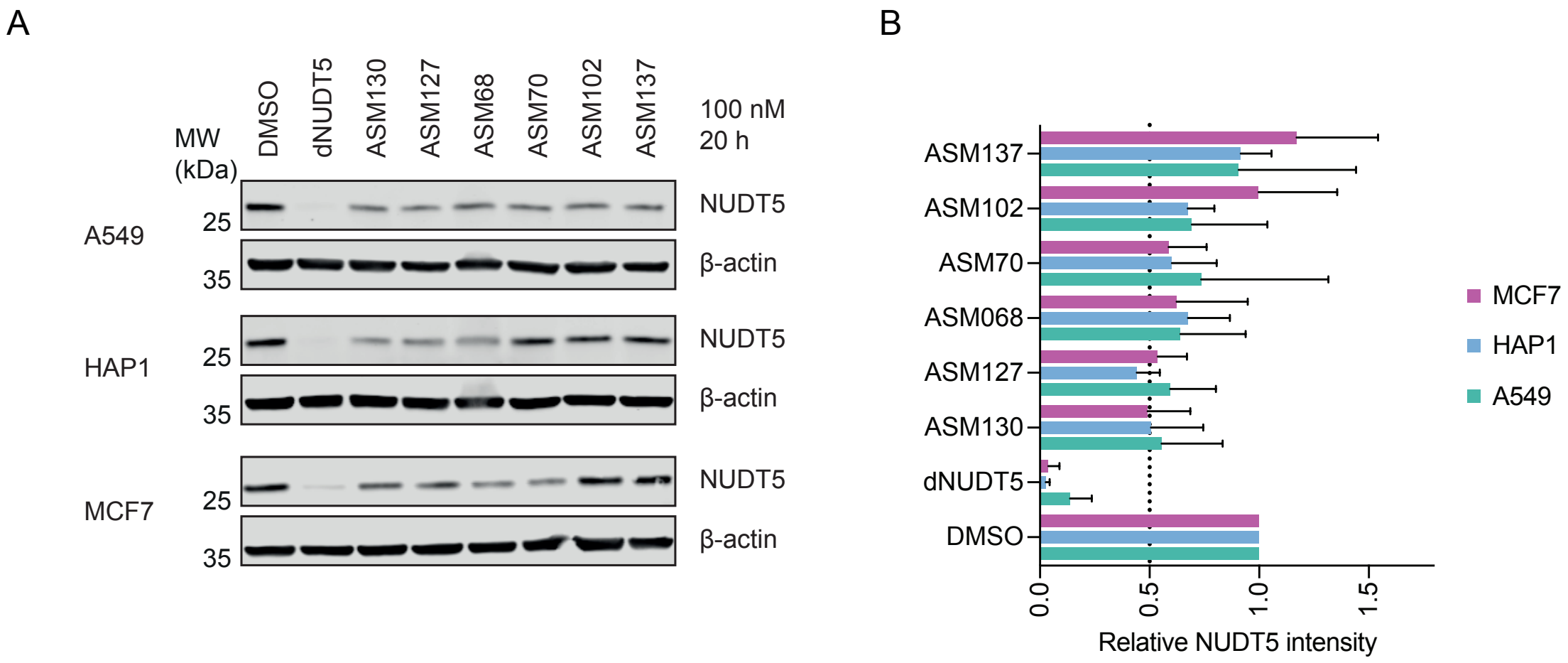

Supplementary Figure 7. NUDT5 degradation efficiency of dNUDT5, ASM130, ASM127, ASM68, ASM102 and ASM137 assessed by Western blotting in A549, HAP1, and MCF7 cells. A) Representative Western blot. B) Bar graph showing mean ± SD of relative NUDT5 band intensities following PROTAC treatment, normalised to DMSO control. Data represent four independent biological replicates (n = 4).

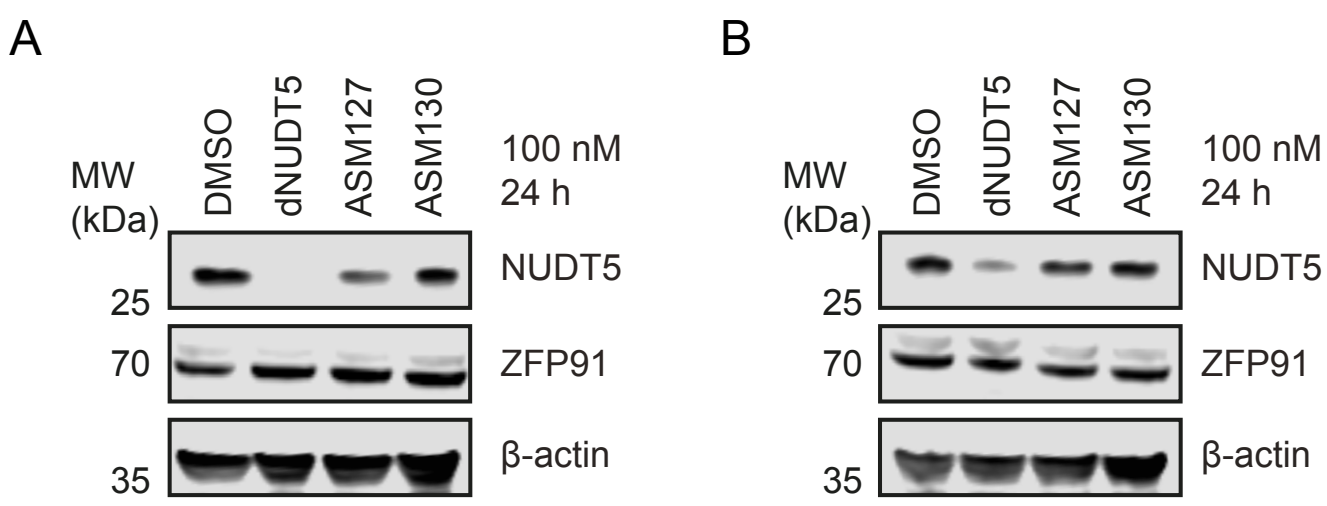

Supplementary Figure 8. dNUDT5 does not affect ZFP91 levels. PROTACs incubated at 100 nM for 24 h in HEK293 (A) and HAP1 (B) cells. Western blots are representative of three independent biological replicates (n = 3).

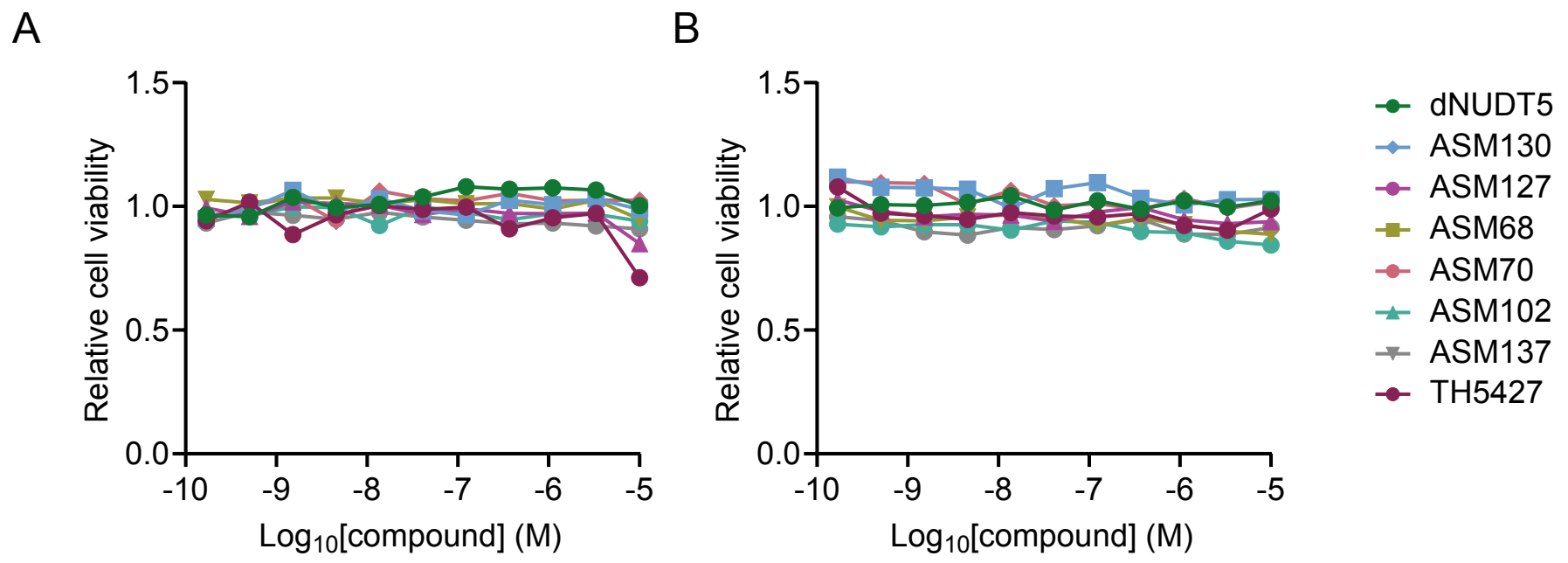

Supplementary Figure 9. NUDT5 PROTACs do not affect cell viability of HEK293 and HAP1 cells. Cell viability was measured by CellTiter Glo assay in (A) HEK293 and (B) HAP1 cells following PROTAC treatment for 72 h. Data are shown as mean values derived from three technical replicates. Graphs are representative of two independent biological replicates (n = 2).

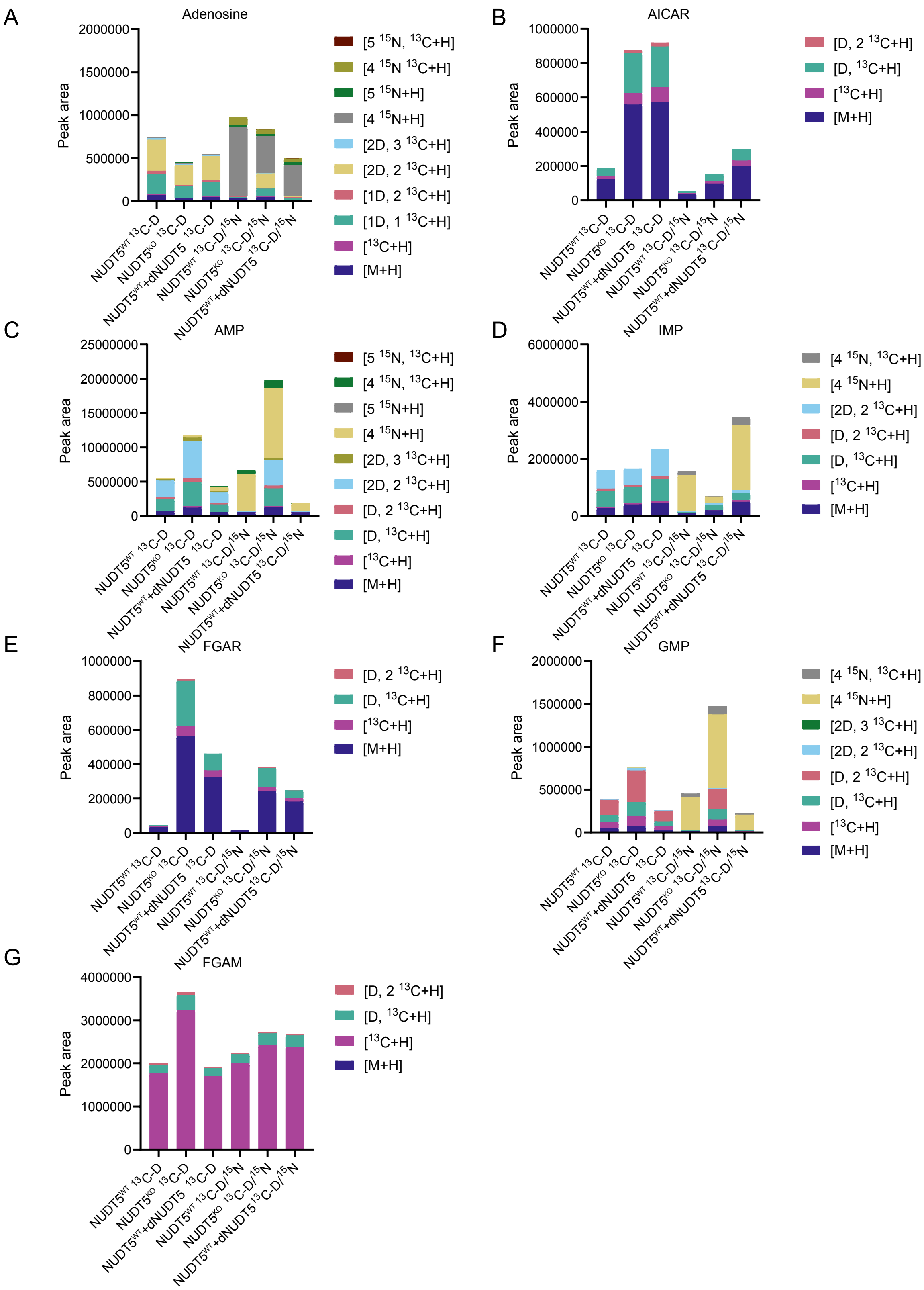

Supplementary Figure 10. Metabolomics of cells treated with dNUDT5. Peak areas (n = 2, biological replicates) of mass spectrometry signals of indicated metabolites in WT cells, WT cells treated with 100 nM dNUDT5 and WT NUDT5^{KO} cells and cultured in dialysed FBS and with 1 mM isotopically labelled formate or with 1 mM labelled formate and 100 μM labelled adenosine for 24 h.

Colored labels represent the incorporation of the labelled formate and adenosine into respective metabolites through the purine *de novo* synthesis or salvage pathway.

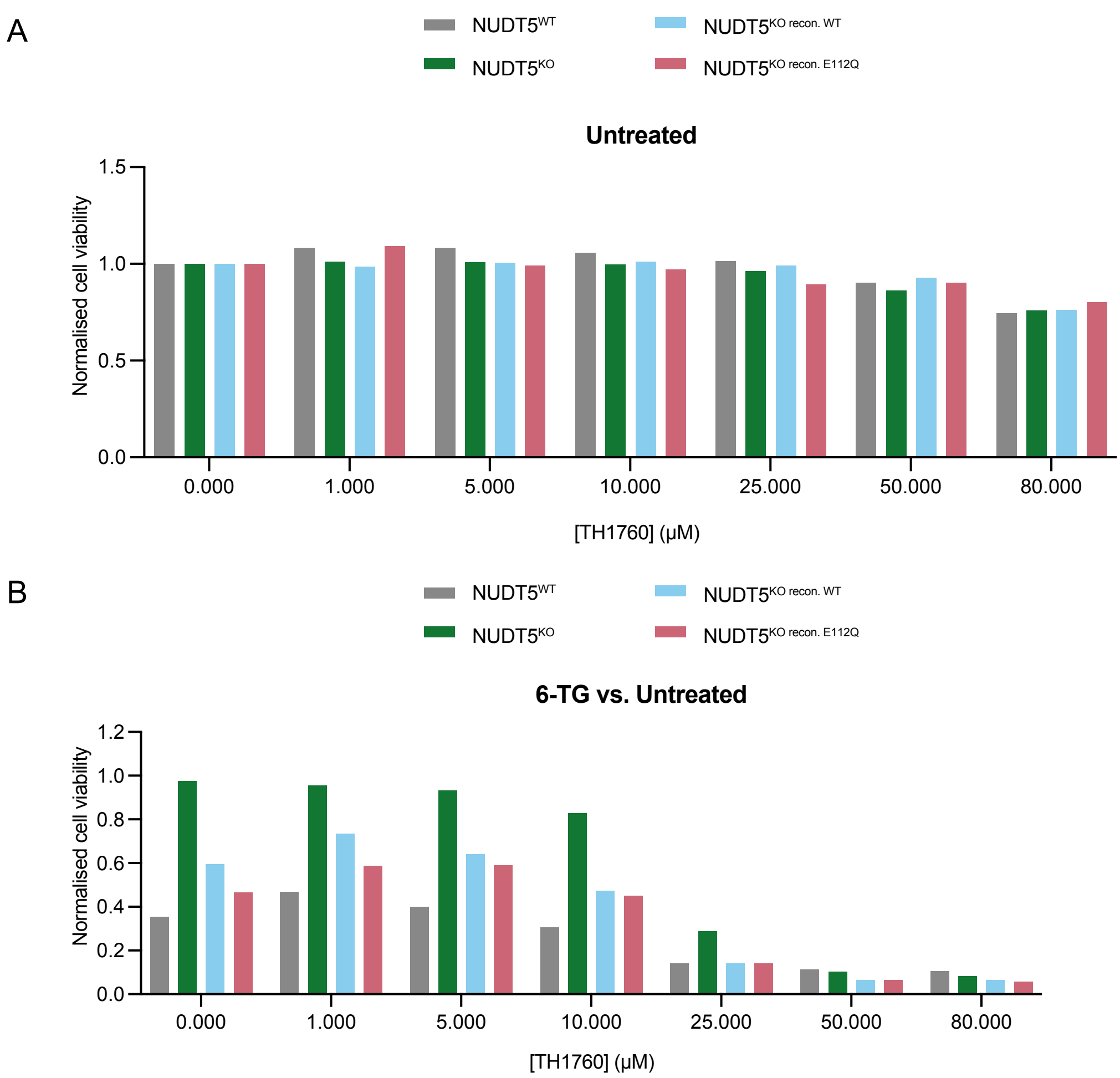

Supplementary Figure 11. Treatment with the NUDT15 inhibitor TH1760 increases cell sensitivity toward 6-thioguanine, which can be reversed by NUDT5 depletion. (A) Inhibition of NUDT15 at concentrations up to 50 µM does not affect HAP1 cell viability. Indicated cell lines were incubated with respective concentrations of TH1760 for 72 h (WT, wild-type; KO, knock-out; 6-TG, 6-thioguanine). Data are shown as mean values derived from three technical replicates. Graph is representative of two independent biological replicates (n = 2). (B) NUDT5 KO desensitizes cells towards 6-TG when NUDT15 is inhibited. The catalytic activity of NUDT5 (cf. NUDT5 KO vs. recon. NUDT5 WT and NUDT5 E112Q) is dispensable for the rescue mechanism. Indicated cell lines were treated with indicated amounts of TH1760 and in presence or absence of 6-TG (1 µM) for 72 h. Values were normalized to respective TH1760 concentration points. Data are shown as mean values derived from three technical replicates. Graph is representative of two independent biological replicates (n = 2).

Supplementary Tables

Supplementary Table 1. NanoBRET assay results for ibrutinib-derived PROTACs.

NUDT5 target engagement (TE) and PROTAC ternary complex formation measurements were conducted in HEK293 cells at the indicated time points. Results represent the mean of 3 technical replicates.

Compound	Ligand	Linker	E3 ligase ligand	EC_{50} (µM), TE 4h	Ternary complex formation below 1 µM (20 h)
**ASM32**	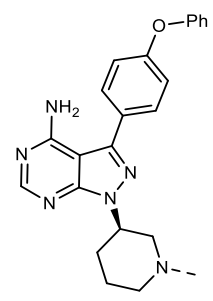	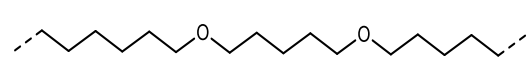	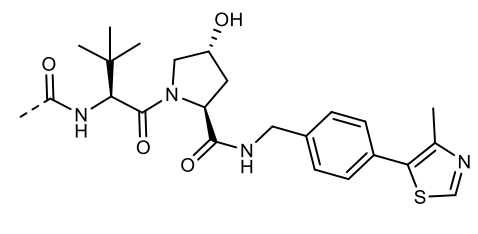	>10 µM	No
**ASM72**	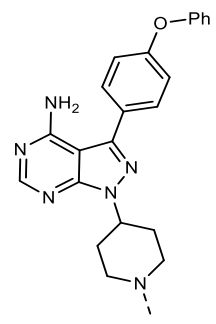	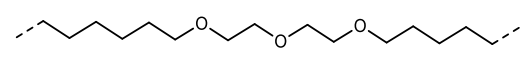	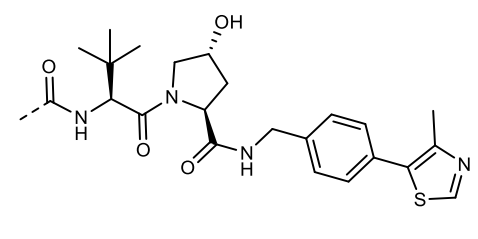	6	No
**ASM73**	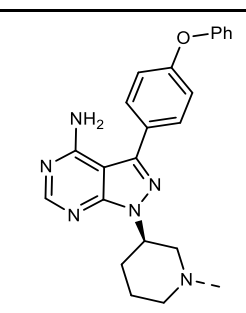	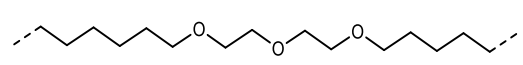	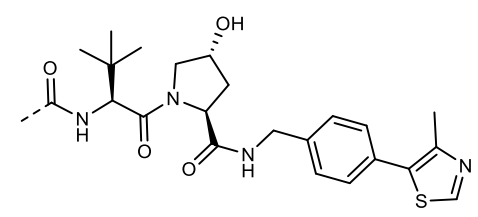	>10 µM	No
**ASM74**	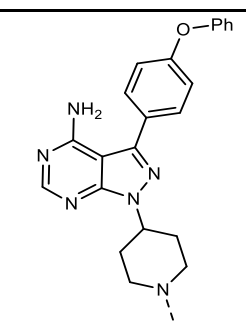	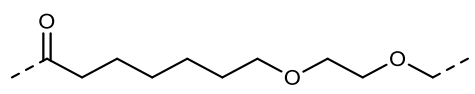	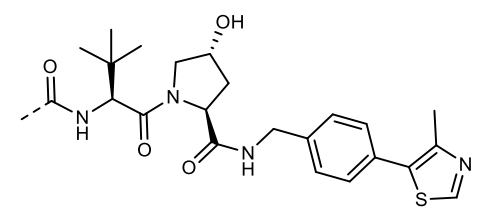	>10 µM	No
**ASM75**	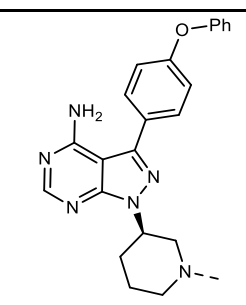	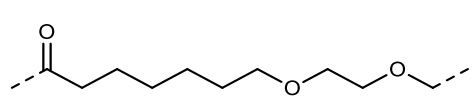	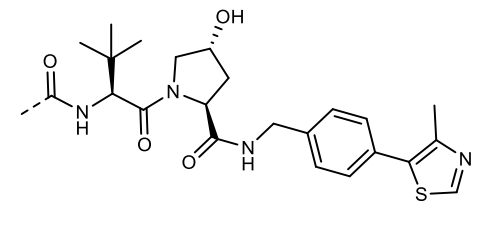	>10 µM	No
**MT-802**	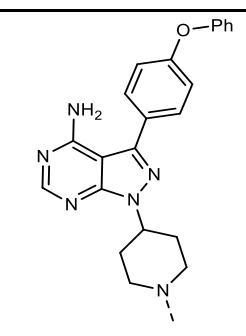	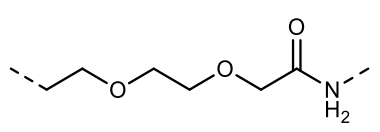	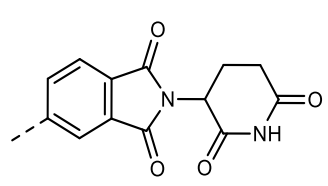	1.3	No

**RG27**	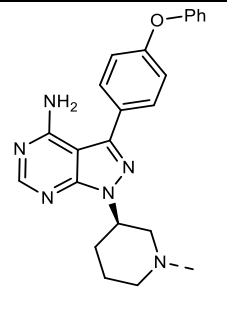	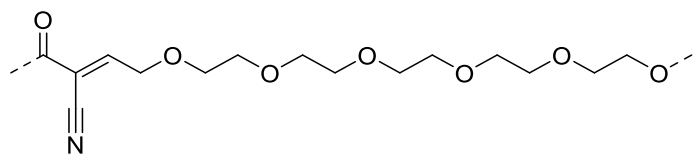	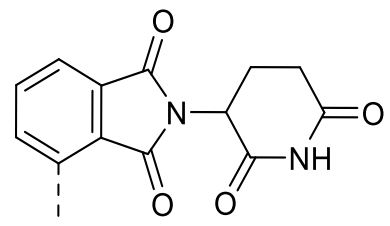	>10 μM	No
**RG38**	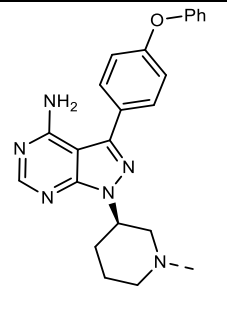	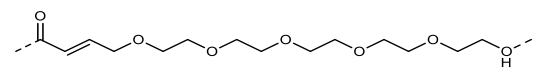	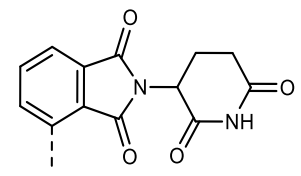	>10 μM	No
**RG52**	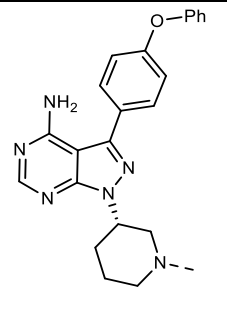	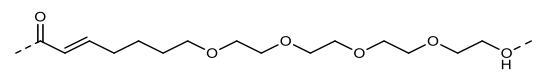	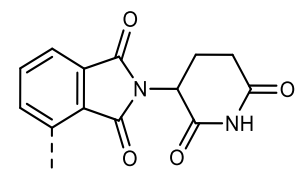	>10 μM	No
**RG55**	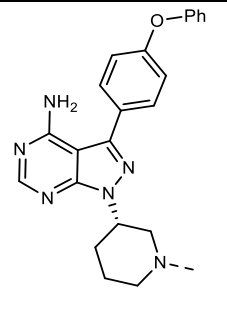	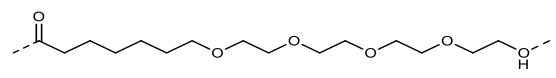	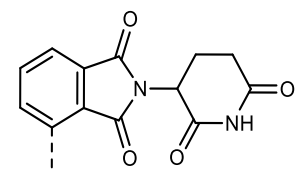	>10 μM	No
**RG66**	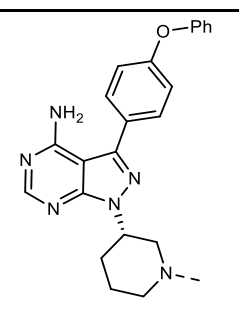	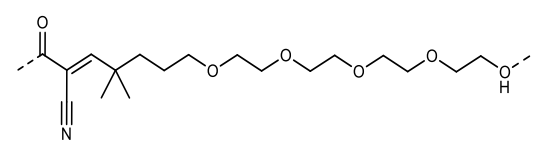	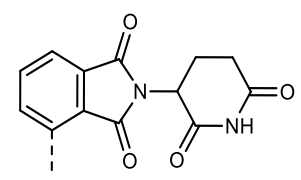	>10 μM	No
**P13I**[1]	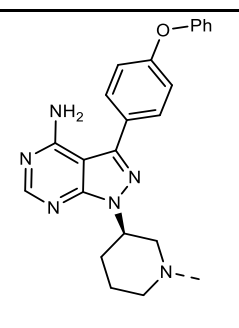	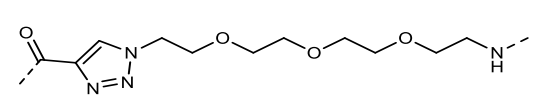	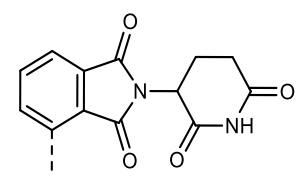	8.9	No

Supplementary Table 2. NanoBRET assay results for flexible linker-based PROTACs.

NUDT5 TE and PROTAC ternary complex formation measurements were conducted in HEK293 cells at the indicated time points. Results represent the mean of 3 technical replicates.

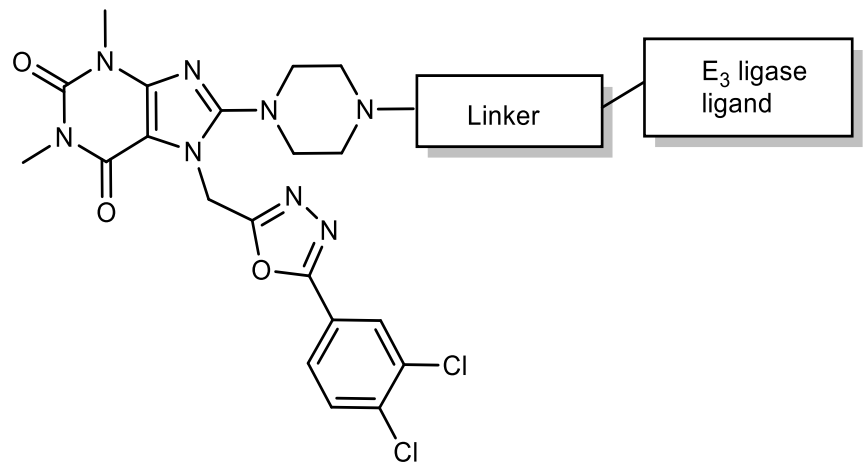

Compound	Linker	E3 ligase ligand	EC_{50} (nM) TE (4h)	Ternary complex formation below 1 µM (4 h)	Ternary complex formation below 1 µM (20 h)
**ASM076**	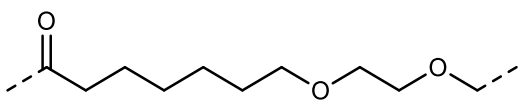	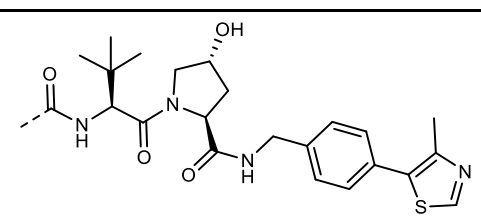	2000	NT[a]	No
**ASM131**	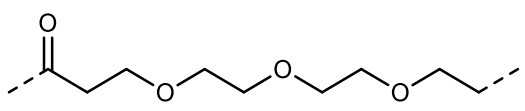	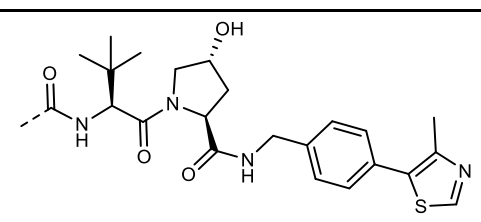	360	No	No
**ASM137**	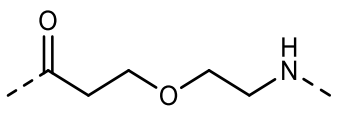	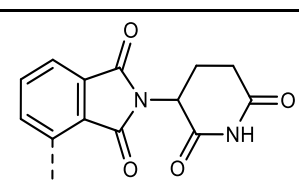	85	Yes	Yes
**ASM68**	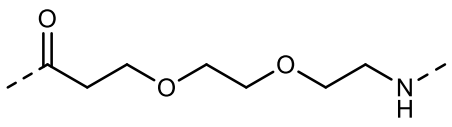	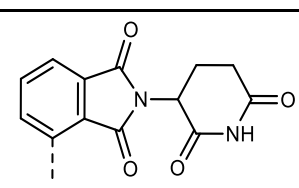	170	NT	Yes
**ASM127**	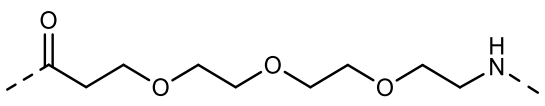	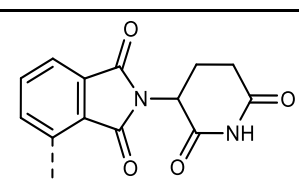	298	Yes	Yes
**ASM130**	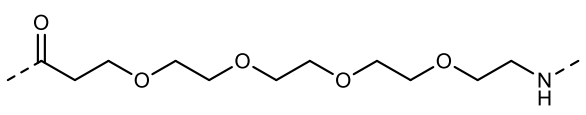	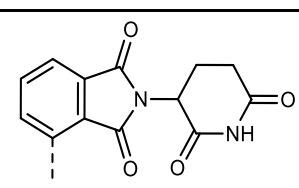	326	Yes	Yes
**ASM111**	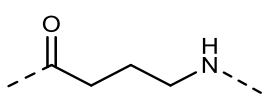	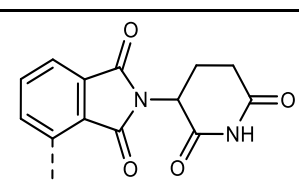	93	NT	Yes
**ASM124**	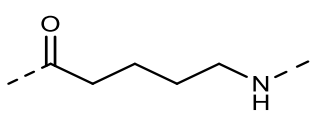	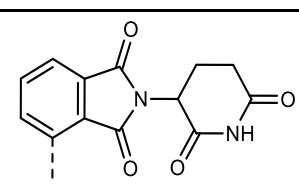	190	Yes	Yes
**ASM122**	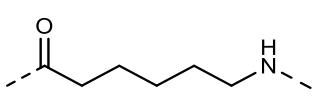	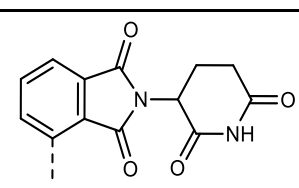	59	Yes	Yes
**ASM84**	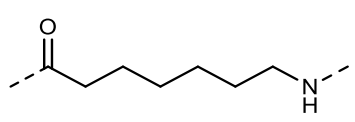	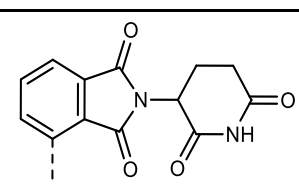	138	NT	Yes

**ASM136**	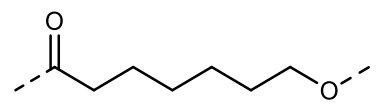	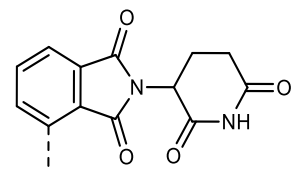	97	No	No
**ASM134**	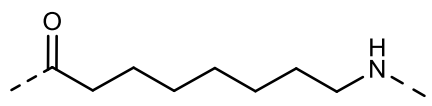	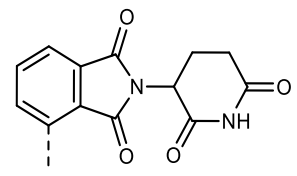	99	Yes	Yes
**ASM57**	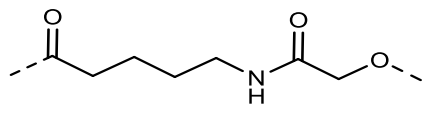	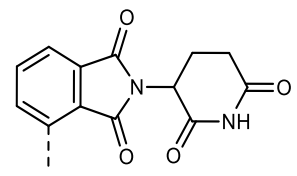	1770	NT	No
**ASM133**	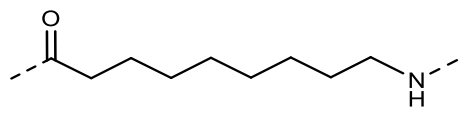	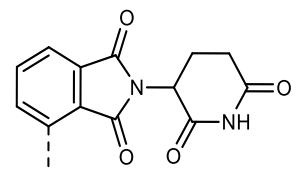	233	Yes	Yes
**ASM135**	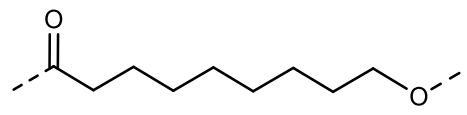	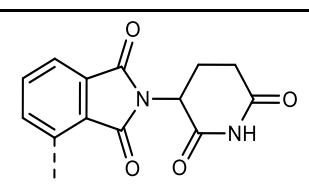	185	Yes	Yes

[a]NT, not tested

Supplementary Table 3. NanoBRET assay results for rigid linker-based PROTACs.
NUDT5 TE and PROTAC ternary complex formation measurements were conducted in HEK293 cells at the indicated time points. Results are the mean of 3 technical replicates.

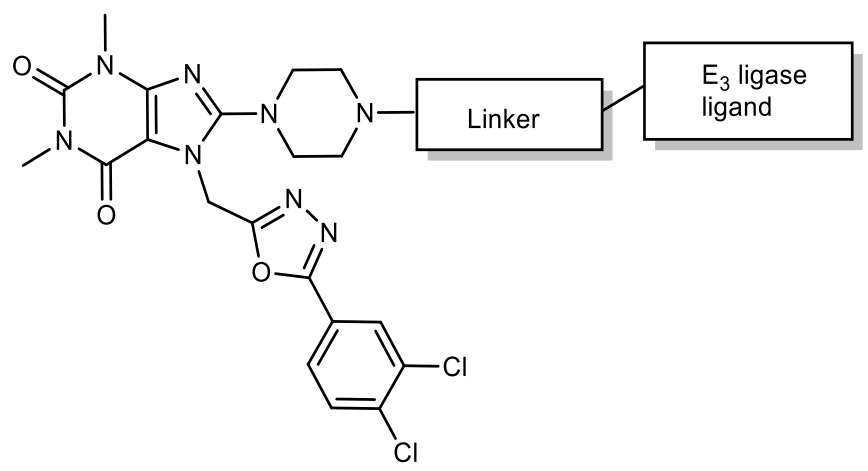

Compound	Linker	E3 ligase ligand	EC_{50} (nM) TE (4h)	Ternary complex formation below 1 µM (20 h)
**ASM105**	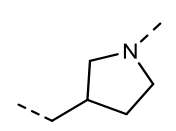	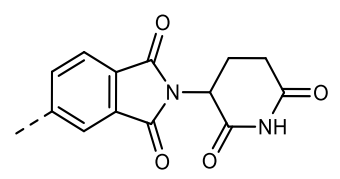	103	No
**ASM110**	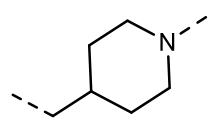	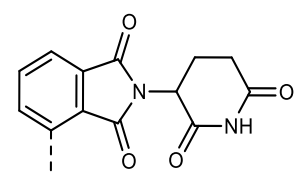	61	No
**ASM70**	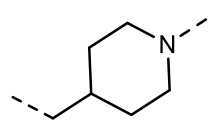	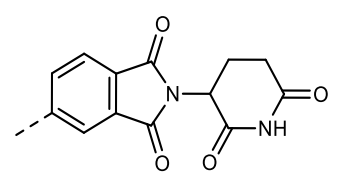	42	Yes
**ASM71**	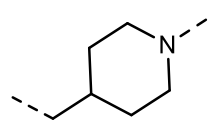	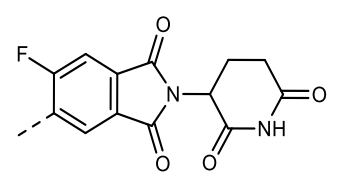	17	No
**ASM102**	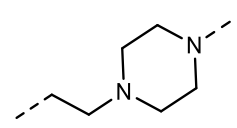	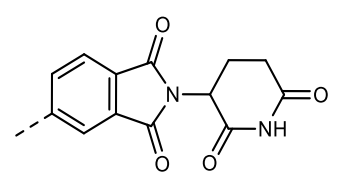	102	Yes

Supplementary Table 4. **Data collection and refinement statistics for the co-crystal structure of dNUDT5 bound to NUDT5.**

	dNUDT5<br>PDB ID: 9G6Y
**Data collection**	
Space group	C 1 2 1
Cell dimensions	
a, *b*, *c* (Å)	113.57, 40.21, 99.96
α, β, γ (°)	90.00, 121.92, 90.00
Resolution (Å)	37.11 - 1.90 (1.97 - 1.90)
R_{sym} or R_{merge}	0.1344 (2.550)
$I / \sigma I$	7.7 (0.3)
Completeness (%)	98.17% (83.55%)
Redundancy	6.8 (6.7)
**Refinement**	
Resolution (Å)	37.11 - 1.90 (1.97 - 1.90)
No. reflections	29973 (2514)
R_{work} / R_{free}	0.2124 (0.4314) / 0.2422 (0.4318)
No. atoms	3278
Protein	3004
Ligand/ion	73
Water	201
B-factors (Average)	46.11
Protein (mean)	45.62
Ligand/ion	64.36
Water	46.80
R.m.s. deviations	
Bond lengths (Å)	0.003
Bond angles (°)	0.55
Ramachandran Plot	
Favored (%)	97.11
Outliers (%)	0.00

*Values in parentheses are for highest-resolution shell.

Supplementary Methods

Synthesis and compound characterization

General remarks

Commercial reagents and solvents were purchased from commercial suppliers (Sigma-Aldrich, Fluorochem, Fisher Scientific) and used without further purification. All reactions involving moisture sensitive reagents were carried out under a nitrogen atmosphere using standard vacuum line techniques and dry solvents. An Elga DV 25 system was used for deionizing water. Thin layer chromatography was performed on aluminium plates coated with 60 F_{254} silica gel. Plates were visualised using UV light (254 nm). Flash column chromatography was performed on a Biotage Isolera One flash column chromatography platform. ^{1}H and ^{13}C NMR spectra were obtained using Bruker NMR spectrometers (400 MHz). The proton and carbon chemical shift values are reported in parts per million (ppm, δ scale) downfield from tetramethylsilane (TMS) and the indicated solvent. NMR spectra were processed and analyzed using MestReNova software. Spin multiplicities are given as s (singlet), d (doublet), t (triplet), q (quartet), dd (doublet of doublets), m (multiplet) and b (broad), coupling constants J are given in hertz (Hz), and signal area integration in natural numbers.

LC-MS was performed with a Kinetex 5 µm EVO C18 100A 100 × 3.0 mm column on a Waters SFO and 515 HPLC pump and Waters Binary Gradient 2545 device using linear gradient of solvent A (93% water, 5% acetonitrile and 2% of 0.5 M ammonium acetate pH 6.0) and solvent B (18% H_2O, 80% acetonitrile and 2% ammonium acetate pH 6.0), eluting at a flow rate of 2 mL/min: 5% B for 0.35 min, 5% B to 95% B for 1 min, 95% to 5% B for 0.1 min and 5% B for 0.8 min. LCMS was used as a measure of compound purity using either UV absorbance (Waters UV/visible Detector 2489), ELSD signal (Waters ELS Detector 2424) or ESI+ TIC (SQ Detector 2). Preparative HPLC was performed on the same system with a Kinetex 5 µm EVO C18 100A 150 x 21.2 mm column using linear gradient of solvent A over 20 min from 85% to 10% eluting at a flow rate of 20 mL/min. LC-MS was acquired using Waters FractionLynx software and processed using MestReNova software. HRMS was acquired with Agilent 6530 RapidFire QTOF mass spectrometer in 384-well polypropylene plates (Greiner, 781280) using an assay buffer consisting of 10 mM ammonium formate pH 7.5. The plate was transferred to a RapidFire RF360 high-throughput sampling robot. Samples were aspirated under vacuum and loaded onto a C4 solid-phase extraction (SPE) cartridge equilibrated and washed for 4.5 s with 0.1% formic acid in LC-MS grade water to remove non-volatile buffer components. After aqueous wash, analytes of interest were eluted from the C4 SPE onto an Agilent 6530 accurate mass Q-TOF in an organic elution step, 85% acetonitrile and 0.1% formic acid in LC-MS grade water. Ion data for the compounds were extracted, and the compound formulas were generated using MassHunter Qualitative Analysis B.06.00 (Agilent). The purity for all final compounds was determined to be >95% by HPLC.

General procedure 1

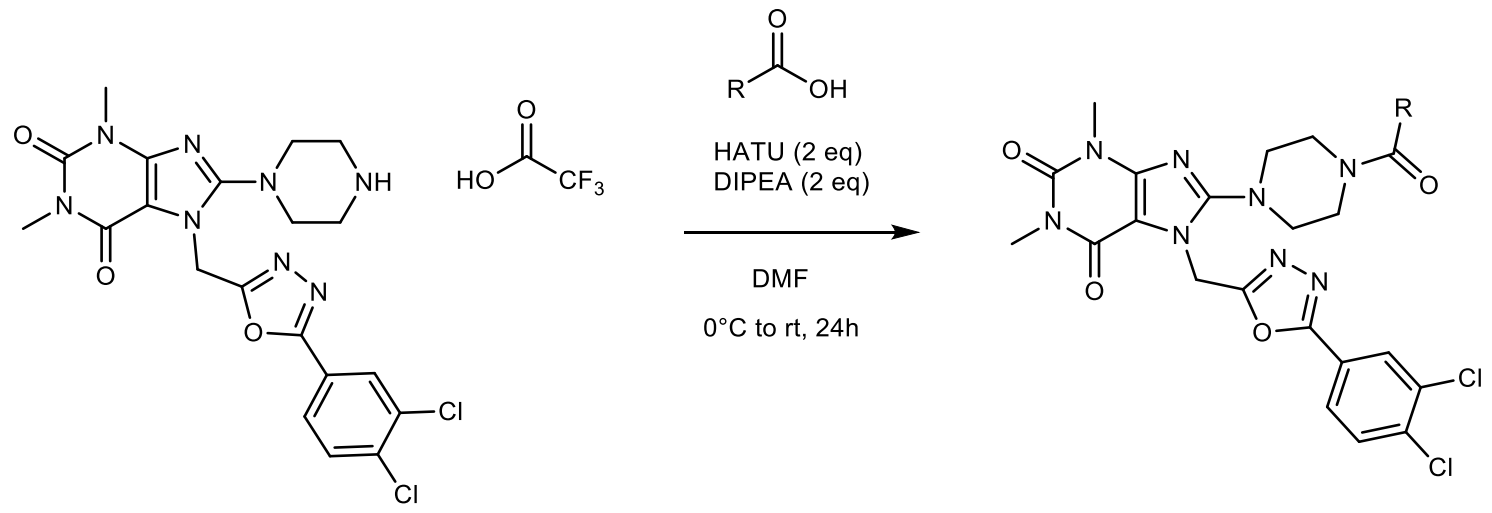

Under nitrogen, HATU (2 eq) was added to a solution of the corresponding acid (1.1-1.2 eq) and DIPEA (2 or 3 eq) in DMF (0.03 M) at 0°C. The mixture was stirred for 15 min at 0°C. A solution of TH5427.TFA (1.2 eq) and DIPEA (1 eq) in DMF (0.03 M) was then added dropwise. The mixture was stirred for 24h at room temperature. After concentration under reduced pressure, the crude was purified by prep HPLC to afford the expected product.

General procedure 2

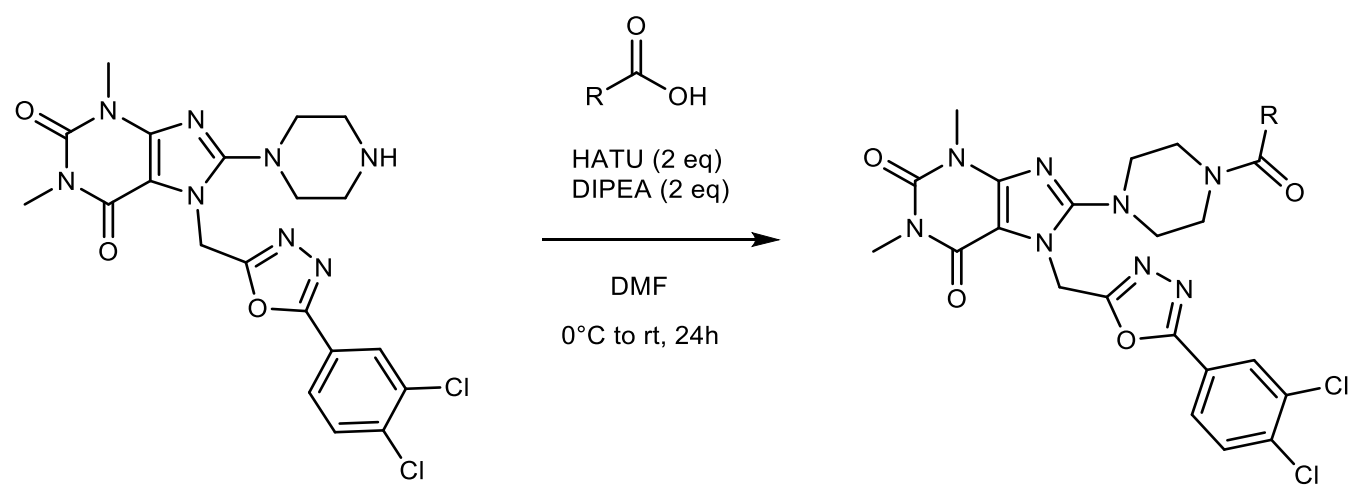

Under nitrogen, HATU was added to a solution of acid and DIPEA (2 or 3 eq) in DMF (0.010 M) at 0°C. The mixture was stirred for 15 min at 0°C. The amine was then added portion wise. The mixture was stirred for 24h at room temperature. After concentration under reduced pressure, the crude was purified by prep HPLC to afford the expected products.

General procedure 3

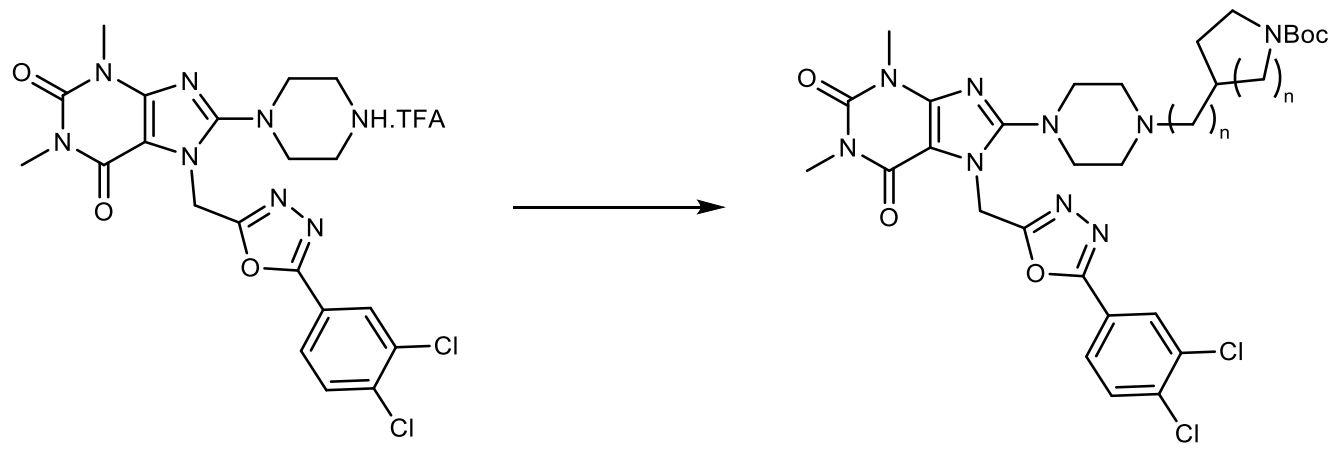

Under nitrogen, sodium triacetoxyborohydride was added to a solution of the corresponding aldehyde and TH5427.TFA in THF. The resulting mixture was stirred at room temperature overnight. The crude was purified by flash column chromatography (Si-35, DCM/MeOH, gradient from 100/0 to 95/5) to afford the expected product.

General procedure 4

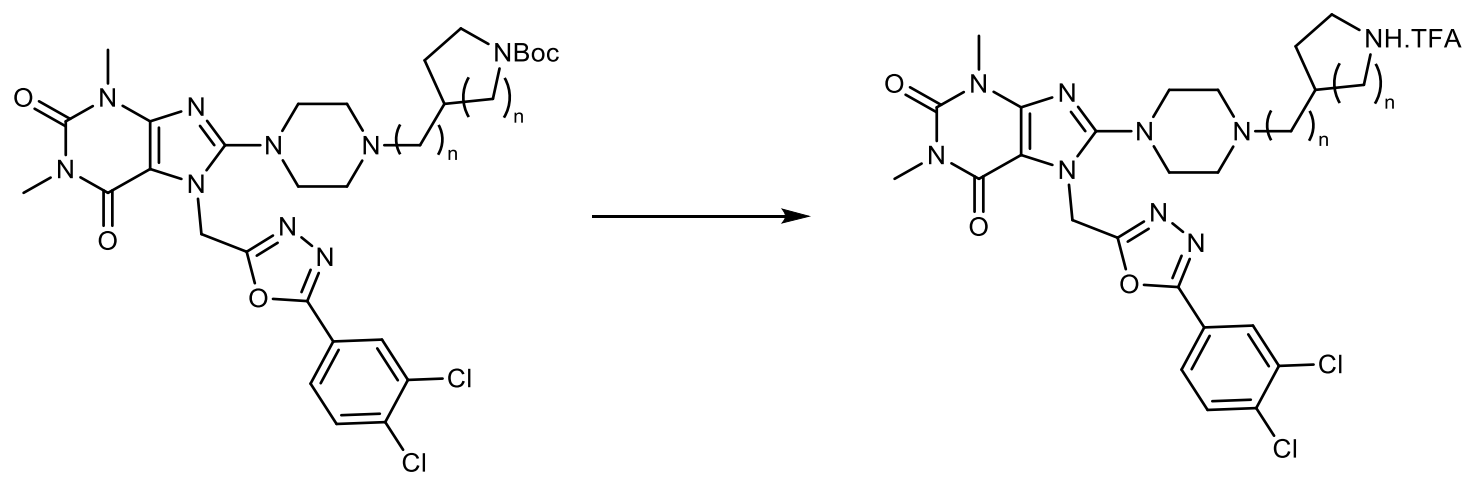

Under nitrogen, TFA was added to a solution of *N*-Boc protected compound in DCM at 0°C. The solution was stirred for the indicated time at room temperature. After completion, the crude was concentrated under reduced pressure and used in the next step without further purification.

General procedure 5

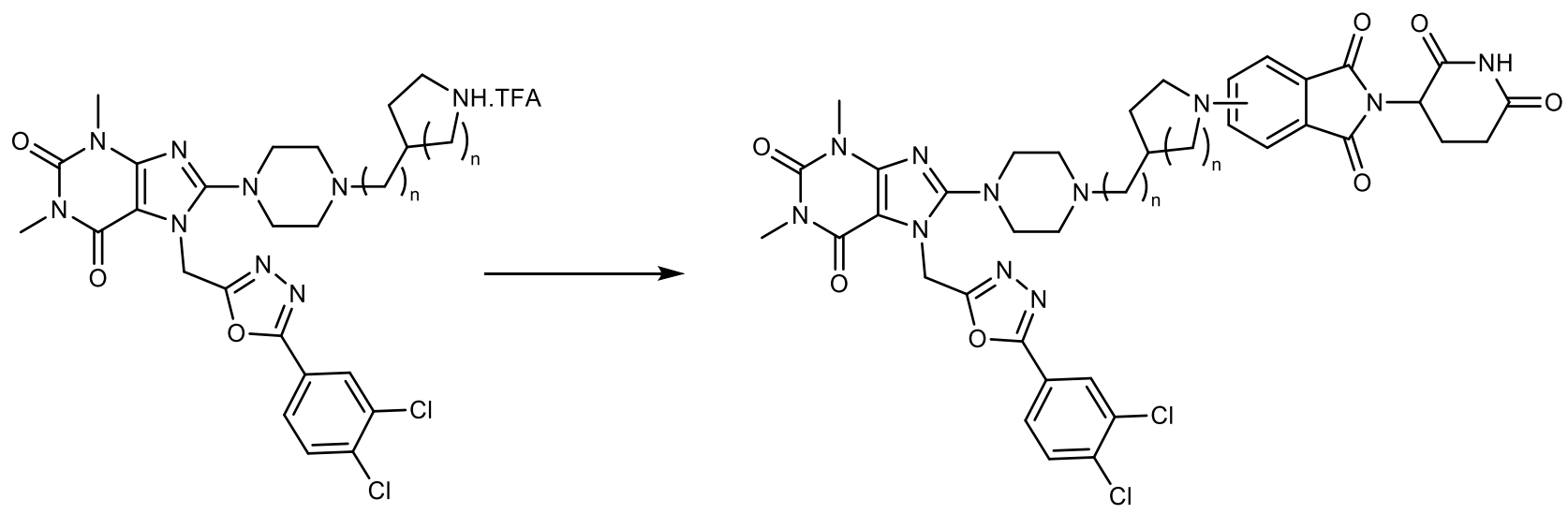

Under nitrogen, TRIETHYLAMINE (3 eq) was added to a solution of amine trifluoroacetic salt (1 eq) and fluoro thalidomide (1 eq) in DMSO. The mixture was heated under microwave at 200°C for 15-20 min. The crude was purified by prep HPLC to afford the expected product.

General procedure 6

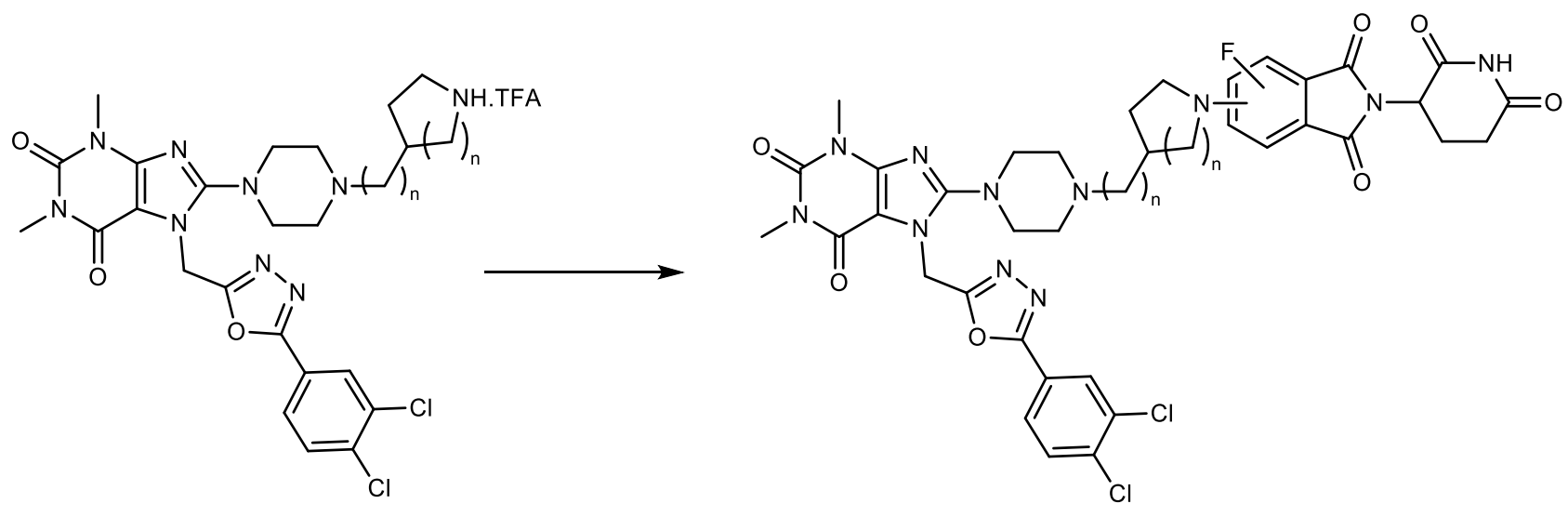

Under nitrogen, DIPEA (3 eq) was added to a solution of amine (1 eq) and mono- or di-fluoro thalidomide (1 eq) in DMSO. The mixture was heated at 100°C overnight. The crude was purified by prep HPLC to afford the expected product.

ASM57

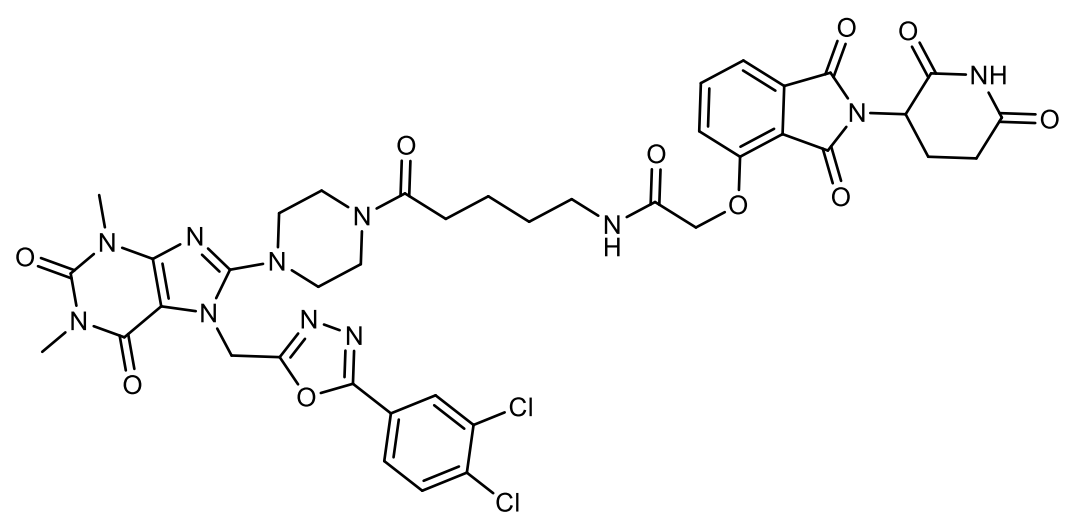

N-(5-(4-(7-((5-(3,4-dichlorophenyl)-1,3,4-oxadiazol-2-yl)methyl)-1,3-dimethyl-2,6-dioxo-2,3,6,7-tetrahydro-1*H*-purin-8-yl)piperazin-1-yl)-5-oxopentyl)-2-((2-(2,6-dioxopiperidin-3-yl)-1,3-dioxoisoindolin-4-yl)oxy)acetamide

ASM57 was prepared according to the general procedure 1 using TH5427.TFA (28.9 mg, 0.034 mmol, 1.1 eq), 5-(2-((2-(2,6-dioxopiperidin-3-yl)-1,3-dioxoisoindolin-4-yl)oxy)acetamido)pentanoic acid (10.3 mg, 0.031 mmol, 1 eq), DIPEA (22 µL, 0.12 mmol, 4 eq) and HATU (23.6 mg, 0.062 mmol, 2 eq). After purification, a white solid was obtained (3.3 mg, 0.024 mmol, 12%).

^{1}H NMR (400 MHz, $CDCl_3$) δ 8.99 (s, 1H), 8.10 (d, J = 2Hz, 1H), 7.85 (dd, J1 = 8.4 Hz, J2 = 2.0 Hz, 1H), 7.74 (dd, J1 = 7.4 Hz, J2 = 8.4 Hz, 1H), 7.68-7.65 (m, 1H), 7.60-7.54 (m, 2H), 7.21 (d, J = 8.4 Hz, 1H), 5.69 (s, 2H), 4.98-4.94 (m, 1H), 4.70-4.60 (m, 2H), 3.75-3.67 (m, 2H), 3.59-3.56 (m, 2H), 3.54 (s, 3H), 3.47-3.42 (m, 1H), 3.37 (s, 3H), 3.35-3.32 (m, 1H), 3.27-3.24 (m, 4H), 2.92-2.85 (m, 1H), 2.82-2.73 (m, 2H), 2.42-2.34 (m, 2H), 2.20-2.15 (m, 1H), 1.66-1.62 (m, 4H).

^{13}C NMR (101 MHz, $CDCl_3$) δ 171.6, 171.3, 168.4, 167.1, 166.7, 166.4, 164.2, 162.0, 155.9, 155.0, 154.9, 151.7, 147.7, 137.3, 137.0, 134.0, 133.7, 131.5, 128.9, 126.2, 123.1, 120.4, 118.5, 117.8, 105.0, 68.8, 50.6 (2C), 49.5, 45.0, 40.1, 39.0, 32.8, 31.5, 30.2, 30.0, 29.1, 28.0, 22.9, 22.5.

HPLC-MS t_R = 1.475 min (93.2 %), ESI+ m/z 904.630; 906.071 $[M+H]^+$

ASM68

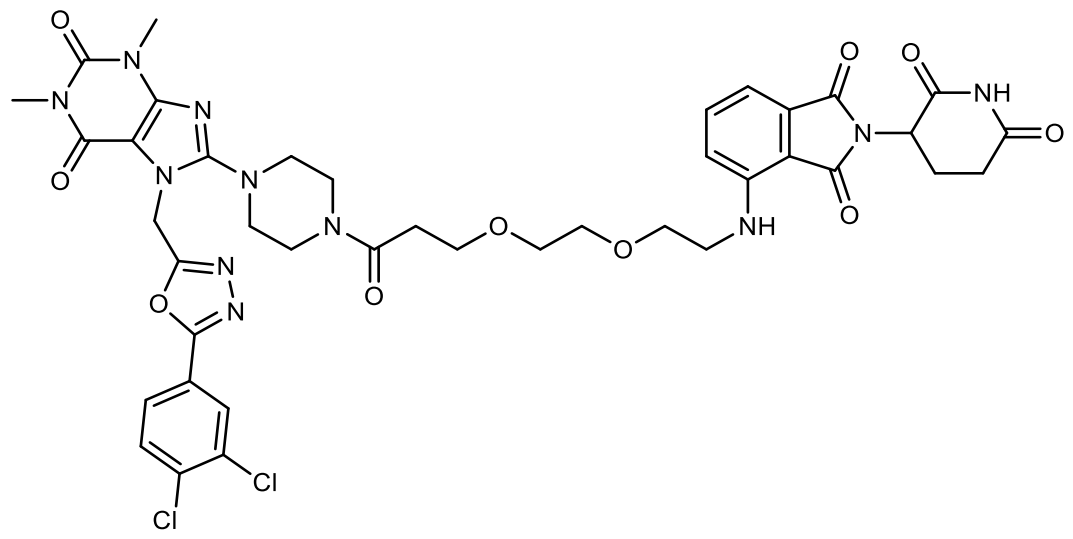

7-((5-(3,4-dichlorophenyl)-1,3,4-oxadiazol-2-yl)methyl)-8-(4-(3-(2-(2-((2-(2,6-dioxopiperidin-3-yl)-1,3-dioxoisoindolin-4-yl)amino)ethoxy)ethoxy)propanoyl)piperazin-1-yl)-1,3-dimethyl-3,7-dihydro-1*H*-purine-2,6-dione

ASM68 was prepared according to the general procedure 1 using TH5427.TFA (20 mg, 0.033 mmol, 1.1 eq), 3-(2-(2-((2-(2,6-dioxopiperidin-3-yl)-1,3-dioxoisoindolin-4-yl)amino)ethoxy)ethoxy) propanoic acid (13 mg, 0.030 mmol, 1 eq), DIPEA (21 µL, 0.12 mmol, 4 eq) and HATU (23 mg, 0.060 mmol, 2 eq). After purification, a yellow solid was obtained (21.4 mg, 0.024 mmol, 79%).

^{1}H NMR (400 MHz, $CDCl_3$) δ 8.88 (s, 1H), 8.07 (d, J = 2.0 Hz, 1H), 7.83 (dd, J1 = 8.4 Hz, J2 = 2.0 Hz, 1H), 7.57 (d, J = 8.4 Hz, 1H), 7.45 (dd, J1 = 7.0 Hz, J2 = 8.4 Hz, 1H), 7.06 (d, J = 7.0 Hz, 1H), 6.88 (d, J = 8.4 Hz, 1H), 6.45 (bs, 1H), 5.68 (s, 2H), 4.93-4.88 (m, 1H), 3.79 (t, J = 6.5 Hz, 2H), 3.71 (bt, J = 5.3 Hz, 4H), 3.65-3.63 (m, 4H), 3.61-3.59 (m, 2H), 3.53 (s, 3H), 3.46-3.42 (m, 2H), 3.35 (s, 3H), 3.29-3.23 (m, 4H), 2.88-2.81 (m, 1H), 2.77-2.72 (m, 2H), 2.64-2.60 (m, 2H), 2.17-2.10 (m, 1H).

^{13}C NMR (101 MHz, $CDCl_3$) δ 171.5, 170.1, 169.4, 168.8, 167.6, 164.1, 162.1, 155.9, 155.0, 151.6, 147.7, 146.88, 136.9, 136.1, 133.9, 132.5, 131.5, 128.8, 126.1, 123.0, 116.9, 111.7, 110.3, 104.9, 70.6, 70.5, 69.5, 67.7, 50.8, 50.3, 49.00, 45.2, 42.5, 41.0, 40.1, 33.7, 31.5, 30.0, 28.0, 22.9.

HPLC-MS t_R = 1.54 min (100%), ESI+ m/z 907.379 $[M+H]^+$

HRMS was calculated for $C_{40}H_{42}Cl_2N_{11}O_{10}$: 906.2493, found: 906.2487

ASM76

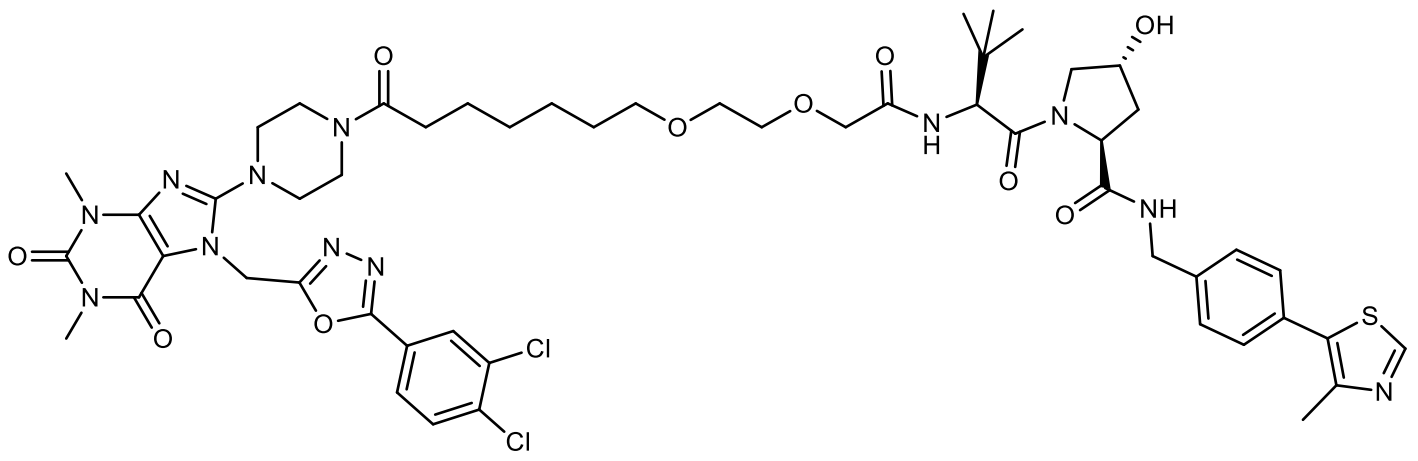

(2*S*,4*R*)-1-((*S*)-2-(2-(2-((7-(4-(7-((5-(3,4-dichlorophenyl)-1,3,4-oxadiazol-2-yl)methyl)-1,3-dimethyl-2,6-dioxo-2,3,6,7-tetrahydro-1*H*-purin-8-yl)piperazin-1-yl)-7-oxoheptyl)oxy)ethoxy)acetamido)-3,3-dimethylbutanoyl)-4-hydroxy-*N*-(4-(4-methylthiazol-5-yl)benzyl)pyrrolidine-2-carboxamide

ASM76 was prepared according to the general procedure 1 using TH5427.TFA (12 mg, 0.020 mmol, 1.1 eq), 7-(2-(2-(((S)-1-((2S,4R)-4-hydroxy-2-((4-(4-methylthiazol-5-yl)benzyl)carbamoyl) pyrrolidin-1-yl)-3,3-dimethyl-1-oxobutan-2-yl)amino)-2-oxoethoxy)ethoxy)heptanoic acid (12 mg, 0.018 mmol, 1 eq), DIPEA (9 µL, 0.054 mmol, 3 eq) and HATU (13.8 mg, 0.036 mmol, 2 eq). After purification, a white solid was obtained (12 mg, 0.011 mmol, 58%).

^{1}H NMR (400 MHz, DMSO-*d6*) δ (major rotamer described) 8.96 (s, 1H), 8.59 (t, J = 6 Hz, 1H), 8.14 (bd, J = 1.9 Hz, 1H) 7.93 (dd, J1 = 1.9 Hz, J2 = 8.5 Hz, 1H), 7.87 (d, J = 8.5 Hz, 1H), 7.42-7.36 (m, 5H), 5.72 (s, 2H), 5.15 (s, 1H), 4.55 (d, J = 9.5 Hz, 1H), 4.46-4.35 (m, 3H), 4.28-4.20 (m, 1H), 3.95 (s, 2H), 3.68-3.63 (m, 1H), 3.62-3.59 (m, 3H), 3.56-3.50 (m, 7H), 3.39 (s, 3H), 3.26-3.19 (m, 4H), 3.14 (s, 3H), 2.42 (s, 3H), 2.27 (t, J = 7.5 Hz, 2H), 2.08-2.03 (m, 1H), 1.93-1.87 (m, 1H), 1.49-1.40 (m, 4H), 1.26-1.22 (m, 4H), 0.94 (s, 9H). *OH not observed*

^{13}C NMR (400 MHz, DMSO-*d6*) δ 171.8, 170.9, 169.1, 168.6, 163.1, 162.7, 155.9, 153.8, 151.4, 150.9, 147.7, 147.2, 139.4, 135.0, 132.5, 132.0, 131.2, 129.7, 128.9, 128.7 (2C), 128.1, 127.5 (2C), 126.6, 123.5, 104.1, 70.6, 70.5, 69.6, 69.2, 68.9, 58.8, 56.6, 55.7, 50.0, 49.7, 44.4, 41.7, 40.4, 37.9, 35.7, 32.2, 29.6, 29.1, 28.6, 27.4, 26.2 (3C), 25.5, 24.7, 15.9.

HPLC-MS t_R = 1.59 min (99.3%), ESI+ m/z 1134.809; 1136.456 $[M+H]^+$

ASM84

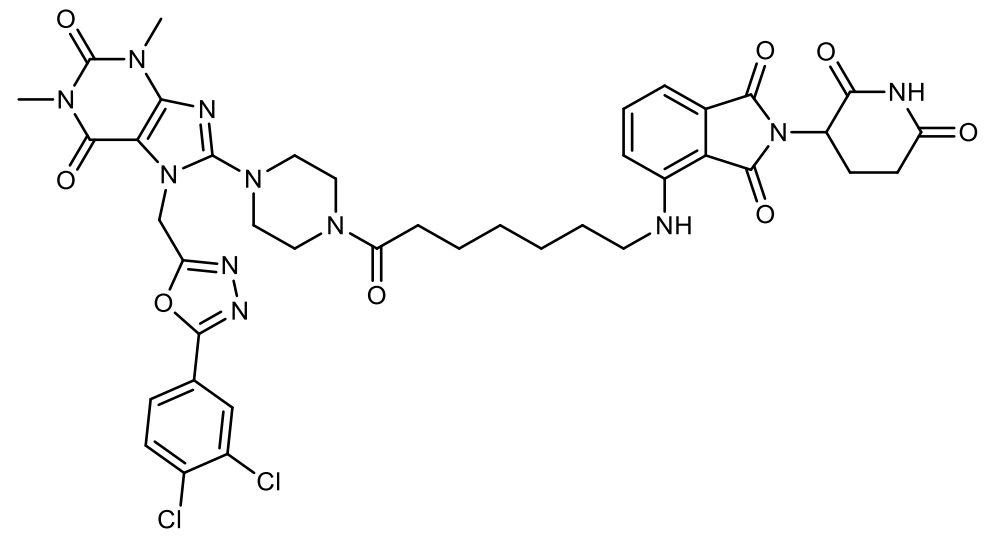

7-((5-(3,4-dichlorophenyl)-1,3,4-oxadiazol-2-yl)methyl)-8-(4-(7-((2-(2,6-dioxopiperidin-3-yl)-1,3-dioxoisoindolin-4-yl)amino)heptanoyl)piperazin-1-yl)-1,3-dimethyl-3,7-dihydro-1*H*-purine-2,6-dione

ASM84 was prepared according to the general procedure 1 using TH5427.TFA (16.6 mg, 0.027 mmol, 1.1 eq), 7-((2-(2,6-dioxopiperidin-3-yl)-1,3-dioxoisoindolin-4-yl)amino)heptanoic acid (10 mg, 0.025 mmol, 1 eq), DIPEA (13 µL, 0.075 mmol, 3 eq) and HATU (18.9 mg, 0.050 mmol, 2 eq). After purification, a yellow solid was obtained (18.2 mg, 0.021 mmol, 84%).

^{1}H NMR (400 MHz, $CDCl_3$) δ 8.46 (s, 1H), 8.10 (d, J = 2Hz, 1H), 7.86 (dd, J1 = 2.0 Hz, J2 = 8.5 Hz, 1H), 7.60 (d, J = 8.5 Hz, 1H) 7.49 (dd, J1 = 7.0 Hz, J2 = 8.4 Hz, 1H) 7.08 (d, J = 7.0 Hz, 1H) 6.88 (d, J = 8.4 Hz, 1H), 6.22 (d, J = 5.7 Hz, 1H), 5.70 (s, 2H), 4.93-4.89 (m, 1H), 3.74-3.71 (m, 2H), 3.60-3.57 (m, 2H), 3.55 (s, 3H), 3.37 (s, 3H), 3.29- 3.24 (m, 6H), 2.90-2.84 (m, 1H), 2.81-2.69 (m, 2H), 2.34 (t, J = 7.3 Hz, 2H), 2.15-2.10 (m, 1H), 1.71-1.63 (m, 4H), 1.49-1.36 (m, 4H).

^{13}C NMR (101 MHz, $CDCl_3$) δ 171.7, 171.2, 169.6, 168.6, 167.7, 164.2, 162.0, 155.9, 155.0, 151.7, 147.7, 147.1, 137.0, 136.3, 134.0, 132.6, 131.5, 128.9, 126.2, 123.0, 116.8, 111.5, 110.0, 105.0, 50.7, 50.6, 49.0, 45.0, 42.6, 40.9, 40.1, 33.1, 31.5, 30.0, 29.1, 29.1, 28.0, 26.8, 25.0, 22.9.

HPLC-MS t_R = 1.68 min (99%), ESI+ m/z 874.120 $[M+H]^+$

ASM111

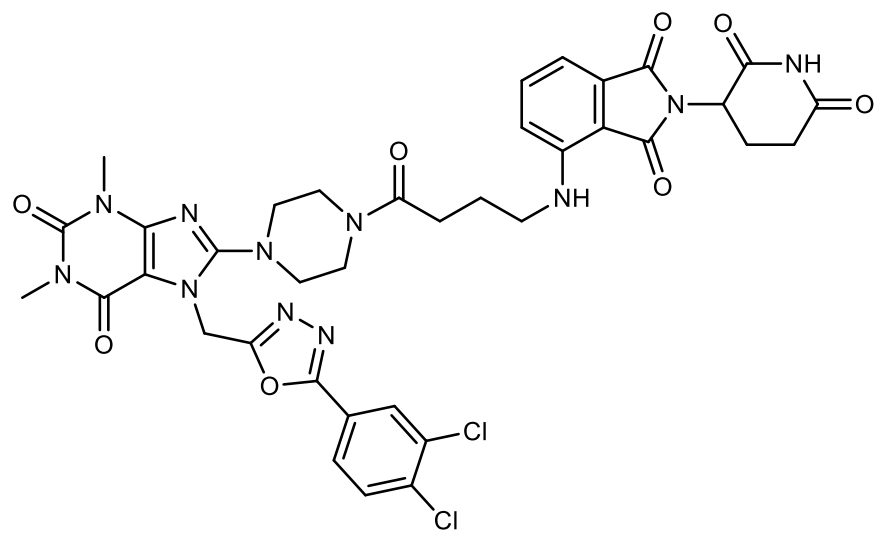

7-((5-(3,4-dichlorophenyl)-1,3,4-oxadiazol-2-yl)methyl)-8-(4-(4-((2-(2,6-dioxopiperidin-3-yl)-1,3-dioxoisoindolin-4-yl)amino)butanoyl)piperazin-1-yl)-1,3-dimethyl-3,7-dihydro-1*H*-purine-2,6-dione

ASM111 was prepared according to the general procedure 1 using TH5427.TFA (27.8 mg, 0.046 mmol, 1.1 eq), 4-((2-(2,6-dioxopiperidin-3-yl)-1,3-dioxoisoindolin-4-yl)amino)butanoic acid (15 mg, 0.042 mmol, 1 eq), DIPEA (22 µL, 0.125 mmol, 3 eq) and HATU (31.7 mg, 0.083 mmol, 2 eq). After purification, a yellow solid was obtained (10.1 mg, 0.012 mmol, 29%).

^{1}H NMR (400 MHz, $CDCl_3$) δ 8.44 (s, 1H), 8.12 (d, J = 2Hz, 1H), 7.87 (dd, J1 = 2.0 Hz, J2 = 8.4 Hz, 1H), 7.61 (d, J = 8.4 Hz, 1H) 7.51 (dd, J1 = 7.0 Hz, J2 = 8.4 Hz, 1H) 7.11 (d, J = 7.0 Hz, 1H) 6.98 (d, J = 8.4 Hz, 1H), 5.70 (S, 2H), 4.93-4.89 (m, 1H), 3.81-3.69 (m, 2H), 3.63-3.56 (m, 2H), 3.56 (s, 3H), 3.40 (t, J = 6.8 Hz, 2H), 3.38 (s, 3H), 3.30- 3.25 (m, 4H), 2.91-2.77 (m, 2H), 2.73-2.67 (m, 1H), 2.45 (t, J = 6.8 Hz, 2H), 2.17-2.12 (m, 1H), 2.09-2.03 (m, 2H). *NH-Ar not observed*

^{13}C NMR (101 MHz, $CDCl_3$) δ 171.2, 170.7, 169.6, 168.7, 167.7, 164.2, 162.0, 155.8, 155.0, 151.7, 147.7, 147.0, 137.0, 136.4, 134.0, 132.6, 131.5, 128.9, 126.2, 123.0, 117.1, 111.9, 110.4, 105.0, 50.6, 50.5, 49.0, 44.9, 42.2, 41.1, 40.1, 31.6, 30.0, 29.9, 28.0, 24.2, 23.0.

ASM122

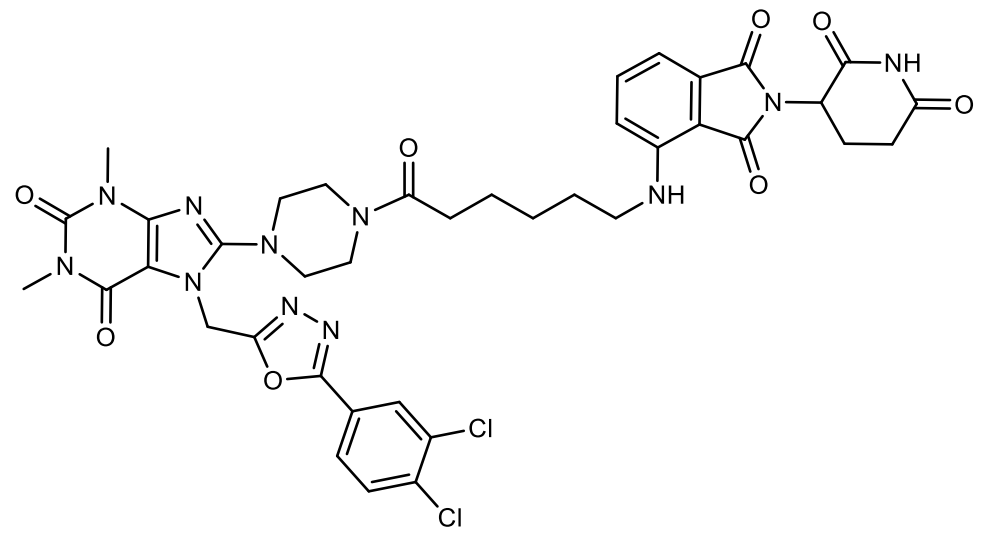

7-((5-(3,4-dichlorophenyl)-1,3,4-oxadiazol-2-yl)methyl)-8-(4-(6-((2-(2,6-dioxopiperidin-3-yl)-1,3-dioxoisoindolin-4-yl)amino)hexanoyl)piperazin-1-yl)-1,3-dimethyl-3,7-dihydro-1*H*-purine-2,6-dione

ASM122 was prepared according to the general procedure 1 using TH5427.TFA (33.2 mg, 0.055 mmol, 1.1 eq), 6-((2-(2,6-dioxopiperidin-3-yl)-1,3-dioxoisoindolin-4-yl)amino)hexanoic acid (25 mg, 0.050 mmol, 1 eq), DIPEA (26 µL, 0.15 mmol, 3 eq) and HATU (37.9 mg, 0.1 mmol, 2 eq). After purification, a yellow solid was obtained (8 mg, 0.009 mmol, 19%).

^{1}H NMR (400 MHz, $CDCl_3$) δ 8.19 (s, 1H), 8.11 (d, J = 2.0 Hz, 1H), 7.86 (dd, J = 2.0 Hz, J = 8.5 Hz, 1H), 7.60 (d, J = 8.5 Hz, 1H), 7.49 (dd, J = 7.1 Hz, J = 8.5 Hz, 1H), 7.09 (d, J = 7.1 Hz, 1H), 6.88 (d, J = 8.5 Hz, 1H), 6.23 (s, 1H), 5.69 (s, 2H), 4.93-4.88 (m, 1H), 3.75-3.72 (m, 2H), 3.59-3.57 (m, 2H), 3.55 (s, 3H), 3.37 (s, 3H), 3.30-3.24 (m, 6H), 2.91-2.86 (m, 1H), 2.84-2.78 (m, 1H), 2.76-2.71 (m, 1H), 2.36 (t, J = 7.3 Hz, 2H), 2.16-2.11 (m, 1H), 1.74-1.67 (m, 4H), 1.51-1.43 (m, 2H).

^{13}C NMR (101 MHz, $CDCl_3$) δ 171.5, 171.1, 169.7, 168.6, 167.7, 164.2, 162.0, 155.9, 155.1, 151.7, 147.8, 147.1, 137.0, 136.3, 134.0, 132.6, 131.5, 128.9, 126.2, 123.1, 116.8, 111.6, 110.0, 105.0, 50.7, 50.6, 49.0, 45.0, 42.4, 41.0, 40.1, 33.1, 31.6, 30.0, 29.1, 28.0, 26.8, 24.8, 23.0.

ASM127

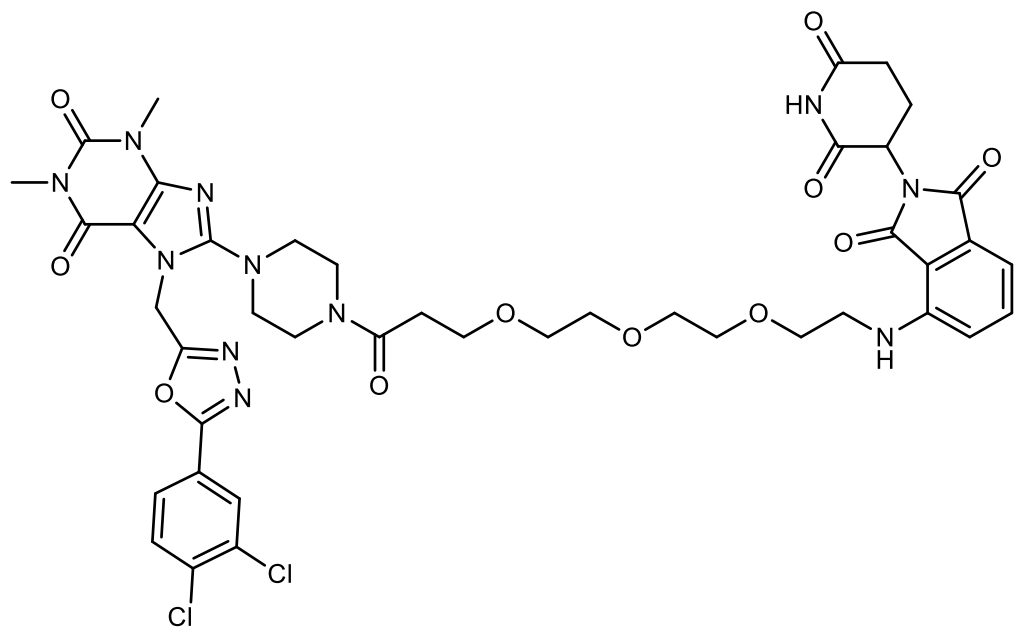

7-((5-(3,4-dichlorophenyl)-1,3,4-oxadiazol-2-yl)methyl)-8-(4-(3-(2-(2-(2-((2-(2,6-dioxopiperidin-3-yl)-1,3-dioxoisoindolin-4-yl)amino)ethoxy)ethoxy)ethoxy)propanoyl)piperazin-1-yl)-1,3-dimethyl-3,7-dihydro-1*H*-purine-2,6-dione

ASM127 was prepared according to the general procedure 2 using TH5427 (26.9 mg, 0.055 mmol, 1.1 eq), 3-(2-(2-(2-((2-(2,6-dioxopiperidin-3-yl)-1,3-dioxoisoindolin-4-yl)amino)ethoxy)ethoxy)ethoxy) propanoic acid (23.8 mg, 0.050 mmol, 1 eq), DIPEA (17 µL, 0.1 mmol, 2 eq) and HATU (37.9 mg, 0.1 mmol, 2 eq). After purification, a yellow solid was obtained (14.9 mg, 0.016 mmol, 31%).

^{1}H NMR (400 MHz, CDCl$_3$) δ 8.86 (s, 1H), 8.10 (d, J = 2Hz, 1H), 7.86 (dd, J1 = 2.0 Hz, J2 = 8.5 Hz, 1H), 7.59 (d, J = 8.5 Hz, 1H) 7.48 (dd, J1 = 7.0 Hz, J2 = 8.5 Hz, 1H) 7.09 (d, J = 7.0 Hz, 1H) 6.90 (d, J = 8.5 Hz, 1H), 5.68 (s, 2H), 4.94-4.89 (m, 1H), 3.80 (t, J = 6.5, 2H), 3.75-3.70 (m, 4H), 3.68-3.60 (m, 10H), 3.54 (s, 3H), 3.47-3.44 (m, 2H), 3.37 (s, 3H), 3.31-3.24 (m, 4H), 2.89-2.82 (m, 1H), 2.79-2.72 (m, 2H), 2.64 (t, J = 6.5, 2H), 2.17-2.10 (m, 1H). *NH-Ar not observed*

^{13}C NMR (101 MHz, CDCl$_3$) δ 171.5, 170.2, 169.4, 168.7, 167.7, 164.1, 162.1, 155.9, 155.0, 151.7, 147.8, 146.9, 136.9, 136.1, 134.0, 132.7, 131.5, 128.9, 126.2, 123.1, 116.8, 111.8, 110.5, 104.9, 71.0, 70.7, 70.6, 70.5, 69.5, 67.5, 50.6, 50.5, 49.0, 45.2, 42.5, 41.0, 40.1, 33.7, 31.6, 30.0, 28.0, 23.0.

HPLC-MS t_R = 1.56 min (96%), ESI+ m/z 951.535 $[M+H]^+$

HRMS was calculated for $C_{42}H_{46}Cl_2N_{11}O_{11}$: 950.2755, found: 950.2727

ASM130

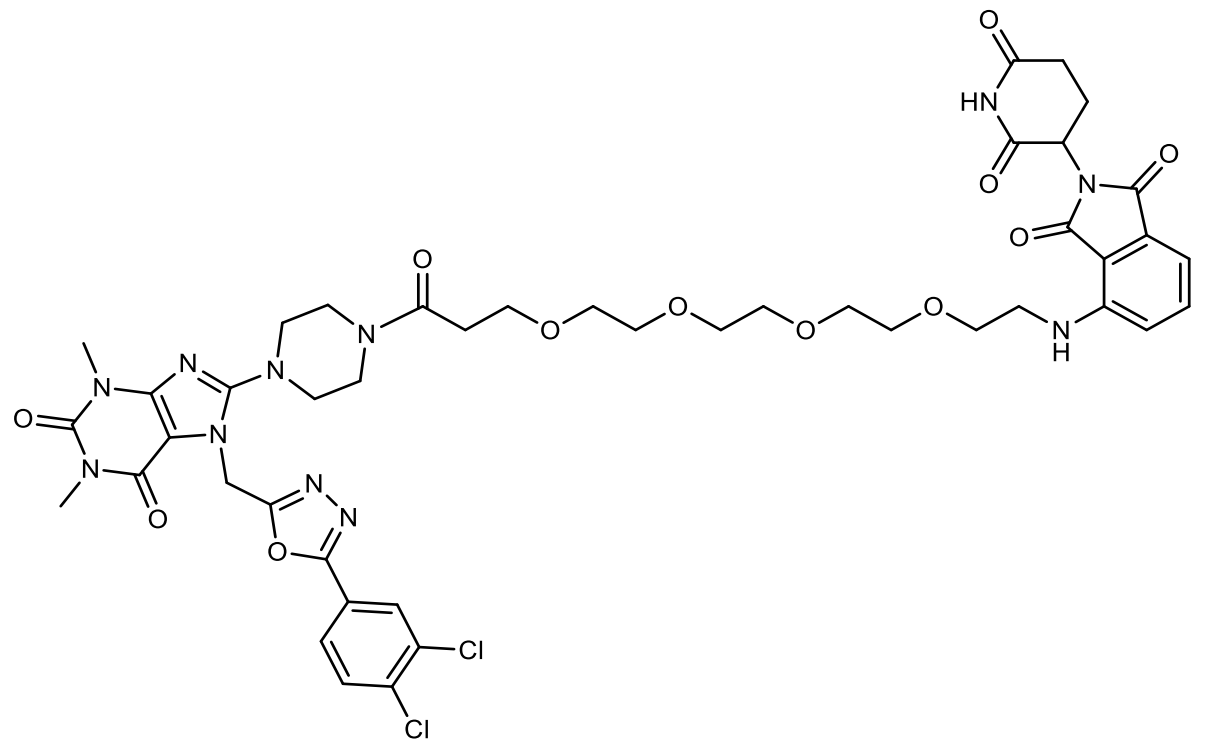

7-((5-(3,4-dichlorophenyl)-1,3,4-oxadiazol-2-yl)methyl)-8-(4-(1-((2-(2,6-dioxopiperidin-3-yl)-1,3-dioxoisoindolin-4-yl)amino)-3,6,9,12-tetraoxapentadecan-15-oyl)piperazin-1-yl)-1,3-dimethyl-3,7-dihydro-1*H*-purine-2,6-dione

ASM130 was prepared according to the general procedure 2 using TH5427 (10.3 mg, 0.021 mmol, 1.1 eq), 1-((2-(2,6-dioxopiperidin-3-yl)-1,3-dioxoisoindolin-4-yl)amino)-3,6,9,12-tetraoxa pentadecan-15-oic acid (9.9 mg, 0.019 mmol, 1 eq), DIPEA (10 µL, 0.057 mmol, 3 eq) and HATU (14.4 mg, 0.038 mmol, 2 eq). After purification, a yellow solid was obtained (4.9 mg, 0.005 mmol, 26%).

^{1}H NMR (400 MHz, $CDCl_3$) δ 8.57 (s, 1H), 8.11 (d, J = 2Hz, 1H), 7.86 (dd, J1 = 2.0 Hz, J2 = 8.5 Hz, 1H), 7.60 (d, J = 8.5 Hz, 1H) 7.49 (dd, J1 = 7.0 Hz, J2 = 8.5 Hz, 1H) 7.10 (d, J = 7.0 Hz, 1H) 6.92 (d, J = 8.5 Hz, 1H), 5.69 (s, 2H), 4.93-4.89 (m, 1H), 3.81 (t, J = 6.5, 2H), 3.74-3.71 (m, 4H), 3.68-3.64 (m, 8H), 3.64-3.61 (m, 6H), 3.56 (s, 3H), 3.47 (t, J = 5.5 Hz, 2H), 3.38 (s, 3H), 3.30-3.27 (m, 4H), 2.90-2.84 (m, 1H), 2.81-2.72 (m, 2H), 2.65 (t, J = 6.5, 2H), 2.16-2.10 (m, 1H). *NH-Ar not observed*

^{13}C NMR (101 MHz, $CDCl_3$) δ 171.3, 170.0, 169.4, 168.6, 167.7, 164.1, 162.1, 155.9, 155.0, 151.7, 147.8, 146.9, 136.9, 136.2, 134.0, 132.7, 131.5, 128.9, 126.2, 123.1, 116.9, 111.8, 110.5, 105.0, 70.9, 70.8, 70.7, 70.6, 70.6, 70.5, 69.6, 67.5, 50.7, 50.5, 49.0, 45.2, 42.5, 41.0, 40.1, 33.7, 31.6, 30.0, 28.0, 23.0.

HPLC-MS t_R = 1.55 min (97.8%), ESI+ m/z 995.315; 996.395 $[M+H]^+$

HRMS was calculated for $C_{44}H_{50}Cl_2N_{11}O_{12}$: 994.3018, found: 994.3007

ASM131

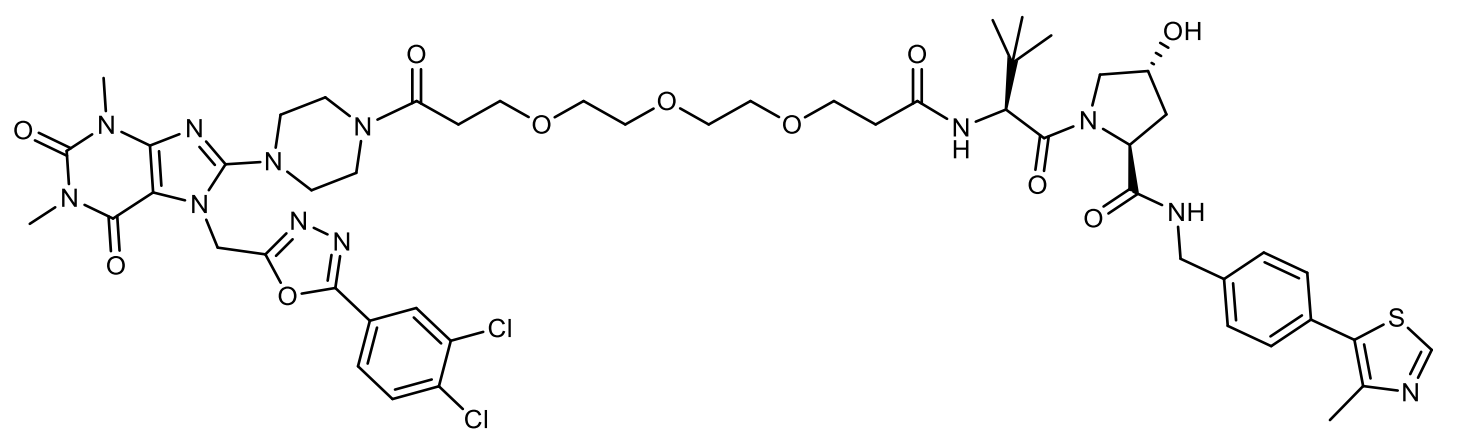

(2*S*,4*R*)-1-((*S*)-2-(*tert*-butyl)-16-(4-(7-((5-(3,4-dichlorophenyl)-1,3,4-oxadiazol-2-yl)methyl)-1,3-dimethyl-2,6-dioxo-2,3,6,7-tetrahydro-1*H*-purin-8-yl)piperazin-1-yl)-4,16-dioxo-7,10,13-trioxa-3-azahexadecanoyl)-4-hydroxy-N-(4-(4-methylthiazol-5-yl)benzyl)pyrrolidine-2-carboxamide

ASM131 was prepared according to the general procedure 2 using TH5427 (10.8 mg, 0.022 mmol, 1.1 eq), (*S*)-15-((2*S*,4*R*)-4-hydroxy-2-((4-(4-methylthiazol-5-yl)benzyl)carbamoyl)pyrrolidine-1-carbonyl)-16,16-dimethyl-13-oxo-4,7,10-trioxa-14-azaheptadecanoic acid (13.3 mg, 0.020 mmol, 1 eq), DIPEA (10 µL, 0.057 mmol, 3 eq) and HATU (15.2 mg, 0.040 mmol, 2 eq). After purification, a yellow solid was obtained (5.2 mg, 0.005 mmol, 24%).

^{1}H NMR (400 MHz, $CDCl_3$) δ 8.87 (s, 1H), 8.11 (d, J = 2 Hz, 1H), 7.86 (dd, J1 = 2.0 Hz, J2 = 8.5 Hz, 1H), 7.60 (d, J = 8.5 Hz, 1H), 7.50-7.47 (m, 1H), 7.40-7.35 (m, 5H), 7.02 (d, J = 8.4 Hz, 1H), 5.70 (s, 2H), 4.72 (t, J = 8.1 Hz, 1H), 4.62-4.57 (m, 1H), 4.53-4.50 (m, 1H), 4.48 (d, J = 8.5 Hz, 1H), 4.37-4.32 (m, 1H), 4.13 (bd, J = 11.6 Hz, 1H), 3.76 (t, J = 6.6 Hz, 2H), 3.74-3.70 (m, 4H), 3.64-3.61 (m, 6H), 3.60-3.58 (m, 5H), 3.55 (s, 3H), 3.37 (s, 3H), 3.32-3.29 (m, 2H), 3.27-3.24 (m, 2H), 2.66-2.62 (m, 2H), 2.57 (s, 3H), 2.53-2.45 (m, 4H), 2.19-2.13 (m, 1H).

^{13}C NMR (101 MHz, $CDCl_3$) δ 172.2, 171.9, 171.2, 170.0, 164.2, 162.1, 155.9, 155.0, 151.7, 151.2, 147.8, 145.9, 139.6, 137.0, 134.0, 131.6, 129.5 (2C), 128.9, 128.5 (2C), 126.2, 123.1, 105.0, 70.6, 70.6, 70.5, 70.5, 70.3, 67.4, 67.3, 58.6, 57.9, 56.9, 50.7, 50.5, 45.2, 43.2, 41.0, 40.2, 36.8, 36.3, 35.0, 33.7, 30.0, 28.0, 26.6 (3C), 15.0.

HPLC-MS t_R = 1.55 min (97.8%), ESI+ m/z 995.315 $[M+H]^+$

HRMS was calculated for $C_{52}H_{65}Cl_2N_{12}O_{11}S$: 1135.3994, found: 1135.3998

ASM133

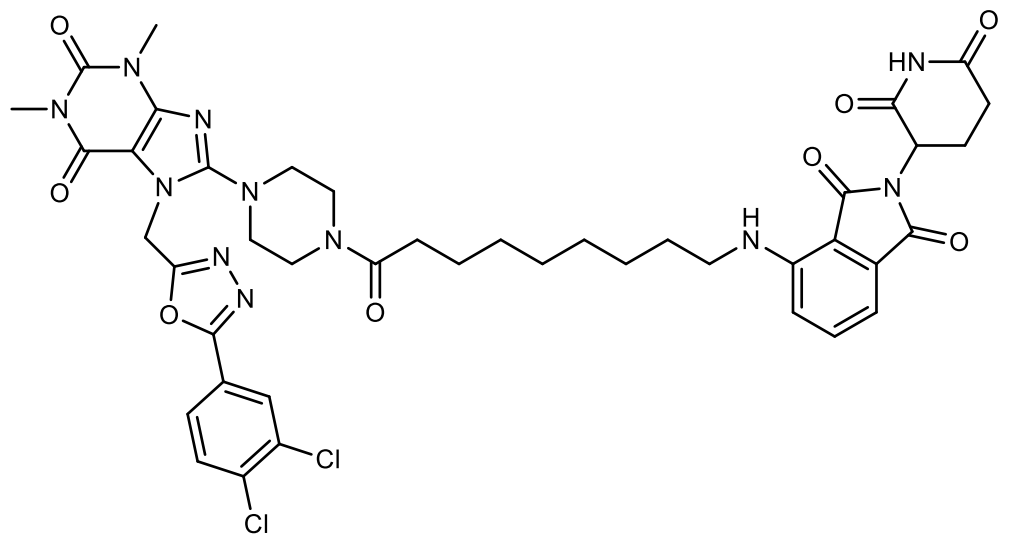

7-((5-(3,4-dichlorophenyl)-1,3,4-oxadiazol-2-yl)methyl)-8-(4-(9-((2-(2,6-dioxopiperidin-3-yl)-1,3-dioxoisoindolin-4-yl)amino)nonanoyl)piperazin-1-yl)-1,3-dimethyl-3,7-dihydro-1*H*-purine-2,6-dione

ASM133 was prepared according to the general procedure 2 using TH5427 (15.1 mg, 0.031 mmol, 1.1 eq), 9-((2-(2,6-dioxopiperidin-3-yl)-1,3-dioxoisoindolin-4-yl)amino)nonanoic acid (12.0 mg, 0.028 mmol, 1 eq), DIPEA (15 µL, 0.084 mmol, 3 eq) and HATU (21.2 mg, 0.056 mmol, 2 eq). After purification, a yellow solid was obtained (2.4 mg, 0.003 mmol, 10%).

^{1}H NMR (400 MHz, $CDCl_3$) δ 8.14 (s, 1H), 8.11 (d, J = 2.0 Hz, 1H), 7.86 (dd, J1 = 2.0 Hz, J2 = 8.5 Hz, 1H), 7.60 (d, J = 8.3 Hz, 1H), 7.51-7.47 (m, 1H), 7.08 (d, J = 7.0 Hz, 1H), 6.88 (d, J = 8.5 Hz, 1H), 5.69 (s, 2H), 4.93-4.89 (m, 1H), 3.71-3.62 (m, 4H), 3.55 (s, 3H), 3.37 (s, 3H), 3.29-3.24 (m, 6H), 2.91-2.80 (m, 2H), 2.78-2.68 (m, 2H), 2.33 (t, J = 7.3 Hz, 2H), 2.16-2.09 (m, 1H), 1.68-1.64 (m, 12H).

HPLC-MS t_R = 1.79 min (97.7%), ESI+ m/z 903.250 $[M+H]^+$

ASM134

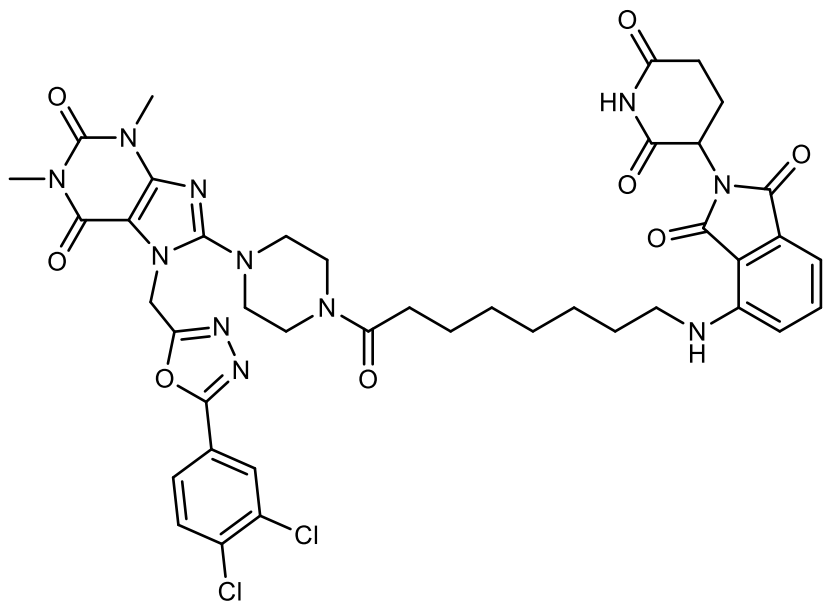

7-((5-(3,4-dichlorophenyl)-1,3,4-oxadiazol-2-yl)methyl)-8-(4-(8-((2-(2,6-dioxopiperidin-3-yl)-1,3-dioxoisoindolin-4-yl)amino)octanoyl)piperazin-1-yl)-1,3-dimethyl-3,7-dihydro-1*H*-purine-2,6-dione

ASM134 was prepared according to the general procedure 2 using TH5427 (15.0 mg, 0.030 mmol, 1.1 eq), 8-((2-(2,6-dioxopiperidin-3-yl)-1,3-dioxoisoindolin-4-yl)amino)octanoic acid (11.5 mg, 0.028 mmol, 1 eq), DIPEA (14 µL, 0.083 mmol, 3 eq) and HATU (21.1 mg, 0.055 mmol, 2 eq). After purification, a yellow solid was obtained (4.8 mg, 0.005 mmol, 19%).

^{1}H NMR (400 MHz, CDCl$_3$) δ 8.15 (s, 1H), 8.11 (d, J = 2.0 Hz, 1H), 7.86 (dd, J = 2.0 Hz, J = 8.4 Hz, 1H), 7.60 (d, J = 8.4 Hz, 1H), 7.49 (dd, J = 7.2 Hz, J = 8.4 Hz, 1H), 7.08 (d, J = 7.2 Hz, 1H), 6.88 (d, J = 8.6 Hz, 1H), 5.69 (s, 2H), 4.93-4.88 (m, 1H), 3.72-3.62 (m, 4H), 3.55 (s, 3H), 3.37 (s, 3H), 3.29- 3.24 (m, 6H), 2.91-2.80 (m, 2H), 2.78-2.68 (m, 2H), 2.33 (t, J = 7.4 Hz, 2H), 2.16-2.11 (m, 1H), 1.68-1.65 (m, 4H), 1.41-1.37 (m, 6H).

^{13}C NMR (101 MHz, CDCl$_3$) δ 171.9, 171.0, 169.7, 168.5, 167.7, 164.2, 162.0, 155.9, 155.1, 151.7, 147.8, 147.1, 137.0, 136.3, 134.0, 132.6, 131.6, 128.9, 126.2, 123.1, 116.8, 111.6, 110.0, 105.0, 50.7 (2C), 49.0, 45.0, 42.8, 40.9, 40.1, 33.3, 31.6, 30.0, 29.4, 29.3, 29.2, 28.0, 26.9, 25.2, 23.0.

HPLC-MS t_R = 1.74 min (97.8%), ESI+ m/z 888.726; 890.467 $[M+H]^+$

HRMS was calculated for $C_{41}H_{44}Cl_2N_{11}O_8$: 888.2751, found: 888.2767

ASM135

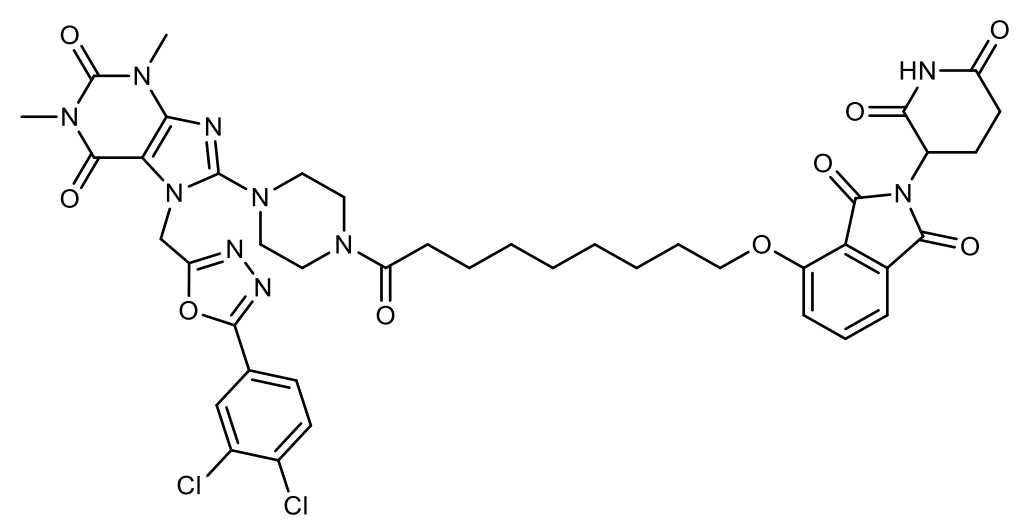

7-((5-(3,4-dichlorophenyl)-1,3,4-oxadiazol-2-yl)methyl)-8-(4-(9-((2-(2,6-dioxopiperidin-3-yl)-1,3-dioxoisoindolin-4-yl)oxy)nonanoyl)piperazin-1-yl)-1,3-dimethyl-3,7-dihydro-1*H*-purine-2,6-dione

ASM135 was prepared according to the general procedure 2 using TH5427 (15.1 mg, 0.031 mmol, 1.1 eq), 9-((2-(2,6-dioxopiperidin-3-yl)-1,3-dioxoisoindolin-4-yl)oxy)nonanoic acid (12 mg, 0.028 mmol, 1 eq), DIPEA (15 µL, 0.084 mmol, 3 eq) and HATU (21.2 mg, 0.056 mmol, 2 eq). After purification, a white solid was obtained (6.1 mg, 0.007 mmol, 24%).

^{1}H NMR (400 MHz, $CDCl_3$) δ 8.33 (s, 1H), 8.10 (d, J = 2Hz, 1H), 7.86 (dd, J1 = 2.0 Hz, J2 = 8.5 Hz, 1H), 7.66 (dd, J1 = 7.3 Hz, J2 = 8.5 Hz, 1H), 7.60 (d, J = 8.5 Hz, 1H), 7.44 (bd, J = 7.4 Hz, 1H) 7.20 (d, J = 8.4 Hz, 1H) 5.69 (s, 2H), 4.97-4.92 (m, 1H), 4.19-4.14 (m, 2H), 3.72 (bs, 2H), 3.59 (bs, 2H), 3.55 (s, 3H), 3.37 (s, 3H), 3.29-3.19 (m, 4H), 2.91-2.77 (m, 2H), 2.76-2.67 (m, 1H), 2.33 (t, J = 7.3 Hz, 2H), 2.16-2.10 (m, 1H), 1.90-1.83 (m, 2H), 1.74 (bs, 2H), 1.64-1.61 (m, 2H), 1.53-1.47 (m, 2H), 1.39-1.36 (m, 4H).

^{13}C NMR (101 MHz, $CDCl_3$) δ 172.1, 171.1, 168.3, 167.2, 165.8, 164.2, 162.0, 156.9, 155.9, 155.1, 151.7, 147.8, 137.0, 136.6, 134.0, 134.0, 131.5, 128.9, 126.2, 123.1, 119.1, 117.3, 115.9, 105.0, 69.6, 50.7 (2C), 49.2, 45.1, 40.9, 40.1, 33.4, 31.6, 30.0, 29.4, 29.3, 29.1, 28.9, 28.0, 25.9, 25.3, 22.8.

HPLC-MS t_R = 1.73 min (98.2%), ESI+ m/z 903.730; 905.531 $[M+H]^+$

ASM136

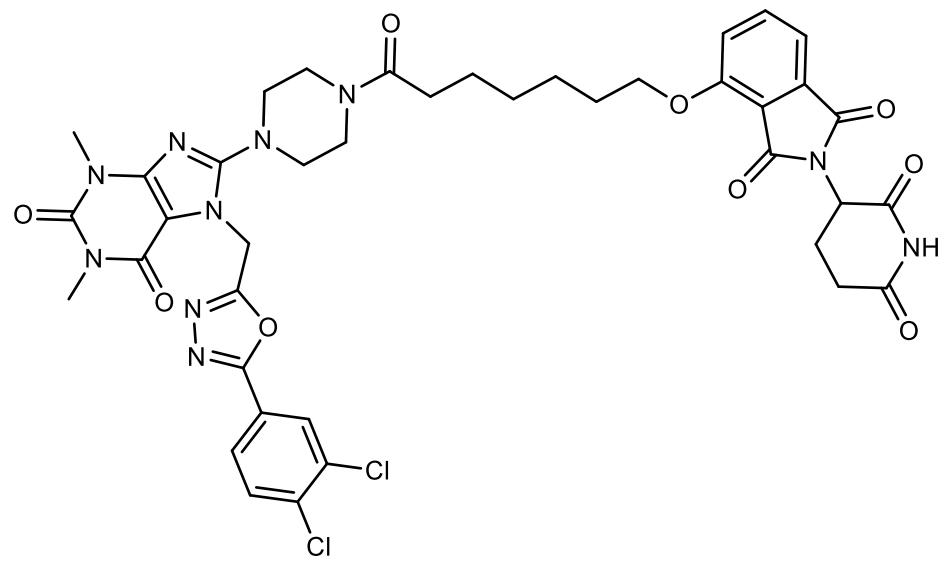

7-((5-(3,4-dichlorophenyl)-1,3,4-oxadiazol-2-yl)methyl)-8-(4-(7-((2-(2,6-dioxopiperidin-3-yl)-1,3-dioxoisoindolin-4-yl)oxy)heptanoyl)piperazin-1-yl)-1,3-dimethyl-3,7-dihydro-1*H*-purine-2,6-dione

ASM136 was prepared according to the general procedure 2 using TH5427 (15.0 mg, 0.030 mmol, 1.1 eq), 7-((2-(2,6-dioxopiperidin-3-yl)-1,3-dioxoisoindolin-4-yl)oxy)heptanoic acid (11.5 mg, 0.028 mmol, 1 eq), DIPEA (14 µL, 0.083 mmol, 3 eq) and HATU (21.1 mg, 0.055 mmol, 2 eq). After purification, a yellow solid was obtained (4.8 mg, 0.005 mmol, 19%).

^{1}H NMR (400 MHz, CDCl$_3$) δ 8.64 (s, 1H), 8.10 (d, J = 2Hz, 1H), 7.85 (dd, J1 = 2.0 Hz, J2 = 8.5 Hz, 1H), 7.67 (dd, J1 = 7.3 Hz, J2 = 8.5 Hz, 1H), 7.59 (d, J = 8.5 Hz, 1H), 7.45 (bd, J = 7.4 Hz, 1H), 7.21 (d, J = 8.5 Hz, 1H), 5.78-5.68 (m, 2H), 4.97-4.92 (m, 1H), 4.17 (t, J = 6.2 Hz, 1H), 3.72-3.62 (m, 4H), 3.62 (s, 3H), 3.38 (s, 3H), 3.29-3.25 (m, 4H), 2.91-2.70 (m, 3H), 2.46-2.30 (m, 2H), 2.17-2.10 (m, 1H), 1.91-1.84 (m, 2H), 1.71-1.66 (m, 4H), 1.61-1.54 (m, 2H), 1.50-1.45 (m, 2H).

^{13}C NMR (101 MHz, CDCl$_3$) δ 172.0, 171.1, 168.5, 167.2, 165.9, 164.2, 162.1, 156.8, 155.9, 155.1, 151.7, 147.7, 137.0, 136.7, 134.0, 133.9, 131.5, 128.9, 126.2, 123.0, 119.0, 117.3, 115.9, 105.0, 69.4, 50.8, 50.5, 49.2, 45.0, 40.9, 40.0, 32.8, 31.6, 30.0, 28.8, 28.7, 28.0, 25.6, 25.0, 22.8.

HPLC-MS t_R = 1.63 min (98.0%), ESI+ m/z; 875.643, 877.563 $[M+H]^+$

HRMS was calculated for $C_{40}H_{41}Cl_2N_{10}O_9$: 875.2435, found: 875.2448

ASM137

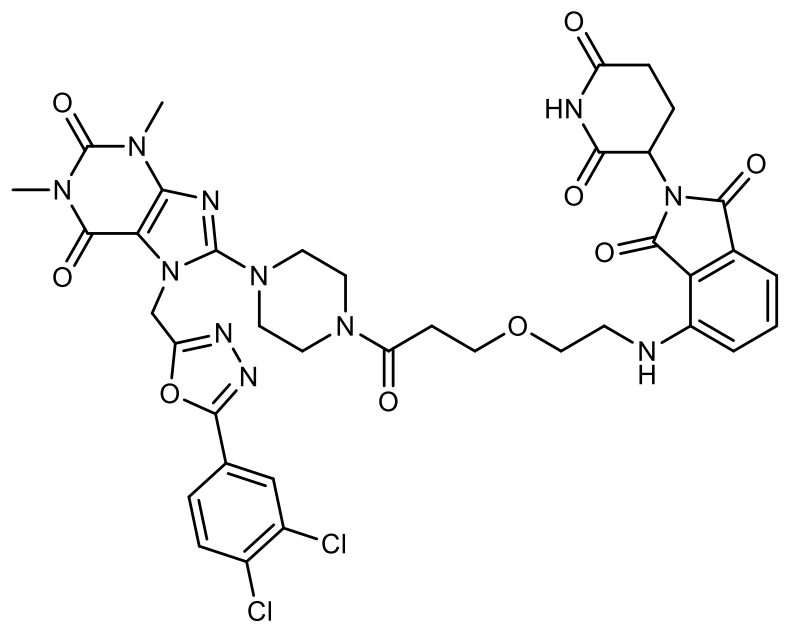

7-((5-(3,4-dichlorophenyl)-1,3,4-oxadiazol-2-yl)methyl)-8-(4-(3-(2-((2-(2,6-dioxopiperidin-3-yl)-1,3-dioxoisoindolin-4-yl)amino)ethoxy)propanoyl)piperazin-1-yl)-1,3-dimethyl-3,7-dihydro-1*H*-purine-2,6-dione

ASM137 was prepared according to the general procedure 2 using TH5427 (15.3 mg, 0.031 mmol, 1.1 eq), 3-(2-((2-(2,6-dioxopiperidin-3-yl)-1,3-dioxoisoindolin-4-yl)amino)ethoxy)propanoic acid (11.0 mg, 0.028 mmol, 1 eq), DIPEA (15 µL, 0.085 mmol, 3 eq) and HATU (21.5 mg, 0.057 mmol, 2 eq). After purification, a yellow solid was obtained (11.4 mg, 0.005 mmol, 47%).

^{1}H NMR (400 MHz, $CDCl_3$) δ 9.00 (s, 1H), 8.09 (d, J = 2Hz, 1H), 7.84 (dd, J1 = 2.0 Hz, J2 = 8.5 Hz, 1H), 7.58 (d, J = 8.5 Hz, 1H), 7.48 (dd, J1 = 7.1 Hz, J2 = 8.5 Hz, 1H), 7.10 (bd, J = 7.1Hz, 1H), 6.88 (d, J = 8.5 Hz, 1H), 5.79- 5.70 (m, 2H), 4.93-4.88 (m, 1H), 3.84 (t, J = 6.2 Hz, 2H), 3.78-3.60 (m, 6H), 3.53 (s, 3H), 3.45-3.43 (m, 2H), 3.38 (s, 3H), 3.28-3.22 (m, 4H), 2.88-2.85 (m, 1H), 2.80-2.71 (m, 2H), 2.70-2.59 (m, 2H), 2.16-2.11 (m, 1H). *NH-Ar not observed*

^{13}C NMR (101 MHz, $CDCl_3$) δ 171.4, 169.8, 169.5, 168.9, 167.6, 164.2, 162.1, 155.9, 155.1, 151.7, 147.7, 146.9, 137.0, 136.2, 134.0, 132.7, 131.5, 128.9, 126.2, 123.0, 116.9, 111.9, 110.5, 105.0, 69.1, 67.4, 50.6, 50.3, 49.0, 45.3, 42.2, 41.1, 40.0, 33.6, 31.5, 30.0, 28.0, 23.1.

HPLC-MS t_R = 1.55 min (97.4%), ESI+ m/z; 862.739, 864.600 $[M+H]^+$

HRMS was calculated for $C_{38}H_{38}Cl_2N_{11}O_9$: 862.2231, found: 862.2251

Procedure for the synthesis of ASM124

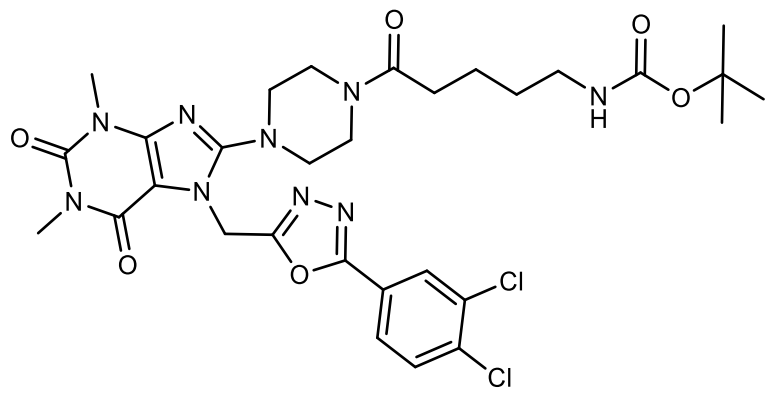

Tert-butyl (5-(4-(7-((5-(3,4-dichlorophenyl)-1,3,4-oxadiazol-2-yl)methyl)-1,3-dimethyl-2,6-dioxo-2,3,6,7-tetrahydro-1*H*-purin-8-yl)piperazin-1-yl)-5-oxopentyl)carbamate

Tert-butyl (5-(4-(7-((5-(3,4-dichlorophenyl)-1,3,4-oxadiazol-2-yl)methyl)-1,3-dimethyl-2,6-dioxo-2,3,6,7-tetrahydro-1*H*-purin-8-yl)piperazin-1-yl)-5-oxopentyl)carbamate was prepared from TH5427.TFA (75 mg, 0.124 mmol, 1.1 eq), 5-boc-amino-pentanoic acid (25 mg, 0.115 mmol, 1 eq), HATU (87.5 mg, 0.230 mmol, 2 eq) and DIPEA (60 µL, 0.345 mmol, 3 eq). Under nitrogen, DIPEA (2 eq) and HATU were added to a solution of acid in DMF (2.3 mL) at 0°C. After stirring for 15 min at 0°C, a solution of amine and DIPEA (1 eq) in DMF (2.3 mL) was added dropwise. The mixture was stirred overnight at room temperature. The crude was purified by flash column chromatography (Si-35, DCM/MeOH, gradient from 100/0 to 95/5) to afford the expected product as brown solid (19.1 mg, 0.028 mmol, 24%).

^{1}H NMR (400 MHz, $CDCl_3$) δ 8.10 (d, J = 1.9 Hz, 1H), 7.85 (dd, J = 1.9 Hz, J = 8.6 Hz, 1H), 7.59 (d, J = 8.6 Hz, 1H) 5.68 (s, 2H), 4.62 (s, 2H), 3.72 (bs, 2H), 3.58 (bs, 2H), 3.54 (s, 3H), 3.36 (s, 3H), 3.28-3.25 (m, 4H), 3.13-3.12 (m, 2H), 2.35 (t, J = 7.1 Hz, 2H), 1.71-1.63 (m, 2H), 1.56-1.49 (m, 2H), 1.42 (s, 9H).

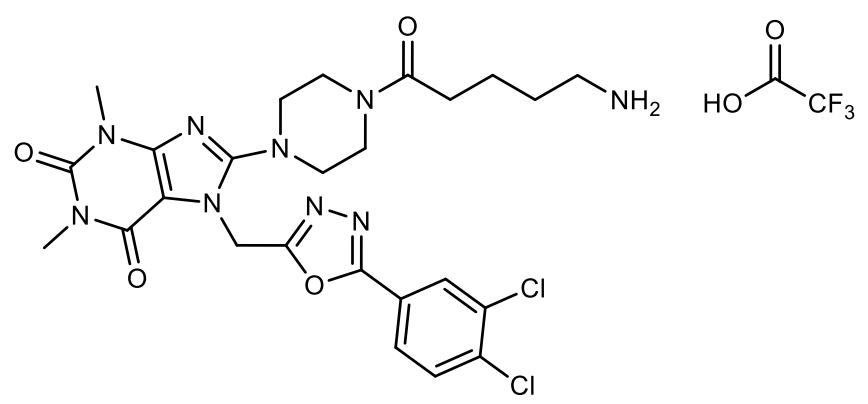

5-(4-(7-((5-(3,4-dichlorophenyl)-1,3,4-oxadiazol-2-yl)methyl)-1,3-dimethyl-2,6-dioxo-2,3,6,7-tetrahydro-1H-purin-8-yl)piperazin-1-yl)-5-oxopentan-1-aminium trifluoroacetate

Tert-butyl (5-(4-(7-((5-(3,4-dichlorophenyl)-1,3,4-oxadiazol-2-yl)methyl)-1,3-dimethyl-2,6-dioxo-2,3,6,7-tetrahydro-1*H*-purin-8-yl)piperazin-1-yl)-5-oxopentyl)carbamate (29 mg, 0.042 mmol, 1 eq) was dissolved in DCM (1.8 mL) then TFA (97 µL) was added at 0C. The mixture was stirred overnight at room temperature then water was added and the aqueous layer was recovered and evaporated under reduced pressure to afford the expected product as a pale white solid (29.6 mg, 0.042 mmol, quantitative).

^{1}H NMR (400 MHz, $CDCl_3$) δ 8.18 (d, J = 2.0 Hz, 1H), 7.95 (dd, J = 2.0 Hz, J = 8.5 Hz, 1H), 7.76 (d, J = 8.5 Hz, 1H) 5.78 (s, 2H), 3.76-3.71 (m, 4H), 3.53 (s, 3H), 3.40-3.37 (m, 2H), 3.34-3.32 (m, 2H), 3.28 (s, 3H), 3.00-2.96 (m, 2H), 2.52 (t, J = 6.6 Hz, 2H), 1.73-1.70 (m, 4H).

ASM124

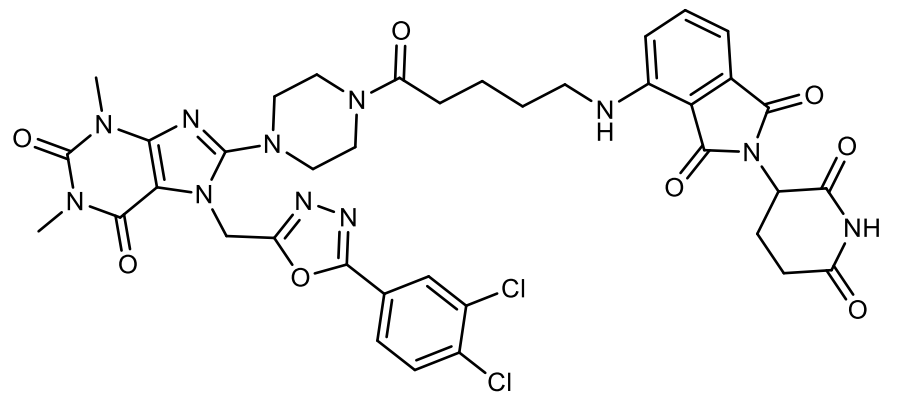

7-((5-(3,4-dichlorophenyl)-1,3,4-oxadiazol-2-yl)methyl)-8-(4-(5-((2-(2,6-dioxopiperidin-3-yl)-1,3-dioxoisoindolin-4-yl)amino)pentanoyl)piperazin-1-yl)-1,3-dimethyl-3,7-dihydro-1*H*-purine-2,6-dione

ASM124 was prepared from 5-(4-(7-((5-(3,4-dichlorophenyl)-1,3,4-oxadiazol-2-yl)methyl)-1,3-dimethyl-2,6-dioxo-2,3,6,7-tetrahydro-1H-purin-8-yl)piperazin-1-yl)-5-oxopentan-1-aminium trifluoroacetate (29.6 mg, 0.042 mmol, 1eq), 4-fluoro thalidomide (11.6 mg, 0.042 mmol, 1 eq) and triethylamine (18 µL, 0.126 mmol, 3eq). A microwave vial was charged with the reagents and DMSO (1 mL) then heated to 200°C for 15 min. The resulting mixture was then purified by prep HPLC to afford the expected product as yellow solid (1.6 mg, 0.002 mmol, 4%).

^{1}H NMR (400 MHz, $CDCl_3$) δ 8.34 (s, 1H), 8.10 (d, J = 2Hz, 1H), 7.86 (dd, J1 = 2.0 Hz, J2 = 8.4 Hz, 1H), 7.60 (d, J = 8.4 Hz, 1H), 7.50 (dd, J1 = 7.1 Hz, J2 = 8.4 Hz, 1H), 7.10 (d, J = 7.1 Hz, 1H), 6.89 (d, J = 8.4 Hz, 1H), 5.70 (s, 2H), 4.92-4.88 (m, 1H), 3.73-3.69 (m, 2H), 3.63-3.57 (m, 2H), 3.55 (s, 3H), 3.38 (s, 3H), 3.34-3.31 (m, 2H), 3.29-3.24 (m, 4H), 2.90-2.84 (m, 1H), 2.80-2.71 (m, 2H), 2.41 (t, J = 6.8 Hz, 2H), 2.16-2.09 (m, 1H), 1.81-1.73 (m, 4H). *NH not observed*

HPLC-MS t_R = 1.59 min (97.7%), ESI+ m/z 848.463 $[M+H]^+$

Procedure for the synthesis of ASM102

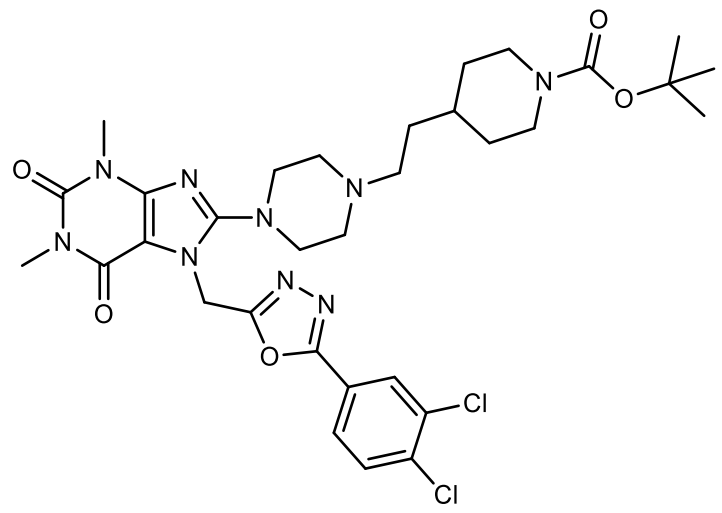

Tert-butyl 4-(2-(4-(7-((5-(3,4-dichlorophenyl)-1,3,4-oxadiazol-2-yl)methyl)-1,3-dimethyl-2,6-dioxo-2,3,6,7-tetrahydro-1*H*-purin-8-yl)piperazin-1-yl)ethyl)piperidine-1-carboxylate

Tert-butyl 4-(2-(4-(7-((5-(3,4-dichlorophenyl)-1,3,4-oxadiazol-2-yl)methyl)-1,3-dimethyl-2,6-dioxo-2,3,6,7-tetrahydro-1*H*-purin-8-yl)piperazin-1-yl)ethyl)piperidine-1-carboxylate was prepared according to the general procedure 3 using TH5427.TFA (33 mg, 0.055 mmol, 1 eq), *N*-Boc-4-piperidineacetaldehyde (14.9 mg, 0.065 mmol, 1.2 eq), sodium triacetoxyborohydride (17.3 mg, 0.082 mmol, 1.5 eq) and THF (1 mL). After purification, a light brown solid was obtained (33.1 mg, 0.047 mmol, 86%).

^{1}H NMR (400 MHz, $CDCl_3$) δ 8.08 (d, J = 2.0 Hz, 1H), 7.83 (dd, J = 2.0 Hz, J = 8.4 Hz, 1H), 7.57 (d, J = 8.4 Hz, 1H), 5.62 (s, 2H), 4.05 (bs, 3H), 3.53 (s, 3H), 3.34 (s, 3H), 3.33-3.32 (m, 4H), 2.68-2.61 (m, 3H) 2.58-2.55 (bs, 4H), 2.44-2.41 (m, 2H), 1.66-1.59 (m, 3H), 1.43 (s, 9H), 1.15-1.04 (m, 3H).

^{13}C NMR (101 MHz, $CDCl_3$) δ 164.0, 162.2, 156.5, 155.0, 154.9, 151.7, 148.0, 136.8, 133.9, 131.5, 128.9, 126.1, 123.2, 104.8, 79.4, 60.3, 55.9, 52.3 (2C), 50.1 (2C), 40.3 (2C), 39.4, 34.4, 32.6, 32.3 (2C), 30.0, 28.6 (3C), 27.9.

HPLC-MS t_R = 1.86 min (94.5%), ESI+ m/z 702.824; 705.992 $[M+H]^+$

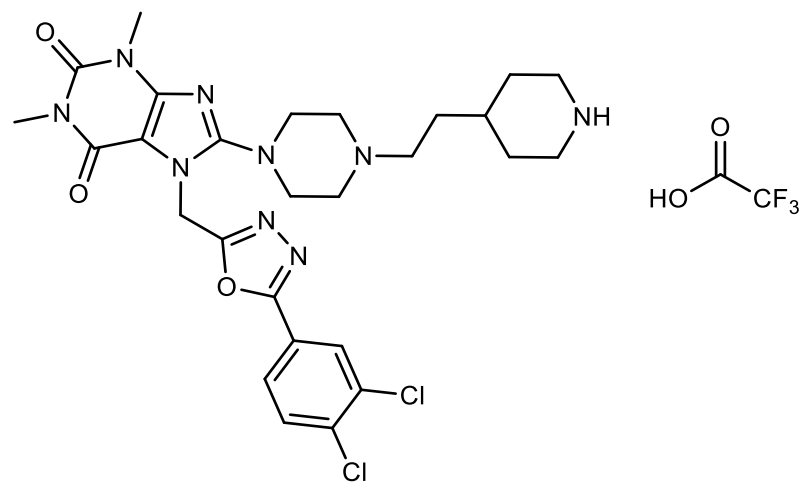

4-(2-(4-(7-((5-(3,4-dichlorophenyl)-1,3,4-oxadiazol-2-yl)methyl)-1,3-dimethyl-2,6-dioxo-2,3,6,7-tetrahydro-1*H*-purin-8-yl)piperazin-1-yl)ethyl)piperidin-1-ium trifluoroacetate

4-(2-(4-(7-((5-(3,4-dichlorophenyl)-1,3,4-oxadiazol-2-yl)methyl)-1,3-dimethyl-2,6-dioxo-2,3,6,7-tetrahydro-1*H*-purin-8-yl)piperazin-1-yl)ethyl)piperidin-1-ium trifluoroacetate was prepared from *tert*-butyl 4-((4-(7-((5-(3,4-dichlorophenyl)-1,3,4-oxadiazol-2-yl)methyl)-1,3-dimethyl-2,6-dioxo-2,3,6,7-tetrahydro-1*H*-purin-8-yl)piperazin-1-yl)ethyl)piperidine-1-carboxylate (33 mg, 0.047 mmol, 1eq), TFA (109 µL, 1.410 mmol, 30 eq) and DCM (2.8 mL) using the general procedure 4. The crude was used directly in the next step without further purification.

^{1}H NMR (400 MHz, MeOD) δ 8.15-8.14 (d, J = 2.0 Hz, 1H), 7.92 (dd, J = 2.0 Hz, J = 8.4 Hz, 1H), 7.73 (d, J = 8.4 Hz, 1H), 5.78 (s, 2H), 3.79-3.77 (m, 3H), 3.51 (s, 3H), 3.43-3.36 (m, 8H), 3.30 (s, 3H), 3.03-2.98 (m, 4H), 2.02-2.01 (m, 2H), 1.85-1.80 (m, 2H), 1.55-1.51 (m, 2H) *(NH.TFA not observed).*

ASM102

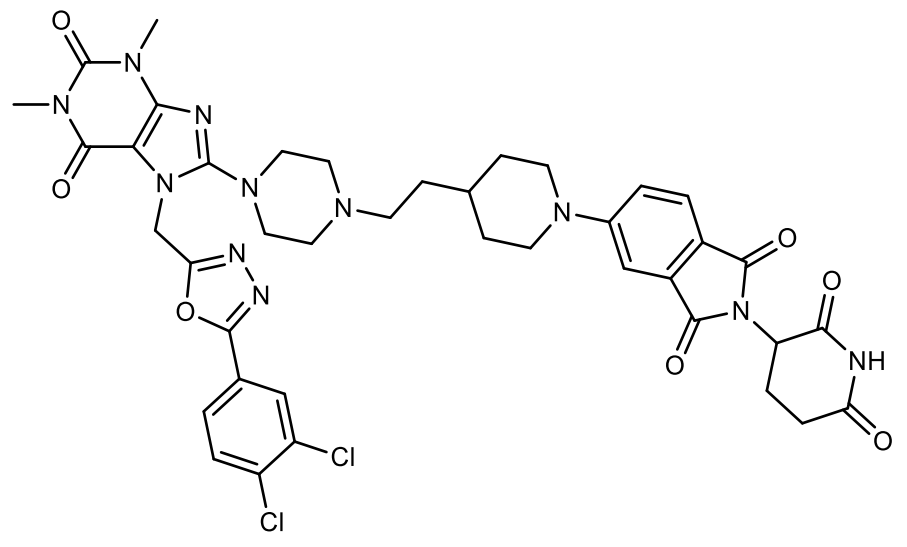

7-((5-(3,4-dichlorophenyl)-1,3,4-oxadiazol-2-yl)methyl)-8-(4-(2-(1-(2-(2,6-dioxopiperidin-3-yl)-1,3-dioxoisoindolin-5-yl)piperidin-4-yl)ethyl)piperazin-1-yl)-1,3-dimethyl-3,7-dihydro-1*H*-purine-2,6-dione

ASM102 was prepared according to the general procedure 5 using 4-(2-(4-(7-((5-(3,4-dichlorophenyl)-1,3,4-oxadiazol-2-yl)methyl)-1,3-dimethyl-2,6-dioxo-2,3,6,7-tetrahydro-1*H*-purin-8-yl)piperazin-1-yl)ethyl)piperidin-1-ium trifluoroacetate (33 mg, 0.046 mmol, 1 eq), 5-fluoro thalidomide (12.7 mg, 0.046 mmol, 1eq), TRIETHYLAMINE (19 µL, 0.138 mmol, 3 eq) and DMSO (1.2 mL). After purification, a yellow solid was obtained (1.7 mg, 0.002 mmol, 4%).

^{1}H NMR (400 MHz, CDCl$_3$) δ 8.12 (d, J = 2.0 Hz, 1H), 8.00 (bs, 1H), 7.86 (dd, J = 2.0 Hz, J = 8.5 Hz, 1H), 7.67 (d, J = 8.4 Hz, 1H) 7.61 (d, J = 8.4 Hz), 7.05- 7.02 (m, 1H), 5.65 (s, 2H), 4.96-4.91 (m, 1H), 3.96-3.93 (m, 2H), 3.55 (s, 3H), 3.37 (s, 3H), 3.00-2.92 (m, 4H), 2.88-2.70 (m, 5H), 2.16-2.12 (m, 2H), 1.85-1.82 (m, 3H), 1.62-1.58 (m, 9H).

HRMS was calculated for $C_{40}H_{42}Cl_2N_{11}O_7$: 858.2646, found: 858.2644

Procedure for the synthesis of ASM105

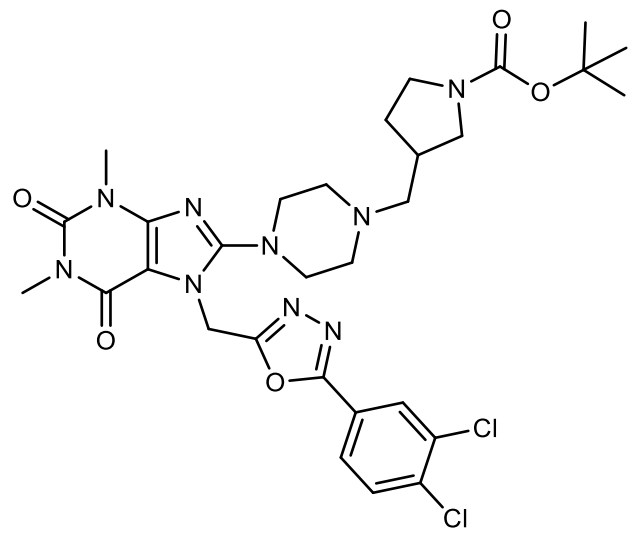

Tert-butyl 3-((4-(7-((5-(3,4-dichlorophenyl)-1,3,4-oxadiazol-2-yl)methyl)-1,3-dimethyl-2,6-dioxo-2,3,6,7-tetrahydro-1*H*-purin-8-yl)piperazin-1-yl)methyl)pyrrolidine-1-carboxylate

Tert-butyl 3-((4-(7-((5-(3,4-dichlorophenyl)-1,3,4-oxadiazol-2-yl)methyl)-1,3-dimethyl-2,6-dioxo-2,3,6,7-tetrahydro-1*H*-purin-8-yl)piperazin-1-yl)methyl)pyrrolidine-1-carboxylate was prepared according to the general procedure 3 using TH5427.TFA (50 mg, 0.083 mmol, 1 eq), *tert*-butyl 3-formylpyrrolidine-1-carboxylate (19.7 mg, 0.099 mmol, 1.2 eq), sodium triacetoxyborohydride (26.3 mg, 0.124 mmol, 1.5 eq) and THF (1.5 mL). After purification, an off-white solid was obtained (39.8 mg, 0.059 mmol, 71%).

^{1}H NMR (400 MHz, $CDCl_3$) δ 8.08 (d, J = 1.9 Hz, 1H), 7.84 (dd, J = 8.4 Hz, J = 2.0 Hz, 1H), 7.58 (d, J = 8.4 Hz, 1H), 5.63 (s, 2H), 3.54 (s, 3H), 3.50-3.42 (m, 4H), 3.35 (s, 3H), 3.31-3.17 (m, 4H), 3.11-2.99 (m, 2H), 2.60-2.51 (m, 3H) 2.43-2.32 (m, 3H), 1.98-1.93 (m, 1H), 1.44 (s, 9H).

^{13}C NMR (101 MHz, $CDCl_3$) δ 164.0, 162.2, 154.9, 154.7, 151.7, 148.0, 136.8, 133.9, 131.5, 128.9, 126.1, 123.2, 104.8, 79.3, 64.5, 61.4, 61.2, 52.7, 50.5, 50.2, 45.4, 45.2, 45.1, 40.3, 30.0, 28.6 (3C), 28.5, 27.9.

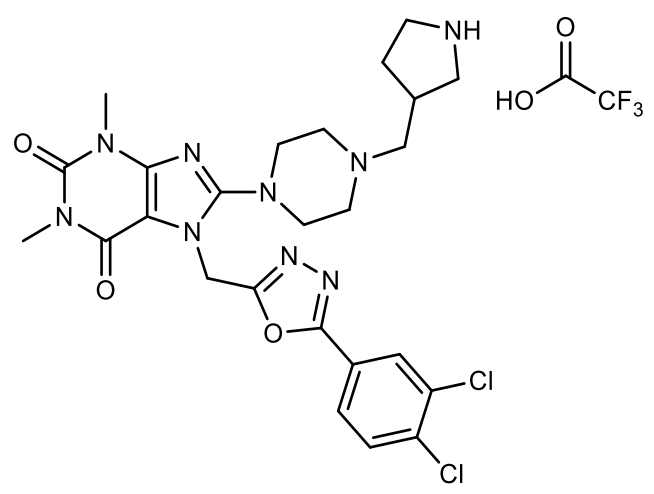

3-((4-(7-((5-(3,4-dichlorophenyl)-1,3,4-oxadiazol-2-yl)methyl)-1,3-dimethyl-2,6-dioxo-2,3,6,7-tetrahydro-1*H*-purin-8-yl)piperazin-1-yl)methyl)pyrrolidin-1-ium trifluoroacetate

3-((4-(7-((5-(3,4-dichlorophenyl)-1,3,4-oxadiazol-2-yl)methyl)-1,3-dimethyl-2,6-dioxo-2,3,6,7-tetrahydro-1*H*-purin-8-yl)piperazin-1-yl)methyl)pyrrolidin-1-ium trifluoroacetate was prepared according to the general procedure 4 using tert-butyl 3-((4-(7-((5-(3,4-dichlorophenyl)-1,3,4-oxadiazol-2-yl)methyl)-1,3-dimethyl-2,6-dioxo-2,3,6,7-tetrahydro-1*H*-purin-8-yl)piperazin-1-yl)methyl) pyrrolidine-1-carboxylate (39.8 mg, 0.059 mmol, 1eq), TFA (136 µL, 1.77 mmol, 30 eq) and DCM (2.0mL) using the general procedure 4. The crude was used directly in the next step without further purification.

ASM105

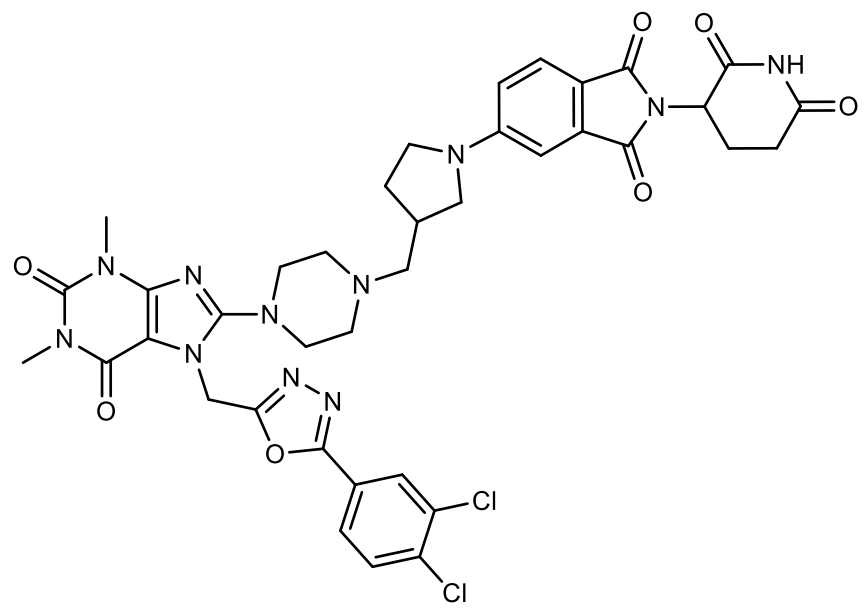

7-((5-(3,4-dichlorophenyl)-1,3,4-oxadiazol-2-yl)methyl)-8-(4-((1-(2-(2,6-dioxopiperidin-3-yl)-1,3-dioxoisoindolin-5-yl)pyrrolidin-3-yl)methyl)piperazin-1-yl)-1,3-dimethyl-3,7-dihydro-1*H*-purine-2,6-dione

ASM105 was prepared according to the general procedure 5 using 3-((4-(7-((5-(3,4-dichlorophenyl)-1,3,4-oxadiazol-2-yl)methyl)-1,3-dimethyl-2,6-dioxo-2,3,6,7-tetrahydro-1*H*-purin-8-yl)piperazin-1-yl)methyl)pyrrolidin-1-ium trifluoroacetate (40 mg, 0.058 mmol, 1 eq), 5-fluoro thalidomide (16 mg, 0.058 mmol, 1 eq), TRIETHYLAMINE (24 µL, 0.174 mmol, 3 eq) and DMSO (1.5 mL). After purification, a yellow solid was obtained (15.2 mg, 0.018 mmol, 32%).

^{1}H NMR (400 MHz, $CDCl_3$) δ 8.11 (d, J = 2.0 Hz, 1H), 8.06 (bs, 1H), 7.86 (dd, J = 2.0 Hz, J = 8.4 Hz, 1H), 7.65 (d, J = 8.4 Hz, 1H), 7.60 (d, J = 8.4 Hz, 1H), 6.94 (d, J = 2.0 Hz, 1H), 6.70-6.68 (m, 1H), 5.66 (s, 2H), 4.96-4.91 (m, 1H), 3.56 (s, 3H), 3.51 (bs, 1H), 3.46-3.41 (m, 2H), 3.37 (s, 3H), 3.34-3.29 (m, 2H), 3.24-3.19 (bs, 1H), 2.91-2.81 (m, 2H), 2.80-2.69 (m, 2H), 2.66-2.42 (m, 6H), 2.15-2.10 (m, 1H), 1.65-1.62 (m, 4H).

^{13}C NMR (101 MHz, $CDCl_3$) δ 171.3, 168.7, 168.3, 167.7, 164.1, 162.2, 155.0, 152.1, 151.7, 147.9, 136.9, 134.6, 133.9, 131.5, 128.9, 126.2, 125.6, 123.1, 116.8, 115.3, 106.3, 104.9, 61.4, 52.6, 50.4, 50.2 (2C), 49.2, 47.6, 40.3, 36.1, 35.9 (2C), 31.6, 30.0, 29.8, 28.0, 22.9.

HPLC-MS t_R = 1.66 min (99.0%), ESI+ m/z 830.598 $[M+H]^+$

HRMS was calculated for $C_{38}H_{38}Cl_2N_{11}O_7$: 830.2333, found: 830.2330

Procedure for the synthesis of ASM110

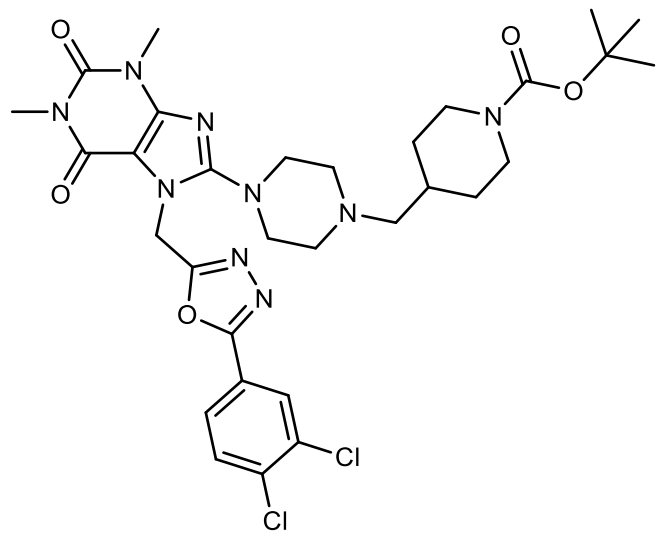

Tert-butyl 4-((4-(7-((5-(3,4-dichlorophenyl)-1,3,4-oxadiazol-2-yl)methyl)-1,3-dimethyl-2,6-dioxo-2,3,6,7-tetrahydro-1*H*-purin-8-yl)piperazin-1-yl)methyl)piperidine-1-carboxylate

Tert-butyl 4-((4-(7-((5-(3,4-dichlorophenyl)-1,3,4-oxadiazol-2-yl)methyl)-1,3-dimethyl-2,6-dioxo-2,3,6,7-tetrahydro-1*H*-purin-8-yl)piperazin-1-yl)methyl)piperidine-1-carboxylate was prepared according to the general procedure 3 using TH5427.TFA (50 mg, 0.083 mmol, 1 eq), 1-Boc-piperidine-4-carboxaldehyde (21.1 mg, 0.099 mmol, 1.2 eq), sodium triacetoxyborohydride (26.3 mg, 0.124 mmol, 1.5 eq) and THF (1.5 mL). After purification, an off-white solid was obtained (40 mg, 0.058 mmol, 70%).

^{1}H NMR (400 MHz, $CDCl_3$) δ 8.08 (d, J = 2.0 Hz, 1H), 7.83 (dd, J = 2.0 Hz, J = 8.4 Hz, 1H), 7.57 (d, J = 8.4 Hz, 1H), 5.63 (s, 2H), 4.06 (bs, 2H), 3.53 (s, 3H), 3.34 (s, 3H), 3.30-3.27 (m, 4H), 2.66 (t, J = 12.7, 2H) 2.49 (bs, 4H), 2.19 (d, J = 6.7 Hz, 2H), 1.70 (bd, J = 13.2 Hz, 2H), 1.61-1.59 (m, 1H), 1.43 (s, 9H), 1.10-1.00 (m, 2H).

^{13}C NMR (101 MHz, $CDCl_3$) δ 163.9, 162.2, 156.7, 155.0, 154.9, 151.7, 148.0, 136.8, 133.9, 131.4, 128.8, 126.1, 123.2, 104.8, 79.4, 64.4, 52.9 (2C), 50.5 (2C), 40.3 (2C), 33.6, 30.7 (2C), 29.9, 28.6 (3C), 27.9.

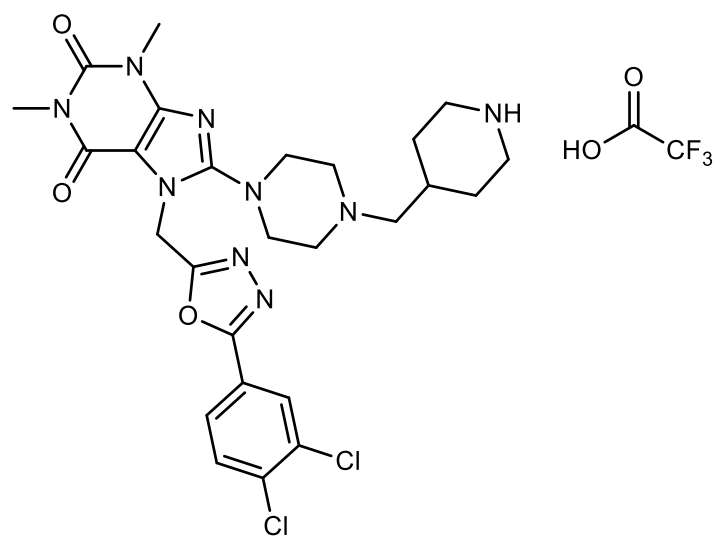

4-((4-(7-((5-(3,4-dichlorophenyl)-1,3,4-oxadiazol-2-yl)methyl)-1,3-dimethyl-2,6-dioxo-2,3,6,7-tetrahydro-1*H*-purin-8-yl)piperazin-1-yl)methyl)piperidin-1-ium trifluoroacetate

4-((4-(7-((5-(3,4-dichlorophenyl)-1,3,4-oxadiazol-2-yl)methyl)-1,3-dimethyl-2,6-dioxo-2,3,6,7-tetrahydro-1H-purin-8-yl)piperazin-1-yl)methyl)piperidin-1-ium trifluoroacetate was prepared from *tert*-butyl 4-((4-(7-((5-(3,4-dichlorophenyl)-1,3,4-oxadiazol-2-yl)methyl)-1,3-dimethyl-2,6-dioxo-2,3,6,7-tetrahydro-1H-purin-8-yl)piperazin-1-yl)methyl)piperidine-1-carboxylate (40 mg, 0.058 mmol, 1eq), TFA (134 µL, 1.743 mmol, 30 eq) and DCM (2.5 mL) using the general procedure 4. The crude was used directly in the next step without further purification.

^{1}H NMR (400 MHz, MeOD) δ 8.12-8.11 (d, J = 2.0 Hz, 1H), 7.90 (dd, J = 2.0 Hz, J = 8.4 Hz, 1H), 7.71 (d, J = 8.4 Hz, 1H), 5.77 (s, 2H), 3.71-3.59 (m, 7H), 3.49 (s, 3H), 3.48-3.42 (m, 3H), 3.24 (s, 3H), 3.23-3.21 (m, 2H), 3.10-3.01 (m, 2H), 2.35-2.27 (m, 1H), 2.11-2.07 (m, 2H), 1.63-1.50 (m, 2H) *NH.TFA not observed.*

HPLC-MS t_R = 1.33 min (90.9%), ESI+ m/z 588.714; 590.634 $[M+H]^+$ *(free NH)*

ASM110

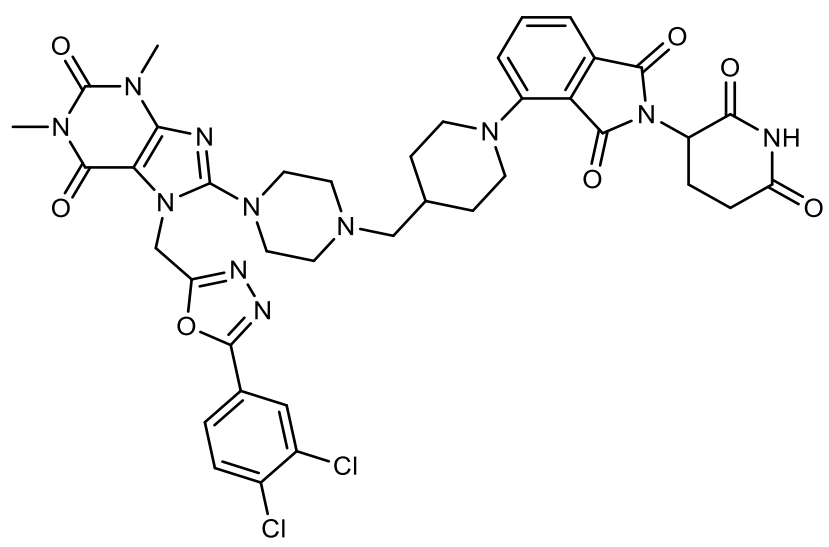

7-((5-(3,4-dichlorophenyl)-1,3,4-oxadiazol-2-yl)methyl)-8-(4-((1-(2-(2,6-dioxopiperidin-3-yl)-1,3-dioxoisoindolin-4-yl)piperidin-4-yl)methyl)piperazin-1-yl)-1,3-dimethyl-3,7-dihydro-1*H*-purine-2,6-dione

ASM110 was prepared according to the general procedure 5 using 4-((4-(7-((5-(3,4-dichlorophenyl)-1,3,4-oxadiazol-2-yl)methyl)-1,3-dimethyl-2,6-dioxo-2,3,6,7-tetrahydro-1*H*-purin-8-yl)piperazin-1-yl)methyl)piperidin-1-ium trifluoroacetate (23 mg, 0.033 mmol, 1 eq), 4-fluoro thalidomide (9 mg, 0.033 mmol, 1 eq), TRIETHYLAMINE (14 µL, 0.098 mmol, 3 eq) and DMSO (0.9 mL). After purification, a yellow solid was obtained (12.2 mg, 0.014 mmol, 44%).

^{1}H NMR (400 MHz, $CDCl_3$) δ 8.26 (bs, 1H), 8.10 (d, J = 2.0 Hz, 1H), 7.85 (dd, J = 2.0 Hz, J = 8.4 Hz, 1H), 7.59 (d, J = 8.4 Hz, 1H), 7.56-7.54 (m, 1H), 7.36 (d, J = 7.1 Hz, 1H), 7.16 (d, J = 8.4 Hz, 1H), 5.65 (s, 2H), 4.97-4.93 (m, 1H), 3.76- 3.71 (m, 2H), 3.55 (s, 3H), 3.45-3.38 (m, 2H), 3.36 (s, 3H), 2.91-2.82 (m, 4H), 2.80-2.68 (m, 3H), 2.37 (bs, 1H), 2.14-2.08 (m, 1H), 1.94-1.91 (m, 2H), 1.79-1.66 (m, 5H), 1.50-1.47 (m, 2H).

^{13}C NMR (101 MHz, $CDCl_3$) δ 171.1, 168.4, 167.5, 166.8, 164.1, 162.2, 155.0, 151.8, 150.9, 148.0, 136.9, 135.6, 134.2, 133.9, 131.5, 128.9, 126.2, 123.8, 123.2, 117.4, 115.5, 104.9, 64.4, 51.9 (2C), 51.6, 50.1, 49.2, 41.1, 40.3, 32.9, 31.6 (2C), 31.0 (2C), 30.0, 29.8, 28.0, 22.8.

HPLC-MS t_R = 1.71 min (96.6%), ESI+ m/z 844.535 $[M+H]^+$

HRMS was calculated for $C_{39}H_{40}Cl_2N_{11}O_7$: 844.2489, found: 844.2487

ASM70

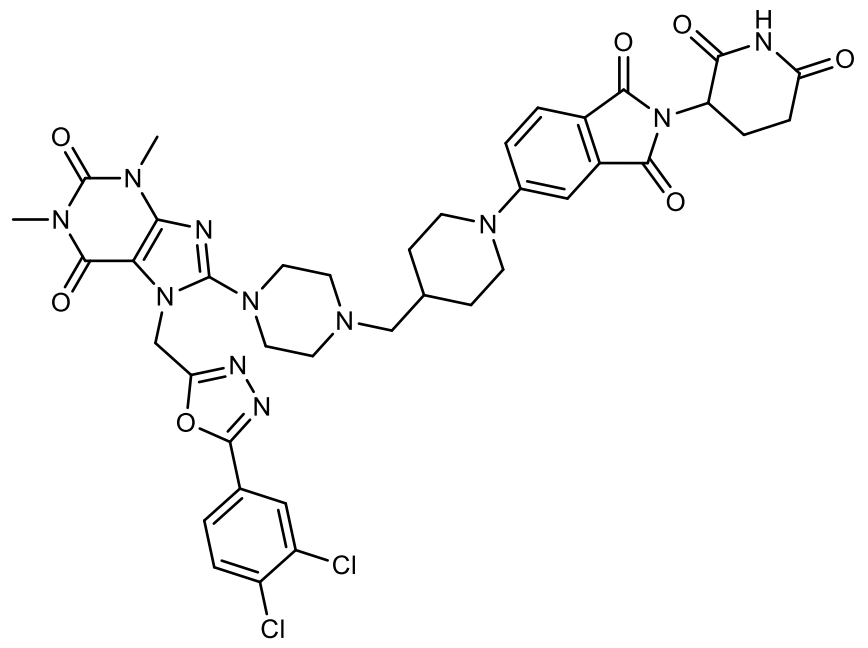

7-((5-(3,4-dichlorophenyl)-1,3,4-oxadiazol-2-yl)methyl)-8-(4-((1-(2-(2,6-dioxopiperidin-3-yl)-1,3-dioxoisoindolin-5-yl)piperidin-4-yl)methyl)piperazin-1-yl)-1,3-dimethyl-3,7-dihydro-1*H*-purine-2,6-dione

ASM70 was prepared according to the general procedure 6 using 4-((4-(7-((5-(3,4-dichlorophenyl)-1,3,4-oxadiazol-2-yl)methyl)-1,3-dimethyl-2,6-dioxo-2,3,6,7-tetrahydro-1*H*-purin-8-yl)piperazin-1-yl)methyl)piperidin-1-ium trifluoroacetate (27 mg, 0.038 mmol, 1 eq), 5-fluoro thalidomide (10.6 mg, 0.038 mmol, 1 eq), DIPEA (14 µL, 0.098 mmol, 3 eq) and DMSO (1.1 mL). After purification, a yellow solid was obtained (7.4 mg, 0.009 mmol, 23%).

^{1}H NMR (400 MHz, $CDCl_3$) δ 8.18 (s, 1H), 8.10 (d, J = 2.0 Hz, 1H), 7.85 (dd, J = 2.0 Hz, J = 8.5 Hz, 1H), 7.66 (d, J = 8.5 Hz, 1H) 7.59 (d, J = 8.5 Hz, 1H), 7.27 (d, J = 2.3 Hz, 1H), 7.03 (dd, J = 2.3 Hz, J = 8.5 Hz, 1H), 5.65 (s, 2H), 4.95-4.91 (m, 1H), 3.96-3.93 (m, 2H), 3.56 (s, 3H), 3.37 (s, 3H), 3.32 (bs, 3H), 2.98-2.90 (m, 2H), 2.86-2.68 (m, 4H), 2.53 (bs, 4H), 2.25 (bs, 2H), 2.14-2.09 (m, 1H), 1.90-1.87 (m, 3H), 1.74, 1.32-1.26 (m, 2H).

^{13}C NMR (101 MHz, $CDCl_3$) δ 171.2, 168.5, 168.2, 167.4, 164.0, 162.2, 156.7, 155.5, 155.0, 151.8, 148.1, 136.9, 134.5, 134.0, 131.5, 128.9, 126.2, 125.6, 123.2, 118.7, 118.0, 108.8, 104.9, 64.2, 53.0 (2C), 50.5 (2C), 49.3, 48.1 (2C), 40.3, 33.4, 31.6, 30.1 (2C), 30.0, 28.0, 22.9.

HPLC-MS t_R = 1.68 min (96.6%), ESI+ m/z 844.795; 845.995 $[M+H]^+$

HRMS was calculated for $C_{39}H_{40}Cl_2N_{11}O_7$: 844.2489, found: 844.2483

ASM71

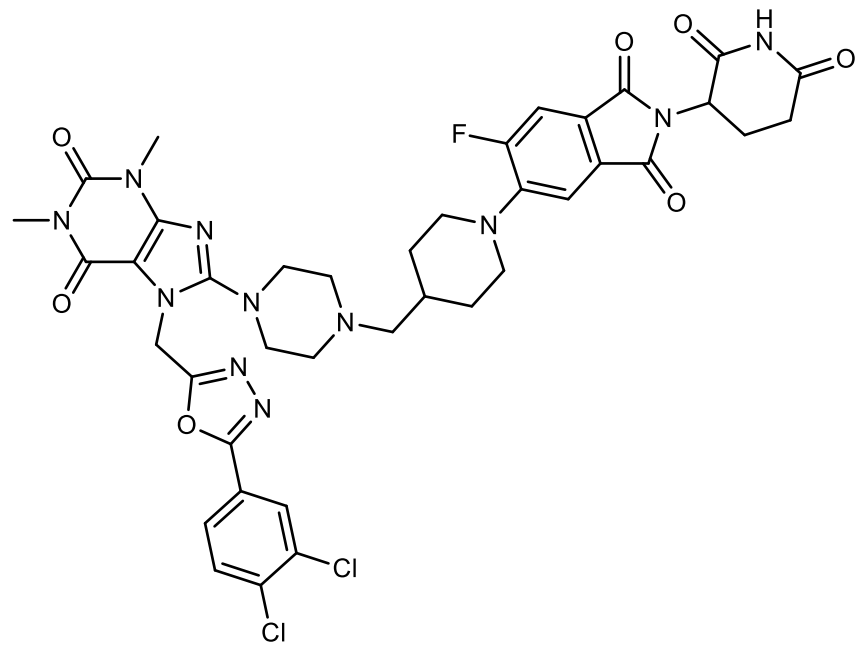

7-((5-(3,4-dichlorophenyl)-1,3,4-oxadiazol-2-yl)methyl)-8-(4-((1-(2-(2,6-dioxopiperidin-3-yl)-6-fluoro-1,3-dioxoisoindolin-5-yl)piperidin-4-yl)methyl)piperazin-1-yl)-1,3-dimethyl-3,7-dihydro-1*H*-purine-2,6-dione

ASM71 was prepared according to the general procedure 6 using 4-((4-(7-((5-(3,4-dichlorophenyl)-1,3,4-oxadiazol-2-yl)methyl)-1,3-dimethyl-2,6-dioxo-2,3,6,7-tetrahydro-1*H*-purin-8-yl)piperazin-1-yl)methyl)piperidin-1-ium trifluoroacetate (27 mg, 0.038 mmol, 1 eq), 4,5-difluoro thalidomide (11.3 mg, 0.038 mmol, 1 eq), DIPEA (14 µL, 0.098 mmol, 3 eq) and DMSO (1.1 mL). After purification, a yellow solid was obtained (6.5 mg, 0.008 mmol, 20%).

^{1}H NMR (400 MHz, $CDCl_3$) δ 8.17 (s, 1H), 8.11 (d, J = 2.0 Hz, 1H) 7.85 (dd, J = 2.0 Hz, J = 8.4 Hz, 1H), 7.59 (d, J = 8.4 Hz, 1H), 7.45 (d, J = 11.0 Hz, 1H) 7.37 (d, J = 7.2 Hz, 1H), 5.65 (s, 2H), 4.95-4.90 (m, 1H), 3.67-3.64 (m, 2H), 3.56 (s, 3H), 3.36 (s, 3H), 3.34-3.28 (m, 2H), 2.92-2.82 (m, 4H), 2.80-2.69 (m, 3H), 2.61-2.55 (m, 3H), 2.30 (bs, 1H), 2.17-2.09 (m, 1H), 1.91 (bs, 2H), 1.42 (bs, 2H), 1.28-1.25 (m, 3H).

^{19}F NMR (376 MHz, $CDCl_3$) δ -110.98

HPLC-MS t_R = 1.77 min (97.8%), ESI+ m/z 862.919; 864.240 $[M+H]^+$

ASM32

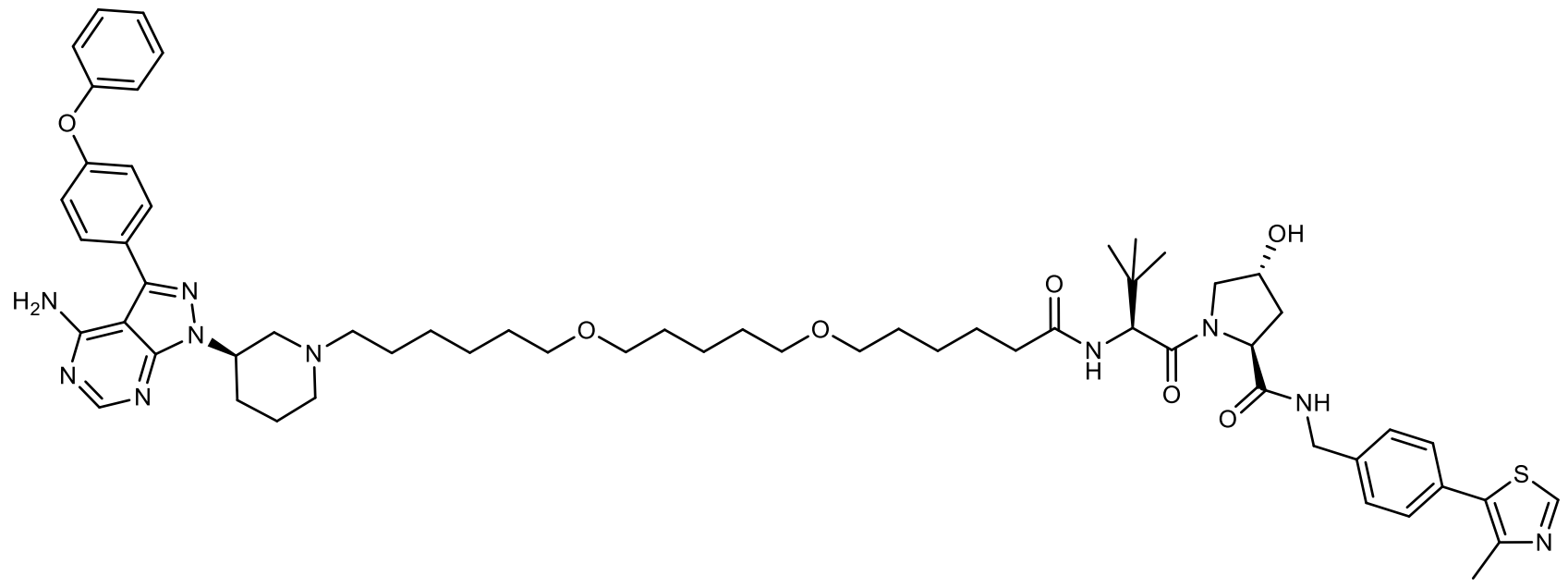

(2*S*,4*R*)-1-((*S*)-2-(6-((5-((6-((*R*)-3-(4-amino-3-(4-phenoxyphenyl)-1*H*-pyrazolo[3,4-*d*]pyrimidin-1-yl)piperidin-1-yl)hexyl)oxy)pentyl)oxy)hexanamido)-3,3-dimethylbutanoyl)-4-hydroxy-*N*-(4-(4-methylthiazol-5-yl)benzyl)pyrrolidine-2-carboxamide

(*R*)-3-(4-phenoxyphenyl)-1-(piperidin-3-yl)-1*H*-pyrazolo[3,4-*d*]pyrimidin-4-amine* (15.5 mg, 0.021 mmol, 1 eq) was dissolved in DMF (0.6 mL) then cesium carbonate (8.1 mg, 0.025 mmol, 1.2 eq) and (2*S*,4*R*)-1-((*S*)-2-(6-((5-((6-chlorohexyl)oxy)pentyl)oxy)hexanamido)-3,3-dimethylbutanoyl)-4-hydroxy-*N*-(4-(4-methylthiazol-5-yl)benzyl)pyrrolidine-2-carboxamide (8.0 mg, 0.021 mmol, 1 eq) were added. The mixture was stirred at 70°C overnight. After concentration under reduced pressure, the crude was purified by preparative HPLC to afford the expected product as white solid (3 mg, 0.003 mmol, 13%).

^{1}H NMR (400 MHz, $CDCl_3$) δ 8.66 (s, 1H), 8.35 (s, 1H), 7.64-7.62 (m, 2H), 7.40-7.29 (m, 7H), 7.18-7.12 (m, 3H), 7.08-7.05 (m, 2H), 6.51 (d, J = 8.9 Hz, 1H) 5.69 (bs, 2H), 5.04 (s, 1H), 4.72 (t, J = 8.1 Hz, 1H), 4.60-4.51 (m, 3H), 4.32 (dd, J = 5.2 Hz, 14.8 Hz, 1H), 4.13-4.10 (m, 1H), 3.59 (dd, J = 3.4 Hz, 11.3 Hz, 1H), 3.39-3.33 (m, 8H), 3.20-3.19 (m, 1H), 2.99-2.97 (m, 1H), 2.65-2.64 (m, 1H), 2.56-2.51 (m, 1H), 2.49 (s, 3H), 2.45-2.43 (m, 2H), 2.22-2.11 (m, 7H), 1.87 (bs, 2H), 1.63-1.48 (m, 12H), 1.40-1.29 (m, 8H), 0.93 (s, 9H).
^{13}C NMR (101 MHz, $CDCl_3$) δ 174.0, 172.2, 170.9, 158.5, 157.9, 156.6, 155.7, 154.2, 150.4, 148.6, 148.1, 143.7, 138.2, 131.7, 131.1, 130.2 (2C), 130.1 (2C), 129.7 (2C), 128.3 (2C), 128.1, 124.1, 119.6 (2C), 119.3 (2C), 100.0, 98.6, 71.0, 70.9, 70.8, 70.7, 70.1, 58.6, 57.7, 57.0, 53.8, 53.3, 43.4, 36.4, 36.0, 35.0, 31.0, 30.1, 29.8, 29.7, 29.6, 29.5, 27.4, 26.6 (3C), 26.2, 26.0, 25.5, 24.4, 22.9, 16.2.
HPLC-MS t_R = 1.82 min (97.9%), ESI+ m/z 1100.346; 1103.450 $[M+H]^+$
* Synthesized according to literature[2]

ASM73

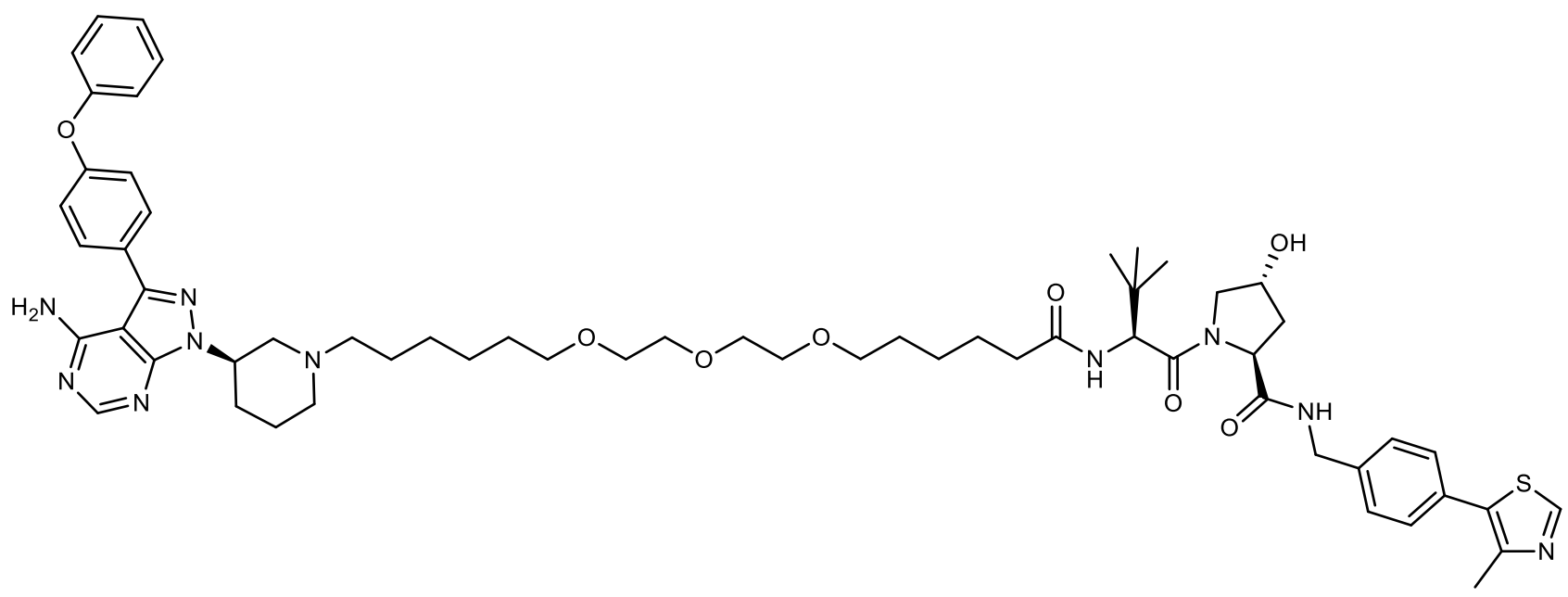

(2*S*,4*R*)-1-((*S*)-22-((*R*)-3-(4-amino-3-(4-phenoxyphenyl)-1*H*-pyrazolo[3,4-d]pyrimidin-1-yl)piperidin-1-yl)-2-(tert-butyl)-4-oxo-10,13,16-trioxa-3-azadocosanoyl)-4-hydroxy-*N*-(4-(4-methylthiazol-5-yl)benzyl)pyrrolidine-2-carboxamide

(*R*)-3-(4-phenoxyphenyl)-1-(piperidin-3-yl)-1*H*-pyrazolo[3,4-*d*]pyrimidin-4-amine (11.3 mg, 0.029 mmol, 1 eq) was dissolved in DMF (0.84 mL) then cesium carbonate (11.4 mg, 0.035 mmol, 1.2 eq) and (2*S*,4*R*)-1-((*S*)-2-(*tert*-butyl)-22-chloro-4-oxo-10,13,16-trioxa-3-azadocosanoyl)-4-hydroxy-*N*-(4-(4-methylthiazol-5-yl)benzyl) pyrrolidine-2-carboxamide (22 mg, 0.029 mmol, 1 eq) were added. The mixture was stirred at 70°C overnight. After concentration under reduced pressure, the crude was purified by preparative HPLC to afford the expected product as white solid (3.5 mg, 0.003 mmol, 11%).

Major rotamer

^{1}H NMR (400 MHz, $CDCl_3$) δ 8.67 (s, 1H), 7.64-7.61 (m, 1H), 7.40-7.30 (m, 6H), 7.18-7.11 (m, 2H), 6.23 (d, J = 8.8 Hz, 1H), 4.70 (t, J = 8.1 Hz, 1H), 4.59-4.50 (m, 3H), 4.35-4.29 (m, 1H), 4.10-4.07 (m, 1H), 3.63-3.50 (m, 13H), 3.46-3.39 (m, 5H), 2.83-2.66 (m, 5H), 2.50 (s, 3H), 2.49-2.45 (m, 2H), 2.21-2.10 (m, 4H), 1.80-1.72 (m, 2H), 1.64-1.51 (m, 8H), 1.48-1.29 (m, 8H), 0.92 (s, 9H).

ASM74

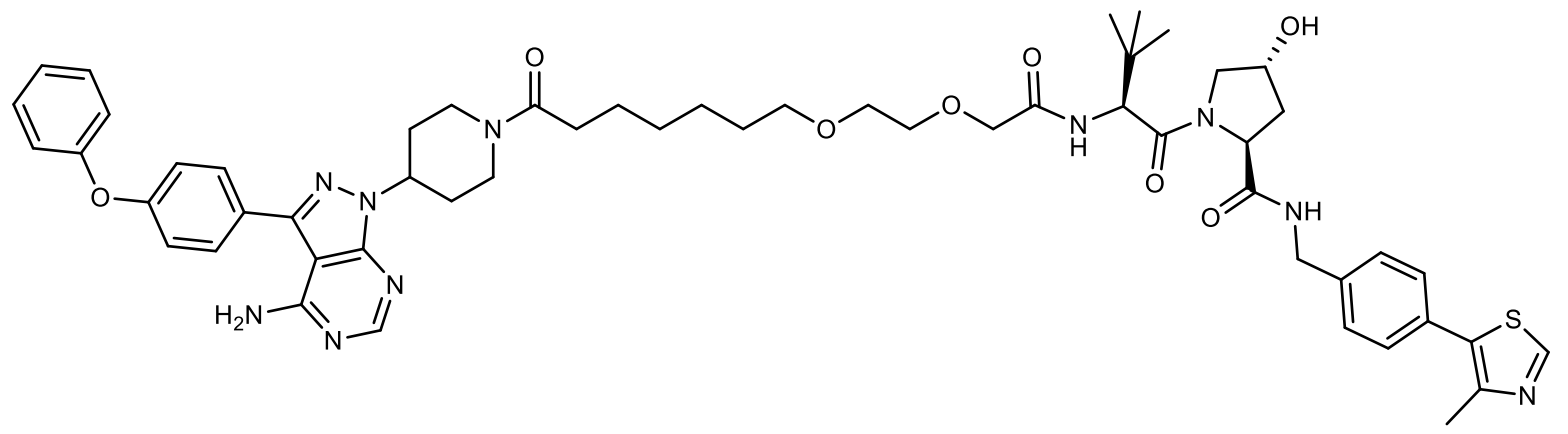

(2*S*,4*R*)-1-((*S*)-2-(2-(2-((7-(4-(4-amino-3-(4-phenoxyphenyl)-1*H*-pyrazolo[3,4-*d*]pyrimidin-1-yl)piperidin-1-yl)-7-oxoheptyl)oxy)ethoxy)acetamido)-3,3-dimethylbutanoyl)-4-hydroxy-*N*-(4-(4-methylthiazol-5-yl)benzyl)pyrrolidine-2-carboxamide

ASM74 was prepared according to the general procedure 2 using 3-(4-phenoxyphenyl)-1-(piperidin-3-yl)-1*H*-pyrazolo[3,4-*d*]pyrimidin-4-amine (7.7 mg, 0.020 mmol, 1.1 eq), 7-(2-(2-(((*S*)-1-((2*S*,4*R*)-4-hydroxy-2-((4-(4-methylthiazol-5-yl)benzyl)carbamoyl)pyrrolidin-1-yl)-3,3-dimethyl-1-oxobutan-2-yl)amino)-2-oxoethoxy)ethoxy)heptanoic acid (12 mg, 0.018 mmol, 1 eq), DIPEA (6 µL, 0.036 mmol, 2 eq) and HATU (13.8 mg, 0.036 mmol, 2 eq). After purification, a white solid was obtained (7.2 mg, 0.007 mmol, 39%).

^{1}H NMR (400 MHz, CDCl$_3$) δ 8.66 (s, 1H), 8.35 (s, 1H), 7.64-7.60 (m, 2H), 7.57-7.52 (m, 1H), 7.40-7.31 (m, 7H), 7.19-7.12 (m, 3H), 7.08-7.06 (m, 2H), 5.70 (s, 2H), 5.02-4.96, 4.79-4.69 (m, 2H), 4.59-4.50 (m, 3H), 4.34 (dd, J = 15.0 Hz, J = 5.5 Hz, 1H), 4.10-4.03 (m, 2H), 4.00-3.99 (m, 2H), 3.67-3.57 (m, 4H), 3.46 (t, J = 6.5 Hz, 2H), 3.25 (t, J = 13.1 Hz, 1H), 2.82-2.78 (m, 1H), 2.50 (s, 3H), 2.47-2.45 (m, 1H), 2.38-2.31 (m, 2H), 2.27-2.20 (m, 2H), 2.16-2.03 (m, 5H), 1.65-1.55 (m, 4H), 1.36-1.34 (m, 4H), 0.96 (s, 9H).

HPLC-MS t_R = 1.59 min (98.7%), ESI+ m/z 1030.154 $[M+H]^+$

ASM75

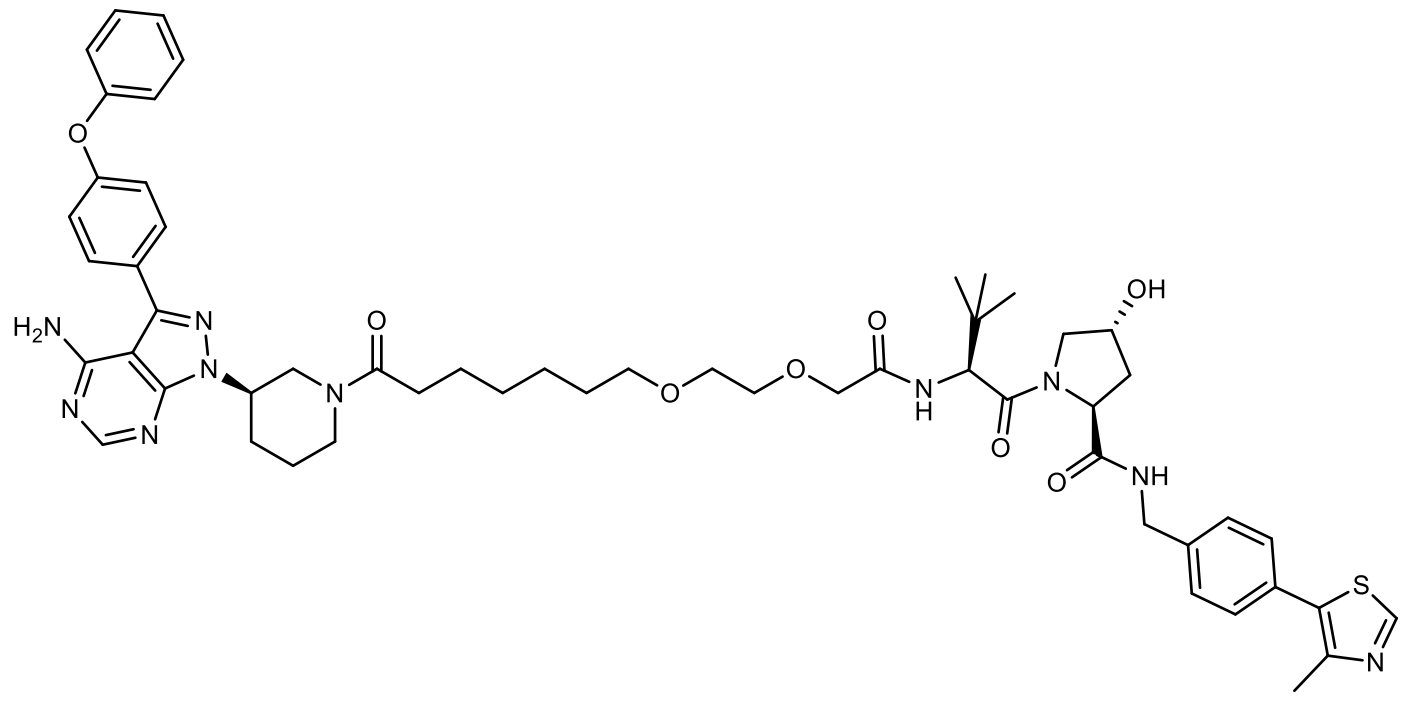

(2*S*,4*R*)-1-((*S*)-2-(2-(2-((7-((*R*)-3-(4-amino-3-(4-phenoxyphenyl)-1*H*-pyrazolo[3,4-d]pyrimidin-1-yl)piperidin-1-yl)-7-oxoheptyl)oxy)ethoxy)acetamido)-3,3-dimethylbutanoyl)-4-hydroxy-*N*-(4-(4-methylthiazol-5-yl)benzyl)pyrrolidine-2-carboxamide

ASM75 was prepared according to the general procedure 2 using (*R*)-3-(4-phenoxyphenyl)-1-(piperidin-3-yl)-1*H*-pyrazolo[3,4-*d*]pyrimidin-4-amine (7.7 mg, 0.020 mmol, 1.1 eq), 7-(2-(2-(((*S*)-1-((2*S*,4*R*)-4-hydroxy-2-((4-(4-methylthiazol-5-yl)benzyl)carbamoyl)pyrrolidin-1-yl)-3,3-dimethyl-1-oxobutan-2-yl)amino)-2-oxoethoxy)ethoxy)heptanoic acid (12 mg, 0.018 mmol, 1 eq), DIPEA (6 µL, 0.036 mmol, 2 eq) and HATU (13.8 mg, 0.036 mmol, 2 eq). After purification, a white solid was obtained (8.7 mg, 0.008 mmol, 46%).

Major rotamer

^{1}H NMR (400 MHz, $CDCl_3$) δ 8.65 (s, 1H), 8.34 (s, 1H), 7.64-7.55 (m, 3H), 7.41-7.32 (m, 7H), 7.19-7.13 (m, 3H), 7.09-7.06 (m, 2H), 5.75 (s, 2H), 4.85-4.69 (m, 2H), 4.59-4.49 (m, 3H), 4.36-4.31 (m, 1H), 4.09-4.05 (m, 1H), 4.02-3.97 (m, 2H), 3.67-3.53 (m, 6H), 3.48-3.40 (m, 2H), 3.30 (t, J = 12.6 Hz, 1H), 2.49 (s, 3H), 2.48-2.44 (m, 1H), 2.39-2.29 (m, 3H), 2.25-2.19 (m, 3H), 2.16-2.11 (m, 2H), 2.00-1.95 (m, 1H), 1.68-1.52 (m, 5H), 1.35-1.28 (m, 4H), 0.95 (s, 9H).

HPLC-MS t_R = 1.66 min (98.8%), ESI+ m/z 1030.090 $[M+H]^+$

Procedures for the synthesis of dNUDT5*

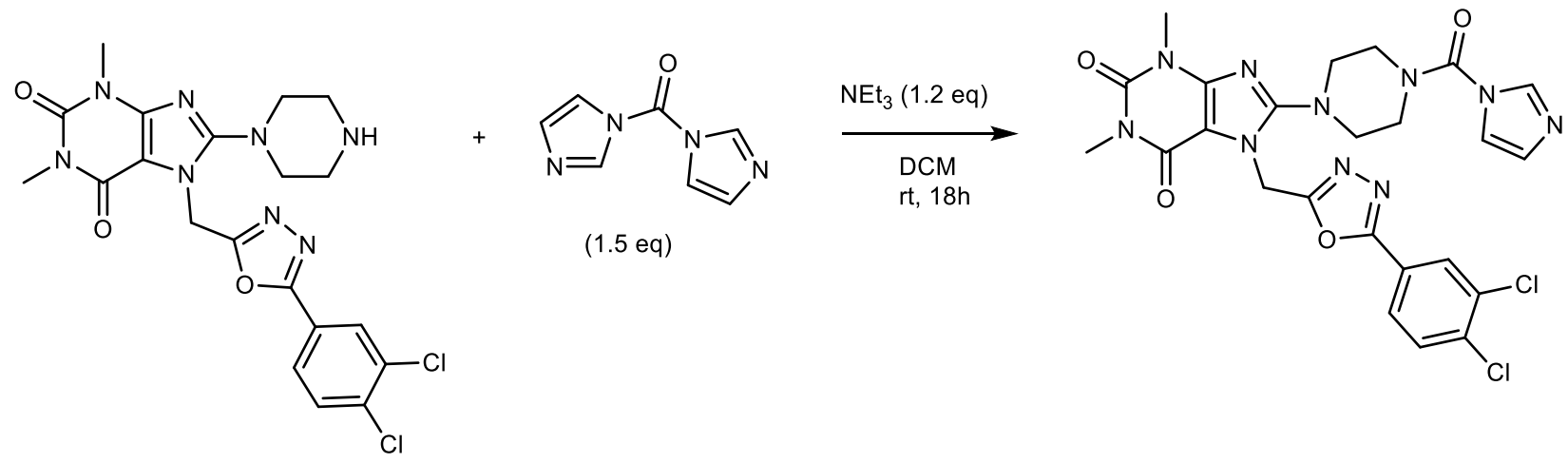

8-(4-(1*H*-imidazole-1-carbonyl)piperazin-1-yl)-7-((5-(3,4-dichlorophenyl)-1,3,4-oxadiazol-2-yl)methyl)-1,3-dimethyl-3,7-dihydro-1*H*-purine-2,6-dione

Under nitrogen, 1,1′-carbonyldiimidazole (88.6 mg, 0.546 mmol, 1.5 eq) was added at -5°C to a mixture of TH5427 (179 mg, 0.364 mmol, 1 eq) and triethylamine (61 µL, 0.437 mmol, 1.2 eq) in DCM (14.3 mL). The mixture was then stirred overnight at room temperature. After concentration under reduced pressure, MeOH was added and the resulting solid was collected by filtration to afford the expected product as white solid (213 mg, 0.364 mmol, quantitative).

^{1}H NMR (400 MHz, $CDCl_3$) δ 8.12 (d, 1H, J = 2.0 Hz), 8.03 (bs, 1H), 7.87 (dd, 1H, J = 2.0 Hz J = 8.4 Hz), 7.61 (d, 1H, J = 8.4 Hz), 7.24 (bs, 1H), 7.16 (bs, 1H), 5.77 (s, 2H), 3.79-3.77 (m, 4H), 3.56 (s, 3H), 3.41-3.40 (m, 4H), 3.39 (s, 3H).

^{13}C NMR (101 MHz, $CDCl_3$) δ 164.3, 161.9, 155.2, 155.1, 151.6, 150.9, 147.6, 137.1, 137.0, 134.0, 131.6, 129.8, 128.9, 126.2, 123.0, 118.1, 105.1, 50.5, 45.9 (2C), 40.0 (2C), 30.0, 28.0.

HPLC-MS t_R = 1.46 min (97.2%), ESI+ m/z 585.533; 587.153 $[M+H]^+$

*Adapted from[3]

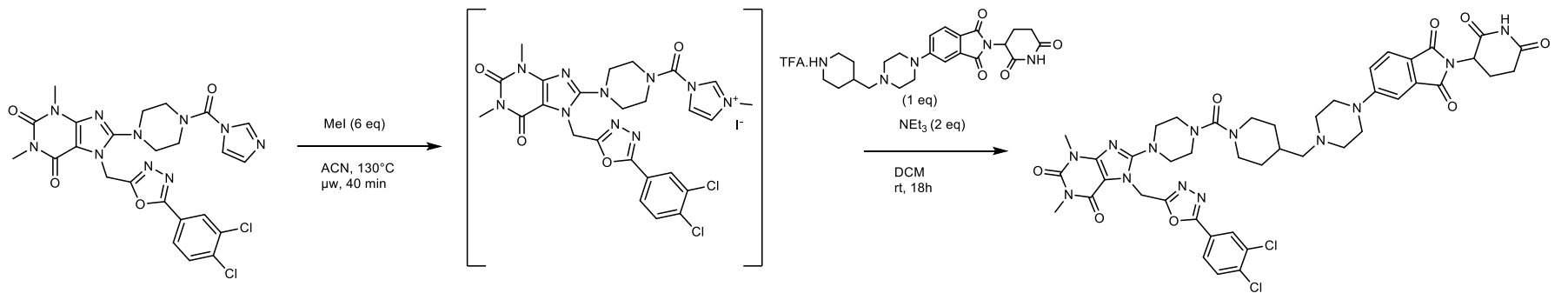

7-((5-(3,4-dichlorophenyl)-1,3,4-oxadiazol-2-yl)methyl)-8-(4-(4-((4-(2-(2,6-dioxopiperidin-3-yl)-1,3-dioxoisoindolin-5-yl)piperazin-1-yl)methyl)piperidine-1-carbonyl)piperazin-1-yl)-1,3-dimethyl-3,7-dihydro-1*H*-purine-2,6-dione

Under N_2, methyl iodide (16 µL, 0.256 mmol, 6 eq) was added to a mixture of 8-(4-(1H-imidazole-1-carbonyl)piperazin-1-yl)-7-((5-(3,4-dichlorophenyl)-1,3,4-oxadiazol-2-yl)methyl)-1,3-dimethyl-3,7-dihydro-1H-purine-2,6-dione (25 mg, 0.043 mmol, 1 eq) in acetonitrile (1.7 mL). The mixture was stirred for 40 min at 130°C under microwave heating (the reaction was checked by LCMS – 100% conversion).* After concentration under reduced pressure, the crude was dissolved in DCM (2 mL). Under N_2, triethylamine (12 µL, 0.086 mmol, 2 eq) and 2-(2,6-dioxopiperidin-3-yl)-5-(4-((1-(2,2,2-trifluoroacetyl)-1λ4-piperidin-4-yl)methyl)piperazin-1-yl)isoindoline-1,3-dione, trifluoroacetic salt (23.8 mg, 0.043 mmol, 1 eq) were added. The mixture was stirred overnight at room temperature. After concentration under reduced pressure, the crude was purified by HPLC to afford the expected product as yellow solid (6.9 mg, 0.007 mmol, 17% over 2 steps).

^{1}H NMR (400 MHz, $CDCl_3$) δ 8.11 (d, J = 2.0 Hz, 1H), 8.06 (s, 1H), 7.86 (dd, J = 2.0 Hz, J = 8.4 Hz, 1H), 7.67 (d, J = 8.4 Hz, 1H), 7.60 (d, J = 8.4 Hz, 1H), 7.28 (d, J = 2.2 Hz, 1H), 7.05 (dd, J = 2.0 Hz, J = 8.5 Hz, 1H), 5.66 (s, 2H), 4.96-4.91 (m, 1H), 3.75-3.72 (m, 2H), 3.55 (s, 3H), 3.43-3.40 (m, 4H), 3.37 (s, 3H), 3.35-3.33 (m, 3H), 3.30-3.28 (m, 4H), 2.91-2.81 (m, 2H), 2.80-2.69 (m, 3H), 2.60-2.55 (m, 3H), 2.27-2.26 (m, 2H), 2.17-2.10 (m, 1H), 1.81-1.78 (m, 2H), 1.74-1.69 (m, 2H), 1.21-1.12 (m, 2H)
^{13}C NMR (101 MHz, $CDCl_3$) δ 171.0, 168.3, 168.0, 167.3, 164.1, 164.0, 162.1, 156.3, 155.6, 155.0, 151.7, 147.9, 136.9, 134.4, 134.0, 131.5, 128.9, 126.2, 125.5, 123.1, 119.7, 118.0, 108.8, 104.9, 64.4, 53.2 (2C), 50.5 (2C), 49.3, 47.6 (2C), 47.1 (2C), 46.5 (2C), 40.2, 33.9, 31.6, 30.8 (2C), 30.0, 28.0, 22.9.
HPLC-MS t_R = 1.56 min (96.9%), ESI+ m/z 957.9246; 959.9651 $[M+H]^+$
HRMS was calculated for $C_{44}H_{48}Cl_2N_{13}O_8$: 956.3126, found: 956.3120

*Adapted from[4]

Procedures for the synthesis of dNUDT5nc

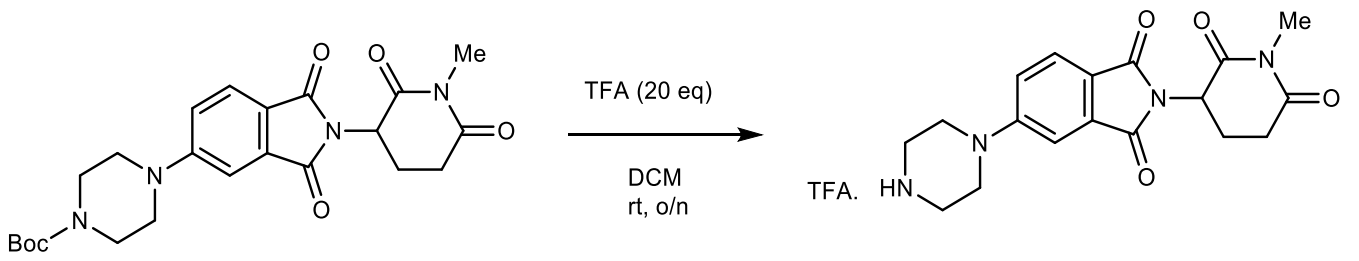

2-(1-methyl-2,6-dioxopiperidin-3-yl)-5-(piperazin-1-yl)isoindoline-1,3-dione (trifluoroacetic salt)

Trifluoroacetic acid (429 µL, 5.564 mmol, 20 eq) was added dropwise to a solution of tert-butyl 4-(2-(1-methyl-2,6-dioxopiperidin-3-yl)-1,3-dioxoisoindolin-5-yl)piperazine-1-carboxylate (127 mg, 0.278 mmol, 1 eq) in DCM (4 mL) at 0°C. The mixture was stirred for 3 h at room temperature. Water was added and the aqueous layer was recovered and concentrated under reduced pressure to afford the expected product as a yellow oil (99.5 mg, 0.212 mmol, 76%).

^{1}H NMR (400 MHz, MeOD) δ 7.74 (d, J = 8.5 Hz, 1H), 7.44 (d, J = 2.3 Hz, 1H), 7.32 (dd, J = 2.3 Hz, J = 8.5 Hz, 1H), 5.11 (dd, J = 5.5 Hz, J = 13.0 Hz, 1H), 3.71-3.69 (m, 4H), 3.41-3.38 (m, 4H), 3.31 (s, 3H), 2.91-2.87 (m, 2H), 2.75-2.68 (m, 1H), 2.13-2.06 (m, 1H).

^{13}C NMR (101 MHz, MeOD) δ 173.6, 171.4, 169.0, 168.7, 156.2, 135.5, 126.1, 120.6, 110.5, 51.2, 49.0, 46.0 (2C), 44.3 (2C), 32.5, 27.3, 22.9 (TFA not detected).

HPLC-MS t_R = 1.08 min (100%), ESI+ m/z 357.4402 $[M+H]^+$

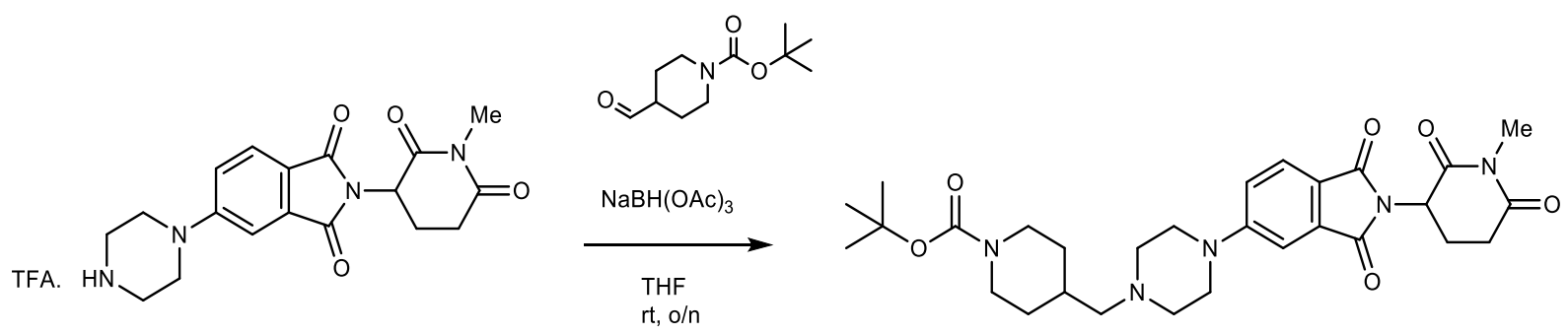

Tert-butyl 4-((4-(2-(1-methyl-2,6-dioxopiperidin-3-yl)-1,3-dioxoisoindolin-5-yl)piperazin-1-yl)methyl)piperidine-1-carboxylate

Sodium triacetoxyborohydride (67 mg, 0.316 mmol, 1.5 eq) was added to a mixture of 2-(1-methyl-2,6-dioxopiperidin-3-yl)-5-(piperazin-1-yl)isoindoline-1,3-dione, trifluoroacetic salt (99 mg, 0.210 mmol, 1 eq) and tert-butyl 4-formylpiperidine-1-carboxylate (54 mg, 0.253 mmol, 1.2 eq) in THF (4.6 mL). The mixture was stirred overnight at room temperature. After concentration under reduced pressure, the crude was purified by column chromatography (Si-35, DCM/MeOH gradient from 100% DCM to 95%) to afford the expected product as yellow solid (112.3 mg, 0.203 mmol, 96%).

^{1}H NMR (400 MHz, $CDCl_3$) δ 7.65 (d, J = 8.6 Hz, 1H), 7.24 (d, J = 2.3 Hz, 1H), 7.02 (dd, J = 2.3 Hz, J = 8.6 Hz, 1H), 4.94-4.90 (m, 1H), 4.07 (bs, 2H), 3.41 (bs, 4H), 3.16 (s, 3H), 2.98-2.89 (m, 1H), 2.81-2.65 (m, 4H), 2.58-2.56 (m, 4H), 2.24 (bs, 2H), 2.08-2.04 (m, 1H), 1.75-1.67 (m, 3H), 1.43 (s, 9H), 1.13-1.03 (m, 2H).

^{13}C NMR (101 MHz, $CDCl_3$) δ 171.3, 169.1, 168.1, 167.4, 155.4, 154.9, 134.3, 134.3, 125.3, 117.9, 108.6, 79.3, 64.3, 53.0 (4C), 49.9, 47.4 (2C), 33.5, 32.0 (2C), 30.7, 28.5, 27.2, 22.1 (C=O Boc not detected).

HPLC-MS t_R = 1.77 min (97.3%), ESI+ m/z 554.8069 $[M+H]^+$

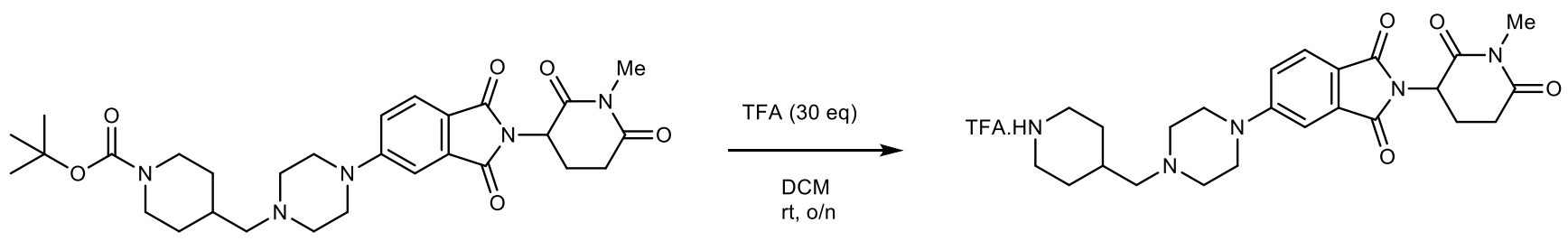

2-(1-methyl-2,6-dioxopiperidin-3-yl)-5-(4-(piperidin-4-ylmethyl)piperazin-1-yl)isoindoline-1,3-dione (trifluoroacetic salt)

Trifluoroacetic acid (468 µL, 6.069 mmol, 30 eq) was added dropwise to a solution of tert-butyl 4-((4-(2-(1-methyl-2,6-dioxopiperidin-3-yl)-1,3-dioxoisoindolin-5-yl)piperazin-1-yl)methyl)piperidine-1-carboxylate (835 mg, 1.547 mmol, 1 eq) in DCM (10 mL) at 0°C. The mixture was stirred for 3h at room temperature. Water was added and the aqueous layer was recovered and concentrated under reduced pressure to afford the expected product as a yellow solid (863 mg, 1.54 mmol, quantitative).

^{1}H NMR (400 MHz, MeOD) δ 7.77 (d, J = 8.5 Hz, 1H), 7.48 (d, J = 2.3 Hz, 1H), 7.35 (dd, J = 2.3 Hz, J = 8.5 Hz, 1H), 5.15-5.10 (m, 1H), 3.78 (bs, 3H), 3.48-3.45 (m, 6H), 3-18-3.16 (m, 2H), 3.14 (s, 3H), 3.09-3.02 (m, 2H), 2.91-2.87 (m, 2H), 2.75-2.64 (m, 1H), 2.32-2.27 (m, 1H), 2.12-2.08 (m, 3H), 1.61-1.50 (m, 2H) (TFA not detected).

^{13}C NMR (101 MHz, MeOD) δ 173.6, 171.4, 169.0, 168.7, 155.9, 135.6, 126.1, 122.9, 120.5, 110.4, 62.3, 53.1 (2C), 51.2, 46.1 (2C), 44.4 (2C), 32.5, 30.3, 27.8 (2C), 27.3, 23.0 (TFA not detected).

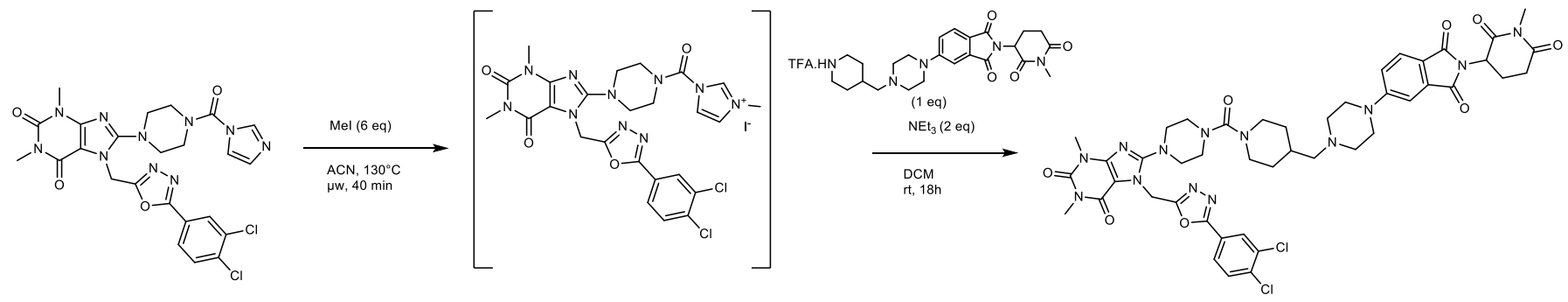

7-((5-(3,4-dichlorophenyl)-1,3,4-oxadiazol-2-yl)methyl)-1,3-dimethyl-8-(4-(4-((4-(2-(1-methyl-2,6-dioxopiperidin-3-yl)-1,3-dioxoisoindolin-5-yl)piperazin-1-yl)methyl)piperidine-1-carbonyl)piperazin-1-yl)-3,7-dihydro-1*H*-purine-2,6-dione

Under N_2, methyl iodide (16 µL, 0.256 mmol, 6 eq) was added to a mixture of 8-(4-(1H-imidazole-1-carbonyl)piperazin-1-yl)-7-((5-(3,4-dichlorophenyl)-1,3,4-oxadiazol-2-yl)methyl)-1,3-dimethyl-3,7-dihydro-1*H*-purine-2,6-dione (25 mg, 0.043 mmol, 1 eq) in acetonitrile (1.7 mL). The mixture was stirred for 40 min at 130°C under microwave heating (the reaction was checked by LCMS – 95% conversion). After concentration under reduced pressure, the crude was dissolved in DCM (1.8 mL). Under N_2, were added triethylamine (12 µL, 0.086 mmol, 2 eq) and 2-(1-methyl-2,6-dioxopiperidin-3-yl)-5-(4-(piperidin-4-ylmethyl)piperazin-1-yl)isoindoline-1,3-dione, trifluoroacetic salt (24.4 mg, 0.043 mmol, 1 eq). The mixture was stirred overnight at room temperature. After concentration under reduced pressure, the crude was purified by HPLC to afford the expected product as yellow solid (5.9 mg, 0.006 mmol, 14% over 2 steps).

^{1}H NMR (400 MHz, $CDCl_3$) δ 8.12 (d, J = 2.0 Hz, 1H), 7.87 (dd, J = 2.0 Hz, J = 8.4 Hz, 1H), 7.70 (d, J = 8.4 Hz, 1H), 7.60 (d, J = 8.4 Hz, 1H), 7.29 (d, J = 2.0 Hz, 1H), 7.06 (dd, J = 2.0 Hz, J = 8.4 Hz, 1H), 5.67 (s, 2H), 4.97-4.92 (m, 1H), 3.76-3.73 (m, 2H), 3.56 (s, 3H), 3.44-3.41 (m, 4H), 3.38 (s, 3H), 3.36-3.34 (m, 3H), 3.31-3.29 (m, 4H), 3.32 (s, 3H), 3.00-2.94 (m, 1H), 2.85-2.74 (m, 4H), 2.60-2.57 (m, 4H), 2.27 (d, J = 6.7 Hz, 2H), 2.13-2.08 (m, 1H), 1.83-1.79 (m, 2H), 1.22-1.11 (m, 4H).

^{13}C NMR (101 MHz, $CDCl_3$) δ 171.4, 169.1, 168.2, 167.5, 164.1, 164.0, 162.1, 156.3, 155.6, 155.0, 151.7, 147.9, 137.0, 134.4, 134.0, 131.5, 128.9, 126.2, 125.5, 123.1, 119.8, 118.0, 108.7, 105.0, 77.2, 64.4, 53.2 (2C), 50.5 (2C), 50.1, 47.6 (2C), 47.1 (2C), 46.5 (2C), 40.2, 33.9, 32.1, 30.8 (2C), 30.0, 28.0, 27.4, 22.2.

HPLC-MS t_R = 1.71 min (98.7%), ESI+ m/z 970.7936; 971.9340 $[M+H]^+$

Supplementary References

1 Sun, Y. *et al.* PROTAC-induced BTK degradation as a novel therapy for mutated BTK C481S induced ibrutinib-resistant B-cell malignancies. *Cell Res* **28**, 779-781 (2018). https://doi.org/10.1038/s41422-018-0055-1

2 Liu, N. *et al.* Direct and two-step bioorthogonal probes for Bruton's tyrosine kinase based on ibrutinib: a comparative study. *Org Biomol Chem* **13**, 5147-5157 (2015). https://doi.org/10.1039/c5ob00474h

3 Patrick, D. A. *et al.* Urea Derivatives of 2-Aryl-benzothiazol-5-amines: A New Class of Potential Drugs for Human African Trypanosomiasis. *J Med Chem* **60**, 957-971 (2017). https://doi.org/10.1021/acs.jmedchem.6b01163

4 Batey, R. A., Santhakumar, V., Yoshina-Ishii, C. & Taylor, S. D. An efficient new protocol for the formation of unsymmetrical tri- and tetrasubstituted ureas. *Tetrahedron Lett* **39**, 6267-6270 (1998). https://doi.org/10.1016/S0040-4039(98)01330-6
