# Supplementary material for: Targeted Protein Degradation of NUDT5 Dissociates Catalytic Inhibition from Protein Loss in 6-Thioguanine Response
Source: Nat Commun. 2026 Jun 30;17:8192. doi: 10.1038/s41467-026-74489-9 (PMC13462928; doi:10.1038/s41467-026-74489-9)
Supplement: Supplementary file 2 [file 41467_2026_74489_MOESM2_ESM.pdf]

nature portfolio

Corresponding author(s): Kilian Huber
Last updated by author(s): May 13, 2026

Reporting Summary

Nature Portfolio wishes to improve the reproducibility of the work that we publish. This form provides structure for consistency and transparency in reporting. For further information on Nature Portfolio policies, see our Editorial Policies and the Editorial Policy Checklist.

Statistics

For all statistical analyses, confirm that the following items are present in the figure legend, table legend, main text, or Methods section.

n/a	Confirmed	
☐	☒	The exact sample size (*n*) for each experimental group/condition, given as a discrete number and unit of measurement
☐	☒	A statement on whether measurements were taken from distinct samples or whether the same sample was measured repeatedly
☐	☒	The statistical test(s) used AND whether they are one- or two-sided<br>*Only common tests should be described solely by name; describe more complex techniques in the Methods section.*
☐	☒	A description of all covariates tested
☐	☒	A description of any assumptions or corrections, such as tests of normality and adjustment for multiple comparisons
☐	☒	A full description of the statistical parameters including central tendency (e.g. means) or other basic estimates (e.g. regression coefficient) AND variation (e.g. standard deviation) or associated estimates of uncertainty (e.g. confidence intervals)
☐	☒	For null hypothesis testing, the test statistic (e.g. *F*, *t*, *r*) with confidence intervals, effect sizes, degrees of freedom and *P* value noted<br>*Give P values as exact values whenever suitable.*
☐	☒	For Bayesian analysis, information on the choice of priors and Markov chain Monte Carlo settings
☐	☒	For hierarchical and complex designs, identification of the appropriate level for tests and full reporting of outcomes
☐	☒	Estimates of effect sizes (e.g. Cohen's *d*, Pearson's *r*), indicating how they were calculated

Our web collection on statistics for biologists contains articles on many of the points above.

Software and code

Policy information about availability of computer code

Data collection	Luminescence: PHERAstar FS or FSX plate reader; Western blot imaging: Odyssey imager (Licor); Proteomics: Orbitrap Fusion Lumos Tribrid Mass Spectrometer (Thermo Scientific); X-ray: Eiger2 XE 16M detector, NMR: Bruker NMR spectrometers (400 MHz); LCMS: Waters FractionLynx software; HRMS: Agilent 6530 RapidFire QTOF mass spectrometer
Data analysis	Proteomics: DIA-NN v.1.8.1, Perseus v.2.0.9.0, R studio v.4.3.2; X-ray: PHASER, Coot, PHENIX, ChimeraX; Statistical analysis: GraphPad Prism v.9 and v.10; NMR and LCMS: MestreNova v.14.3.0; HRMS: MassHunter Qualitative Analysis B.06.00 (Agilent)

For manuscripts utilizing custom algorithms or software that are central to the research but not yet described in published literature, software must be made available to editors and reviewers. We strongly encourage code deposition in a community repository (e.g. GitHub). See the Nature Portfolio guidelines for submitting code & software for further information.

Data

Policy information about availability of data

All manuscripts must include a data availability statement. This statement should provide the following information, where applicable:

- Accession codes, unique identifiers, or web links for publicly available datasets
- A description of any restrictions on data availability
- For clinical datasets or third party data, please ensure that the statement adheres to our policy

All data presented in this study are included in the article and its Supplementary Information. Source data are provided with this paper. The mass spectrometry proteomics data have been deposited to the ProteomeXchange Consortium via the PRIDE partner repository with the dataset identifier PXD068003 [https://

www.ebi.ac.uk/pride/archive/projects/PXD068003] (Total proteome analysis of NUDT5 PROTACs). The metabolomics raw data generated in this study have been deposited to the MassIVE repository with the dataset identifier MSV000101725 [ftp://massive-ftp.ucsd.edu/v12/MSV000101725/] (Metabolomics analysis of dNUDT5 treated cells). The crystallographic coordinates and structure factor data generated in this study have been deposited in the Protein Data Bank with the accession code 9G6Y [https://doi.org/10.2210/pdb9G6Y/pdb]. Additional crystal structures used in this study are 8RDZ [https://doi.org/10.2210/pdb8RDZ/pdb] and 8RIY [https://doi.org/10.2210/pdb8RIY/pdb].

Research involving human participants, their data, or biological material

Policy information about studies with human participants or human data. See also policy information about sex, gender (identity/presentation), and sexual orientation and race, ethnicity and racism.

Reporting on sex and gender	*Use the terms sex (biological attribute) and gender (shaped by social and cultural circumstances) carefully in order to avoid confusing both terms. Indicate if findings apply to only one sex or gender; describe whether sex and gender were considered in study design; whether sex and/or gender was determined based on self-reporting or assigned and methods used. Provide in the source data disaggregated sex and gender data, where this information has been collected, and if consent has been obtained for sharing of individual-level data; provide overall numbers in this Reporting Summary. Please state if this information has not been collected. Report sex- and gender-based analyses where performed, justify reasons for lack of sex- and gender-based analysis.*
Reporting on race, ethnicity, or other socially relevant groupings	*Please specify the socially constructed or socially relevant categorization variable(s) used in your manuscript and explain why they were used. Please note that such variables should not be used as proxies for other socially constructed/relevant variables (for example, race or ethnicity should not be used as a proxy for socioeconomic status). Provide clear definitions of the relevant terms used, how they were provided (by the participants/respondents, the researchers, or third parties), and the method(s) used to classify people into the different categories (e.g. self-report, census or administrative data, social media data, etc.) Please provide details about how you controlled for confounding variables in your analyses.*
Population characteristics	*Describe the covariate-relevant population characteristics of the human research participants (e.g. age, genotypic information, past and current diagnosis and treatment categories). If you filled out the behavioural & social sciences study design questions and have nothing to add here, write "See above."*
Recruitment	*Describe how participants were recruited. Outline any potential self-selection bias or other biases that may be present and how these are likely to impact results.*
Ethics oversight	*Identify the organization(s) that approved the study protocol.*

Note that full information on the approval of the study protocol must also be provided in the manuscript.

Field-specific reporting

Please select the one below that is the best fit for your research. If you are not sure, read the appropriate sections before making your selection.

☒ Life sciences ☐ Behavioural & social sciences ☐ Ecological, evolutionary & environmental sciences

For a reference copy of the document with all sections, see nature.com/documents/nr-reporting-summary-flat.pdf

Life sciences study design

All studies must disclose on these points even when the disclosure is negative.

Sample size	No statistical methods were used to predetermine sample sizes. Instead, sample sizes were chosen based on established standards in the field of chemical biology and drug discovery to ensure biological and technical reproducibility.
Data exclusions	No data was excluded.
Replication	The experimental replicates are stated in the figure legends. Panels/blots showing representative results were repeated independently showing similar results.
Randomization	No randomization was performed, as the study involved controlled in vitro experiments with predefined treatment conditions (e.g., compound dosing in cell lines), where random allocation is not applicable.
Blinding	Blinding was not relevant to this study since the primary endpoints were quantitative and generated by automated instrumentation, which minimizes operator bias. Data analysis was performed using predefined computational pipelines and software, which apply uniform criteria to all samples regardless of their group allocation.

Reporting for specific materials, systems and methods

We require information from authors about some types of materials, experimental systems and methods used in many studies. Here, indicate whether each material, system or method listed is relevant to your study. If you are not sure if a list item applies to your research, read the appropriate section before selecting a response.

Materials & experimental systems

n/a	Involved in the study
☐	☒ Antibodies
☐	☒ Eukaryotic cell lines
☒	☐ Palaeontology and archaeology
☒	☐ Animals and other organisms
☒	☐ Clinical data
☒	☐ Dual use research of concern
☒	☐ Plants

Methods

n/a	Involved in the study
☒	☐ ChIP-seq
☒	☐ Flow cytometry
☒	☐ MRI-based neuroimaging

Antibodies

Antibodies used

Alexa Fluor 680 anti-rabbit IgG (Life Technologies, A21109); Alexa Fluor 750 anti-mouse IgG (Life Technologies, A21037); NUDT5 (Abcam, ab129172); β-Actin (Santa Cruz, sc-47778); Alexa Fluor 750 goat anti-mouse IgG (A21037, Life technologies); goat anti-rabbit IgG (H+L) cross-adsorbed secondary Antibody; Alexa Fluor 647 (A21244, Life technologies); PrimaryNUDT5 (ab129172, Abcam); NUDT5 (sc-398644); β-actin (sc-69879); PPAT (15401-1-AP, Proteintech).

Validation

All antibodies used in this study are commercially available and were validated by the manufacturer including reference list

Eukaryotic cell lines

Policy information about cell lines and Sex and Gender in Research

Cell line source(s)

HEK293, MCF7, A549, HAP1 and HL-60 (from ATCC)

Authentication

All cell lines were used as provided by vendors without further characterisation.

Mycoplasma contamination

Cell lines were tested regulary checked for mycoplasma contamination by standard tests. Tests were negative.

Commonly misidentified lines
(See ICLAC register)

No commonly misidentified cell lines were used in this study.

Plants

Seed stocks

Report on the source of all seed stocks or other plant material used. If applicable, state the seed stock centre and catalogue number. If plant specimens were collected from the field, describe the collection location, date and sampling procedures.

Novel plant genotypes

Describe the methods by which all novel plant genotypes were produced. This includes those generated by transgenic approaches, gene editing, chemical/radiation-based mutagenesis and hybridization. For transgenic lines, describe the transformation method, the number of independent lines analyzed and the generation upon which experiments were performed. For gene-edited lines, describe the editor used, the endogenous sequence targeted for editing, the targeting guide RNA sequence (if applicable) and how the editor was applied.

Authentication

Describe any authentication procedures for each seed stock used or novel genotype generated. Describe any experiments used to assess the effect of a mutation and, where applicable, how potential secondary effects (e.g. second site T-DNA insertions, mosiacism, off-target gene editing) were examined.
