# Supplementary material for: Targeted Protein Degradation of NUDT5 Dissociates Catalytic Inhibition from Protein Loss in 6-Thioguanine Response
Source: Nat Commun. 2026 Jun 30;17:8192. doi: 10.1038/s41467-026-74489-9 (PMC13462928; doi:10.1038/s41467-026-74489-9)
Supplement: Supplementary file 3 [file 41467_2026_74489_MOESM3_ESM.pdf]

nature portfolio

Peer Review File

Targeted Protein Degradation of NUDT5 Dissociates Catalytic Inhibition from Protein Loss in 6-Thioguanine Response

Corresponding Author: Professor Kilian Huber

This manuscript has been previously reviewed at another journal. This document only contains information relating to versions considered at Nature Communications.

This file contains all reviewer reports in order by version, followed by all author rebuttals in order by version.

Version 0:

Reviewer comments:

Reviewer #2

(Remarks to the Author)
The AUthors have directed most of the concerns to the companion manuscript published in Science. While the companion manuscript addresses some concerns, others remain outstanding. Particularly, it's not clear what compensatory mechanisms exist for purine metabolism that are activated upon NUDT5 loss via the degrader toolkit?

Reviewer #3

(Remarks to the Author)
In this study, the authors developed NUDT5-targeted PROTAC and claimed that NUDT5 degrader is more potent than the conventional inhibitor. Despite the satisfying response of the authors, I do not think this study is qualified for Nature Communications. I do not find real breakthrough for PROTAC screening and molecular design. The authors claimed advantage of the PROTAC over inhibitor, however, there is no direct comparison was provided. Therefore, I recommend more specific journal for this study.

Open Access This Peer Review File is licensed under a Creative Commons Attribution 4.0 International License, which permits use, sharing, adaptation, distribution and reproduction in any medium or format, as long as you give appropriate credit to the original author(s) and the source, provide a link to the Creative Commons license, and indicate if changes were made.
In cases where reviewers are anonymous, credit should be given to 'Anonymous Referee' and the source.
The images or other third party material in this Peer Review File are included in the article's Creative Commons license, unless indicated otherwise in a credit line to the material. If material is not included in the article's Creative Commons license and your intended use is not permitted by statutory regulation or exceeds the permitted use, you will need to obtain permission directly from the copyright holder.
To view a copy of this license, visit https://creativecommons.org/licenses/by/4.0/

Response to reviewers

Reviewer #2 (Remarks to the Author):

The AUthors have directed most of the concerns to the companion manuscript published in Science. While the companion manuscript addresses some concerns, others remain outstanding. Particularly, it's not clear what compensatory mechanisms exist for purine metabolism that are activated upon NUDT5 loss via the degrader toolkit?

We thank the reviewer for highlighting the importance of understanding compensatory mechanisms in purine metabolism following targeted degradation of NUDT5. To address this, we have expanded our bioinformatic analyses of our dNUDT5 proteomic datasets. The results indicate minimal global changes in purine biosynthesis enzyme abundance upon NUDT5 loss, suggesting that regulation occurs at the level of pathway activity rather than protein expression.

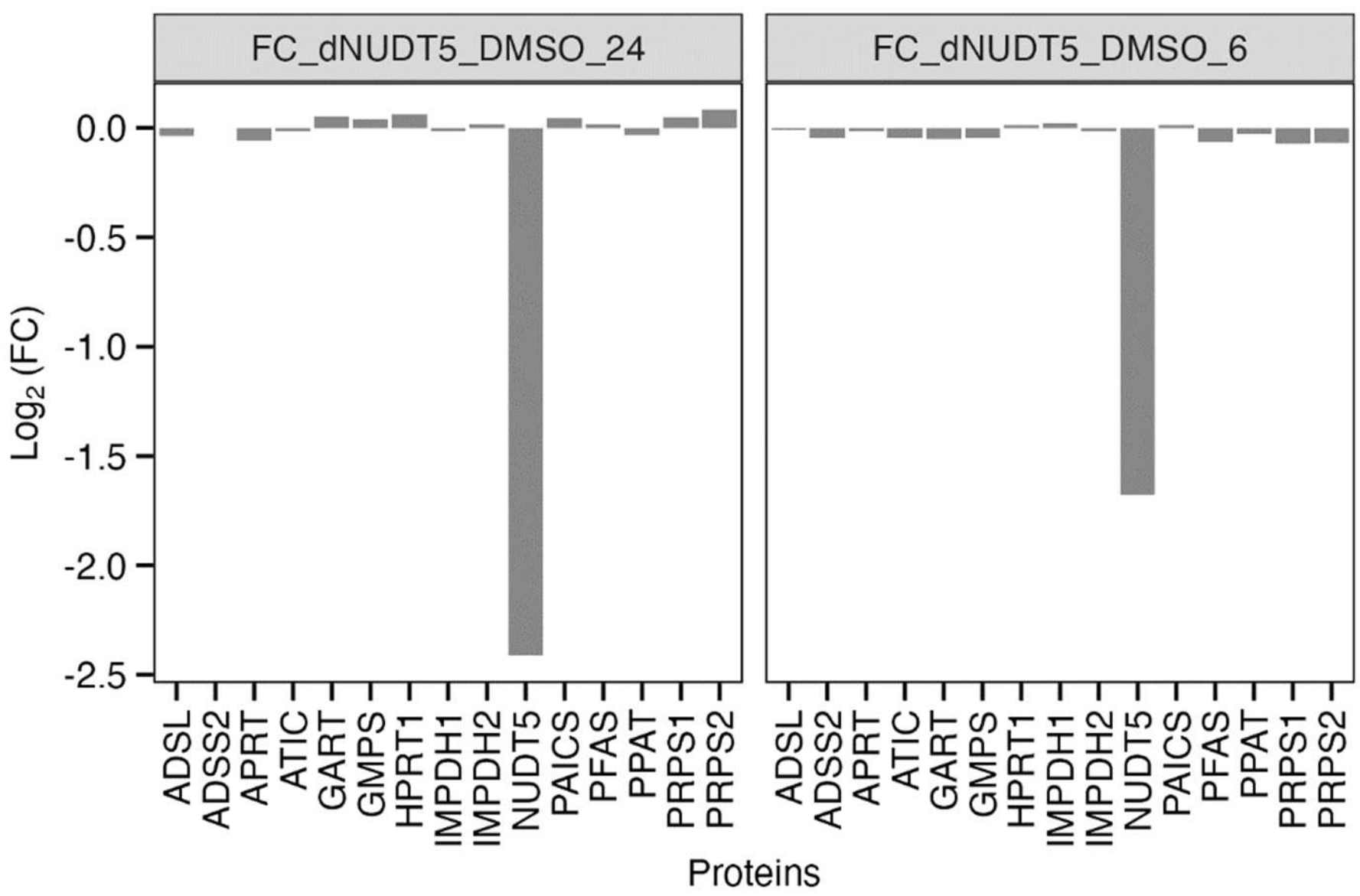

Consistent with this, global analysis across cancer cell lines using CCLE data shows that expression of purine biosynthesis enzymes is largely independent of NUDT5 abundance.

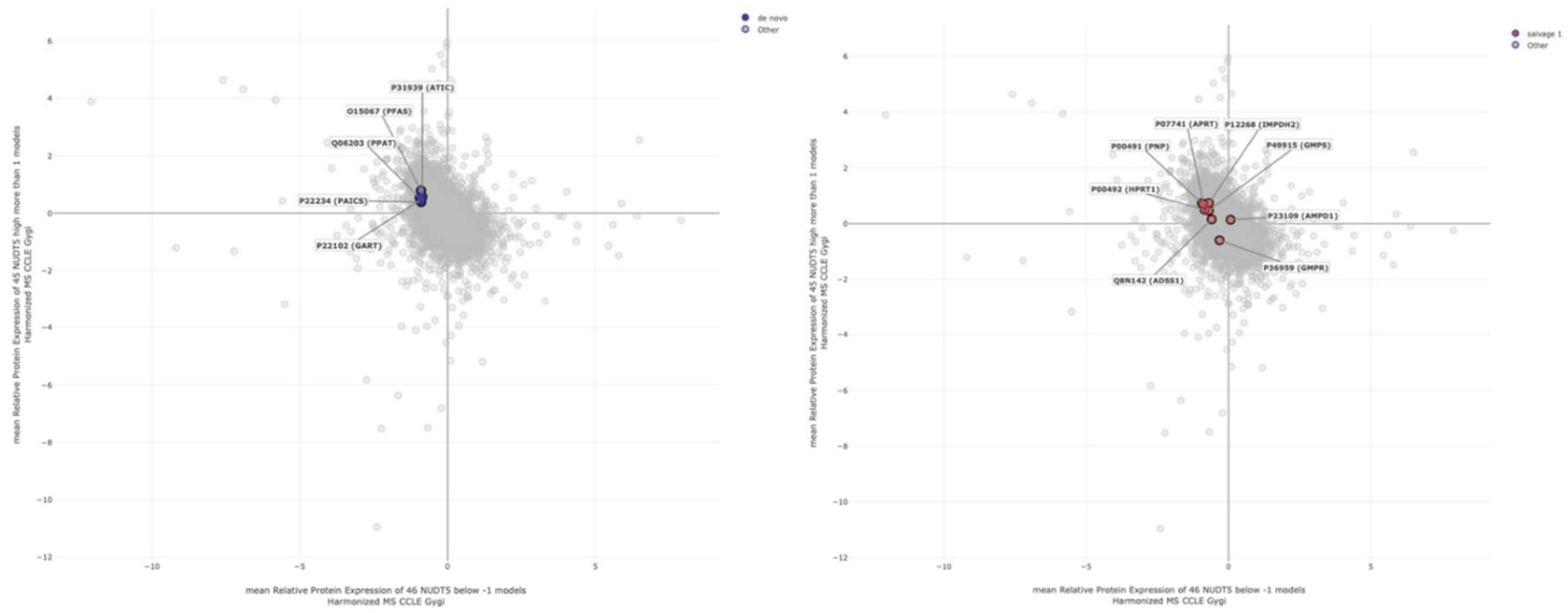

Analysis of CCLE data indicates protein levels of purine *de novo* and salvage pathway enzymes are independent of NUDT5 expression levels.

Together, these observations argue against a compensatory mechanism driven by broad reprogramming at the level of enzyme expression. At the same time, we agree that the present data do not allow us to distinguish definitively among all possible regulatory mechanisms underlying the compensatory phenotype. Rather, the results are most consistent with regulation occurring at the level of pathway activity rather than pathway enzyme abundance. This could plausibly involve altered enzyme activity, changes in metabolite allocation, altered pathway organization, or other post-translational or allosteric regulatory effects. Accordingly, although the added proteomic and expression analyses help exclude broad enzyme abundance remodelling as the principal compensatory response, they do not on their own identify a single exclusive regulatory mechanism.

<u>Reviewer #3 (Remarks to the Author):</u>

In this study, the authors developed NUDT5-targeted PROTAC and claimed that NUDT5 degrader is more potent than the conventional inhibitor. Despite the satisfying response of the authors, I do not think this study is qualified for Nature Communications. I do not find real breakthrough for PROTAC screening and molecular design. The authors claimed advantage of the PROTAC over inhibitor, however, there is no direct comparison was provided. Therefore, I recommend more specific journal for this study.

We appreciate the reviewer's request for a direct comparison between catalytic inhibition and targeted degradation. We agree that this is critical for substantiating the central claim of the study and have prepared the below head-to-head comparisons to clarify the dramatic differences between NUDT5 degraders and enzymatic inhibitors. These data establish a clear

functional dissociation between catalytic inhibition and protein depletion and directly support the conclusion that NUDT5's role in this context cannot be explained by enzymatic activity alone.

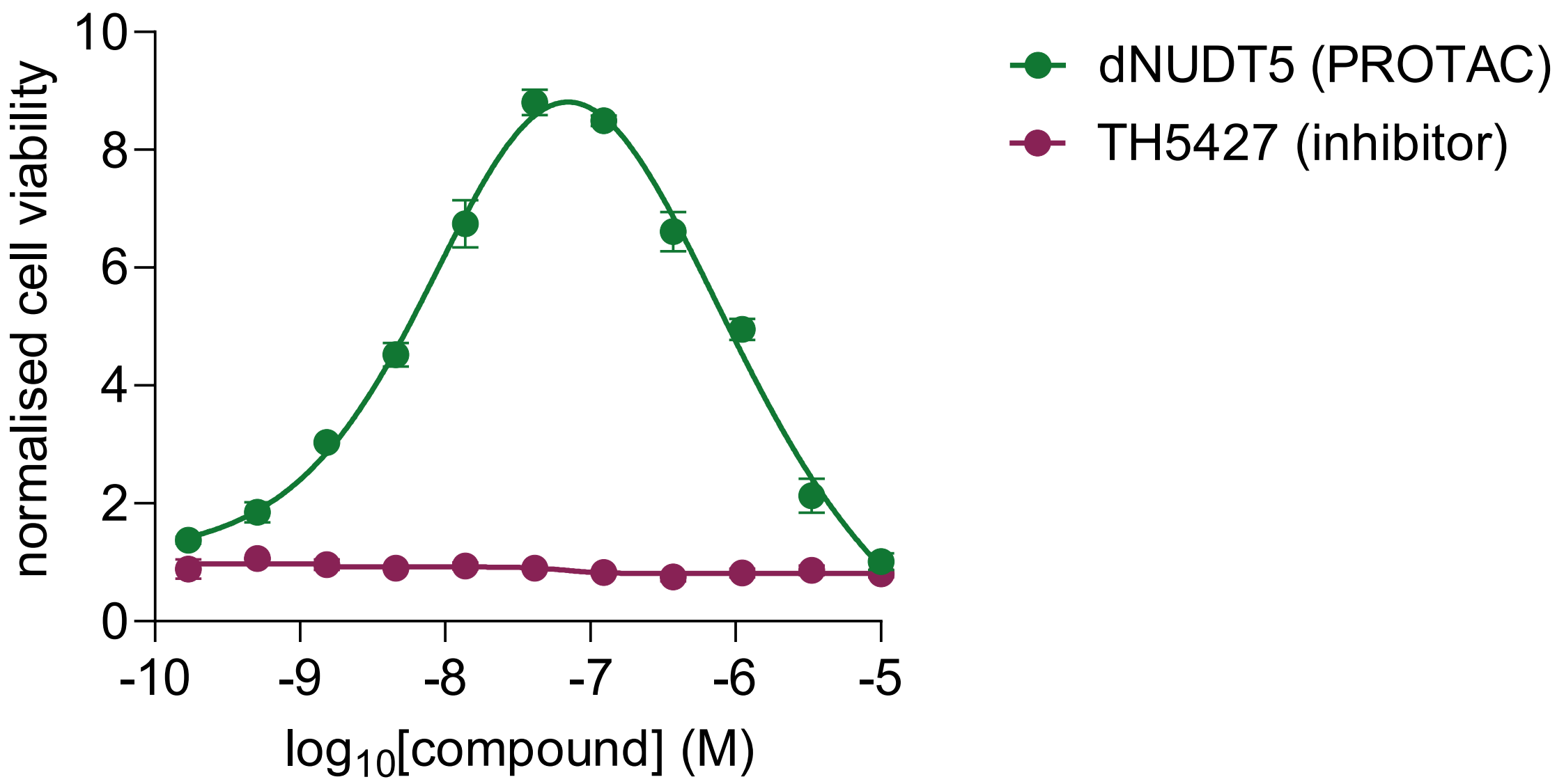

Targeted degradation of NUDT5 by the PROTAC dNUDT5 rescues sensitivity of cancer cells toward 6-TG in a dose-dependent manner mirroring the effect on NUDT5 protein levels whilst the catalytic inhibitor TH5427 exhibits no significant effect.

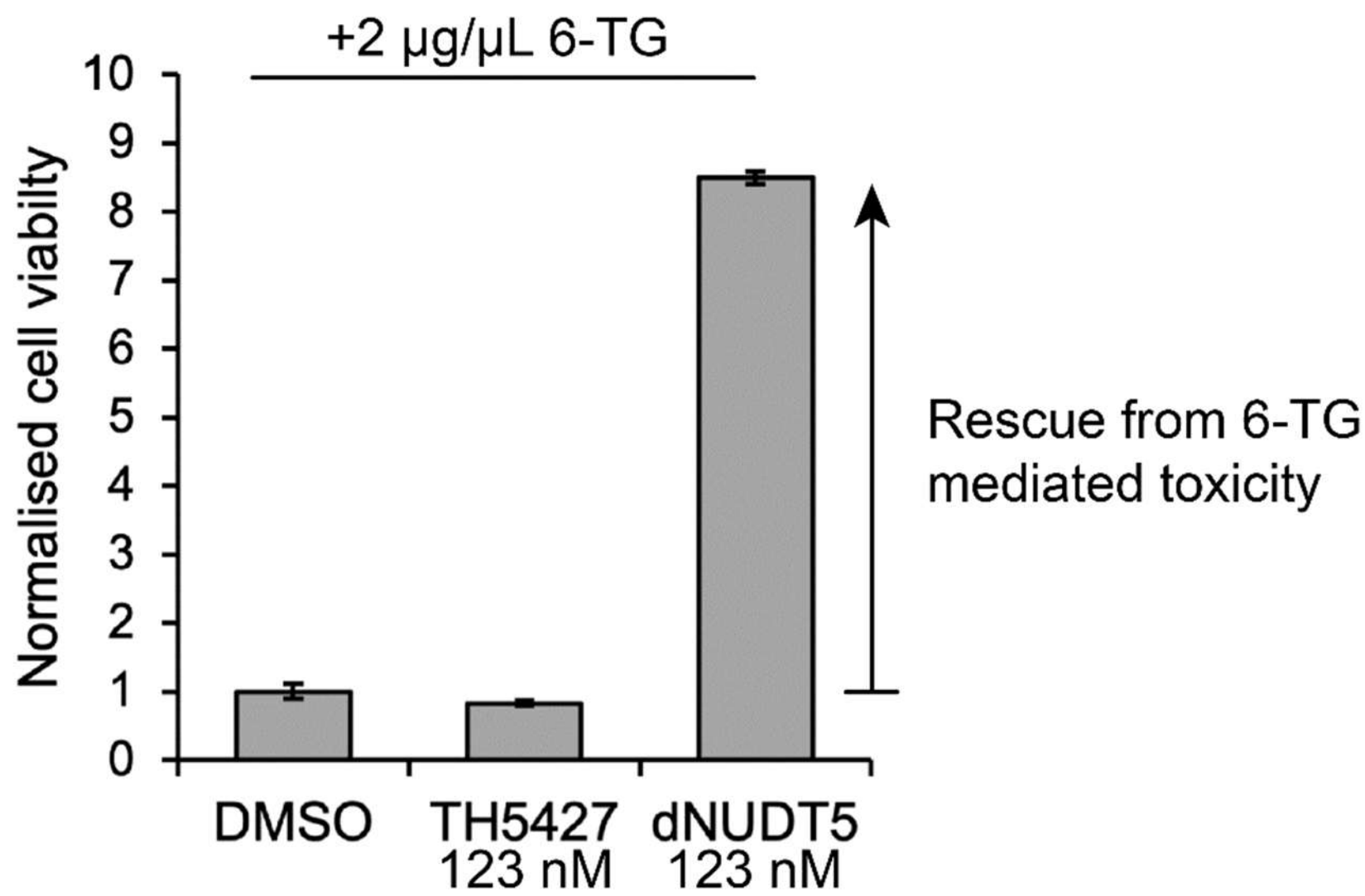

Targeted degradation of NUDT5 by the PROTAC dNUDT5 boosts cancer cell viability more than 8-fold in presence of 6-TG compared to the catalytic inhibitor TH5427 and DMSO control.
