# Supplementary material for: Targeted Protein Degradation of NUDT5 Dissociates Catalytic Inhibition from Protein Loss in 6-Thioguanine Response
Source: Nat Commun. 2026 Jun 30;17:8192. doi: 10.1038/s41467-026-74489-9 (PMC13462928; doi:10.1038/s41467-026-74489-9)
Supplement: Supplementary file 4 [file 41467_2026_74489_MOESM4_ESM.zip › NatComms-26-013996A_uncropped_blots.pdf]

Figure 2E (left)

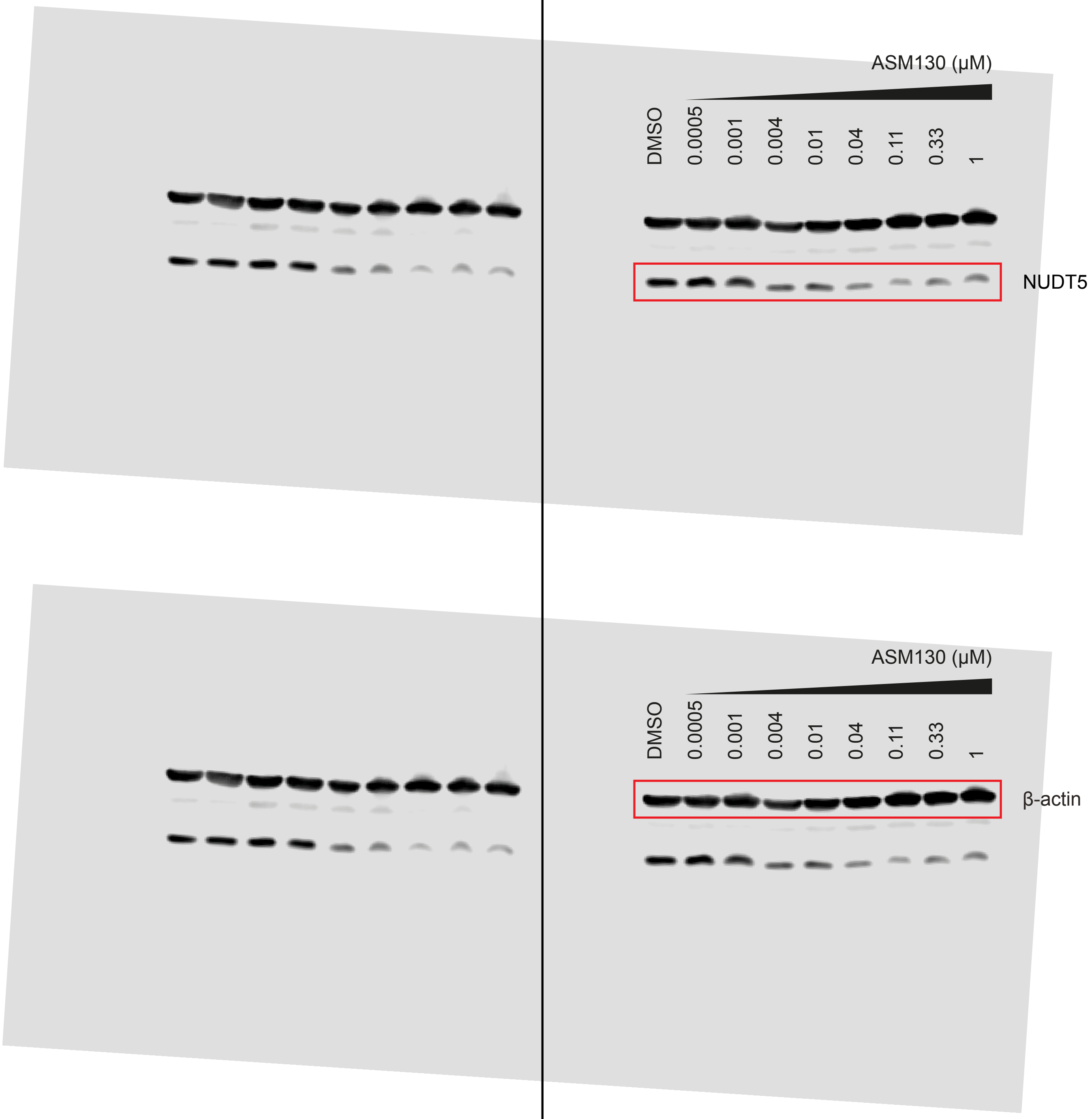

Figure 2E (right)

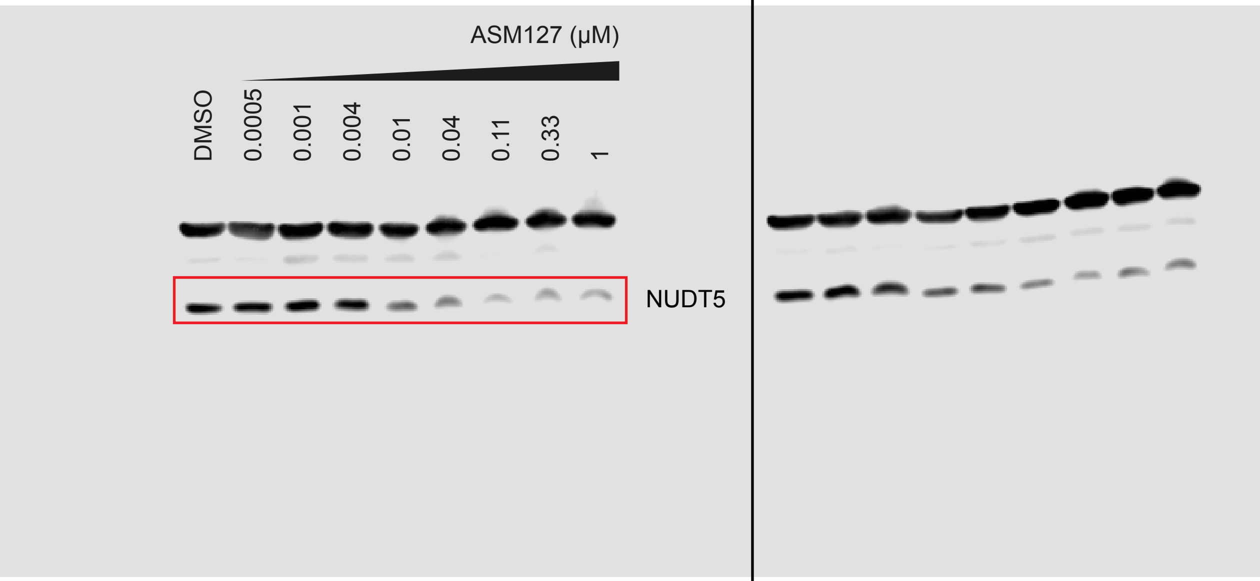

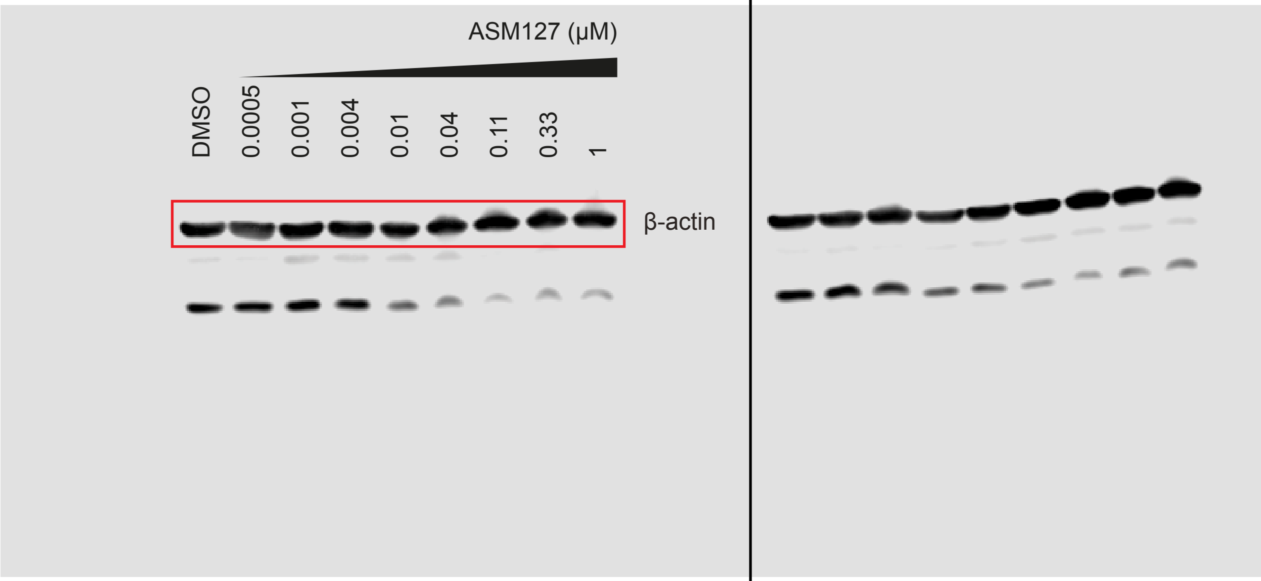

Figure 2G (left)

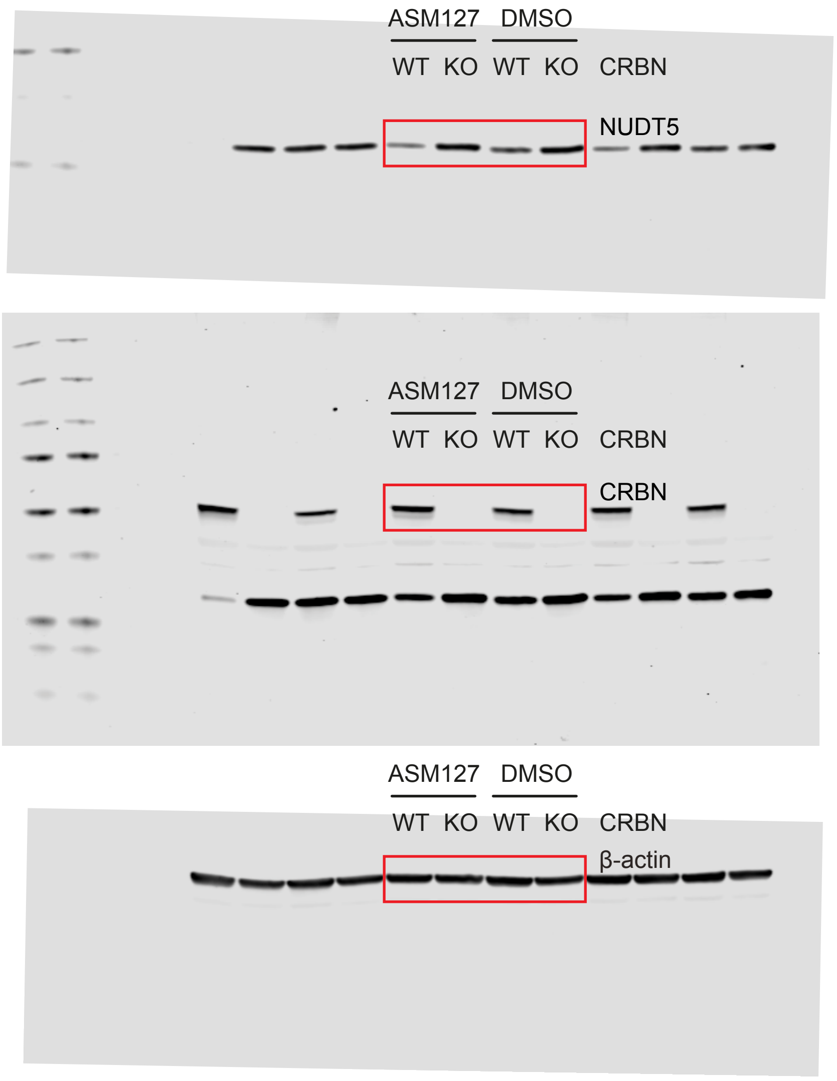

Figure 2G (right)

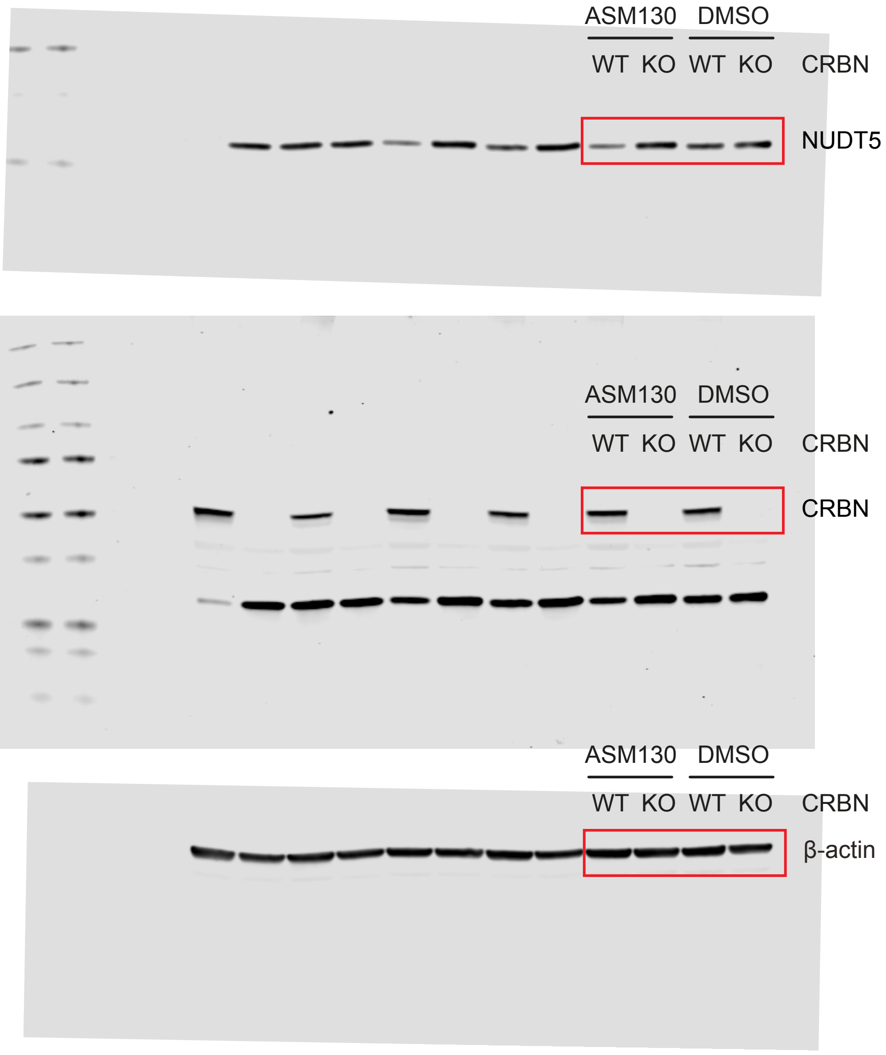

Figure 3B

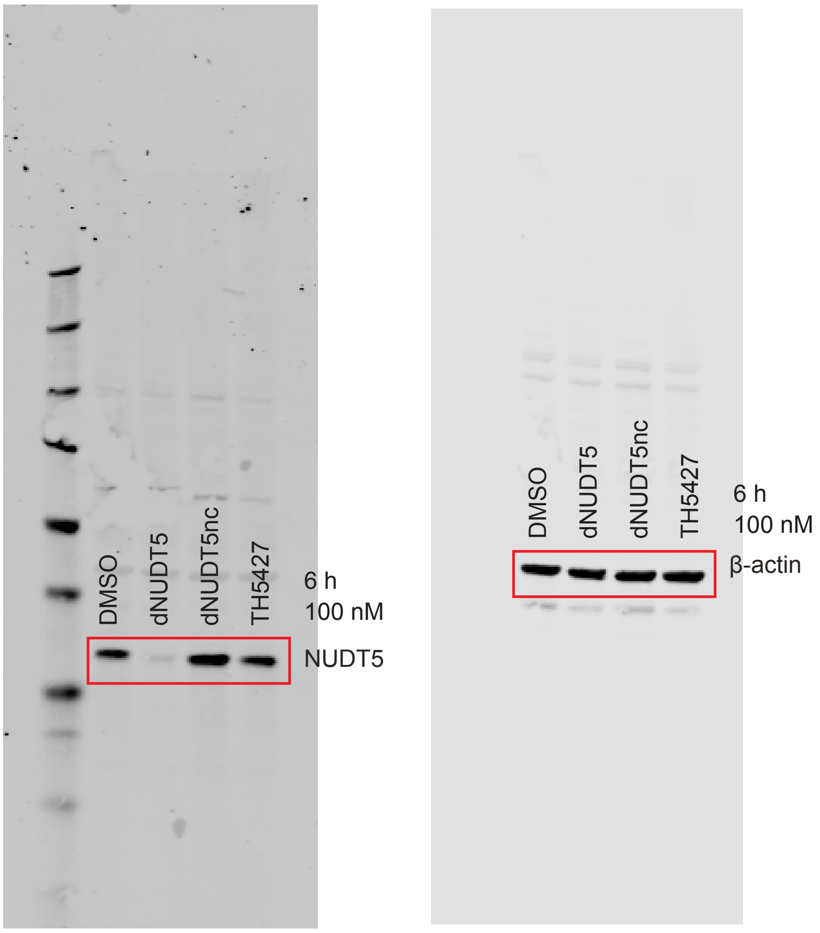

Figure 3D

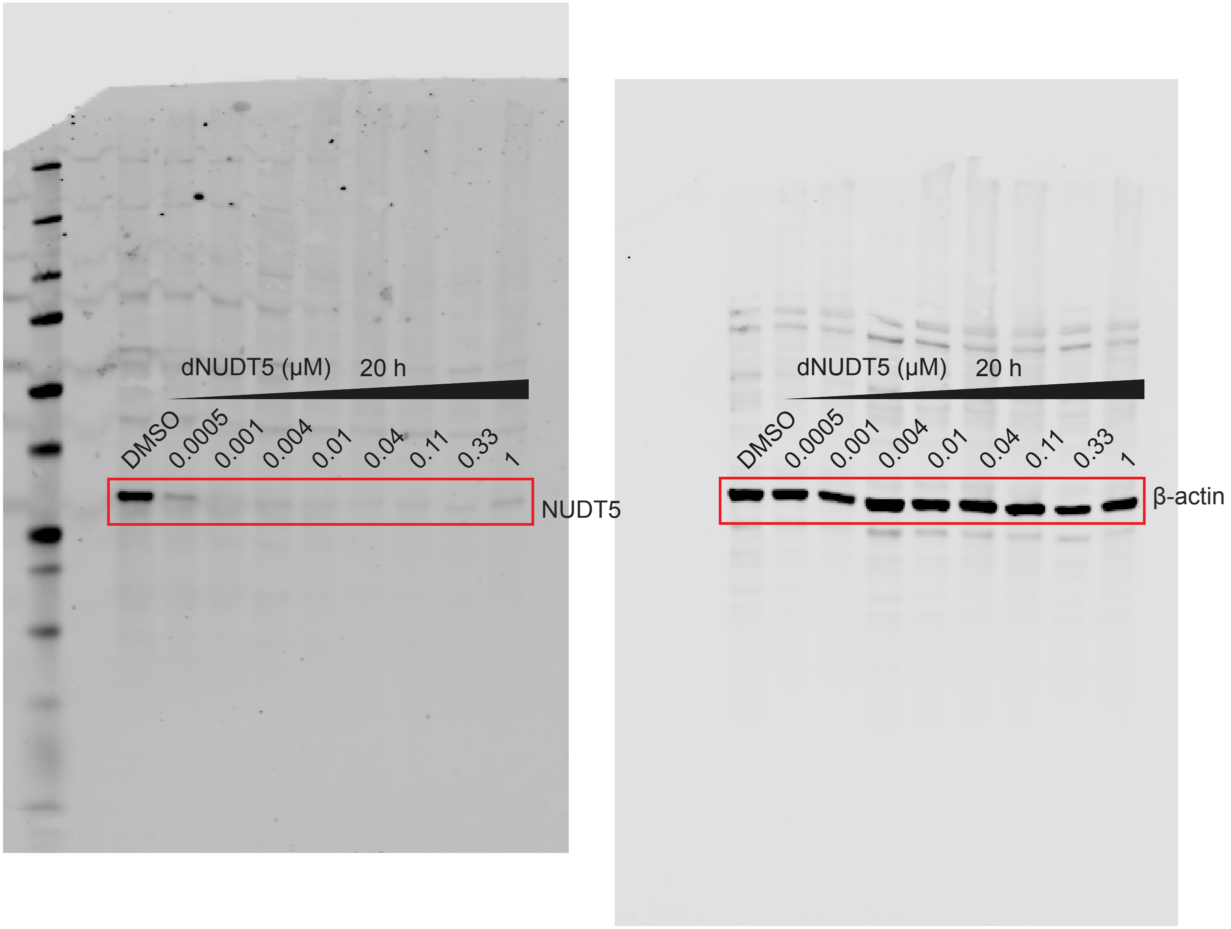

Figure 3E

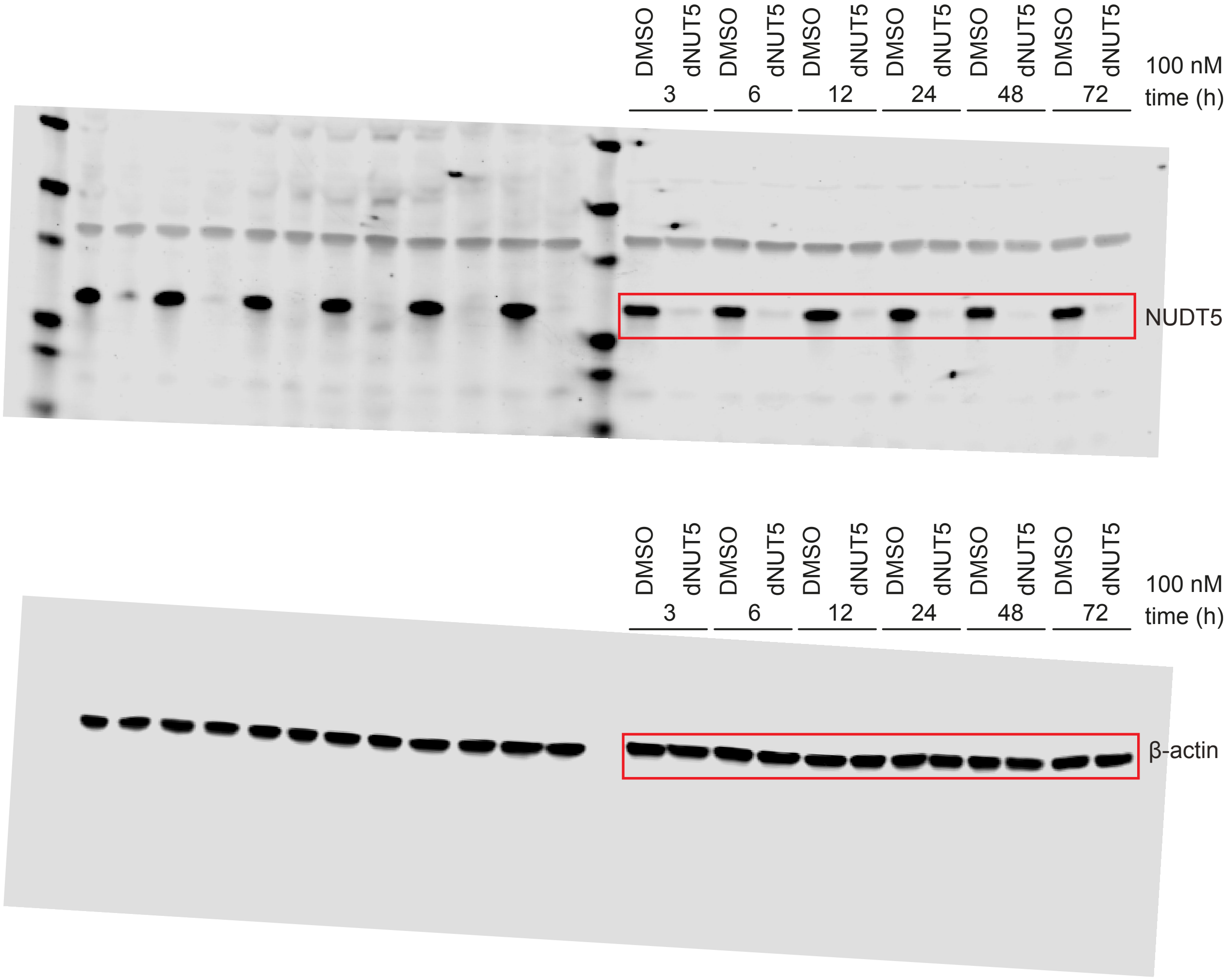
DMSO
dNUT5
DMSO
dNUT5
DMSO
dNUT5
DMSO
dNUT5
DMSO
dNUT5
DMSO
dNUT5
100 nM
3
6
12
24
48
72
time (h)
NUDT5
DMSO
dNUT5
DMSO
dNUT5
DMSO
dNUT5
DMSO
dNUT5
DMSO
dNUT5
DMSO
dNUT5
100 nM
3
6
12
24
48
72
time (h)
β-actin

Figure S2

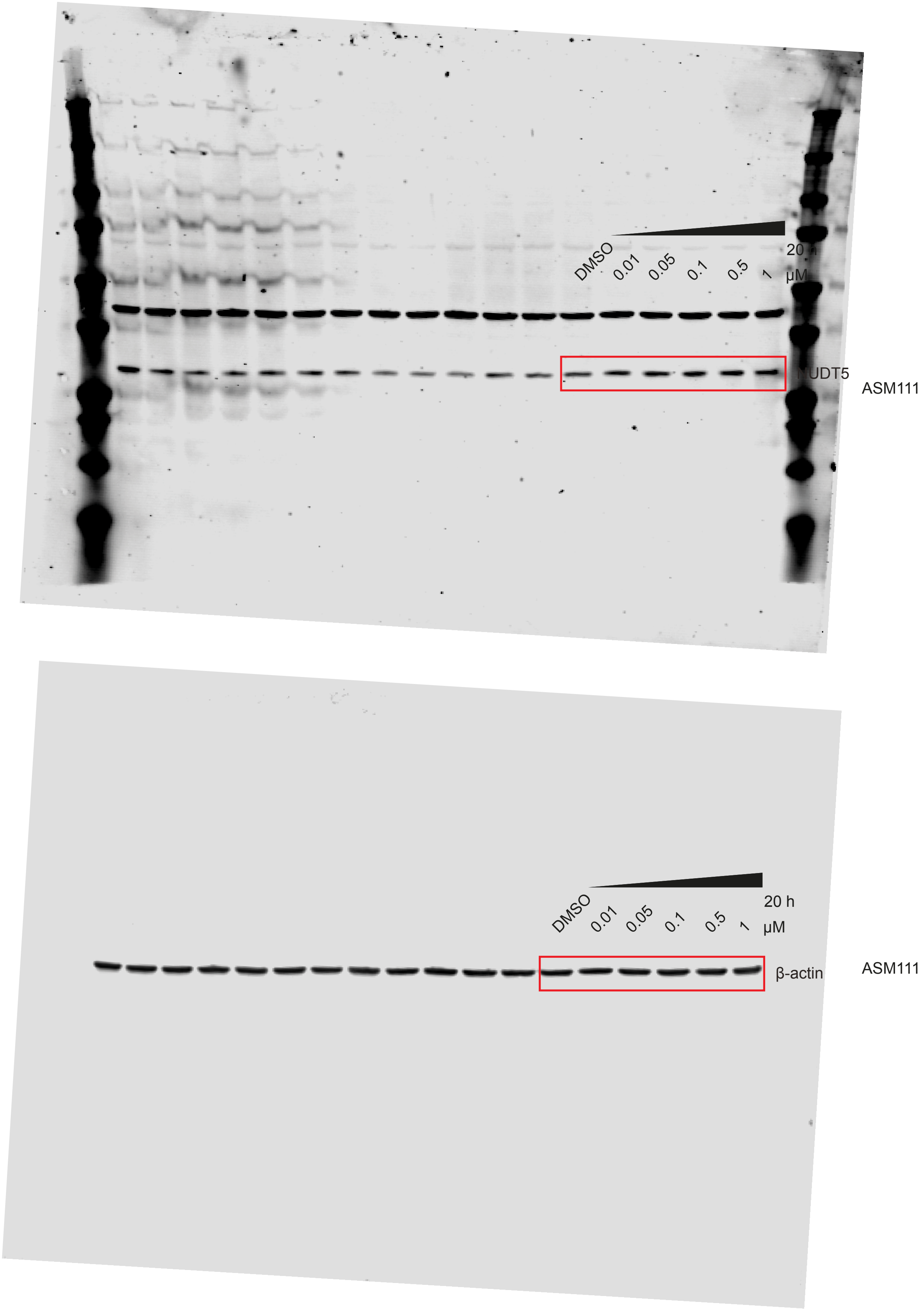
DMSO
0.01
0.05
0.1
0.5
1
20
μM
UDT5
ASM111
DMSO
0.01
0.05
0.1
0.5
1
20 h
μM
β-actin
ASM111

Figure S2

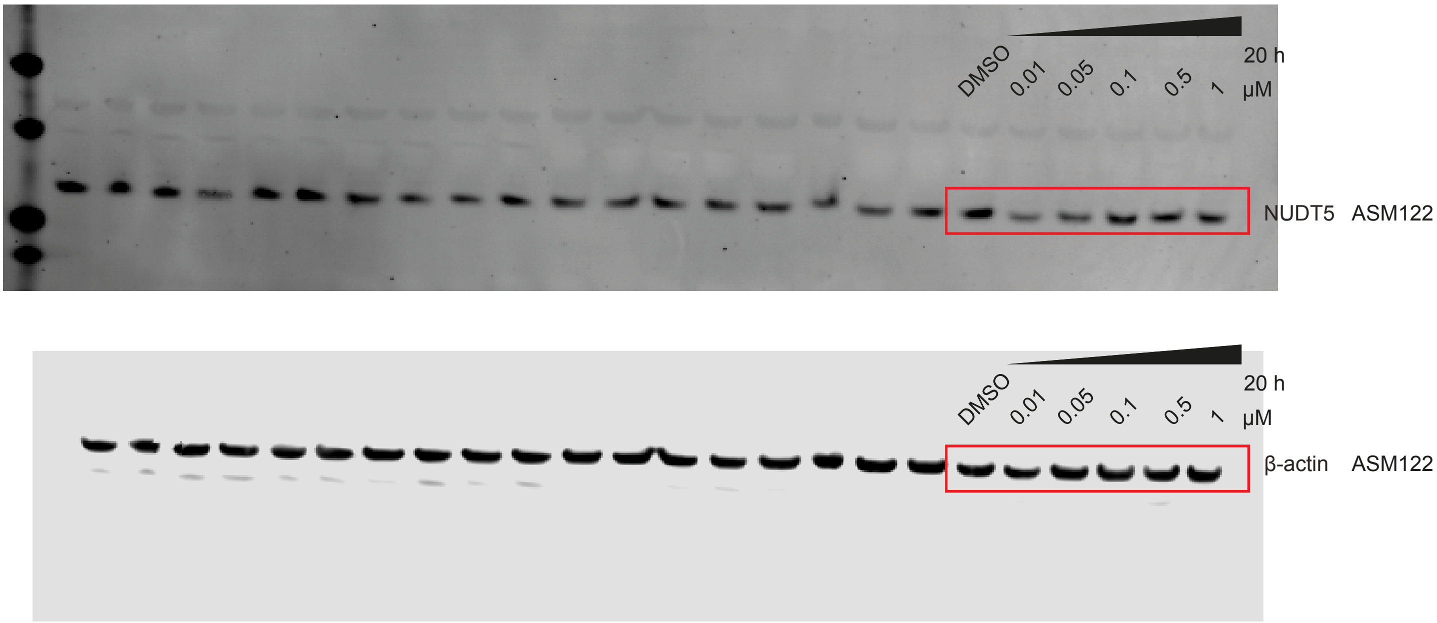

Figure S2

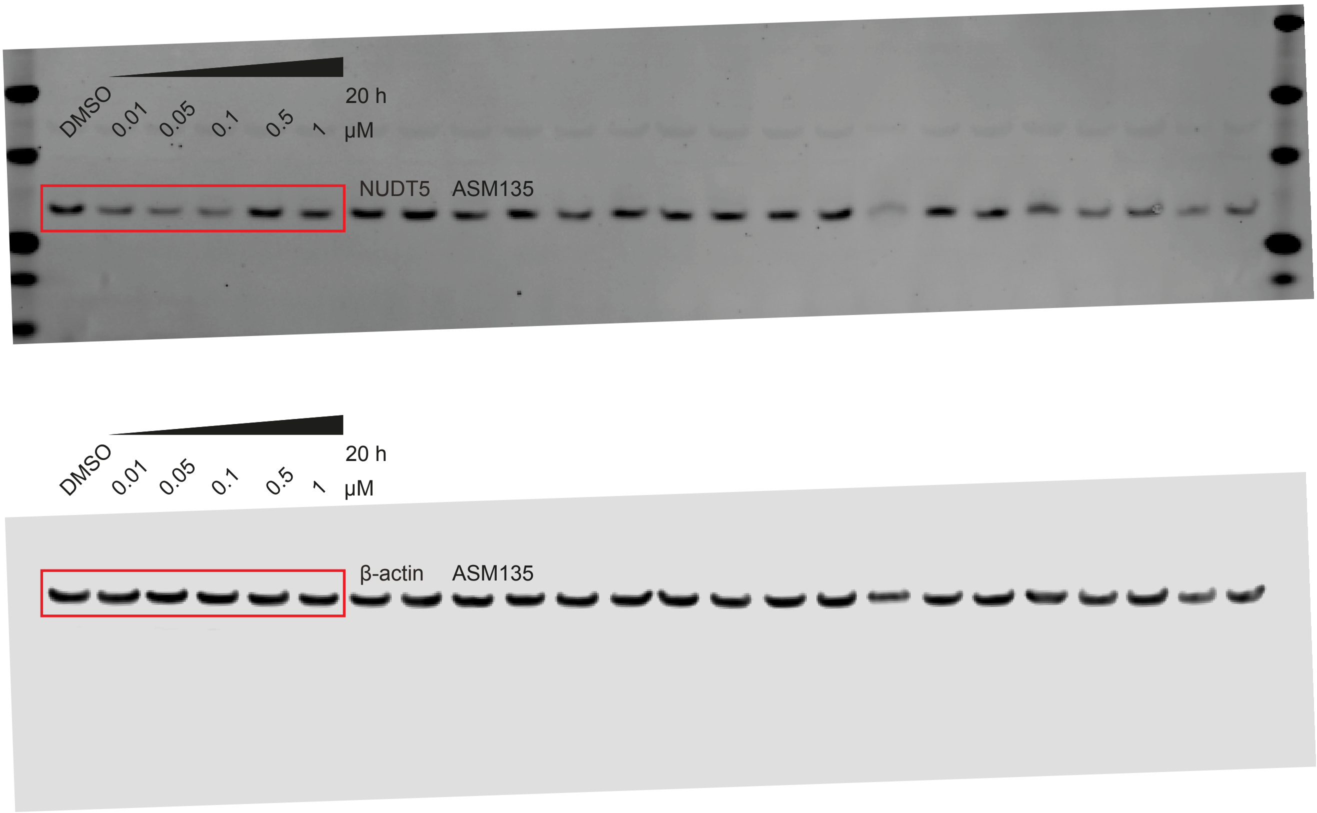

Figure S2

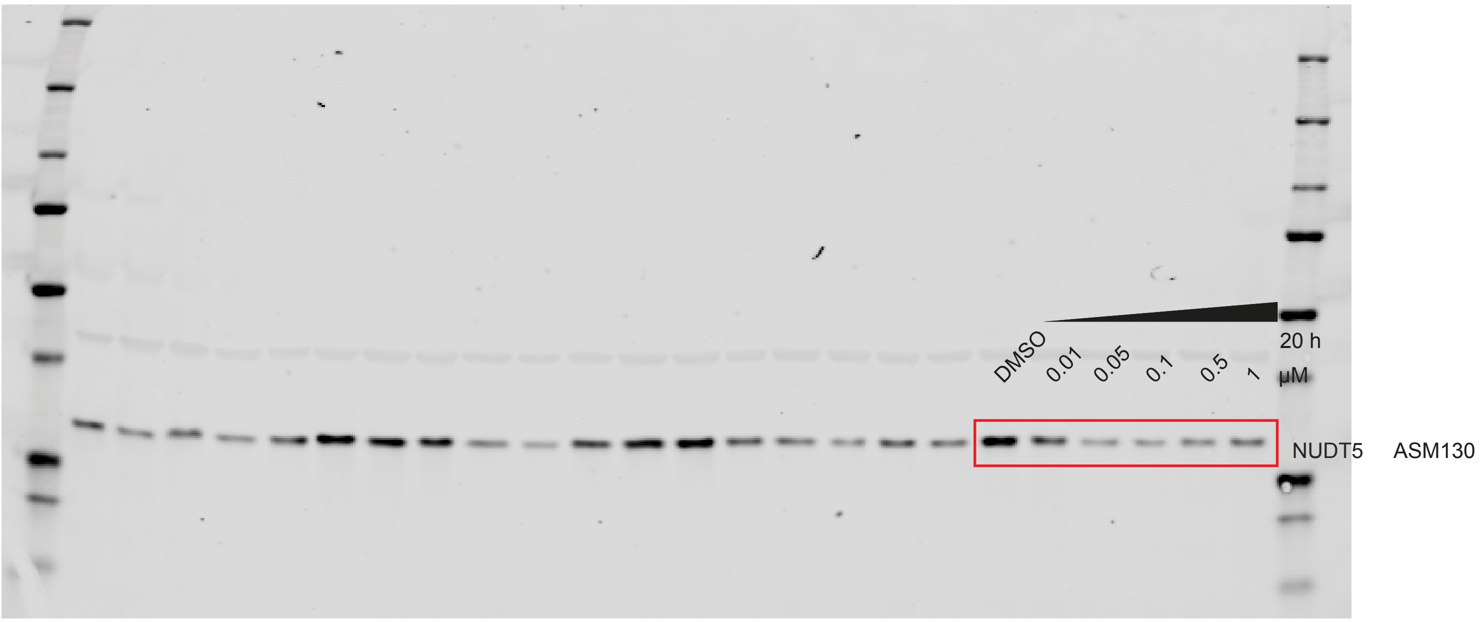
DMSO
0.01
0.05
0.1
0.5
1
20 h
μM
NUDT5
ASM130

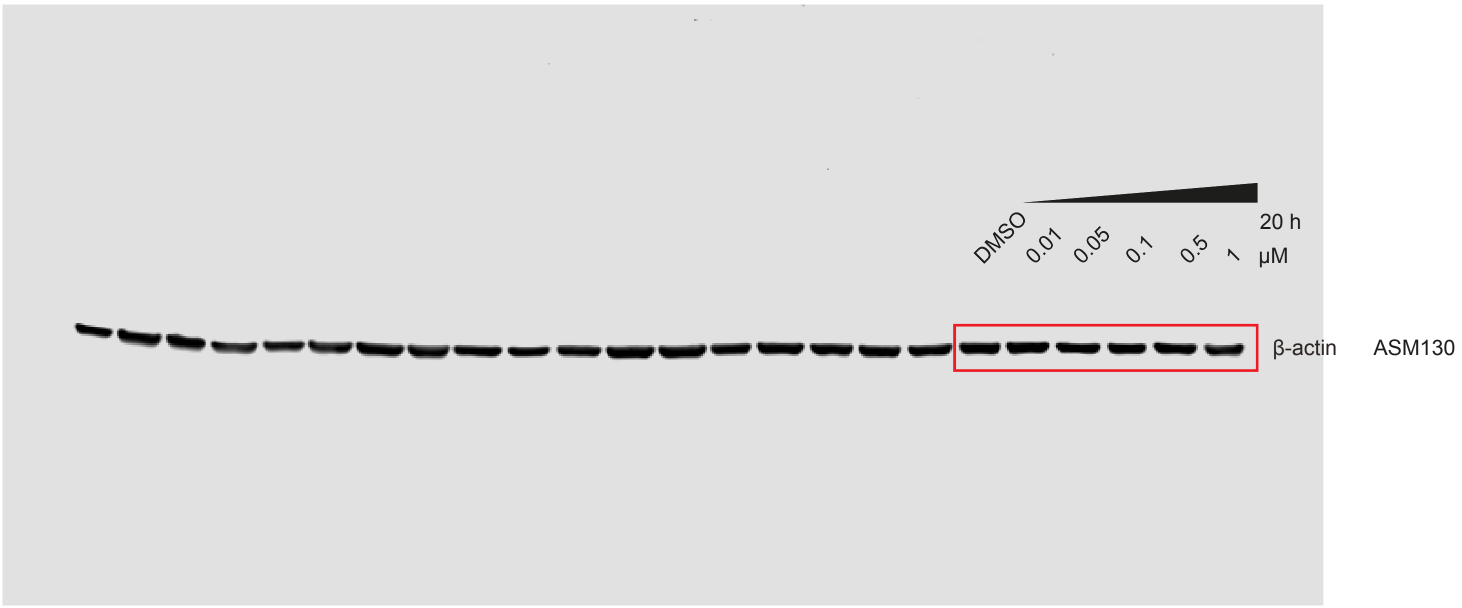
DMSO
0.01
0.05
0.1
0.5
1
20 h
μM
β-actin
ASM130

Figure S2

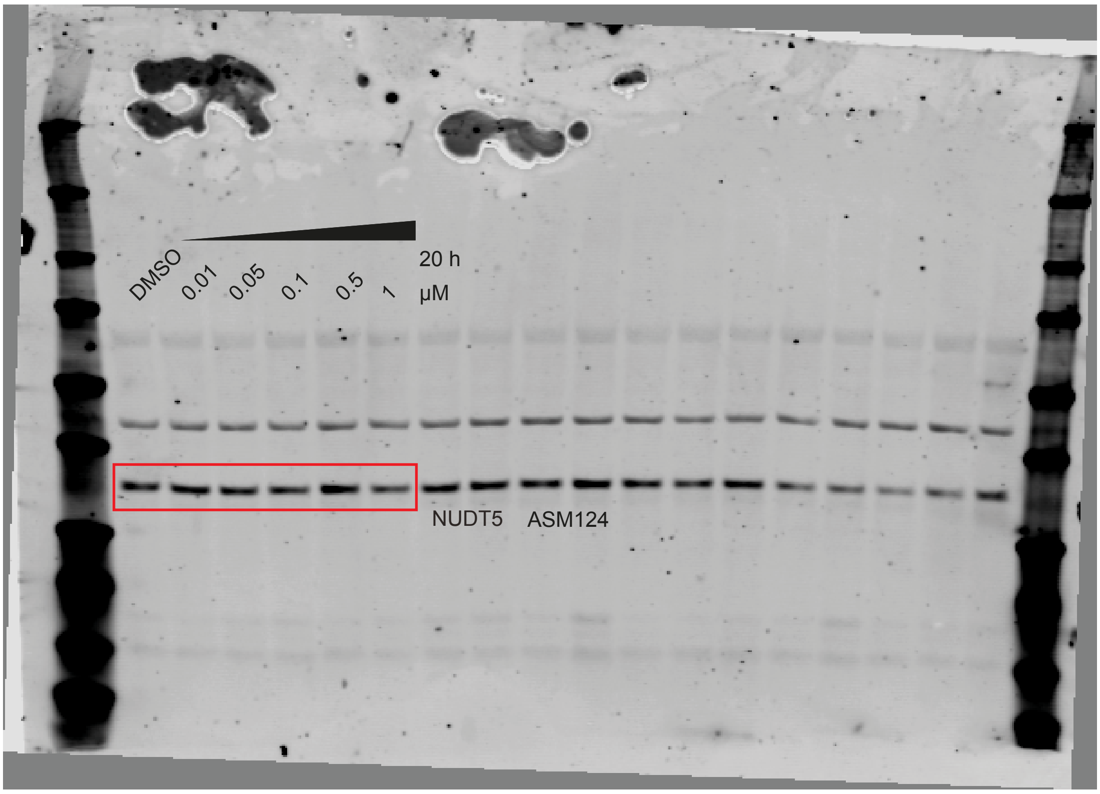

DMSO
0.01
0.05
0.1
0.5
1
20 h
µM
NUDT5
ASM124

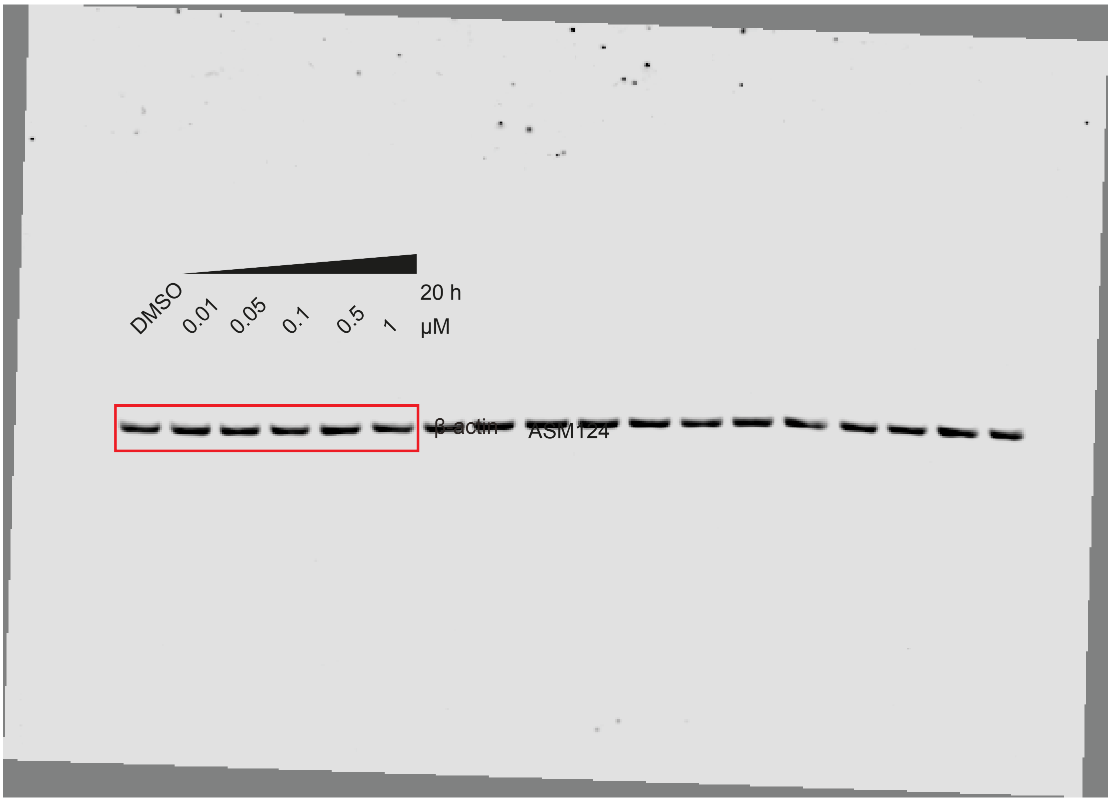

DMSO
0.01
0.05
0.1
0.5
1
20 h
µM
β-actin
ASM124

Figure S2

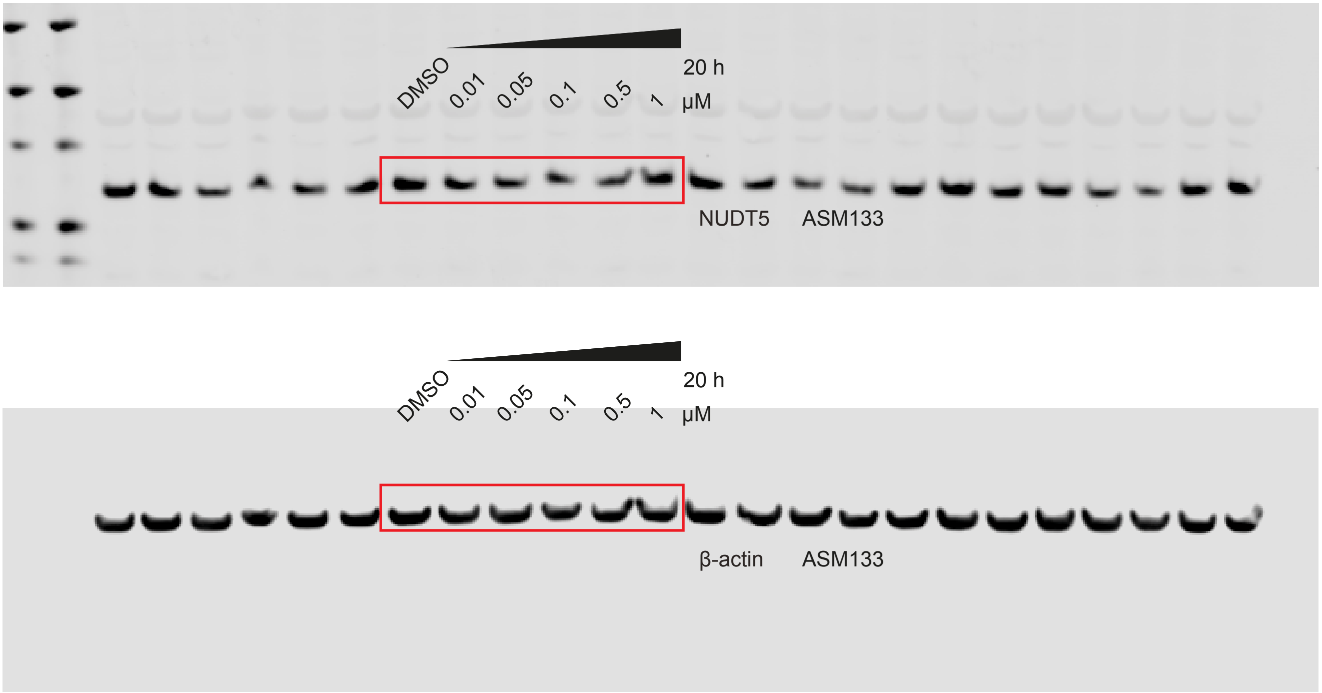

Figure S2

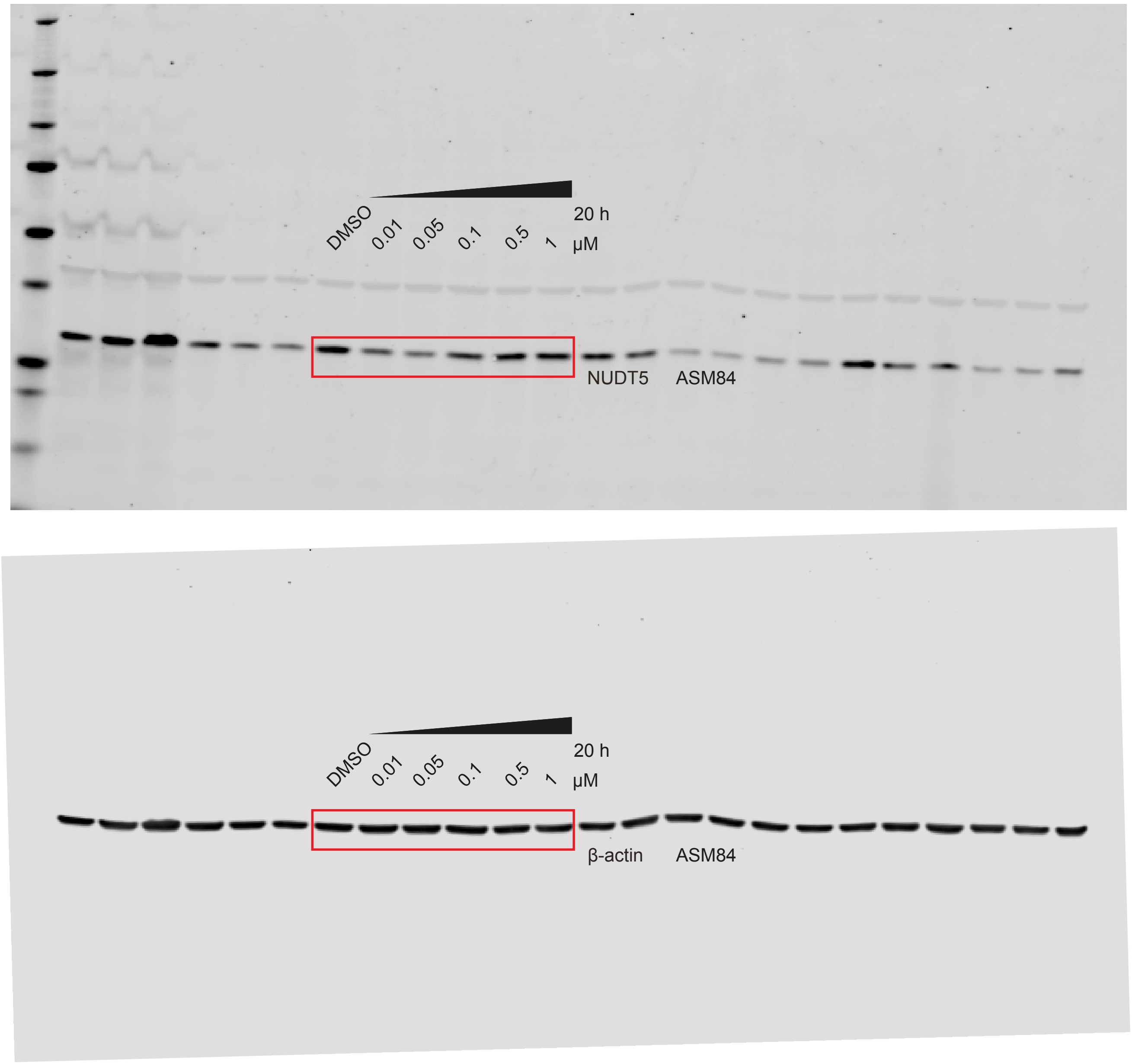

Figure S2

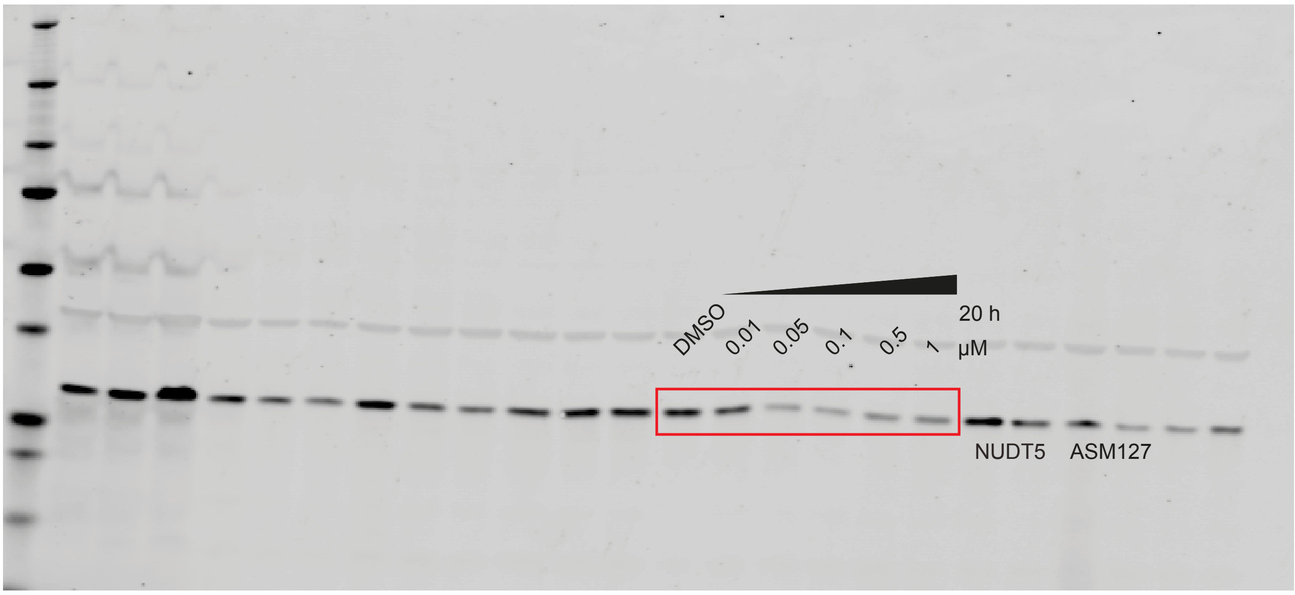
DMSO
0.01
0.05
0.1
0.5
1
20 h
µM
NUDT5
ASM127

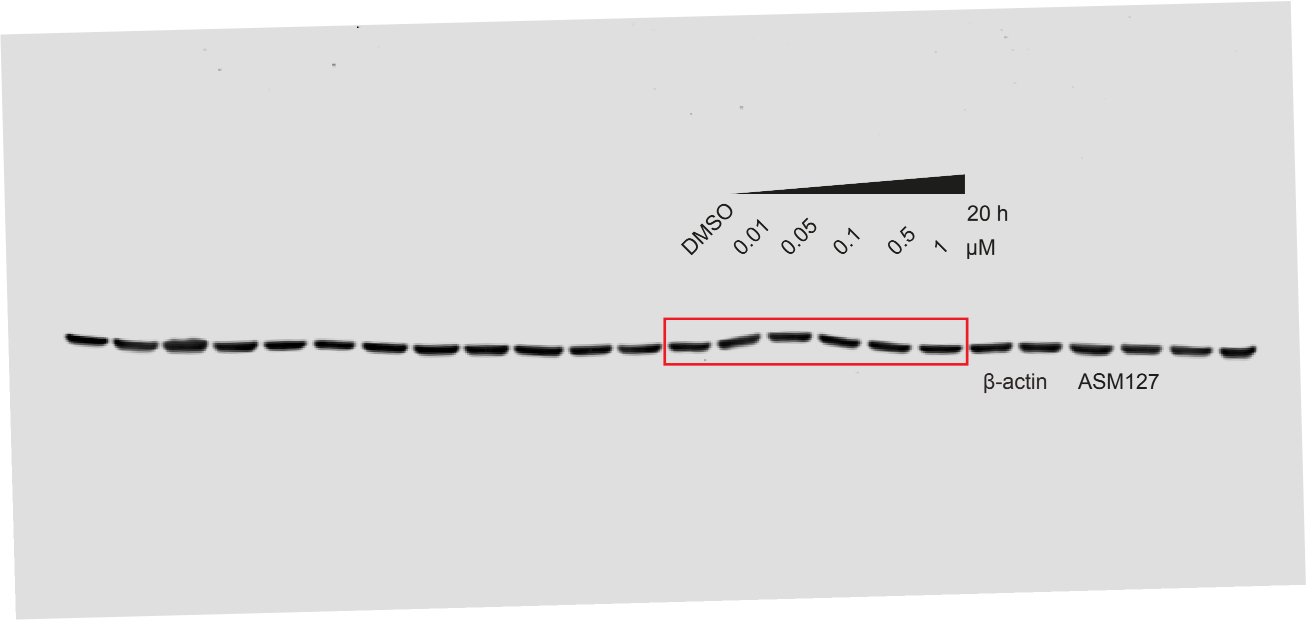
DMSO
0.01
0.05
0.1
0.5
1
20 h
µM
β-actin
ASM127

Figure S2

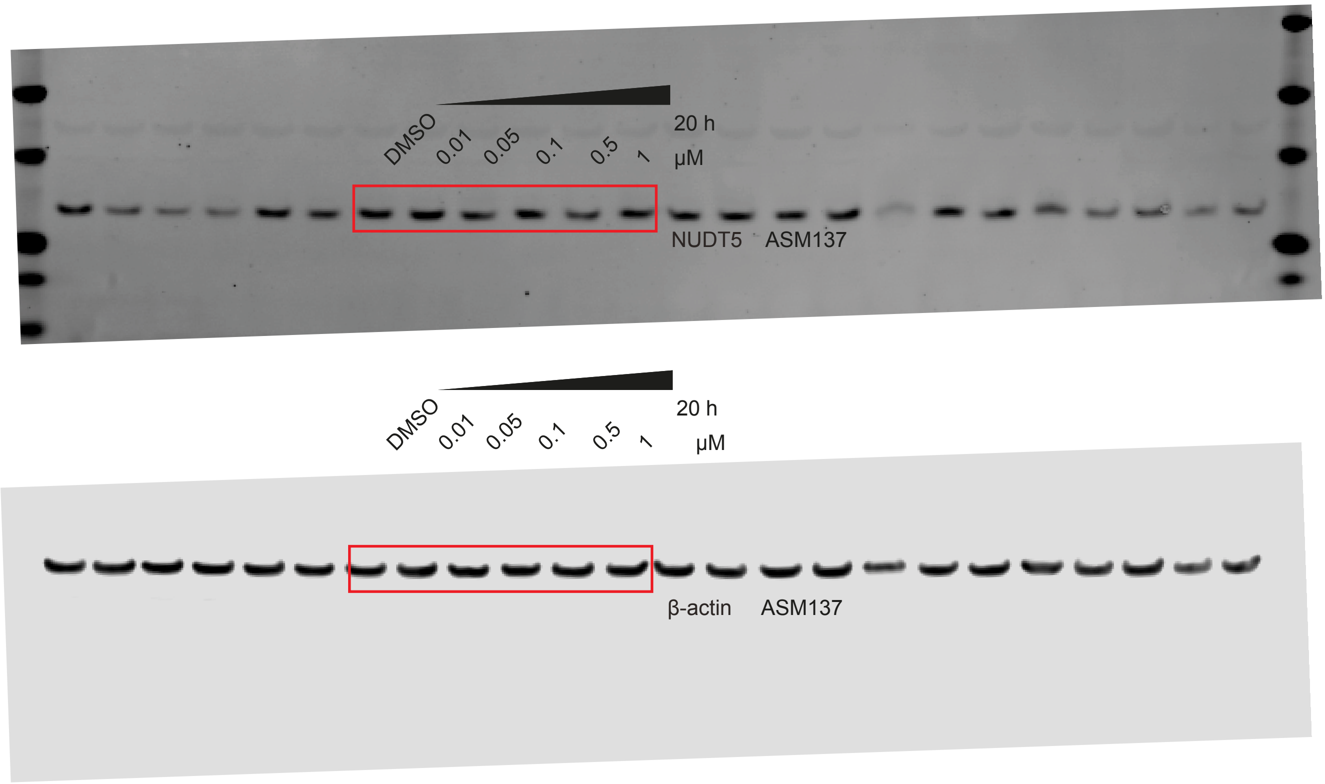
20 h
DMSO
0.01
0.05
0.1
0.5
1
μM
NUDT5
ASM137
20 h
DMSO
0.01
0.05
0.1
0.5
1
μM
β-actin
ASM137

Figure S2

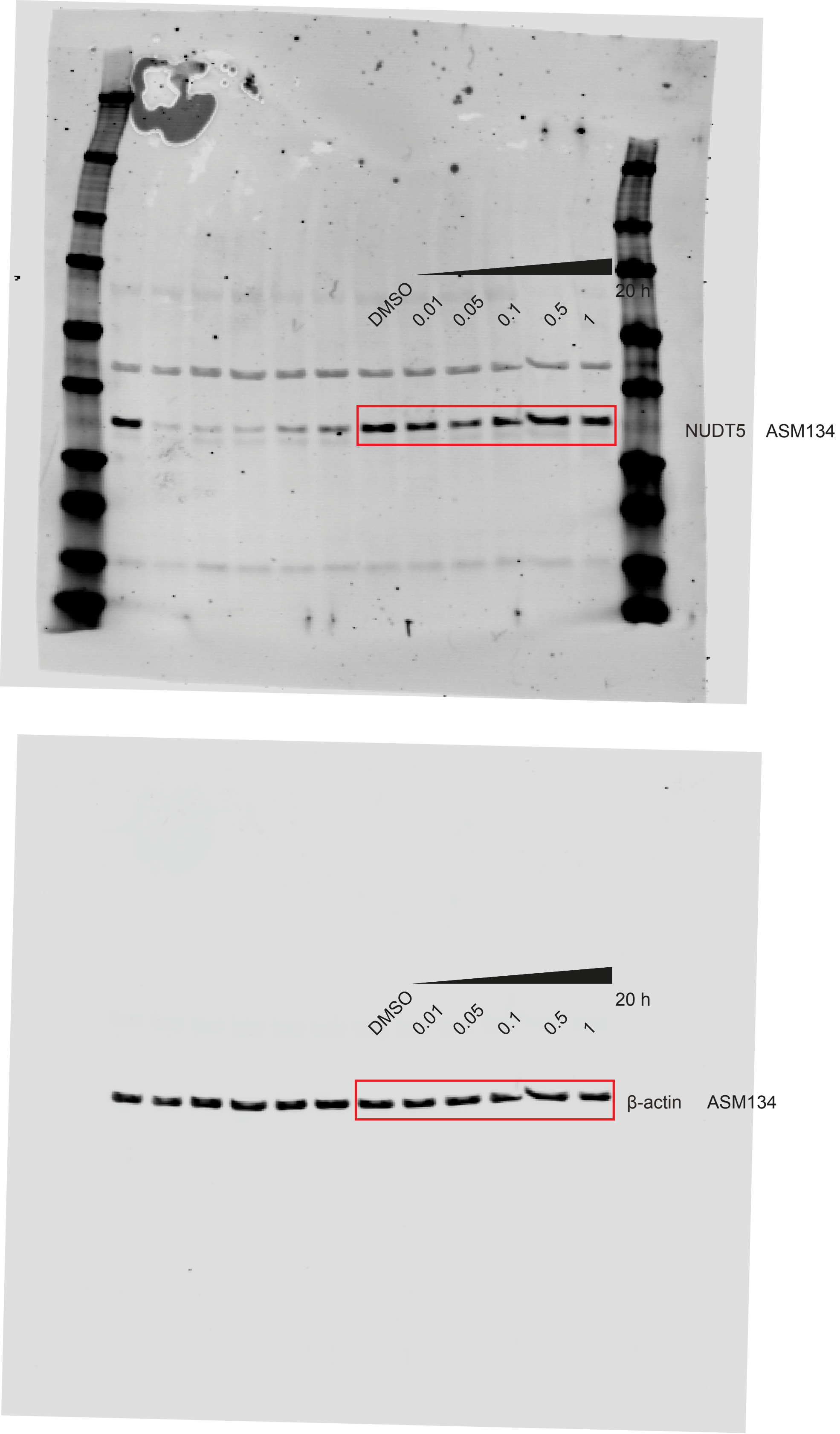
20 h
DMSO
0.01
0.05
0.1
0.5
1
NUDT5
ASM134
20 h
DMSO
0.01
0.05
0.1
0.5
1
β-actin
ASM134

Figure S2

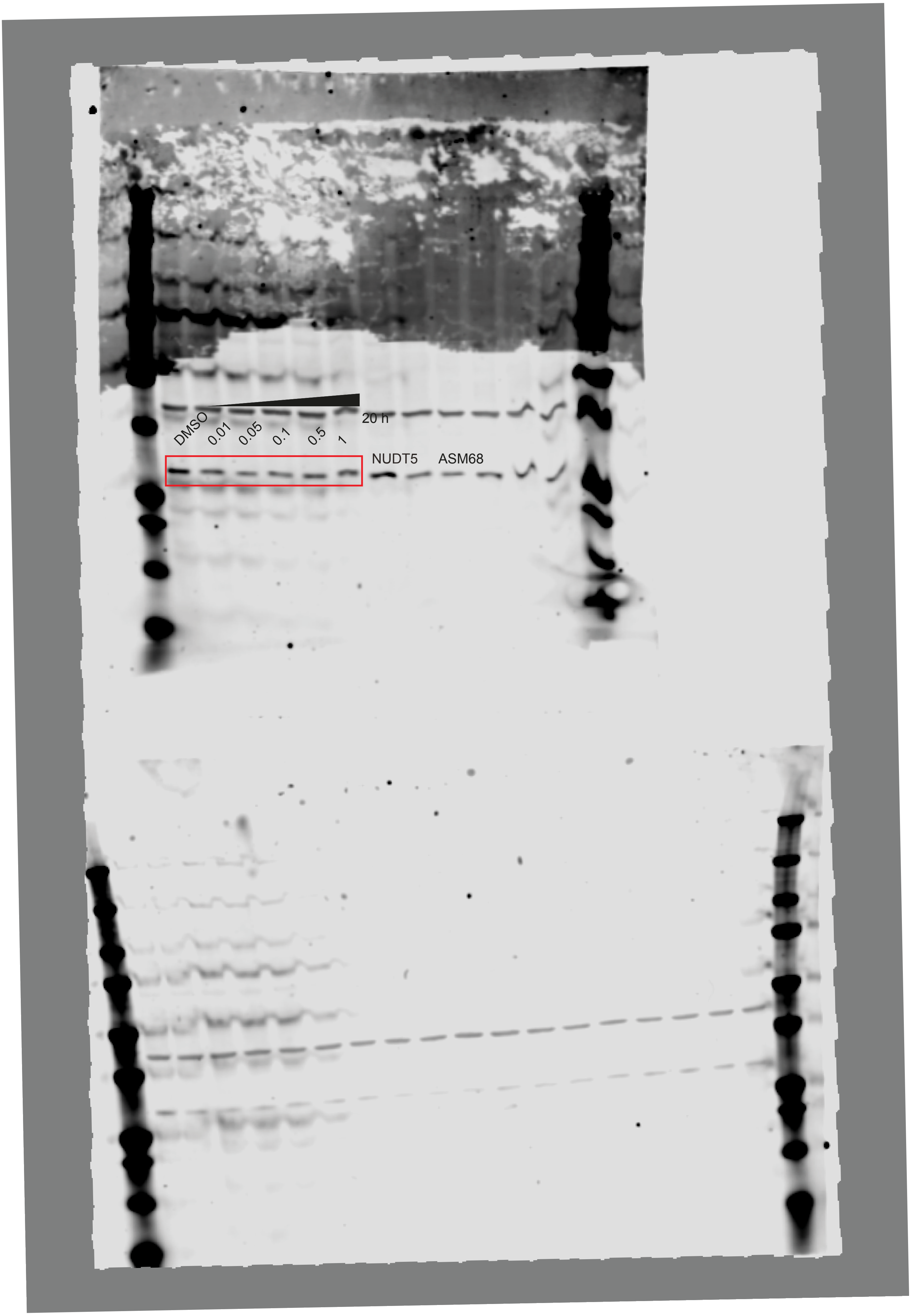

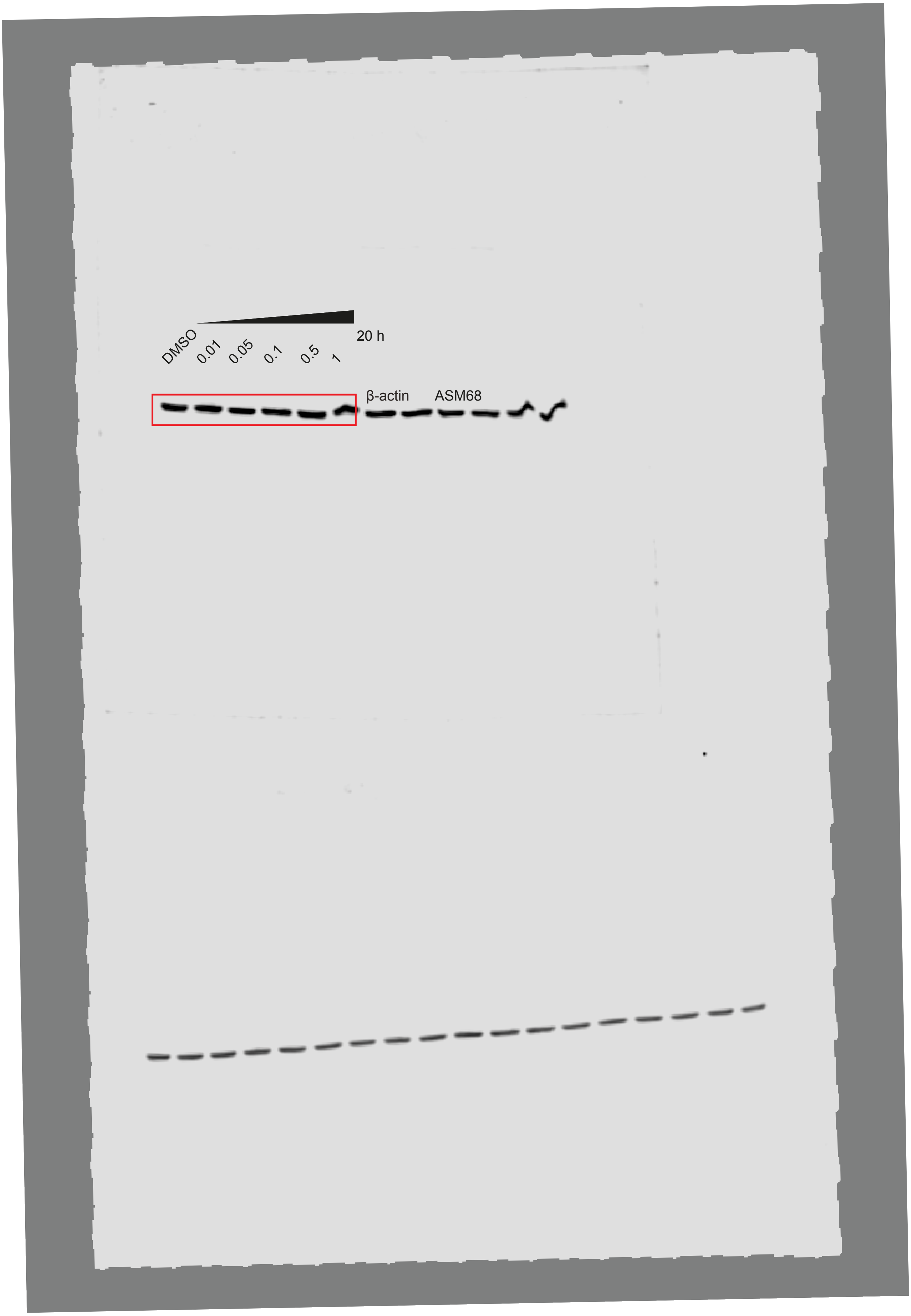
DMSO
0.01
0.05
0.1
0.5
1
20 h
β-actin
ASM68

Figure S4

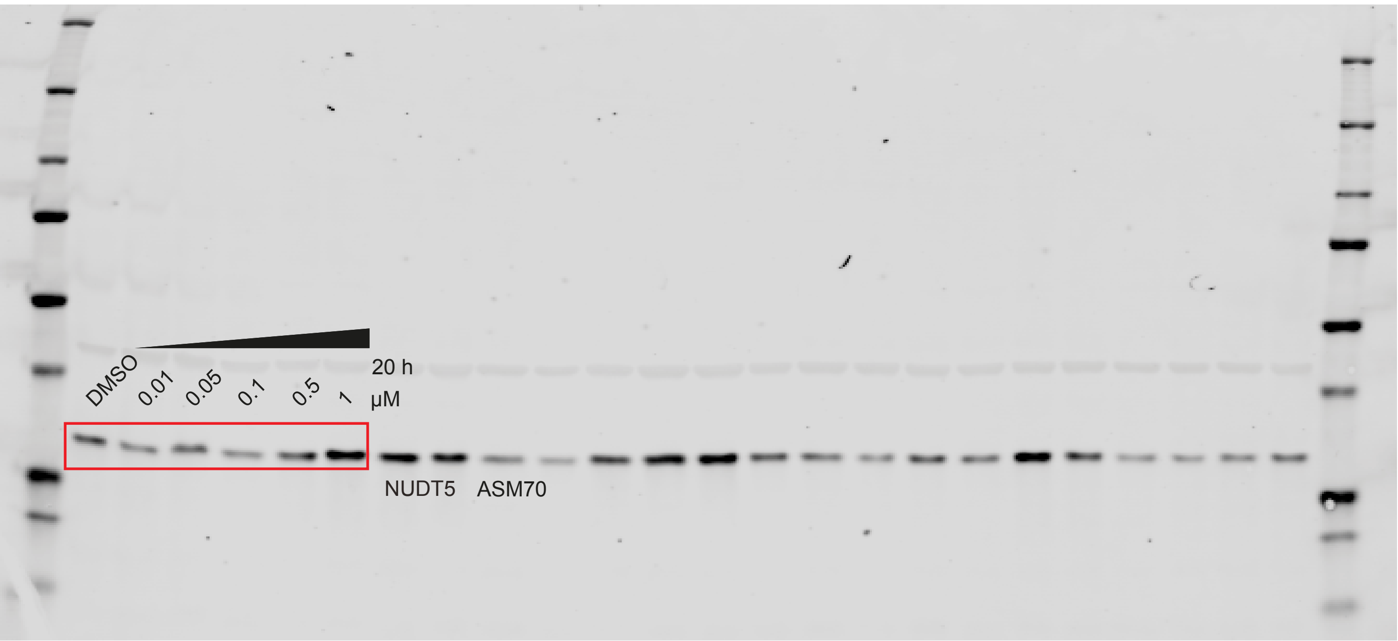
DMSO
0.01
0.05
0.1
0.5
1
20 h
µM
NUDT5
ASM70

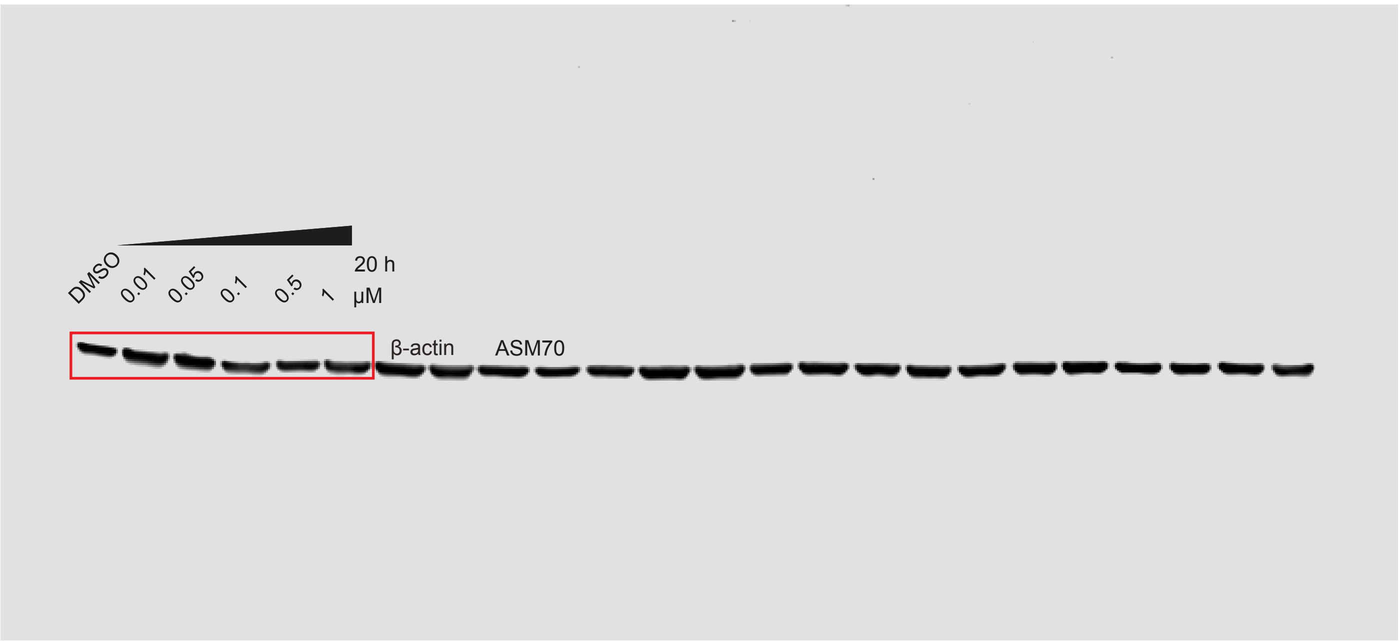
DMSO
0.01
0.05
0.1
0.5
1
20 h
µM
β-actin
ASM70

Figure S4

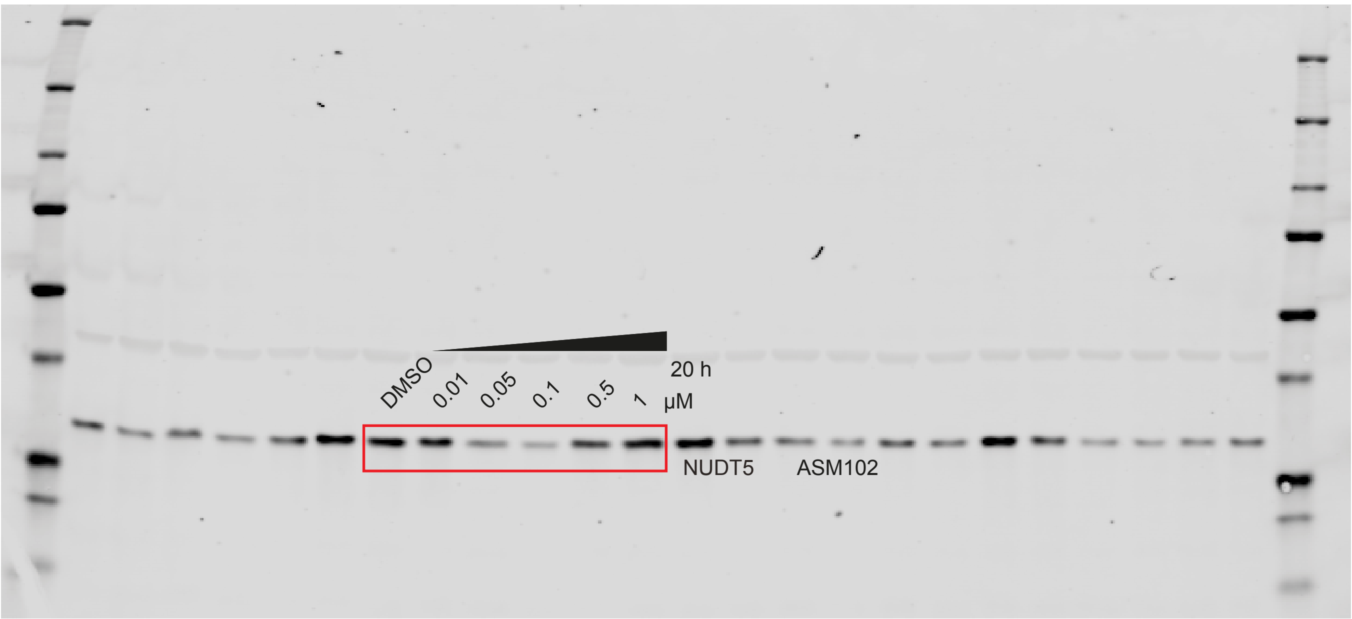
DMSO
0.01
0.05
0.1
0.5
1
20 h
μM
NUDT5
ASM102

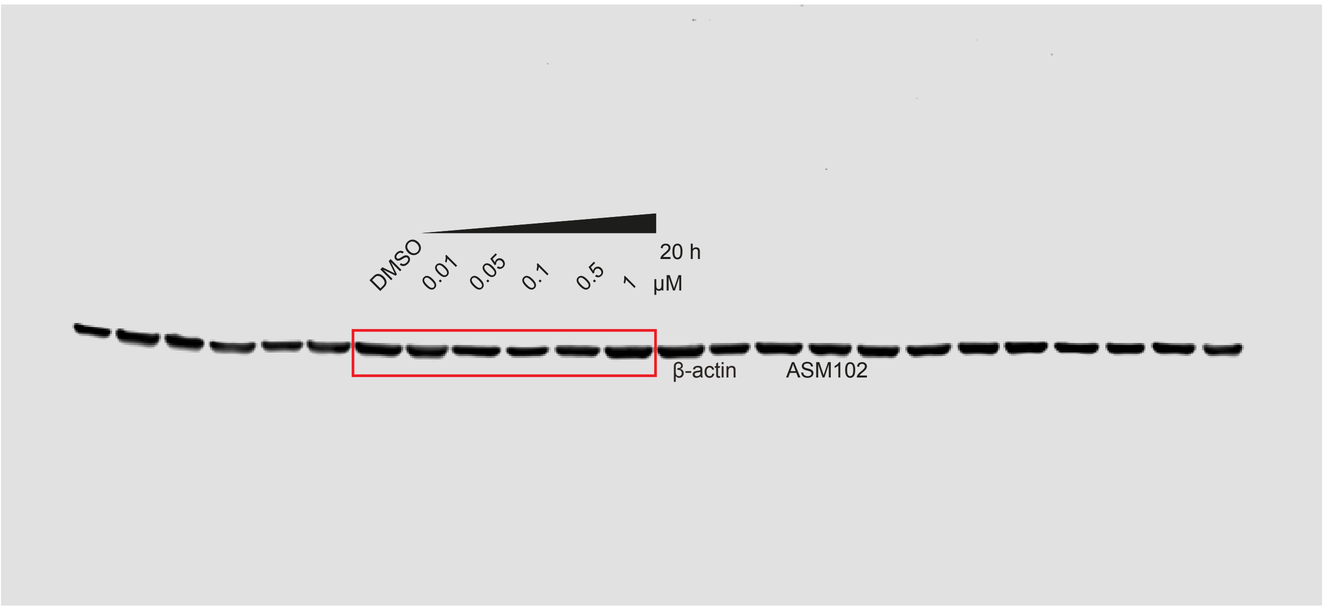
DMSO
0.01
0.05
0.1
0.5
1
20 h
μM
β-actin
ASM102

Figure S5

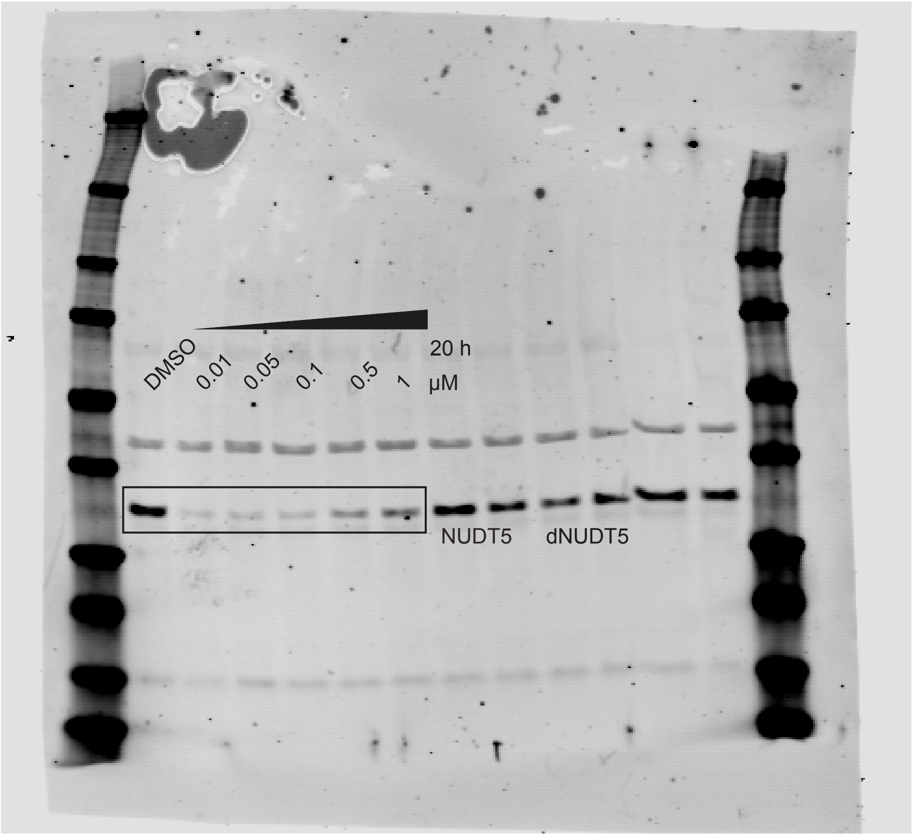

20 h
DMSO
0.01
0.05
0.1
0.5
1
μM
NUDT5
dNUDT5

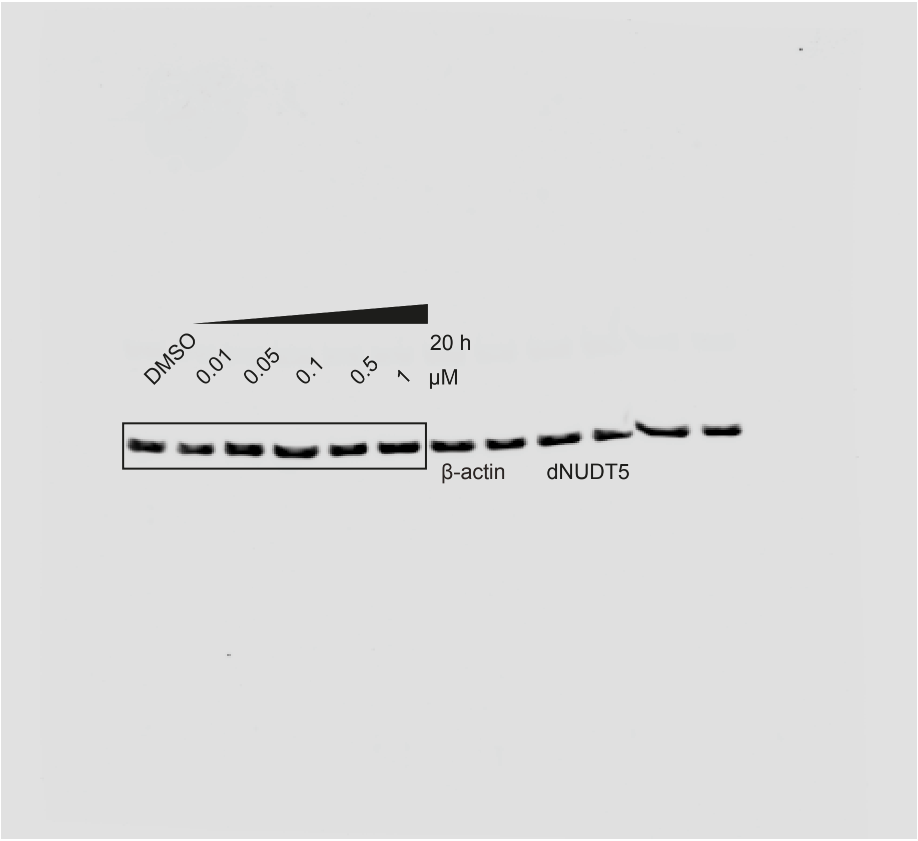

20 h
DMSO
0.01
0.05
0.1
0.5
1
μM
β-actin
dNUDT5

Figure S6A

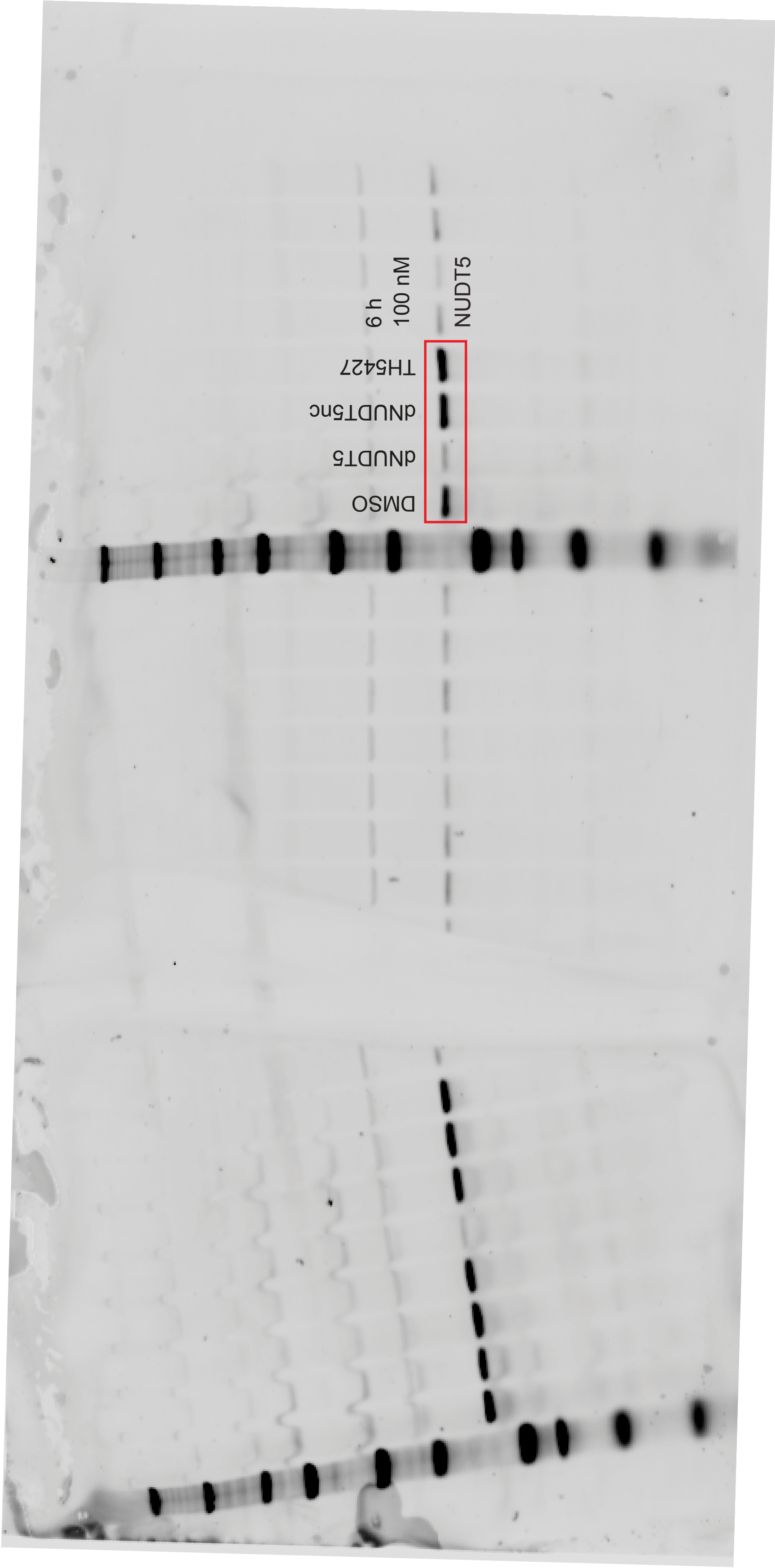

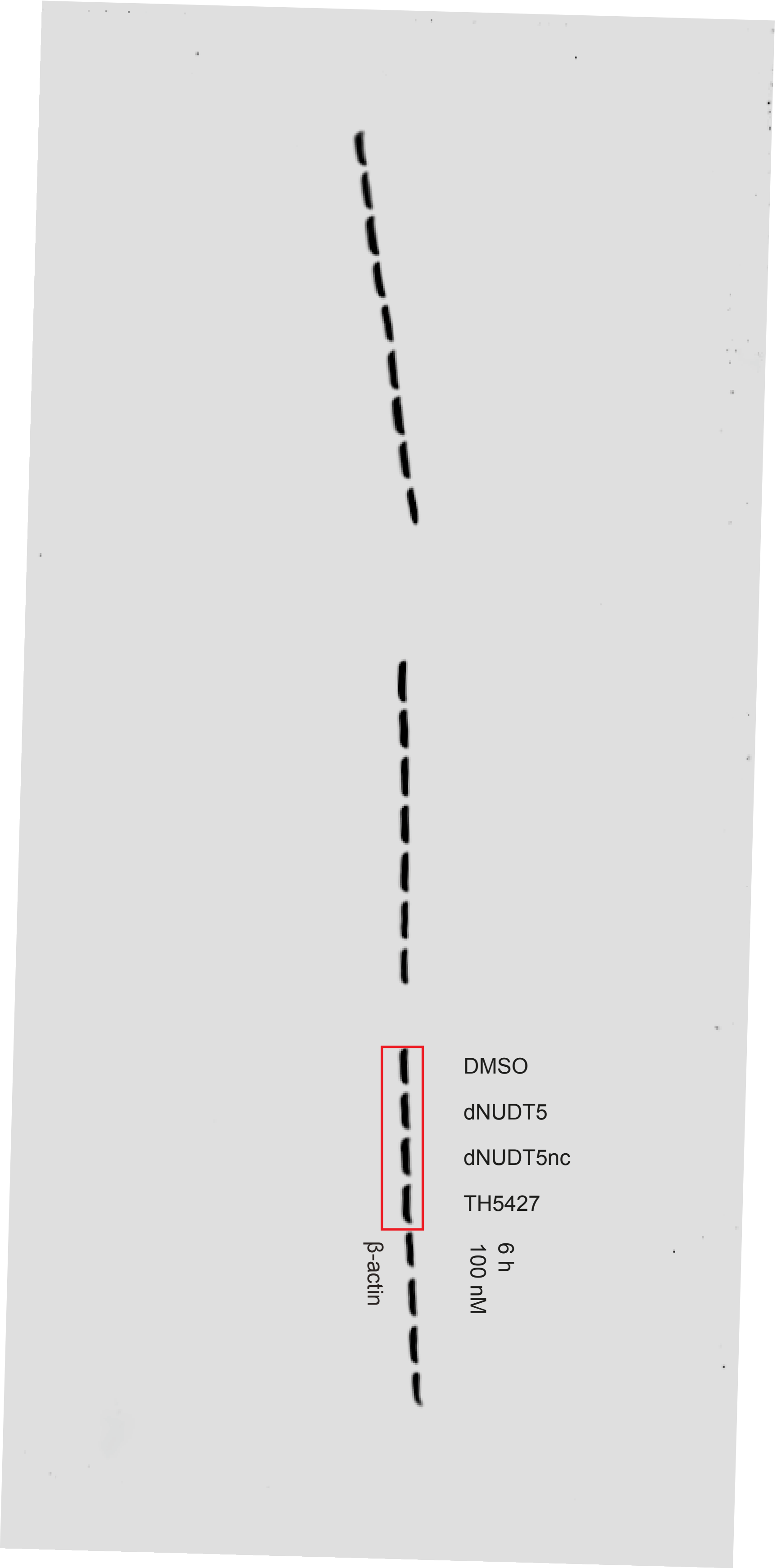

DMSO
dNUDT5
dNUDT5nc
TH5427
6 h
100 nM
β-actin

Figure S6C

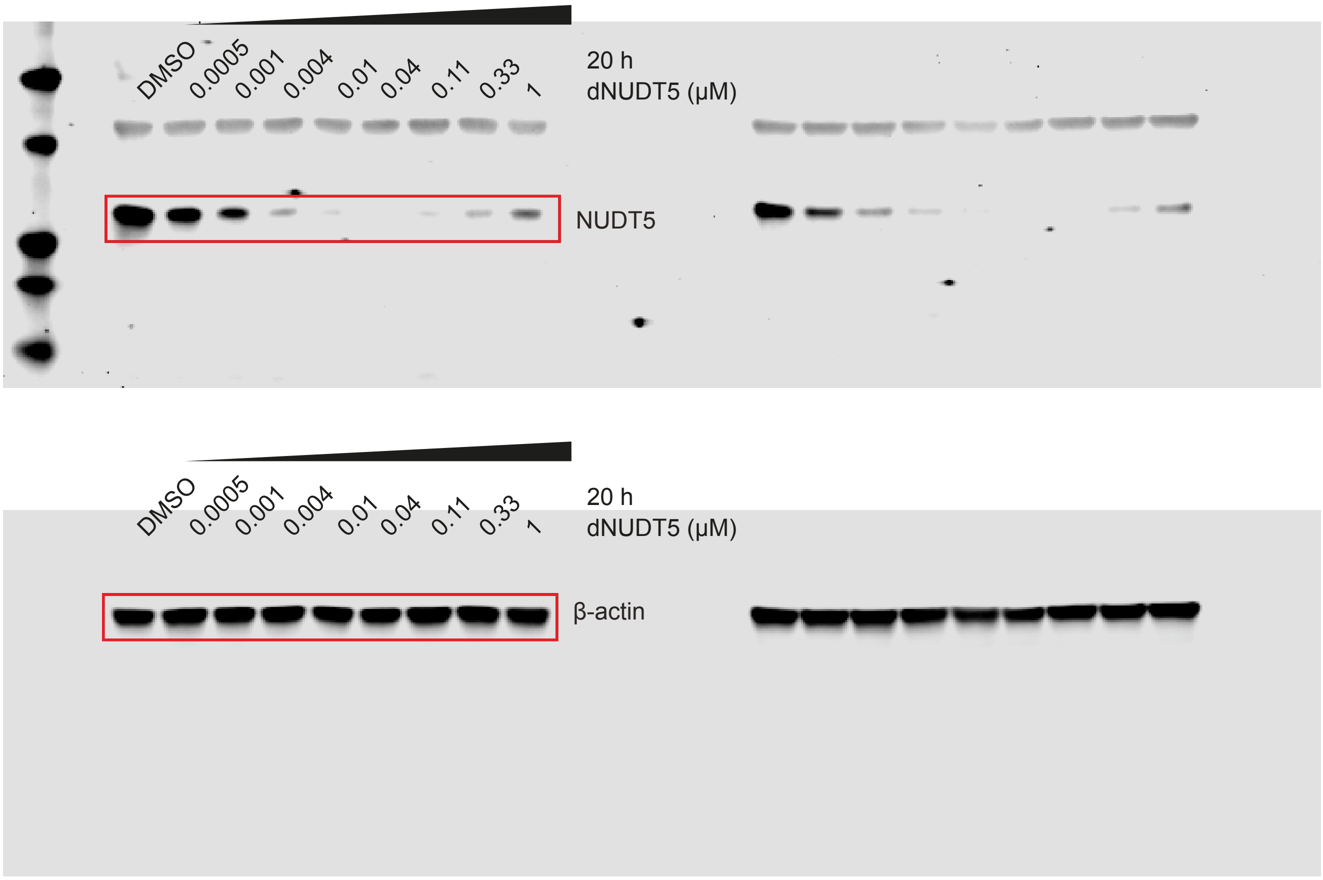

Figure S6D

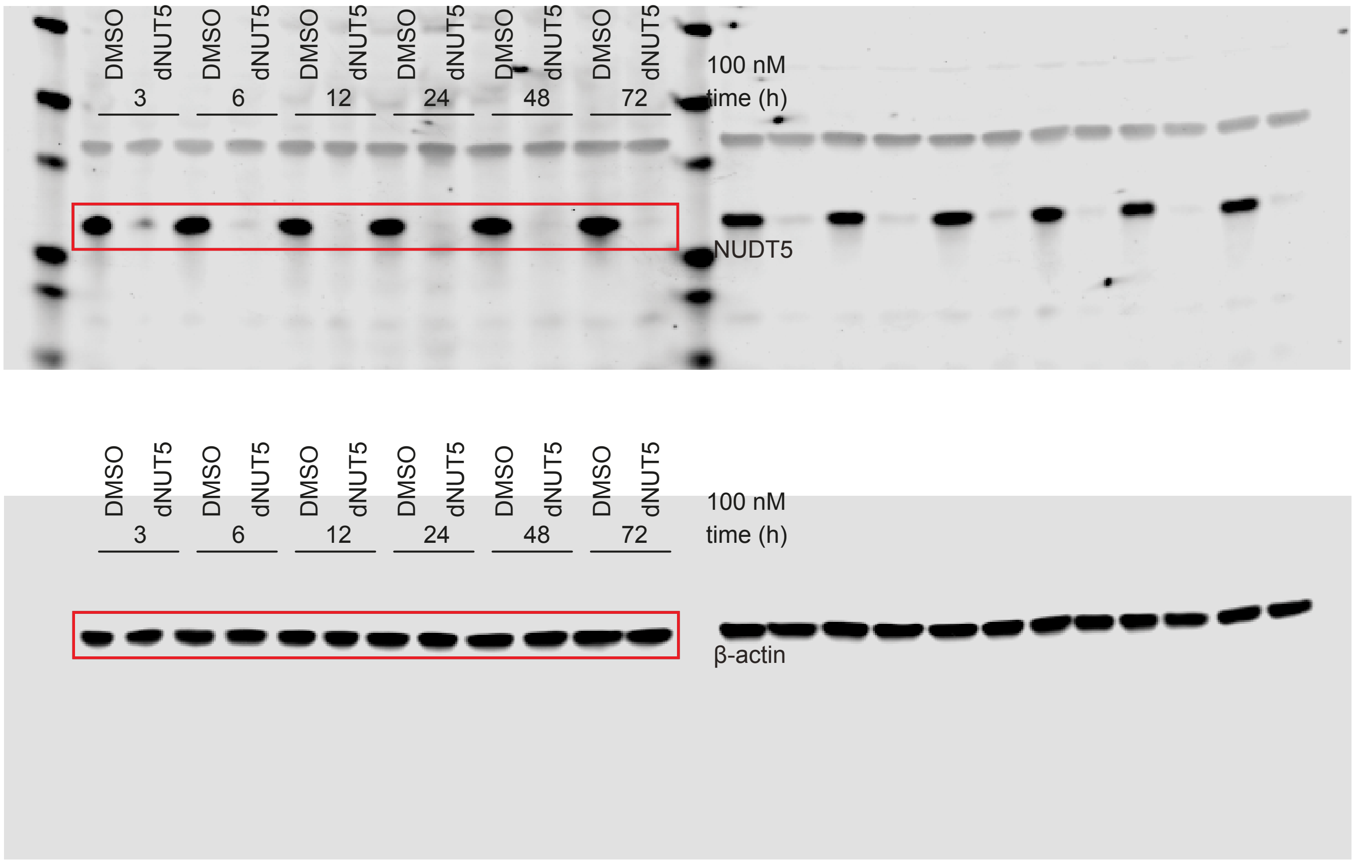

Figure S6E (top)

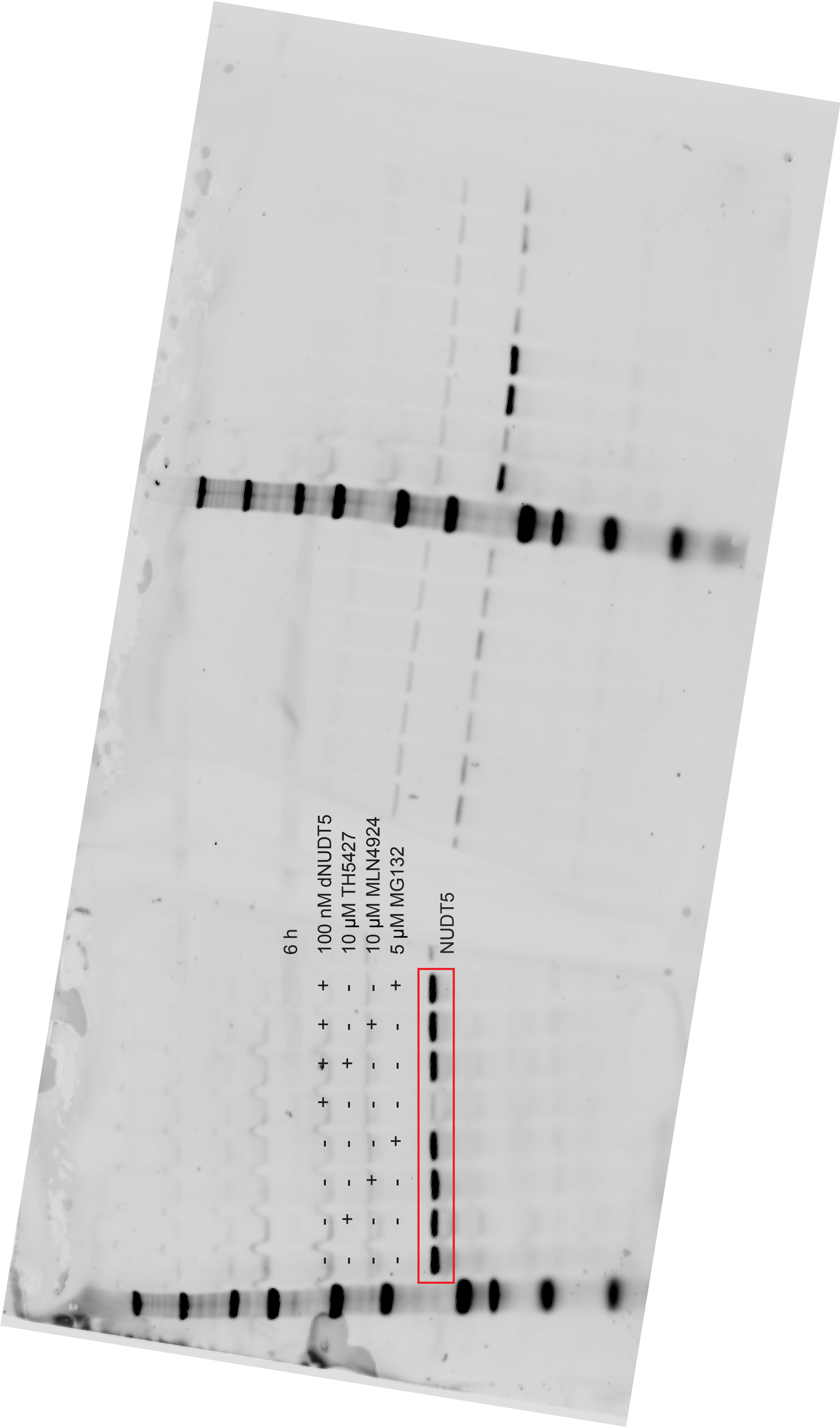

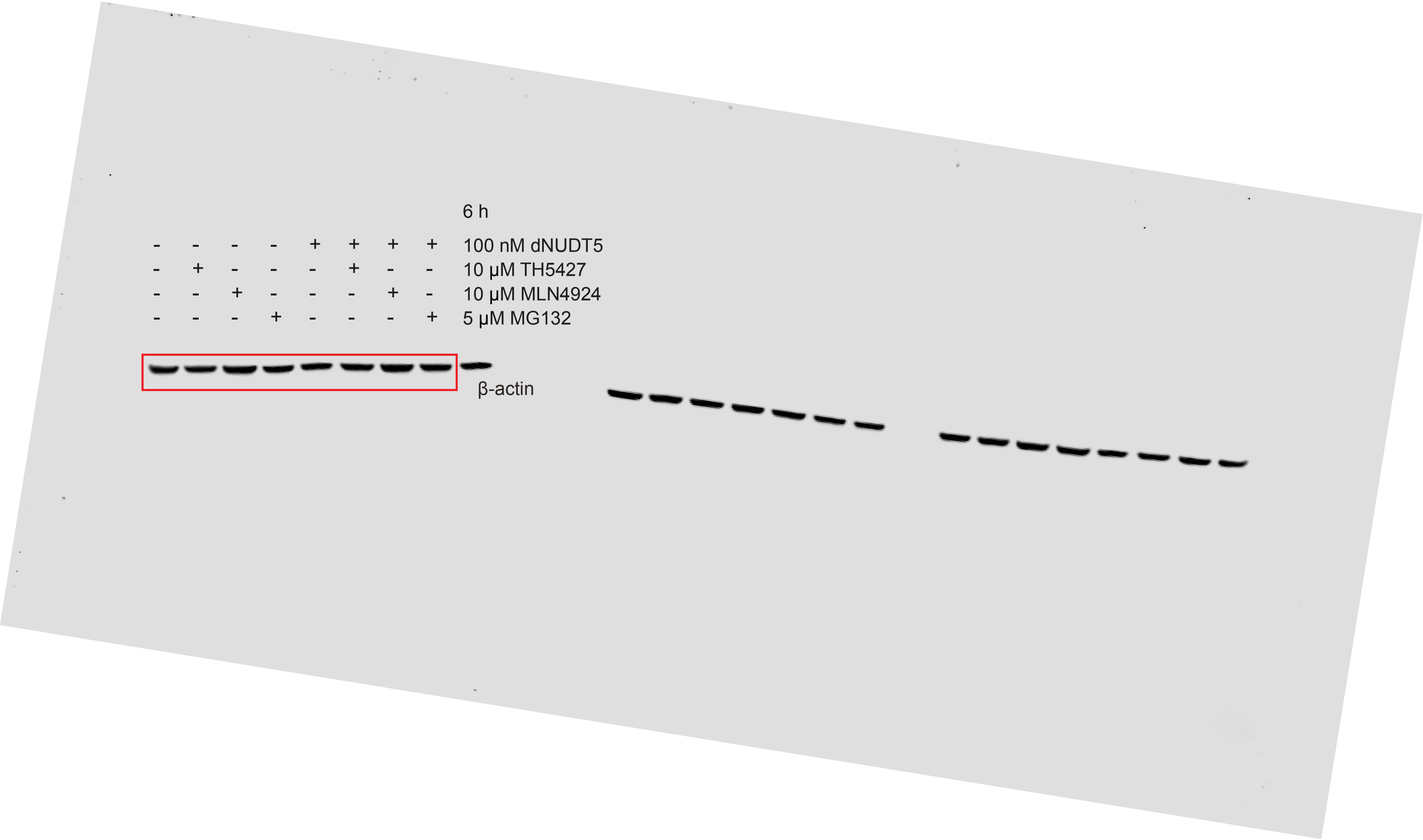

6 h
- - - - + + + + 100 nM dNUDT5
- + - - - + - - 10 µM TH5427
- - + - - - + - 10 µM MLN4924
- - - + - - - + 5 µM MG132
β-actin

Figure S6E (bottom)

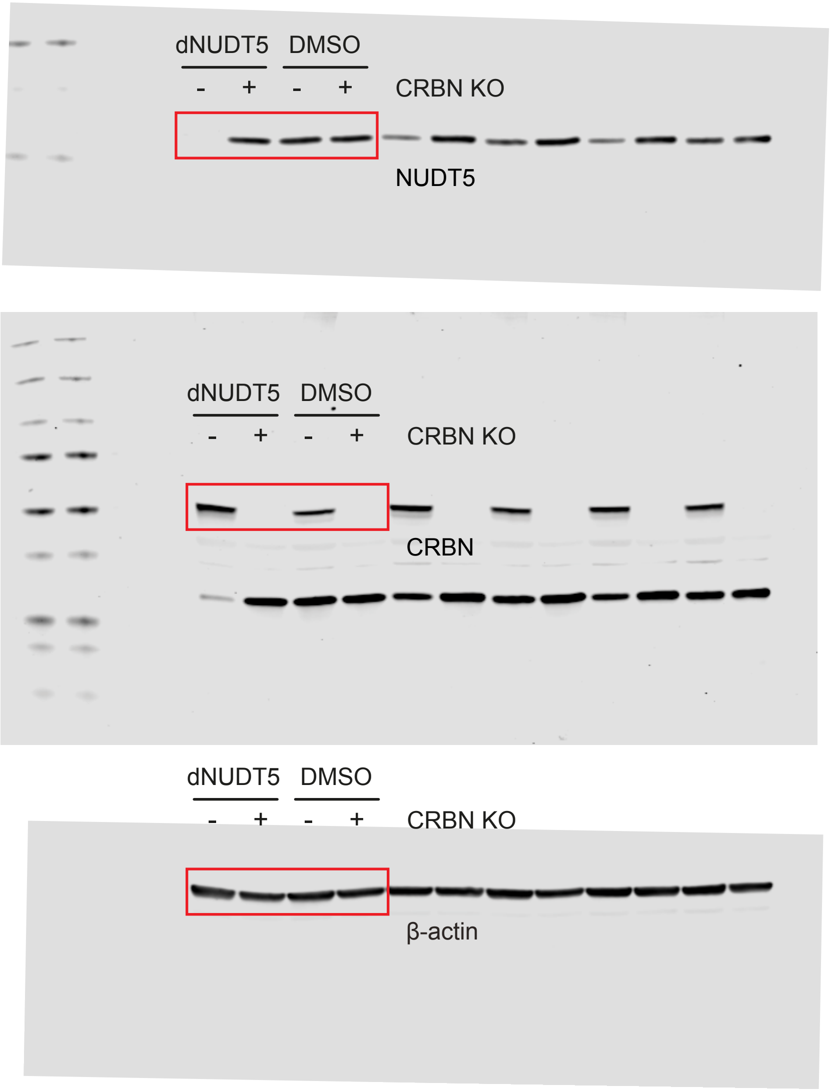

Figure S7

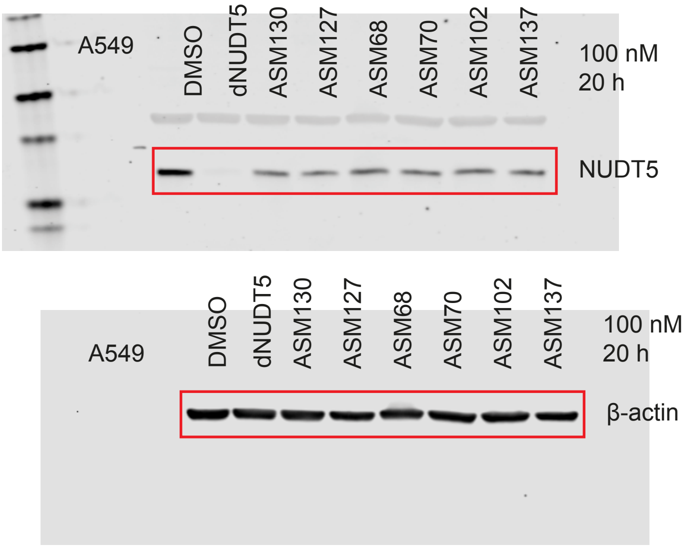

Figure S7

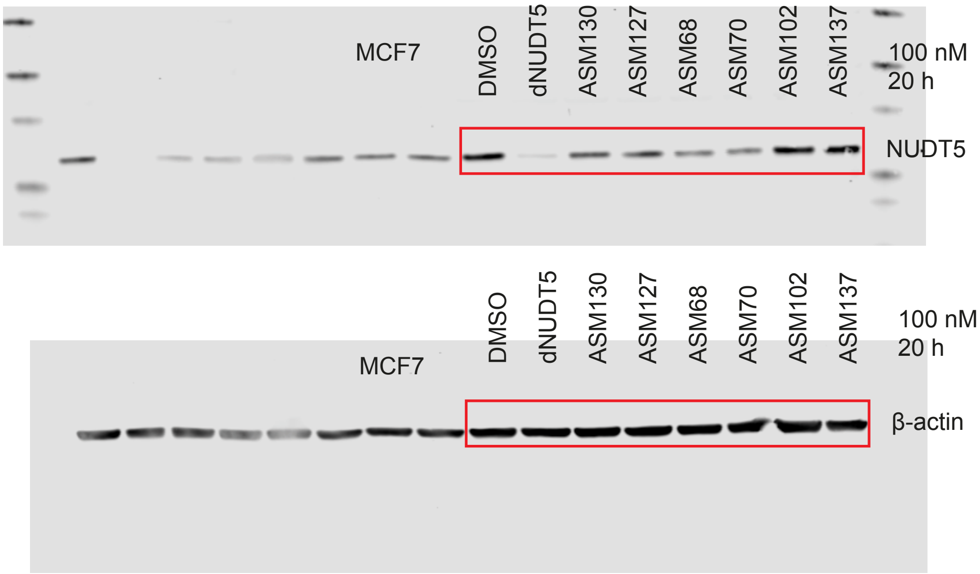

Figure S7

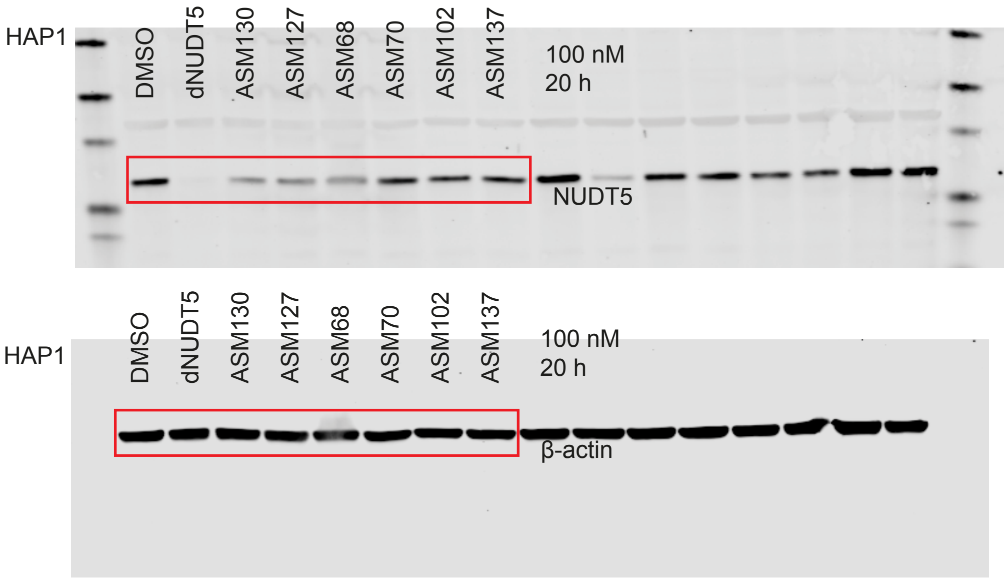

Figure S8A

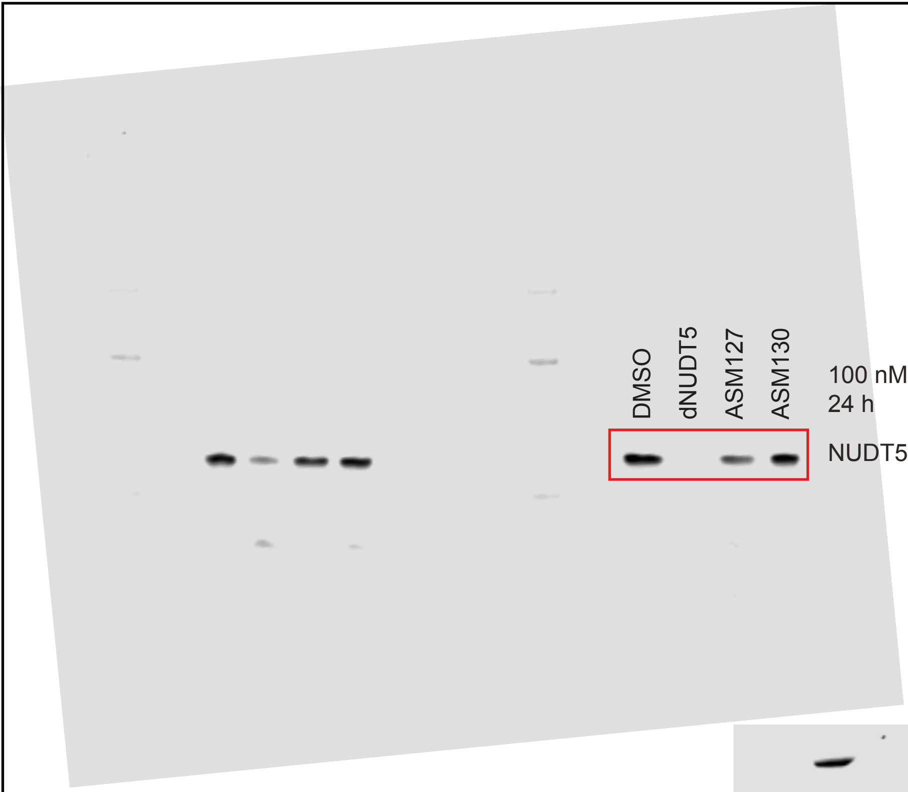
DMSO
dNUDT5
ASM127
ASM130
100 nM
24 h
NUDT5

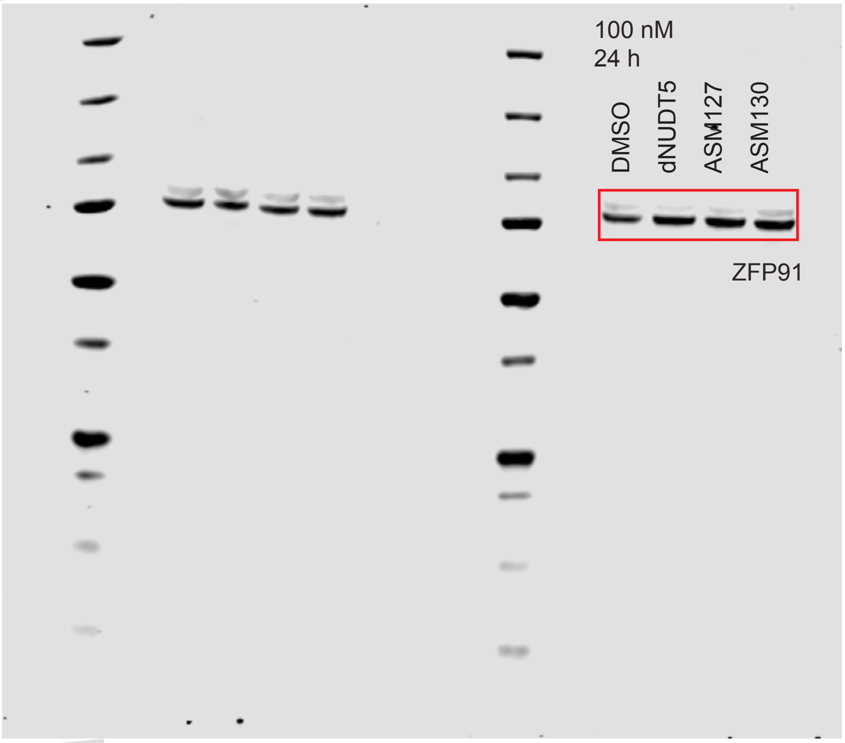
100 nM
24 h
DMSO
dNUDT5
ASM127
ASM130
ZFP91

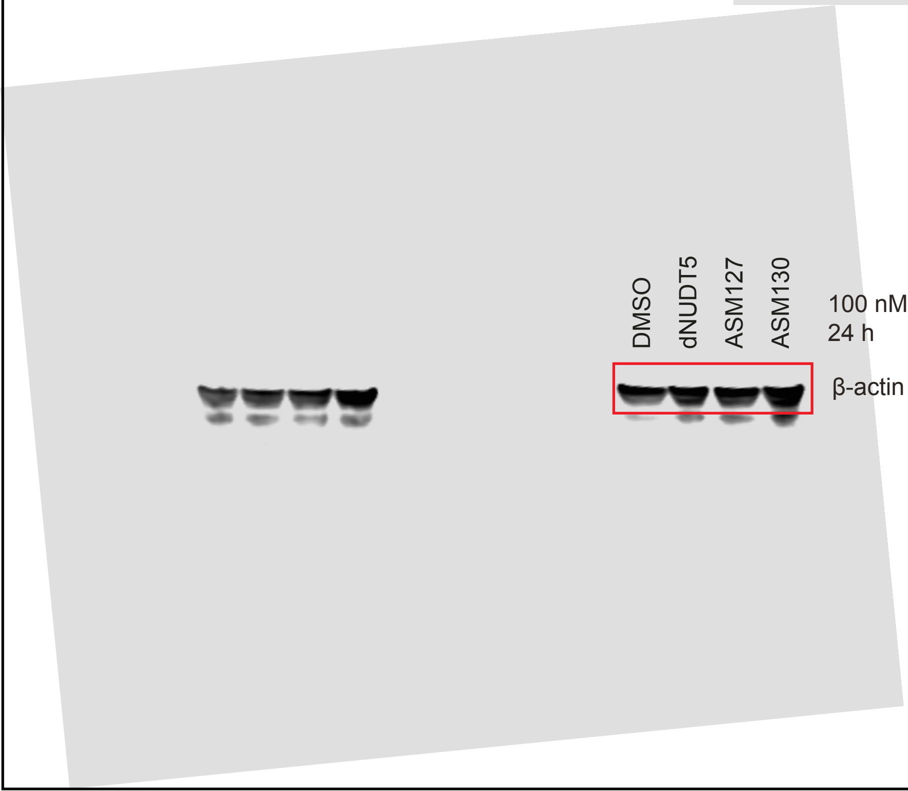
DMSO
dNUDT5
ASM127
ASM130
100 nM
24 h
β-actin

Figure S8B

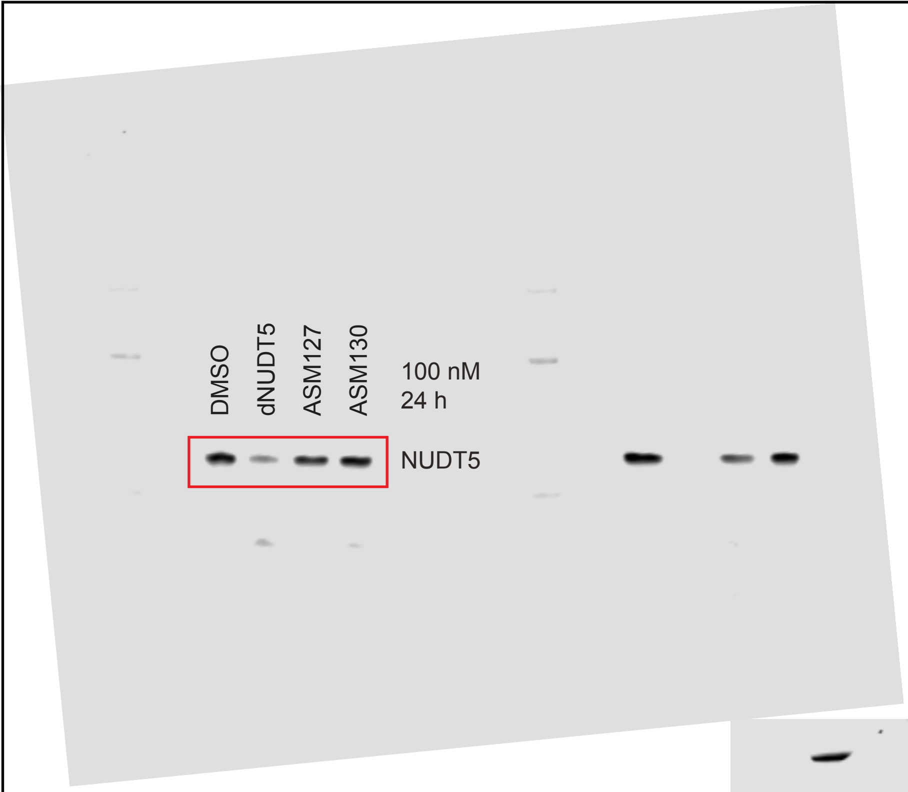
DMSO
dNUDT5
ASM127
ASM130
100 nM
24 h
NUDT5

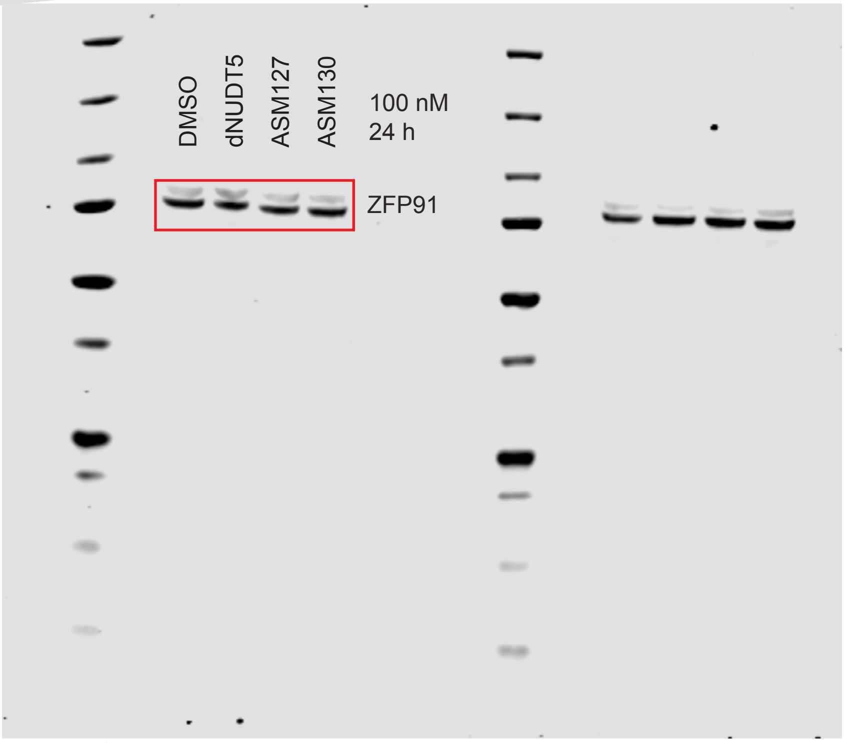
DMSO
dNUDT5
ASM127
ASM130
100 nM
24 h
ZFP91

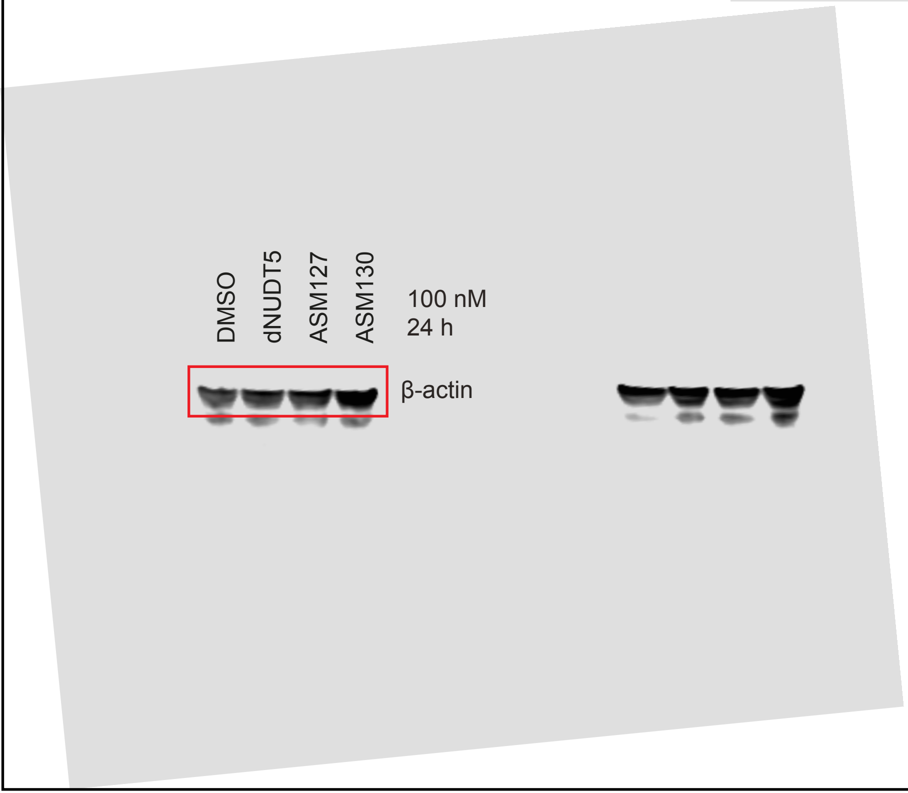
DMSO
dNUDT5
ASM127
ASM130
100 nM
24 h
β-actin
